# Novel 5-Aryl-[1,2,4]triazoloquinazoline Fluorophores: Synthesis, Comparative Studies of the Optical Properties and ICT-Based Sensing Application

**DOI:** 10.3390/molecules30224420

**Published:** 2025-11-15

**Authors:** Alexandra E. Kopotilova, Julia V. Permyakova, Ekaterina S. Starnovskaya, Tatyana N. Moshkina, Alexander S. Novikov, Pavel A. Slepukhin, Emiliya V. Nosova

**Affiliations:** 1 Organic and Biomolecular Chemistry Department, Chemical Technology Institute, Ural Federal University, Mira St. 19, Ekaterinburg 620002, Russia; 2 Institute of Chemistry, Saint Petersburg State University, 7/9 Universitetskaya Nab., Saint Petersburg 199034, Russia; a.s.novikov@spbu.ru; 3 Research Institute of Chemistry, Peoples’ Friendship University of Russia (RUDN University), 6 Miklukho-Maklaya St., Moscow 117198, Russia; 4 Postovsky Institute of Organic Synthesis, Ural Branch of the Russian Academy of Sciences, S. Kovalevskaya Str., 22, Ekaterinburg 620108, Russia

**Keywords:** nitrogen heterocycles, luminescence, push-pull quinazolines, two-photon absorption, electronic-structure calculations

## Abstract

Novel tricyclic fluorophores were obtained from 2-aryl-[1,2,4]triazolo[1,5-c]quinazoline-5(6*H*)-ones through chlorodesoxygenation and subsequent Suzuki–Imamura cross-coupling reactions. Their π-extended analogues were synthesized from 5-(4-bromophenyl)-[1,2,4]triazoloquinazolines. The structure of target fluorophores was confirmed by X-ray single crystal diffraction. Photophysical properties in solutions and solid state were studied. 5-Aminoaryl-substituted [1,2,4]triazolo[1,5-*c*]quinazolines revealed bright blue fluorescence in toluene (Φ_F_ > 95%), which can be tuned by solvent polarity, the electronic nature and rigidity of the donor fragment, the π-spacer length, and the annelation pattern. Intramolecular charge transfer (ICT) behaviour was demonstrated using both experimental and theoretical data. Distinct acid-induced spectral and fluorescence changes upon protonation were observed for diethylamino-containing derivatives, indicating their potential applicability as dual-mode (polarity and pH) molecular sensors.

## 1. Introduction

**Figure 1:**
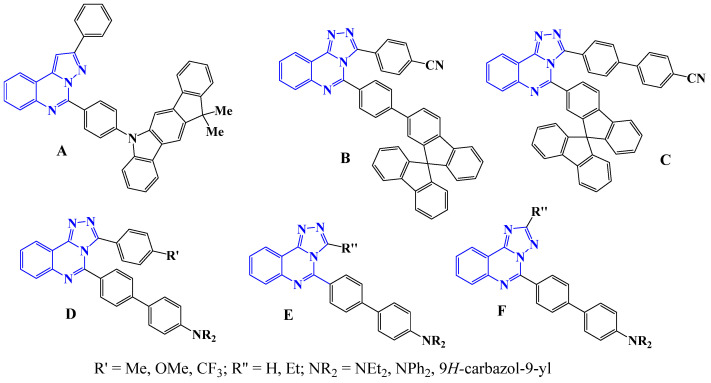
Representatives of tricyclic quinazoline-based fluorophores (electron-withdrawing azoloquinazoline core is shown in blue).

[1,2,4]Triazoloquinazolines provide a promising molecular platform for various pharmaceutical applications [1,2] and materials sciences [3]. In particular, pyrazoloquinazoline- and triazoloquinazoline-based organic compounds (**A**–**C**, Figure 1) have been developed over the past decade for light-emitting devices [4,5].

The quinazolinyl moiety represents strong electron-withdrawing part of push–pull chromophores coursing considerable intramolecular charge transfer (ICT) [6]. Notably, annelation of a triazole ring can further enhance the acceptor strength of the quinazoline core. In this context, we recently designed push–pull fluorophores based on [1,2,4]triazolo[4,3-*c*]quinazolines **D** (Figure 1) [7] and demonstrated that their photophysical properties depend on the nature of arylamino donor, the 3-aryltriazole fragment, as well as solvent polarity. X-ray analysis revealed twisted conformations of fluorophores that suppress strong intermolecular π–π stacking, thereby favouring bright solid-state and aggregation-enabled luminescence [7]. The synthesis of **D** involved hydrazone formation from 2-(4-bromophenyl)-4-hydrazinoquinazoline and arylaldehyde, oxidative cyclization of the hydrazone with bromine in glacial acetic acid at room temperature [8], and a subsequent cross-coupling reaction [7]. Later we developed a synthetic approach to 5-(4-bromophenyl)-[1,2,4]triazolo[4,3-*c*]quinazolines E and their [1,5-*c*]-isomers **F**: treatment of 2-(4-bromophenyl)-4-hydrazinoquinazoline with ortho esters under solvent-free conditions or in absolute ethanol led to the formation of [4,3-*c*]-annelated triazoloquinazolines, whereas acidic medium promoted Dimroth rearrangement and formation of [1,5-*c*] isomers [9]. The bromophenyl derivatives were functionalized by introducing aminoaryl donor fragments via palladium-catalyzed cross-coupling reactions with boronic acids or their esters; the photophysical properties of two series of 5-(4′-aminophenyl-[1,1′]-biphenyl-4-yl)[1,2,4]-triazolo-quinazoline fluorophores **E** and **F** (Figure 1) were studied. The quantum yields of triazoloquinazolines with [1,5-*c*] annelation type were found to be higher than those of their [1,2,4]triazolo[4,3-*c*]quinazoline counterparts [9].

**Scheme 1:**
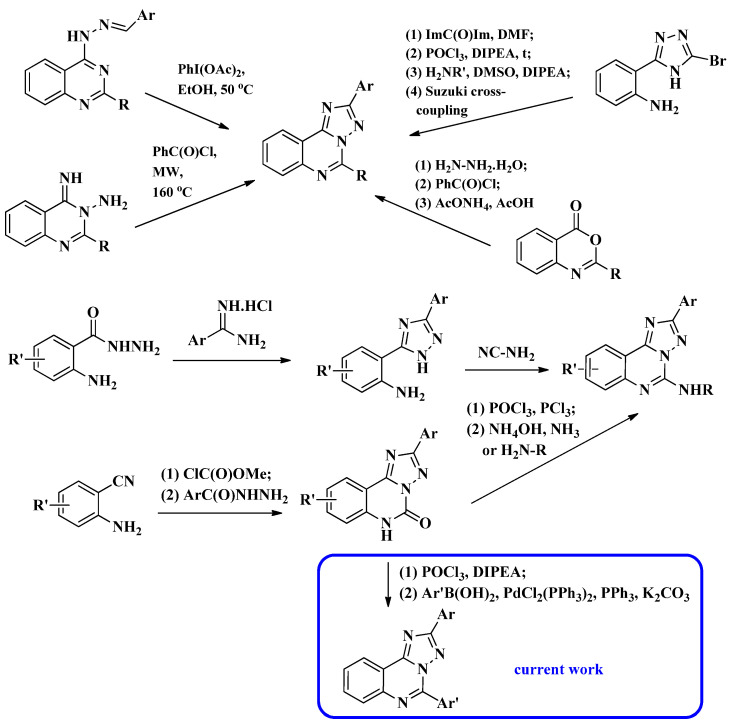
Synthetic approaches to 5-substituted 2-aryl-[1,2,4]triazolo[1,5-*c*]quinazolines.

Synthetic routes to 2-aryl-[1,2,4]triazolo[1,5-*c*]quinazolines remain limited. Formation of the [1,5-*c*] isomer of triazoloquinazoline from arylhydrazone in the presence of PhI(OAc)_2_ at 50 °C was mentioned [10] (Scheme 1); however, the evidence of structure and Dimroth rearrangement details were not discussed. Some derivatives were obtained by the condensation of anilino-substituted bromotriazole with 1,1-carbonyldiimidazole and subsequent incorporation of aryl fragment by cross-coupling reaction [11]. The synthesis of 2,5-diphenyl-[1,2,4]triazolo[1,5-*c*]quinazoline was achieved through the condensation of 4-iminoquinazoline-3-amine with benzoyl chloride [12]. Anthranylhydrazide, arylamidine, and cyanamide were used as reagents for the synthesis of 2-aryl-5-amino[1,2,4]triazolo[1,5-*c*]quinazolines [13]. The formation of limited range of 2,5-diaryl-[1,2,4]triazolo[1,5-*c*]quinazolines was achieved by the reaction of 2-aryl-3-aminoquinazolin-4-one, benzoyl chloride and ammonium acetate [14] (Scheme 1).

An approach to 2-aryl-[1,2,4]triazolo[1,5-*c*]quinazolin-5-one, based on the interaction of anthranilonitrile, methyl chloroformate as a one-carbon synthon, and arylcarbohydrazide, opens wide opportunities for variation in the substituent in position 2 [15]. Diverse modifications of position 5 via nucleophilic substitution of chlorine have been performed (Scheme 1), and some S_N_ reactions yielded valuable biologically active compounds [16]; however, incorporation of an aryl fragment into position 5 by cross-coupling reactions in 2-aryl-5-chloro-[1,2,4]triazolo[1,5-*c*]quinazolines has not yet been investigated. Nonetheless, this creates a pathway to a new class of push–pull fluorophores. Additionally, considering the aforementioned points, spectrofluorimetric data for [1,2,4]triazoloquinazoline derivatives are scarce, and systematic structure–property correlations have not been previously studied in depth.

Accordingly, we present the design and synthesis of a series of push–pull [1,2,4]triazoloquinazolines bearing an aryl donor directly attached at C-5 or linked through a π-spacer. Novel 2,5-diaryl-[1,2,4]triazolo[1,5-*c*]quinazoline tricyclic fluorophores were obtained by a concise two-step method from 2-aryl-[1,2,4]triazolo[1,5-*c*]quinazolin-5(6*H*)-ones (Scheme 1), via chlorodeoxygenation followed by Suzuki–Miyaura cross coupling with arylboronic acids or their esters. Their π-extended analogues were prepared via functionalization of 5-(4-bromophenyl)-[1,2,4]triazoloquinazoline. All structures were confirmed by spectroscopic methods, including single crystal X-ray diffraction. Photophysical properties of these compounds were carefully studied experimentally and theoretically using DFT calculations. The influence of conjugation length, annelation mode, and substituent electronic nature was determined in obtained series and by comparison with already reported triazoloquinazolines **E** and **F** (Figure 1) [9]. Solvatochromic behaviour and acid-responsive properties were investigated for a series of [1,2,4]triazoloquinazolines, demonstrating their potential as polarity-sensitive and pH-responsive molecular sensors.

## 2. Results

### 2.1. Synthesis

**Scheme 2:**
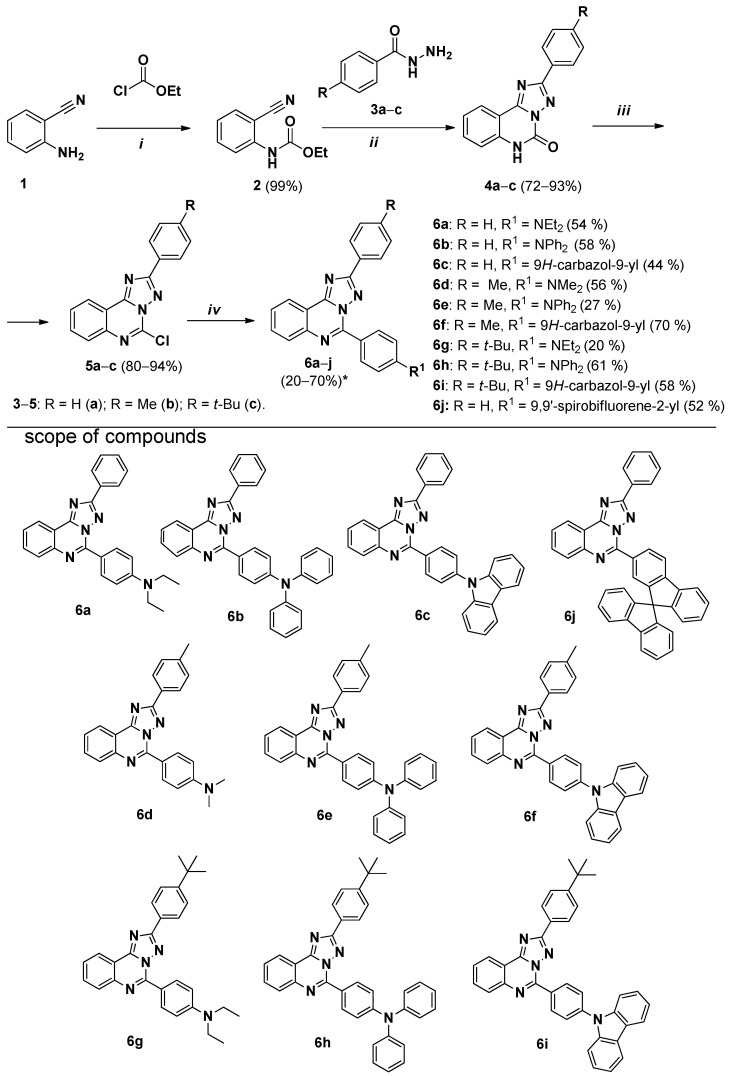
Synthesis of quinazolines **6a**–**j**. *Reagents and conditions*: (***i***) K_2_CO_3_, THF, 85 °C, 24 h; (***ii***) DMF, 120 °C, 12 h; (***iii***) POCl_3_, DIPEA, 110 °C, 20 h; (***iv***) corresponding arylboronic acid or ester, PdCl_2_(PPh_3_)_2_, PPh_3_, K_2_CO_3_, toluene, EtOH, H_2_O, argon, 85 °C, 14–30 h. * unoptimized yields.

A synthetic strategy toward 2,5-diaryl-[1,2,4]triazolo[1,5-*c*]quinazolines **6a**–**j** is illustrated in Scheme 2. Starting from 2-aminobenzonitrile **1**, treatment with ethyl chloroformate gave N-(2-cyanophenyl)carbamate **2**, which then underwent reaction with corresponding arylhydrazides **3a**–**c** to afford key intermediates, namely 2-aryl-[1,2,4]triazolo[1,5-*c*]quinazolin-5-ones **4a**–**c** [11]. This process involves nucleophilic substitution of the ethoxy group in intermediate **2** by the hydrazide fragment of arylhydrazide **3**, followed by nucleophilic attack on the cyano group and subsequent cyclization via triazole ring closure (involving nucleophilic addition and elimination of water), as schematized in Figure S1, SI. Upon prolonged reflux of quinazolinones **4a**–**c** in phosphorus oxychloride in the presence of N,N-diisopropylethylamine (DIPEA), the corresponding 5-chloro derivatives **5a**–**c** were obtained [11]. Further Suzuki–Miyaura cross-coupling reactions with arylboronic acids or their esters resulted in the desired final products **6a**–**j**, although yields varied broadly from 20% to 70% in the absence of optimization.

**Scheme 3:**
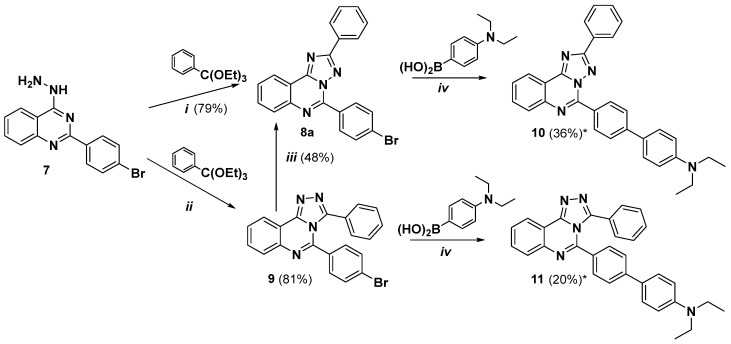
Synthesis of 2-phenyl[1,2,4]triazolo[1,5-*c*]quinazoline **10** and 3-phenyl[1,2,4]triazolo[4,3-*c*]quinazoline **11.** Reagents and conditions: (***i***) CH_3_COOH, reflux, 16 h; (***ii***) reflux, 16 h; (***iii***) CH_3_COOH, reflux, 20 h; (***iv***) PdCl_2_(PPh_3_)_2_, PPh_3_, K_2_CO_3_, toluene, EtOH, H_2_O, argon, 85 °C, 11–16 h. * unoptimized yields.

Additionally, we synthesized 2-phenyl[1,2,4]triazolo[1,5-*c*]quinazoline **10** (Scheme 2), featuring an extended π-conjugation system, and its [4,3-*c*]-annelated counterpart **11** (Scheme 3), to investigate the effect of conjugation length and annulation type on the photophysical properties of the [1,2,4]triazoloquinazoline series. It was demonstrated that the intermediate **8a** could be formed by refluxing 4-hydrazinoquinazoline **7**, prepared as described previously [8], with triethoxymethylbenzene in acetic acid for 16 h (Scheme 3). In contrast, the [4,3-*c*]-annulated counterpart isomer **9** was obtained in good yield from the same reagents upon refluxing either under neat conditions or in absolute ethanol. In this case, the setup included a drying tube filled with calcium chloride to protect the reaction mixture from atmospheric moisture. Notably, conversion of compound **9** into its [1,5-*c*] isomer **8a** was observed upon prolonged heating under acidic conditions via a Dimroth rearrangement, as depicted in Figure S2, SI [9].

**Scheme 4:**
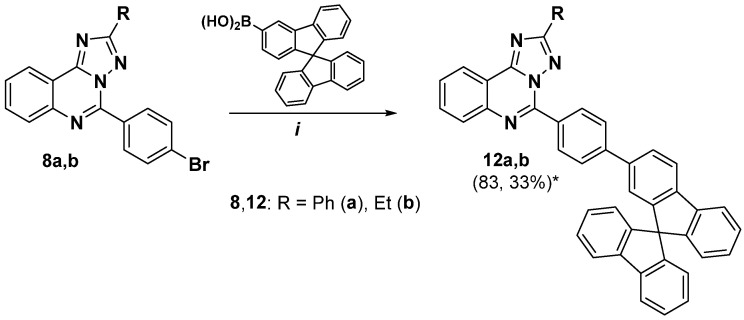
Synthesis of 9,9′-spirobifluorene-substituted [1,2,4]triazolo[1,5-*c*]quinazolines **12a**,**b**. Reagents and conditions: (***i***) PdCl_2_(PPh_3_)_2_, PPh_3_, K_2_CO_3_, toluene, EtOH, H_2_O, argon, 85 °C, 14 h. * unoptimized yields.

Finally, 9,9′-spirobifluorene-containing 2-phenyl- and 2-ethyl-[1,2,4]triazolo[1,5-*c*]quinazolines **12a** and **12b** were obtained from the corresponding bromo derivatives **8a**,**b** [9], Scheme 4.

The structures of the intermediate compounds were confirmed by ^1^H, ^13^C NMR spectroscopy and electron ionization mass spectrometry (EI-MS), see Figures S3–S12. Full characterization of the target compounds was performed using ^1^H and ^13^C NMR spectroscopy, EI-MS, and elemental analysis, see Figures S14–S27. Additionally, a ^1^H–^1^H NOESY experiment was conducted for one representative compound to assist in the assigning NMR signals to specific hydrogen atoms (Figure S19b).

**Figure 2:**
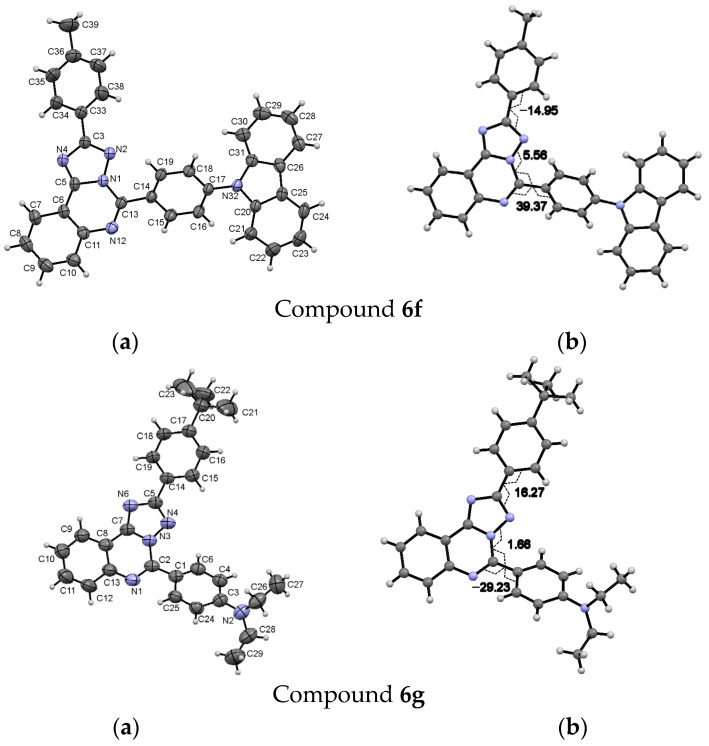

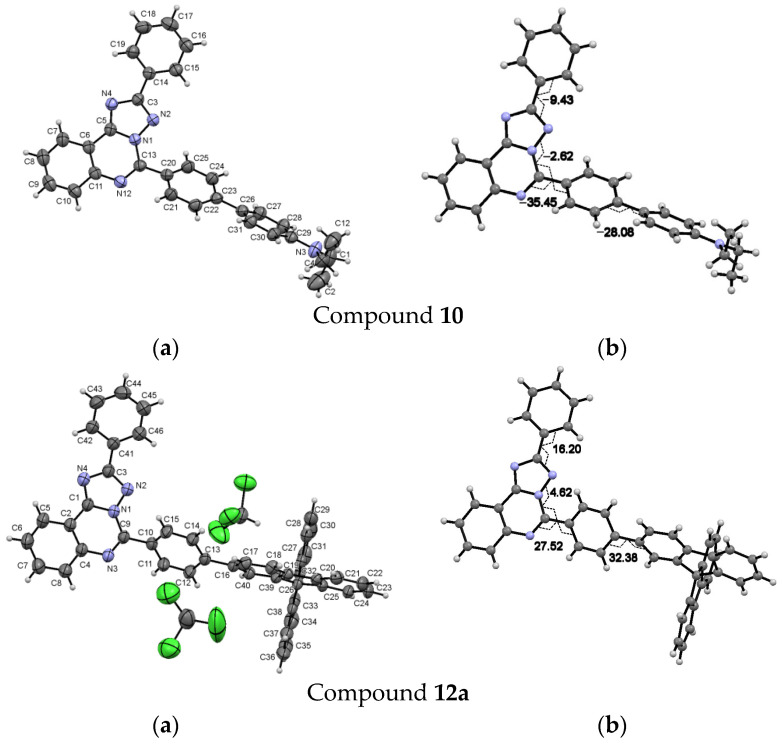
(**a**) Molecular structure of compounds **6f**, **6g**, **10**, and **12a** in the thermal ellipsoids of 50% probability). (**b**) Selected torsion angles of compounds **6f**, **6g**, **10**, and **12a**.

According to X-ray diffraction analyses, Figure 2, Tables S1–S8, performed on single crystals of compounds **6f**, **6g**, **10**, and **12a**, the molecular structures showed the expected connectivity and spatial arrangement.

According to XRD data, the compound **6f** is crystallized in the centrosymmetrical space group as solvate with CHCl_3_ (1:1). The molecule of CHCl_3_ is disordered into two positions and forms the shortened C-H…N contacts with the nitrogen’s atoms of the heterocycle. The geometry of the heterocyclic compound (the bond distances and angles) is near to the expectation. The heterocyclic core of the molecule is planar, the plane of the phenylene bridge in the molecule is turned toward the plane of the heterocycle on the angle 40°, the plane of the carbazole’s moiety is oriented approximately in the plane of the tricyclical system. The molecular crystal packing is layered. The layers are oriented in the plane (1 0 0). The distances between the molecular layers 3.45–3.50 Å and significantly shortened π-π-contacts (lesser when 3.35 Å) between molecules are not observed.

The compound **6g** crystallizes in a centrosymmetric space group of the triclinic system. The bond lengths and angles in the molecule are close to the expected values. The heterotricyclic core is planar within 0.04 Å. The *t*-Bu-C_6_H_4_ group is approximately coplanar with the heterocycle. The diethylaminophenylene group exhibits disorder of the Et and C_6_H_4_ substituents over two positions, with significant dihedral angles (30° and 40°) between the C_6_H_4_ moiety and the heterocycle (Figure 2). The C_sp_^2^–N and C_sp_^3^–N interatomic distances for the N(2) atom are well resolved. The shortened intermolecular contacts in the crystal primarily involve the disordered moieties, making precise evaluation of their geometry challenging.

The compound **10** crystallizes in a centrosymmetric space group of the monoclinic system. The heterocyclic moiety is planar, and the phenyl substituent is approximately coplanar with the heterocycle. The N atom of the diethylamine group has a planar environment and shows strong conjugation with the C_6_H_4_ moiety (C_sp_^2^–N distance 1.39 Å). In the crystal, shortened centrosymmetric intermolecular π–π contacts are observed between neighbouring heterocycles.

The compound **12a** crystallizes in a centrosymmetric space group of the triclinic system, with two CHCl_3_ molecules in the lattice. The heterocyclic moiety is planar; the phenyl substituent is approximately coplanar with the heterocycle (dihedral angle 16°), while the C_6_H_4_ substituent is rotated toward the plane of the heterocycle by 28°. In the crystal, shortened centrosymmetric intermolecular π–π contacts are observed between adjacent heterocycles, with interatomic distances of 3.4 Å.

### 2.2. UV-Vis and Fluorescence Spectroscopy

The photophysical properties of the synthesized quinazolines **6a**–**j**, **10**, **11**, and **12a**,**b** were investigated in two solvents of different polarity, toluene (ε_r_ = 2.39 [12]), and acetonitrile (MeCN) (ε_r_ = 36.64 [12]), at room temperature (see SI, Figure S28), and the corresponding data are summarized in Table 1.

**Figure 3:**
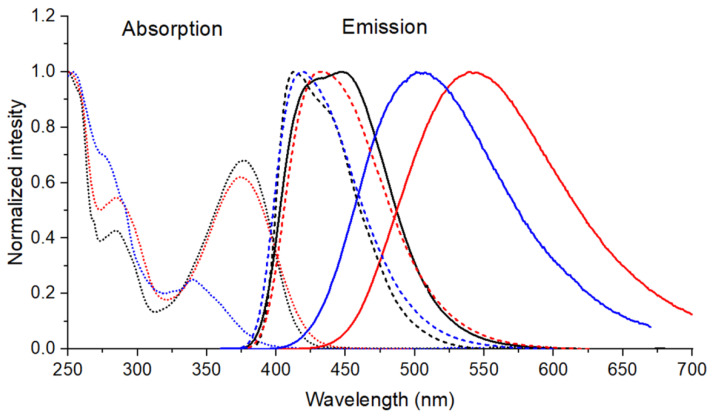
Normalized absorption spectra in MeCN (dotted line), emission spectra in toluene (dashed line), and emission spectra in MeCN (solid lines) of compounds **6a** (black line), **6b** (red line), and **6c** (blue line).

5-Aminoaryl-substituted series of 2-aryl-[1,2,4]triazolo[1,5-*c*]quinazolines **6a**–**i** (Table 1) exhibit long-wavelength absorption bands in the range of 325–425 nm and emission maxima between 420 and 459 nm in toluene. It was found that solvent polarity has little effect on the absorption spectra, Table 1; however, a pronounced bathochromic shift in the emission maximum is observed upon going from toluene to acetonitrile (Figure 3), indicating the stabilization of intramolecular charge transfer state (ICT state) by polar media [18,19]. This solvent-dependent emission shift is more significant for (diphenylamino)phenyl and (carbazolyl)phenyl derivatives **6b**,**c**,**e**,**f**,**h**,**i** than for Alk_2_N-counterparts **6a**,**d**,**g**, Figure 3, predicting stronger ICT character and a larger excited-state dipole moment, along with enhanced solvatochromism (greater red shifts and Stokes shifts in more polar solvents).

**Figure 4:**
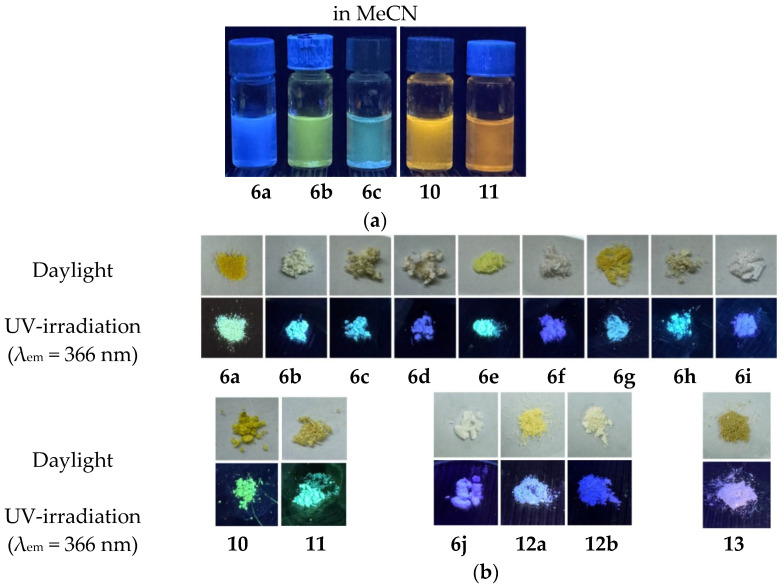
(**a**) Photograph of solutions of the compounds **6a**–**c**, **10**, and **11** in acetonitrile under UV-irradiation (λ_em_ = 366 nm). (**b**) Photograph of the compounds **6a**–**j**, **10**, **11**, and **12a**,**b** in the solid state (powder) in daylight and under UV-irradiation (λ_em_ = 366 nm).

Notably, the nature of the aryl substituent at the triazole ring of considered compounds **6a**–**i** has little influence on the spectrofluorimetric properties, as summarized in Table 1. In contrast, the electronic character of the aryl donor fragment at position 5 of the [1,2,4]triazolo[1,5-*c*]quinazoline core significantly influences the photophysical behaviour. For instance, the absorption maxima of carbazolyl-substituted derivatives **6c**, **6f**, and **6i** are hypsochromically shifted by approximately 30–40 nm relative to their Et_2_N- and Ph_2_N-substituted counterparts, as seen in Figure 3 and Table 1. The emission colour turns from blue to yellow when altering the electron donor fragment from dialkylamino- to carbazolyl- and further to diphenylamino-group in acetonitrile (Figure 4a).

Aminoaryl-substituted compounds **6a**–**i** exhibit strong blue luminescence (Table 1) with fluorescence quantum yields (Φ_F_) exceeding 95% in toluene and above 52% in acetonitrile. It was shown that the presence of a 9,9′-spirobisfluorene residue at position 5 of the [1,2,4]triazolo[1,5-*c*]quinazoline core (compound **6j**) resulted in blue-shifted absorption and emission bands, as well as a significant reduction in Φ_F_, irrespective of the solvent (Table 1).

**Figure 5:**
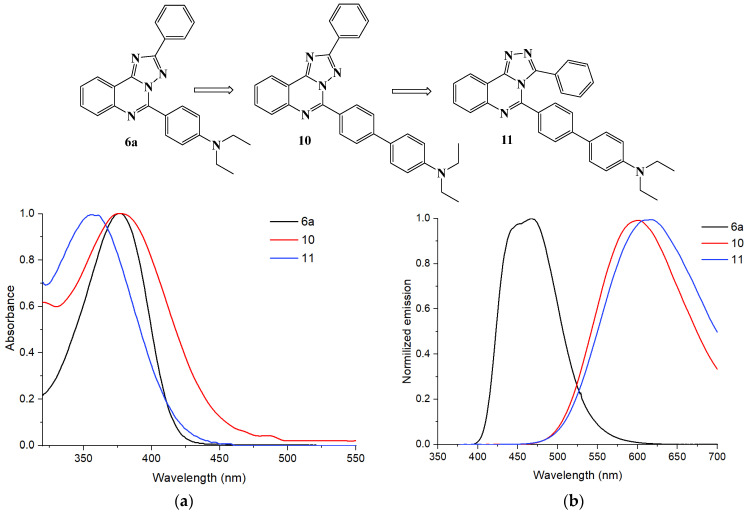
Combined normalized absorption (**a**) and emission (**b**) spectra of phenyl-substituted triazoloquinazolines **6a**, **10**, and **11** in MeCN (C = 10^−5^ M).

The introduction of an additional 1,4-phenylene spacer (compound **10** vs. **6a**) has little effect on the absorption spectrum (Figure 5a), but induces a significant bathochromic shift in the emission band—by 56 nm in toluene, while retaining a high fluorescence quantum yield (>95%), and by 142 nm in acetonitrile, where the quantum yield decreases to 35% (Table 1, Figure 5b). Compared to earlier reports on H- or Et-substituted [1,2,4]triazolo[1,5-*c*]quinazolines **F** [9], compound **10** exhibits a similar absorption band and emission characteristics (λ_em_ and Φ_F_), closely matching those of the H-substituted derivatives (Table S9, SI). The presence of the [4,3-*c*]-annelated fragment (compound **11** versus **10**) causes a hypsochromic shift in the absorption maximum, likely due to decreased molecular planarity and consequent reduction in conjugation length, stemming from steric hindrance from adjacent substituents. Similar twisting tendencies and associated spectral behaviours have been previously documented in related 3-ethyl-substituted [1,2,4]triazolo[4,3-*c*]quinazoline derivatives **E**, as referenced in Table S9, SI [9]. The fluorescence quantum yield of compound **11** does not exceed 34% in solution, and, unlike other compounds in the series, **11** displays diminished emission intensity when transferred from acetonitrile to toluene (Table 1).

Compounds **12a**,**b** displayed absorption with a long-wavelength maximum around 330 nm, along with red-shifted emission bands compared to their counterpart **6j**. Interestingly, the introduction of additional phenylene ring into the structure of 9,9′-spirobisfluorene-substituted derivatives (**12a**,**b** vs. **6j**) resulted in a pronounced enhancement of the emission intensity, with the Et-substituted derivative **12b** exhibiting a remarkable Φ_F_ of up to 95% in MeCN. Both compounds **12a**,**b** demonstrated higher Φ_F_ values in MeCN compared to toluene solution.

To summarize, in solution, stronger electron donors combined with longer effective conjugation (as exemplified by compound **10**) typically intensify ICT, narrow the HOMO–LUMO energy gap (refer to the Quantum chemical calculation section), and cause a red shift in absorption and emission profiles with increased solvatochromism compared to compound **5a**. However, excessive extension of conjugation can promote nonradiative decay, leading to reduced PLQY in polar media, as observed in compound **10** for both toluene and MeCN. Notably, direct attachment of an alkylaminophenyl substituent to the [1,2,4]triazolo[1,5-*c*]quinazoline core (compounds **6a**, **6d**, and **6g**) tends to discourage stabilization of the ICT state and favours emission from a locally excited (LE) state, despite the high donating power of the substituent. This phenomenon can be attributed to reduced effective conjugation across the D–π–A framework, diminishing the charge transfer character in the lowest energy excited state. Consistent with LE-dominated emission, the photoluminescence quantum yield remains high, indicating efficient radiative decay in the absence of a strongly stabilized ICT state. A similar solvent-dependent emission trend was observed for the Me_2_N/Ph_2_N-phenyl 5-nitrobenzo[*d*]thiazole pair [20]. When orbital alignment and π-coupling are optimized, this results in larger ICT dipoles, higher Stokes shifts, and bathochromic trends (observed in triphenyl-contained compounds **6b**, **6e**, **6h**). Conversely, poor alignment or twisted decoupling suppresses ICT and causes blue shifts in the spectra (as seen in carbazolyl-substituted derivatives **6c**, **6f**, and **6i**, as well as 9,9′-spirobisfluorene-substituted derivatives **6j** and **12a**,**b**).

In the solid state (powder form), compounds **6a**–**j**, **10**, **11,** and **12a**,**b** emit in the violet–green region (Figure 4b) with emission maxima ranging from 403 to 513 nm (Figure S29, Table 1). Solid-state emission of these compounds is either unchanged or red-shifted relative to their emission in toluene solution. There is no apparent correlation between the nature of aryl substituents in series **6a**–**i** and the position of the maximum emission band. The additional phenylene ring does not affect the emission maximum when comparing compounds **6a** (λ_max_ = 511 nm) and **10** (λ_max_ = 513 nm), though it leads to a red-shifted emission in the 9,9′-spirobisfluorene-substituted series (**12a**,**b** vs. **6j**). Absolute quantum yields in the solid state were determined to reach 45% (Table 1).

### 2.3. Solvatochromic Effect

The solvatochromic properties of compounds **6a**–**c**, **10**, and **11** were evaluated in a series of solvents of varying polarities (Figure S30), including cyclohexane, toluene, 1,4-dioxane, EtOAc, THF, CH_2_Cl_2_, DMSO, and MeCN. It was found that the emission colour of compound **6a** remains almost unaffected by the nature of the solvent, suggesting negligible changes in dipole moment upon photoexitation. Compounds **6b** and **6c**, however, appeared to be sensitive to changes in the surrounding medium, displaying solvatochromism with emission colours ranging from deep blue to yellow. The most pronounced solvent-dependent behaviour was observed for compounds **10** and **11**, where the solution colour changed from blue to orange with increasing solvent polarity. Importantly, in addition to the visible changes in solution colour, compounds **6b** and **10** exhibited intense fluorescence in all solvents, indicating their potential as solvatochromic fluorescent sensors.

Furthermore, absorption and emission spectrum were recorded for compounds **6a**–**c** and **10** in the same series of solvents. The maxima of the absorption bands were found to be weakly dependent on solvent polarity (Tables S10–S12), indicative of a low molecular dipole moment in the ground state.

**Figure 6:**
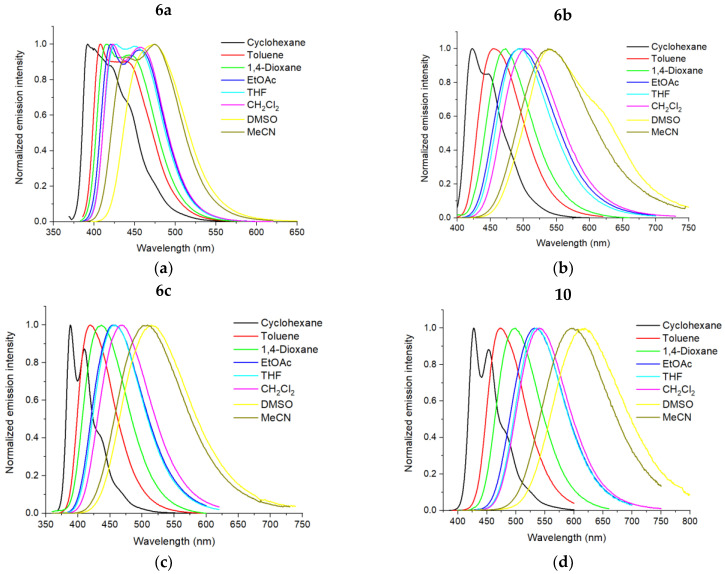
Combined normalized emission spectra of compounds **6a** (**a**), **6b** (**b**), **6c** (**c**), and **10** (**d**) in different solvents.

In line with visual observations, compound **6a** exhibits emission in the 380–500 nm region, showing a relatively modest red shift (Figure 6a and Figure S31). In contrast, the emission maxima of triazoloquinazolines **6b** and **6c** are red-shifted to 600 nm in polar solvents (Figure 6b,c). Finally, the emission profile of compound **10** spans a broad spectral region extending from 400 to 750 nm (Figure 6d and Figure S31).

In nonpolar cyclohexane, the fluorescence spectra of compounds **6a**–**c** and **10** exhibit well-resolved, structured emission bands (Figure 6), indicative of a rigid, less polar excited state with limited solvent–solute interactions. In more polar solvents (except for DMSO), compound **6a** displays a dual-emission profile with two distinct peaks (Figure 6a). This effect is likely attributable to the interplay between locally excited (LE) and intramolecular charge transfer (ICT) states, with solvent polarity modulating their respective contributions. Similar dual-behaviour in emission spectra was observed for smaller D-A molecules with a directly attached electron-donor fragment to an electron-withdrawing core [21]. The emission of other compounds **6b**, **6c**, and **10** becomes featureless, accompanied by broadened full-width-at-half-maximum (FWHM), as shown in Figure 6b–d. This reflects enhanced vibronic relaxation and stronger stabilization of the excited state by solvent dipoles. This behaviour suggests that the electronic transitions involve significant charge redistribution, which is effectively stabilized in polar media. The incremental red shift in emission spectra is likely due to reinforced donor–acceptor interactions in compounds **6b**, **6c**, and **10** compared to **6a.** Correlations between molecular structures and solvatochromic behaviour of compounds **6a**–**c** and **10** upon increasing solvent polarity align with previous literature reports on analogous systems [21], where transformations from a locally excited (LE) state to a charge transfer (CT) state are evident in the emission spectra.

Further, the change in dipole moment (Δ*μ*) between the ground and exited ICT state was estimated for compounds **6b**, **6c**, and **10** from the Ravi Equation (1) [22], using the slope of the Stokes shift dependence on the empirical solvent polarity parameter ($E_{T}^{N}$) proposed by Reichardt [23], Tables S7–S9, Figure S32.

(1) $$v_{a}-v_{f}=11,307.6\times E_{T}^{N}{\left(\frac{∆\mu }{{∆\mu }_{B}}\right)}^{2}{\left(\frac{a_{B}}{a}\right)}^{3}+constant$$

In Equation (1) $v_{a}-v_{f}$ is the Stokes shift (in cm^−1^), $E_{T}^{N}$ is the empirical solvent polarity parameter (Reichardt’s normalized E_T_(30) scale) [23], ∆μ and ${∆\mu }_{B}$—the difference between the dipole moments of the ground and excited states for the target fluorophore and for the standard betaine dye (${inD,∆\mu }_{B}=9D$ [24]), a and $a_{B}$ the Onsager cavity radius of the fluorophore and of the standard betaine dye ($in\;Å,\;a_{B}=6.2Å$ [24]).

Other methods for estimation of Δ*μ* (Lippert-Mataga [25,26], Bakhshiev [27]) were also tested; however, they yielded low correlation coefficient (R^2^ < 0.9), indicating limited reliability for the present series of compounds.

The difference in dipole moment for all studied triazoloquinazoline derivatives was calculated to be approximately 7.5–9 D, Table 2, confirming that the excited state of all fluorophores is more polar than the ground state. This increase in polarity corresponds to the intramolecular charge transfer (ICT) character of the exited state formed upon photoexitation.

The dipole moments of the ground (S_0_) and excited (S_1_) states for compounds **6b**, **6c**, and **10** in the gas phase were calculated by the DFT method at the B3LYP/6-31G*//PM6 level, and the results are presented in Table 2. The observed trends are generally consistent with the experimental data: the higher value of μ* corresponds to compound **10**, demonstrating more pronounced sensitivity of emission to solvent polarity compared to other samples. The difference in dipole moments ∆μ^DFT^ is also consistent with those obtained by the solvatochromic method, Table 2. However, the results are valid only within one series calculated by the adopted method, since the dipole moment values depend on the calculation approach and the geometry of the model system. In addition, the dipole moments obtained from the Ravi equation are limited by a set of assumptions as well as by the assumed Onsager radius [9].

### 2.4. Acid-Induced Spectral Changes in Fluorophores

**Figure 7:**
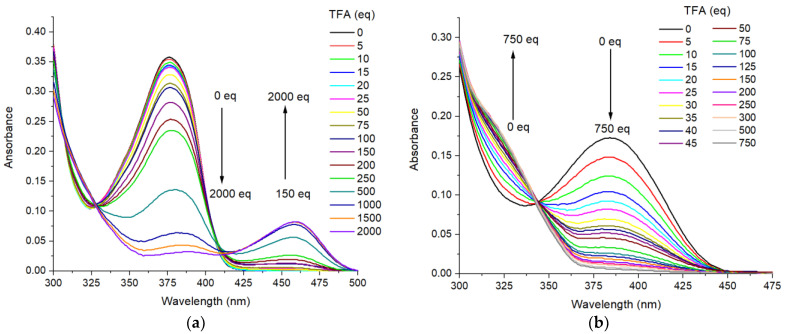

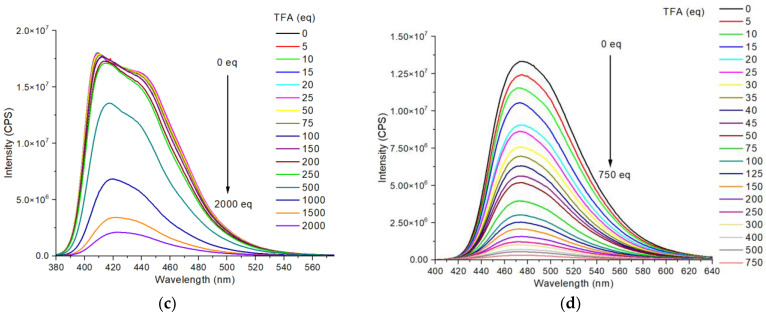
Absorption spectra changes in **6a** (**a**) and **10** (**b**) in toluene (C = 2 × 10^−6^ M) upon gradual addition of CF_3_COOH (TFA). Emission spectra changes in **6a** (**c**) and **10** (**d**) in toluene (C = 2 × 10^−6^ M) upon gradual addition of CF_3_COOH (TFA).

Since the photophysical properties of heteroaromatic fluorophores containing nitrogen atoms can be affected by protonation [7,29,30], the spectral response of the selected compounds **6a** and **10** to acid was examined to assess their potential for acidochromic applications. Initially, the response of the compounds **6a** and **10** solved in toluene to excess of trifluoroacetic acid was evaluated visually under UV light, which revealed full quenching of luminescence, Figure S33. Acid was (C = 0.01 M) then added stepwise to toluene solutions (C = 10^−5^ M) of **6a** and its biphenylene counterpart **10**, and the spectral changes were recorded to monitor the protonation process. Upon TFA titration, **6a** showed attenuation of the initial absorption band and appearance of a bathochromically shifted (by 83 nm) band emerging after 100 equivalents; the intensity of new band reached saturation after the addition of 1000 equivalents (Figure 7a). Compound **10** exhibited disappearance of the initial band, accompanied by a hypsochromic shift (by 58 nm); the original band vanished by 500 equivalents, with the new band remaining effectively constant thereafter (Figure 7b). The distinct responses suggest different protonation sites. For **6a**, protonation at the electron-deficient triazoloquinazoline core is plausible, increasing intramolecular charge transfer (ICT) and shifting the maximum from 376 to 459 nm (Figure S34). In contrast, for **10**, the shift from 384 to 326 nm (Figure S34) is consistent with protonation of the donating aminoaryl unit and excited-state destabilization. In both systems, steady-state fluorescence measurements revealed progressive quenching of emission intensity upon acid addition, with near-complete suppression observed at high concentrations of acid 2000 eq and 750 eq, respectively, Figure 7c,d. This quenching effect is likely associated with increased nonradiative decay pathways and disruption of π-conjugation following protonation. In both cases, the protonation process was reversible, and the colour of the toluene solution returned to its initial state upon addition of TEA to the acidic solutions (Figure S33). Unfortunately, only one reversible cycle was observed for each compound (Figures S36 and S37).

To support the proposed protonation sites, NMR experiments were performed. Upon addition of TFA-d_1_, characteristic downfield shifts were observed for the hydrogen atoms of NEt_2_ group, as well as atoms in phenylene ring adjacent to NEt_2_ group, Figure S38b. Contrary, signals of compound **6a** remain almost unchanged, Figure S38a.

These findings underscore the pivotal role of molecular architecture in dictating the acid-responsive optical behaviour of the studied donor–acceptor fluorophores. The distinct sites of protonation observed for compounds **6a** and **10** can be rationalized by considering the extent of π-conjugation between the electron-donating and electron-accepting units. In compound **6a**, the diethylaminophenyl group is directly attached to the electron-deficient triazoloquinazoline core. This close π-conjugation facilitates electron withdrawal from the donor unit by the electron-deficient triazoloquinazoline core, thereby reducing the electron density on the amino nitrogen and lowering its basicity. Consequently, protonation predominantly occurs at the heterocyclic core, promoting intramolecular charge transfer (ICT) and resulting in a pronounced bathochromic shift in the absorption spectrum. In contrast, compound **10** incorporates an additional phenylene spacer between the donor and acceptor units, effectively disrupting direct conjugation. This spatial separation weakens the electronic interaction between the diethylaminophenyl donor and the heterocyclic acceptor. As a consequence, the electron-rich aminoaryl group retains its basicity and becomes the preferred site for protonation. Protonation at this site diminishes the donor strength and destabilizes the excited state, leading to a hypsochromic shift. Thus, the inclusion of the phenylene spacer in **10** not only modifies the photophysical response but also redirects the protonation preference from the acceptor to the donor moiety due to reduced conjugation and charge delocalization.

### 2.5. Quantum Chemical Calculations

To gain insights into the electronic features of investigated fluorophores **6**, **10**, **11**, and **12**, the distributions of the frontier molecular orbitals, i.e., the highest occupied molecular orbital (HOMO) and the lowest unoccupied molecular orbital (LUMO), were simulated using density functional theory (DFT) at the B3LYP/6-31G*//PM6 level with Gaussian-09, as shown in Figure S12. The HOMO electrons in compounds **6a**–**d**, **6h**, **6j**, **10**, **11**, and **12a**,**b** are predominantly localized on the electron-donating aminoaryl or 9,9′-spirobifluorene units, including the phenylene spacer and, in the case of series **6**, the quinazoline core (this contribution is particularly pronounced in compound **6a**). In contrast, the LUMO electron density is largely shifted toward the electron-deficient phenyl(aryl)-substituted [1,2,4]triazoloquinazoline core, indicating an intramolecular charge transfer upon excitation. Analysis of the molecular orbital (MO) overlap shows that in the series of compounds **6**, the donor–acceptor separation is relatively weaker compared to other compounds, in which ICT character becomes more pronounced with the extension of the π-conjugated system. As expected, **10**, **11**, and **12a**,**b** exhibited more separated HOMO and LUMO electron distributions.

**Figure 8:**
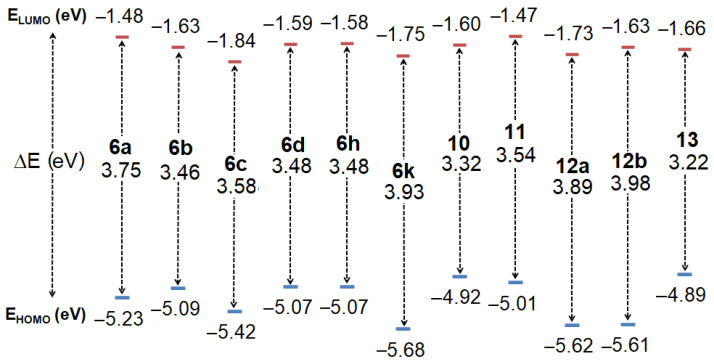
HOMO–LUMO energy levels and Eg for compounds **6a**–**d**, **6h**, **6j**, **10**, **11**, and **12a**,**b** in gas phase (HOMO energy levels are shown in blue, LUMO energy levels are shown in red).

Both the HOMO and LUMO energy values (Figure 8) showed dependence on the electron-donating characteristics of the aminoaryl substituent of the triazoloquinazolines **6a**–**c**. [1,2,4]Triazolo[1,5-*c*]quinazolines **6b**, **6d**, and **6h** with diphenylaminophenyl group demonstrated the lowest energy gap Eg = 3.46–3.48 eV compared to the other compounds of the series **6**. Incorporation of additional phenylene ring influence on both HOMO and LUMO levels and result in reduction in Eg value by 0.43 eV. The trends in the theoretical HOMO and LUMO levels are closely coincident with the UV–Vis absorption data, Table 1. The HOMO levels of 9,9′-spirobisfluoren-substituted triazoloquinazolines **6j**, **12a**, and **12b** turned out to be significantly lower than those of their counterparts depicting the weaker electron donating ability of residue at position 5.

Further, the geometries of **6a**–**d**, **6h**, **6j**, **10**, **11**, and **12a**,**b** were additionally optimized in toluene and MeCN at both the ground and excited states (Figure S13). Structural changes, including variations in dihedral angles (α), and bond lengths (L) between the donor and acceptor group upon excitation, were analyzed (Table S14). For clarity, dihedral angles were reported in the 0–90° range as absolute values to illustrate deviations from planarity.

The molecular geometries of compounds **6a**–**c**, which differ only in the nature of the electron-donating fragment, were compared. The rotation angle of the phenyl substituent (*α*_1_) slightly decreases by 3–15° in the excited state compared to the ground state for compounds **6b**,**c** in both solvents and for **6a** in acetonitrile. At the same time, the bond length *L*_1_ remains unchanged. In contrast, the angle *α*_2_ increases by 0.25–3.21°, which results in a distortion of the triazoloquinazoline core in exited state. The aminoaryl donor fragment is considerably twisted relative to the rest of the molecule in the ground state (angle *α*_3_ *≈* 34.5–55.0°). This torsion angle considerably reduces in S_1_ state in all cases, except for compound **6a** in acetonitrile, which can be attributed to the formation of a twisted intramolecular charge transfer (TICT) state stabilized by the polar solvent [31,32]. In toluene, the angle *α*_3_ does not exceed 4°, and the bond length *L*_2_ shortens by 0.043–0.066 Å, which makes molecular geometry of **6a**–**c** nearly planar. Notably, the diethylaminophenyl fragment (compound **6a**) remains twisted upon excitation in acetonitrile, while the corresponding bond length does not change, indicating the absence of effective conjugation and intramolecular charge transfer.

The introduction of a substituent into the phenyl ring has virtually no effect on the molecular geometry, as evidenced by comparisons between compounds **6e** and **6h** relative to **6b**. Geometry changes between the ground and excited states for compound **6j** resemble those observed for **6b** and **6c**. Compound **10** in toluene is characterized by two nearly coplanar conjugated systems: the 2-phenyltriazoloquinazoline core and the aminobiphenyl fragment, oriented relative to each other at an angle of approximately 40° (angle *α*_3_). However, in acetonitrile, the aminobiphenyl moiety assumes a non-planar configuration, suggesting that intramolecular charge transfer is accompanied by a twisted geometry (TICT effect). In compound **11**, the phenyl and biphenyl substituents are positioned nearly parallel to each other (angles *α*_1_ and *α*_3_) but are significantly rotated (*α*_1_ and *α*_3_ > 55°) relative to the triazoloquinazoline fragment in all states. This distortion originates from steric hindrance induced by the substituents and correlates with the observed hypsochromic shift in the absorption spectra. The aminobiphenyl fragment experiences similar geometric changes as in compound **10**, and the excited state likely corresponds to analogous conformations.

Interestingly, the 9,9′-spirobifluorene-contained 2-phenyl- and 2-ethyl-[1,2,4]triazolo[1,5-c]quinazolines **12a** and **12b** adopt a more planar molecular configuration of (fluorene)phenyl-[1,2,4]triazolo[1,5-*c*]quinazoline core in the S_1_ state compared to S_0_ in both solvents.

## 3. Materials and Methods

### 3.1. General Information

Unless otherwise indicated, all common reagents and solvents were obtained from commercial suppliers and used without further purification. Melting points were determined using Boetius combined heating stages. Thin-layer chromatography (TLC) was performed on ALUGRAM Xtra SIL G/UV254 plates (Macherey-Nagel, Duren, Germany). ^1^H NMR spectra were recorded at room temperature, unless otherwise specified, on a Bruker AVANCE II–400 spectrometer (Bruker BioSpin, Rheinstetten, Germany) operating at 400 MHz, using CDCl_3_ or DMSO-d_6_ as solvents. ^13^C NMR spectra were recorded at room temperature on a Bruker AVANCE II–400 spectrometer (Bruker BioSpin) or Bruker Avance NEO 600 spectrometer (Bruker BioSpin), operating at 100 and 150 MHz, respectively, using CDCl_3_ or DMSO-d_6_ as solvents. Chemical shifts (δ) are reported in parts per million (ppm) relative to the residual solvent signals as internal standards. Coupling constants (J) are given in Hertz (Hz). The following abbreviations were used to describe the NMR signals: s—singlet, br. s—broad singlet, d—doublet, t—triplet, q—quartet, and m—multiplet. Mass spectra were obtained using a Shimadzu GCMS-QP2010 Ultra instrument with electron ionization (EI) (Shimadzu, Duisburg, Germany). Elemental analyses (C, H, N) were carried out on a Perkin–Elmer 2400 elemental analyzer (Perkin–Elmer, Waltham, MA, USA).

### 3.2. Photophysical Characterization

All solvents used for spectrofluorimetric measurements were HPLC grade and used as received: cyclohexane anhydrous, 99.5% (Sigma-Aldrich, St. Louis, MO, USA), toluene for UV, IR, HPLC, GPC, ACS (PanReac AppliChem, Barselona, Spain), 1,4-dioxane for HPLS and spectroscopy, 99.8% (Alpha Chemika, Mumbai, India), ethyl acetate HPLC grade (Scharlau, Barselona, Spain), THF for UV, IR, HPLC, GPC, ACS (PanReac AppliChem), dichloromethane for HPLS and spectroscopy, 99.8% (Alpha Chemika), DMSO HPLC grade (Scharlau), and acetonitrile HPLC grade, ≥99.9% (Fisher Chemical, Thermo Fisher Scientific, Waltham, MA, USA).

UV/Vis absorption spectra were recorded on the Shimadzu UV-1800 (Shimadzu, Duisburg, Germany) using quartz cells with 1 cm path length at room temperature. Photoluminescent spectra were measured on the Horiba FluoroMax-4 (HORIBA Ltd., Kyoto, Japan) or FLS1000-SS time-resolved fluorescence spectrometer (Edinburgh Instruments, Livingston, UK) using quartz cells with 1 cm path length at room temperature. Absolute quantum yields of luminescence of target compounds in solution and solid state were measured using the Integrating Sphere Quanta-φ of the Horiba-Fluoromax-4.

### 3.3. Crystallography

The single crystal of compound **6g** (colourless prism of 0.41 × 0.19 × 0.08 mm), **6j** (yellow plank of 0.45 × 0.16 × 0.07 mm), **10** (yellow block of 0.41 × 0.19 × 0.06 mm), and compound **12a** (colourless prism of 0.46 × 0.35 × 0.15 mm) were used for X-ray analysis. Structural studies of the compounds were performed using equipment available in the Collaborative Access Centre “Spectroscopy and Analysis of the Organic compound” at the Postovsky Institute of the Organic Synthesis, Ural Branch, Russian Academy of Sciences. The X-ray diffraction analysis was performed at room temperature on the Xcalibur 3 diffractometer (Oxford Diffraction). Using Olex2 [33], the structure was solved with the ShelXT structure solution programme using Intrinsic Phasing and refined with the ShelXL [34], refinement package using full-matrix Least Squares minimization. All non-hydrogen atoms were refined in an anisotropic approximation; the H-atoms were placed in the calculated positions and refined isotropically in the “rider” model.

The results of X-ray diffraction analysis for compounds **6f**, **6g**, **10**, and **12a** were deposited with the Cambridge Crystallographic Data Centre (CCDC 2493884, CCDC 2482936, CCDC 2466809 and CCDC 2466371, respectively). These data are free and can be available at www.ccdc.cam.ac.uk (accessed on 12 November 2025).

Crystal data for **6f** C_34_H_23_N_5_ × CHCl_3_, M = 620.94, monoclinic, a = 7.0774(3) Å, b = 31.3482(12) Å, c = 13.5908(6) Å, α = 90°, β = 100.008(5)°, γ = 90°, V = 2969.4(2) Å^3^, space group *P2*_1_*/n*, Z = 4, μ(Mo Kα) = 0.343 mm^−1^. On the angles 2.273 < Θ < 30.851°, 22,547 reflections measured, 8194 unique (R_int_ = 0.0431), which were used in all calculations. Goodness to fit at F^2^ 1.011; the final R_1_ = 0.1627, wR_2_ = 0.2647 (all data) and R_1_ = 0.0906, wR_2_ = 0.2269 (I > 2σ(I)). Largest diff. peak and hole 0.31 and −0.27 ēÅ^−3^.

Crystal data for **6g** C_29_H_31_N_5_, M = 449.59, triclinic, a = 6.1850(4) Å, b = 11.2776(8) Å, c = 18.3493(14) Å, α = 79.834(6)°, β = 86.369(6)°, γ = 82.445(6)°, V = 1247.81(18) Å^3^, space group *P -1*, Z = 2, μ(Mo Kα) = 0.072 mm^−1^. On the angles 2.3280 < Θ < 25.3610°, 8878 reflections measured, 6476 unique (R_int_ = 0.0627), which were used in all calculations. Goodness to fit at F^2^ 1.006; the final R_1_ = 0.1704, wR_2_ = 0.2581 (all data) and R_1_ = 0.0787, wR_2_ = 0.1910 (I > 2σ(I)). Largest diff. peak and hole 0.44 and −0.25 ēÅ^−3^.

Crystal data for **10** C_31_H_27_N_5_, M = 469.57, monoclinic, a = 11.7244(4) Å, b = 17.3697(8) Å, c = 12.1246(5) Å, α = 90°, β = 92.182(4)°, γ = 90°, V = 2467.38(17) Å^3^, space group *P2*_1_*/n*, Z = 4, μ(Mo Kα) = 0.076 mm^−1^. On the angles 2.6290 < Θ < 30.5640°, 11,637 reflections measured, 3574 unique (R_int_ = 0.0531), which were used in all calculations. Goodness to fit at F^2^ 1.002; the final R_1_ = 0.1310, wR_2_ = 0.1813 (all data) and R_1_ = 0.0628, wR_2_ = 0.1429 (I > 2σ(I)). Largest diff. peak and hole 0.22 and −0.19 ēÅ^−3^.

Crystal data for **12a** C_46_H_28_N_4_ × 2(CHCl_3_), M = 875.46, triclinic, a = 11.9241(4) Å, b = 13.3599(6) Å, c = 13.5961(6) Å, α = 77.308(4)°, β = 79.548(3)°, γ = 81.823(3)°, V = 2066.08(15) Å^3^, space group *P -1*, Z = 2, μ(Mo Kα) = 0.457 mm^−1^. On the angles 2.4850 < Θ < 30.6010°, 16,932 reflections measured, 6038 unique (R_int_ = 0.0466), which were used in all calculations. Goodness to fit at F^2^ 1.089; the final R_1_ = 0.1361, wR_2_ = 0.2039 (all data) and R_1_ = 0.0741, wR_2_ = 0.2491 (I > 2σ(I)). Largest diff. peak and hole 0.46 and −0.46 ēÅ^−3^.

### 3.4. Computational Methods

The quantum chemical calculations were carried out at the B3LYP/6-31G*//PM6 level of theory with the help of the Gaussian-09 [35] programme package (Wallingford, CT, USA). No symmetry restrictions were applied during the geometry optimization procedure. The solvent effects were taken into account using the SMD (Solvation Model based on Density) continuum solvation model suggested by Truhlar and co-workers [36] for acetonitrile and toluene.

### 3.5. Synthetic Procedures

#### 3.5.1. Synthesis of the Intermediate Products

Ethyl *N*-(2-cyanophenyl)carbamate (**2**) was prepared similar to describe procedure [11]. To 2-aminobenzonitrile (2.7 g, 22.8 mmol) and potassium carbonate (9.5 g, 68.6 mmol) in tetrahydrofuran (130 mL), ethyl chloroformate (4.4 mL, 46.0 mmol) was added. The mixture was refluxed for 16 h. After cooling, the precipitate was filtered off and washed with tetrahydrofuran. The filtrate was evaporated under reduced pressure to give the title compound. Product was used without further purification. Colourless solid, yield: 99% (4.3 g); ^1^H NMR (DMSO-d_6_, 400 MHz): δ 1.24 (t, ^3^*J* = 6.8, 3H, CH_3_), 4.14 (q, ^3^*J* = 6.8, 2H, CH_2_), 7.30–7.34 (m, 1H), 7.50–7.52 (m, 1H), 7.64–7.68 (m, 1H), 7.77–7.79 (m, 1H), 9.7 (s, 1H, NH); ^13^C {^1^H} NMR (CDCl_3_, 100 MHz): δ 14.5 (CH_3_), 62.1 (CH_2_), 101.1, 116.4, 119.5, 123.2, 132.4, 134.3, 141.1, 153.0.
Compounds **4a**–**c** were obtained following the described procedure [11]. Ethyl *N*-(2-cyanophenyl)carbamate **2** (1.5 g, 7.85 mmol) and corresponding hydrazide **3a**–**c** (9.36 mmol) were stirred in N,N-dimethylformamide (10 mL) at 120 °C for 12 h. After cooling, water was added to the mixture, precipitated product was filtered off and recrystallized from MeCN (for **4a**) or DMSO (for **4b** and **4c**).
2-Phenyl-[1,2,4]triazolo[1,5-*c*]quinazolin-5(6*H*)-one (**4a)**. Colourless solid, yield: 72% (1.5 g); mp 296–298 °C (mp lit. 311–313 °C [37]). ^1^H NMR (DMSO-d_6_, 400 MHz) δ 7.39–7.66 (m, 6H), 8.24–8.25 (m, 3H), 12.31 (s, 1H, NH); ^13^C {^1^H} NMR (DMSO-d_6_, 100 MHz): δ 110.4, 116.1, 123.6, 124.2, 126.8, 129.0, 129.8, 130.4, 132.8, 137.1, 143.9, 153.4, 162.9; EIMS (*m*/*z*, I_rel_%): 263.0888 [M + 1]^+^ (18), 262.0855 [M]^+^ (100); Calcd exact mass for C_15_H_10_N_4_O (262.0855).
2-(*p*-Tolyl)-[1,2,4]triazolo[1,5-*c*]quinazolin-5(6*H*)-one (**4b**). Colourless solid, yield: 83% (1.80 g); mp 305–307 °C. ^1^H NMR (DMSO-d_6_, 400 MHz): δ 2.43 (3H, s, CH_3_), 7.32–7.40 (3H, m, H-3′, H-5′, H-8 or H-9), 7.45–7.47 (1H, m, H-7 or H-10), 7.64–7.64 (1H, m, H-8 or H-9), 8.12–8.14 (2H, d, *^3^J* = 7.5, H-2′, H-6′), 8.22–8.24, (1H, m, H-7 or H-10), 12.28 (1H, s, NH); ^13^C {^1^H} NMR (DMSO-d_6_, 150 MHz): δ 21.0 (CH_3_), 110.4, 116.1, 123.5, 124.2, 126.8, 127.1, 129.5, 132.7, 137.1, 140.2, 143.9, 153.3, 162.9; EIMS (*m*/*z*, I_rel_%): 277.1045 [M + 1]^+^ (20), 276.1011 [M]^+^ (100); Calcd exact mass for C_16_H_12_N_4_O (276.1011).
2-4-(*Tert*-butyl)phenyl-[1,2,4]triazolo[1,5-*c*]quinazolin-5(6*H*)-one (**4c**). Colourless solid, yield: 93% (2.33 g); mp 243–245 °C. ^1^H NMR (CDCl_3_, 400 MHz): δ 1.37 (9H, s, *t*-Bu), 7.36–7.40 (1H, m, H-8 or H-9), 7.51–7.54 (3H, m, H-3’, H-5’, H-7 or H-10), 7.59–7.61 (1H, m, H-8 or H-9), 8.29–8.31 (2H, d, *^3^J* = 7.8, H-2’, H-6’), 8.35–8.37, (1H, m, H-7 or H-10), 11.4 (1H, s, NH); ^13^C {^1^H} NMR (DMSO-d_6_, 150 MHz): δ 31.0 (C(CH_3_)_3_), 34.6 (C(CH_3_)_3_), 110.3, 116.1, 123.5, 124.2, 125.8, 126.7, 127.1, 132.7, 137.0, 143.9, 153.1, 153.3, 162.9; EIMS (*m*/*z*, I_rel_%): 318.1481 (29), 304.1324 [M-CH_3_ + 1]^+^ (23), 303.1241 [M-CH_3_]^+^ (100); Calcd exact mass for C_19_H_18_N_4_O (318.1481).
Compounds **5a**–**c** were obtained following the described procedure [11]. To the dried [1,2,4]triazolo[1,5-*c*]quinazolin-5(6*H*)-one **3a**–**c** (1.14 mmol) in phosphorus(V) oxychloride (7.2 mL, 77 mmol), *Ν,Ν*-diisopropylethylamine (0.4 mL, 2.27 mmol) was added carefully and the mixture was stirred for 20 h at 110 °C. Condenser was equipped with a calcium chloride drying tube. The mixture was concentrated. Resulting product was purified with column chromatography on SiO_2_ using mixture of hexane and EtOAc as eluent.
5-Chloro-2-phenyl-[1,2,4]triazolo[1,5-*c*]quinazoline (**5a**). Colourless solid, yield: 94% (300 mg); mp 170–172 °C. ^1^H NMR (CDCl_3_, 400 MHz) δ 7.53–7.55 (3H, m, H-3′, H-4′, H-5′), 7.75–7.79 (1H, m, H-8 or H-9), 7.85–7.89 (1H, m, H-8 or H-9), 8.01–8.02 (1H, m, H-10), 8.39–8.42 (2H, m, H-2’, H-6’), 8.61 (1H, d, *^3^J* = 7.9, H-7); ^13^C {^1^H} NMR (CDCl_3_, 100 MHz): δ 117.4, 124.3, 128.0, 128.2, 129.0, 129.1, 129.8, 131.0, 132.8, 136.0, 143.0, 153.0, 164.8. EIMS (*m*/*z*, I_rel_%): 282.0486 [M + 2]^+^ (33), 281.0549 [M + 1]^+^ (21), 280.0516 [M]^+^ (100), 245.0822 (18), 163.0058 (18), 102.0464 (15), 89.0386 (15). Calcd exact mass for C_15_H_9_ClN_4_ (280.0516).
5-Chloro-2-(*p*-tolyl)-[1,2,4]triazolo[1,5-*c*]quinazoline (**5b**). Beige solid, yield: 82% (277 mg); mp 166–168 °C. ^1^H NMR (DMSO-d_6_, 400 MHz): δ 2.45 (s, 3H, CH_3_), 7.38 (2H, d, *^3^J* = 8.1, H-3’, H-5’), 7.84–7.88 (1H, m, H-8 or H-9), 7.94–7.98 (1H, m, H-8 or H-9), 8.01–8.03 (1H, m, H-10), 8.22 (2H, d, *^3^J* = 8.1, H-2’, H-6’), 8.54 (1H, d, *^3^J* = 7.8, H-7); ^13^C {^1^H} NMR (CDCl_3_, 100 MHz): δ 21.7 (CH_3_), 117.3, 124.2, 126.9, 127.9, 128.1, 129.0, 129.7, 132.7, 136.0, 141.3, 142.9, 152.9, 164.9; EIMS (*m*/*z*, I_rel_%): 296.0643 [M + 2]^+^ (35), 295.0706 [M + 1]^+^ (25), 294.0672 [M]^+^ (100), 293.7299 (17), 163.0058 (11), 131.0730 (13), 116.0495 (12), 102.0464 (19), 90.0470 (13); Calcd exact mass for C_16_H_11_ClN_4_ (294.0672).
5-Chloro-2-(4-(*Tert*-butyl)phenyl)-[1,2,4]triazolo[1,5-*c*]quinazoline (**5c**). Beige solid, yield: 80% (297 mg); mp 126–128 °C. ^1^H NMR (DMSO-d_6_, 400 MHz): δ 1.39 (s, 9H, 3 CH_3_), 7.56 (2H, d, *^3^J* = 8.5, H-3′, H-5′), 7.82–7.86 (1H, m, H-8 or H-9), 7.93–7.95 (1H, m, H-8 or H-9), 7.99–8.01 (1H, m, H-10), 8.22 (2H, d, *^3^J* = 8.5, H-2′, H-6′), 8.54 (1H, d, *^3^J* = 7.9, H-7); ^13^C {^1^H} NMR (DMSO-d_6_, 100 MHz): δ 31.0 (C(CH_3_)_3_), 34.7 (C(CH_3_)_3_),, 116.9, 123.7, 125.9, 126.6, 127.0, 127.6, 129.1, 132.8, 135.5, 142.4, 152.5, 153.7, 163.0, 163.0; EIMS (*m*/*z*, I_rel_%): 338.1112 [M+2]^+^ (10), 336.1142 [M]^+^ (28), 323.0872 (35), 322.0936 (23), 321.0902 [M-CH_3_]^+^ (100), 163.0058 (11), 146.1096 (13), 102.0464 (11); Calcd exact mass for C_19_H_17_ClN_4_ (336.1142).
4-Hydrazino-2-(4-bromophenyl)quinazoline (**7**) and 5-(4-Bromophenyl)-2-ethyl- [1,2,4]triazolo[1,5-*c*]quinazoline (**8b**) were prepared as described previously [8,9].
Synthesis of 5-(4-Bromophenyl)-2-phenyl-[1,2,4]triazolo[1,5-*c*]quinazoline (**8a**).
Method 1. In a round-bottom flask equipped with a magnetic stirred bar, 4-hydrazino-2-(4-bromophenyl)quinazoline **7** (0.27 g, 0.86 mmol) in glacial acetic acid (7 mL) triethyl orthobenzoate (0.86 mL, 4.3 mmol) were added. The mixture was refluxed for 16 h. After cooling down, the water was added until the formation of precipitate. The product was filtered off and washed with water and recrystallized from DMSO.
Method 2. In a round-bottom flask equipped with a magnetic stirred bar, 5-(4-bromophenyl)-3-phenyl-[1,2,4]triazolo[4,3-*c*]quinazoline **9** (0.18 g, 0.45 mmol) was refluxed in glacial acetic acid (3.6 mL) for 20 h. After cooling down, the water was added until the formation of precipitate. The solid was filtered off and washed with water.
Colourless powder, yield 79%, 0.27 g (method 1), yield 48%, 0,073 g (method 2); mp 190–192 °C; ^1^H NMR (DMSO-d_6_, 400 MHz): δ 7.58–7.60 (3H, m, H-3′, H-4′, H-5′), 7.85–7.91 (3H, m, H-3″, H-5″, H-8 or H-9), 7.96–7.99 (1H, m, H-8 or H-9), 8.13–8.15 (1H, m, H-10), 8.30–8.33 (2H, m, H-2′, H-6′), 8.53–8.55 (3H, m, H-2″, H-6″, H-7); ^13^C {^1^H} NMR (CDCl_3_, 100 MHz, 45 °C): δ 116.9, 123.3, 125.4, 127.1, 128.4, 128.8, 128.9, 129.7, 130.6, 131.3, 132.2, 132.5, 142.2, 145.0, 152.4, 162.8; EIMS (*m*/*z*, I_rel_%): 402.0303 [M + 2]^+^ (100), 401.0357 [M + 1]^+^ (66), 400.0324 [M]^+^ (99); Calcd exact mass for C_21_H_13_BrN_4_ (400.0324).
Synthesis of 5-(4-bromophenyl)-3-phenyl-[1,2,4]triazolo[4,3-*c*]quinazoline (**9**). Starting 2-(4-bromophenyl)-4-hydrazinoquinazoline was preliminarily dried in oven at 100 °C for 4 h.
Method 1. In a round-bottom flask equipped with a magnetic stirred bar, 2-(4-bromophenyl)-4-hydrazinoquinazoline **7** (0.32 g, 1.00 mmol) in absolute ethanol (17 mL) and triethyl orthobenzoate (1 mL, 4.80 mmol) were added. The mixture was refluxed for 4 h. Condenser was equipped with a calcium chloride drying tube. After cooling down and partial evaporation, the solid was filtered off, washed with EtOH (5 mL). The product was purified by column chromatography on SiO_2_ using EtOAc and hexane as eluent, gradually from (1:9) to pure EtOAc.
Method 2. In a round-bottom flask equipped with a magnetic stirred bar, dried 2-(4-bromophenyl)-4-hydrazinoquinazoline 7 (0.20 g, 0.62 mmol) and triethyl orthobenzoate (1 mL, 4.80 mmol) were added. The mixture was refluxed for 4 h. A condenser was equipped with a calcium chloride drying tube. After cooling down, the solid was filtered off, washed with EtOH (5 mL), and dried.
Colourless powder, yield 63% (method 1), yield 81% (method 2); mp 237–239 °C; ^1^H NMR (DMSO-d_6_, 400 MHz): δ 7.13–7.23 (6H, m, H-2″, H-3″, H-5″, H-6″, H-2′, H-6′), 7.29–7.31 (3H, m, H-3′, H-4′, H-5′), 7.78–7.82 (1H, m, H-7 or H-10), 7.84–7.88 (1H, m, H-7 or H-10), 7.96–7.98 (1H, m, H-10), 8.62 (1H, d, *^3^J* = 7.9, H-7); EIMS (*m*/*z*, I_rel_%): 402.0303 [M + 2]^+^ (94), 401.0357 [M + 1]^+^ (79), 400.0324 [M]^+^ (100); Calcd exact mass for C_21_H_13_BrN_4_ (400.0324).

#### 3.5.2. Synthesis of the Target Products

General cross-coupling procedure for the synthesis of compounds **6a**–**j**, **10**, **11,** and **12a**,**b**.
The corresponding boronic acid or boronic acid pinacol ester (0.57 mmol), PdCl_2_(PPh_3_)_2_ (40 mg, 57 μmol), PPh_3_ (30 mg, 114 μmol), saturated solution of K_2_CO_3_ (3.1 mL) and EtOH (3.1 mL) were added to the suspension of the corresponding chloro or bromo derivative (**5a**–**c** or **8a**,**b**, **9**) (0.53 mmol) in toluene (19 mL). The mixture was stirred at 85 °C for 14–30 h in argon atmosphere in round-bottom pressure flask equipped with magnetic stirred bar. The reaction mixture was cooled to room temperature, and EtOAc/H_2_O (10/10 mL) mixture was added. The organic layer was separated, additionally washed with water (10 mL), and evaporated at reduced pressure. The product was purified by column chromatography.
5-(4-Diethylaminophenyl)-2-phenyl-[1,2,4]triazolo[1,5-*c*]quinazoline (**6a**).
The general procedure was applied using 5-chloro-2-phenyl- [1,2,4]triazolo[1,5-*c*]quinazoline **5a** and 4-(diethylamino)phenylboronic acid as the starting materials. Reaction time is 30 h. Column chromatography: SiO_2_, EtOAc and hexane (1:9) was used as an eluent. Pale-yellow solid, yield: 54% (114 mg); mp = 135–137 °C; ^1^H NMR (CDCl_3_, 400 MHz): δ 1.27 (6H, t, ^3^*J* = 7.1, 2CH_3_), 3.50 (4H, q, ^3^*J* = 7.1, 2CH_2_), 6.85 (2H, d, ^3^*J* = 9.2, H-3″, H-5″), 7.47–7.56 (3H, m, H-3′, H-4′, H-5′), 7.60–7.64 (1H, m, H-8), 7.77–7.81 (1H, m, H-9), 8.04–8.05 (1H, m, H-10), 8.43–8.45 (2H, m, H-2′, H-6′), 8.59 (1H, d, ^3^*J* = 8.1, H-7), 8.74 (2H, d, ^3^*J* = 9.2, H-2″, H-6″); ^13^C {^1^H} NMR (CDCl_3_, 100 MHz): δ 12.8 (2 CH_3_), 44.7 (2 CH_2_), 110.8, 116.9, 118.0, 123.8, 127.0, 127.8, 128.3, 128.8, 130.3, 130.8, 131.9, 132.5, 143.6, 146.8, 150.2, 153.2, 163.7; EIMS (*m*/*z*, I_rel_%): 394.1987 [M + 1]^+^ (18), 393.1953 [M]^+^ (58), 379.1752 (29), 378.1719 [M-CH_3_]^+^ (100), 350.1406 (12), 189.0578 (17); Calcd exact mass for C_25_H_23_N_5_ (393.1953). Calcd: C, 76.31, H, 5.89, N, 17.80%; Found: C, 76.22, H, 6.04, N, 17.56%.
5-(4-Diphenylaminophenyl)-2-phenyl-[1,2,4]triazolo[1,5-*c*]quinazoline (**6b**). The general procedure was applied using 5-chloro-2-phenyl-[1,2,4]triazolo[1,5-*c*]quinazoline **5a** and 4-(diphenylamino)phenylboronyc acid as the starting materials. Reaction time is 14 h. Column chromatography: SiO_2_, hexane and CH_2_Cl_2_ (7:3) and then EtOAc and hexane (1:9). Pale-yellow solid, yield: 58% (150 mg); mp = 191–193 °C; ^1^H NMR (CDCl_3_, 400 MHz): δ 7.13–7.16 (2H, m, Phenyl), 7.21–7.24 (6H, m, Phenyl, H-3″, H-5″), 7.33–7.37 (4H, m, Phenyl), 7.51–7.53 (3H, m, H-3′, H-4′, H-5′), 7.66–7.70 (1H, m, H-8), 7.81–7.84 (1H, H-9), 8.08 (1H, d, ^3^*J* = 8.2, H-10), 8.38–8.44 (2H, m, H-2′, H-6′), 8.61–8.65 (3H, m, H-7, H-2″, H-6″); EIMS (*m*/*z*, I_rel_%): 490.1987 [M + 1]^+^ (39), 489.1953 [M]^+^ (100), 488.1870 (14), 77.0386 (11); Calcd exact mass for C_33_H_23_N_5_ (489.1953). Calcd: C, 80.96, H, 4.74, N, 14.30%. Found: C, 80.86, H, 4.87, N, 14.19%.
5-(4-(9*H*-carbazol-9-yl)phenyl)-2-phenyl-[1,2,4]triazolo[1,5-*c*]quinazoline (**6c**).
The general procedure was applied using 5-chloro-2-phenyl- [1,2,4]triazolo[1,5-*c*]quinazoline **5a** and 9*H*-Carbazole-9-(4-phenyl) boronic acid pinacol ester as the starting materials. Reaction time is 21 h. Column chromatography: SiO_2_, hexane and CH_2_Cl_2_ (7:3) and then EtOAc and hexane (1:9). Pale-yellow solid, yield: 44% (113 mg); mp = 235–237 °C; ^1^H NMR (CDCl_3_, 400 MHz): δ 7.33–7.36 (2H, m, carbazolyl), 7.46–7.49 (2H, m, carbazolyl), 7.53–7.57 (3H, m, H-3′, H-4, H-5′), 7.61–7.63 (2H, m, carbazolyl), 7.75–7.79 (1H, m, H-8), 7.87–7.90 (3H, m, H-9, H-3″, H-5″), 8.17–8.19 (3H, m, H-10, carbazolyl), 8.46–8.48 (2H, m, H-2′, H-6′), 8.69 (1H, d, ^3^*J* = 8.2, H-7), 8.98–99.00 (2H, m, H-2″, H-6″); ^13^C {^1^H} NMR (CDCl_3_, 100 MHz): δ 110.1, 117.6, 120.6, 120.6, 123.9, 124.1, 126.3, 126.6, 127.9, 128.6, 129.0, 130.4, 130.6, 130.8, 132.4, 140.6, 141.0, 143.1, 145.7, 153.2, 164.4; EIMS (*m*/*z*, I_rel_%): 488.1831 [M + 1]^+^ (37), 487.1797 [M]^+^ (100). Calcd exact mass for C_33_H_21_N_5_ (487.1797). Calcd: C, 81.29; H, 4.34; N, 14.37%. Found: C, 81.21; H, 4.42; N, 14.28%.
5-(4-Dimethylaminophenyl)-2-(*p*-tolyl)-[1,2,4]triazolo[1,5-*c*]quinazoline (**6d**). The general procedure was applied using 5-chloro-2-(*p*-tolyl)-[1,2,4]triazolo[1,5-*c*]quinazoline **5b** and 4-(dimetylamino)phenylboronyc acid as the starting materials. Reaction time is 21 h. Column chromatography: SiO_2_, hexane and CH_2_Cl_2_ (1:1) and then EtOAc and hexane (1:9). Colourless solid, yield: 56% (112 mg); mp = 176–178 °C; ^1^H NMR (400 MHz, CDCl_3_): δ 2.45 (3H, s, CH_3_), 3.12 (6H, s, N(CH_3_)_2_), 6.88 (2H, d, ^3^*J* = 9.2, H-3″, H-5″), 7.34 (2H, d, ^3^*J* = 8.0, H-3′, H-5′), 7.61–7.65 (1H, m, H-8), 7.77–7.81 (1H, m, H-9), 8.04–8.06 (1H, m, H-10), 8.32 (2H, d, ^3^*J* = 8.0, H-2′, H-6′), 8.58 (1H, d, ^3^*J* = 8.0, H-7), 8.74–8.77 (2H, m, H-2″, H-6″); ^13^C {^1^H} NMR (CDCl_3_, 100 MHz): δ 21.7 (CH_3_), 40.3 (N(CH_3_)_2_), 111.3, 117.0, 119.0, 123.8, 127.1, 127.7, 128.0, 128.3, 129.5, 131.8, 132.3, 140.5, 143.6, 146.8, 152.6, 153.1, 163.8; EIMS (*m*/*z*, I_rel_%): 380.1831 [M + 1]^+^ (28), ), 379.1797 [M]^+^ (100), 378.1714 (29), 190.0652 (10); Calcd exact mass for C_24_H_21_N_5_ (379.1797). Calcd: C, 75.97, H, 5.58, N, 18.45%. Found: C, 75.92, H, 5.63, N, 18.58%.
5-(4-Diphenylaminophenyl)-2-(*p*-tolyl)-[1,2,4]triazolo[1,5-*c*]quinazoline (**6e**). The general procedure was applied using 5-chloro-2-(*p*-tolyl)-[1,2,4]triazolo[1,5-*c*]quinazoline **5b** and 4-(diphenylamino)phenylboronyc acid as the starting materials. Reaction time is 21 h. Column chromatography: SiO_2_, hexane and CH_2_Cl_2_ (1:4) and then EtOAc and hexane (1:9). Pale-yellow, yield: 27% (73 mg); mp = 198–200 °C; ^1^H NMR (CDCl_3_, 400 MHz): δ 2.44 (3H, s, CH_3_), 7.12–7.16 (2H, m, Phenyl), 7.20–7.25 (6H, m, Phenyl, H-3″, H-5″), 7.31–7.36 (6H, m, Phenyl, H-3′, H-5′), 7.65–7.69 (1H, m, H-8), 7.79–7.84 (1H, m, H-9), 8.06–8.08 (1H, m, H-10), 8.29 (2H, d, ^3^*J* = 8.1, H-2′, H-6′), 8.60–8.65 (3H, m, H-7, H-2″, H-6″); ^13^C {^1^H} NMR (CDCl_3_, 100 MHz): δ 21.7 (CH_3_), 117.2, 120.8, 123.9, 124.2, 124.4, 125.9, 127.7, 127.8, 128.6, 129.6, 129.7, 131.9, 132.0, 140.7, 143.3, 146.2, 147.0, 151.0, 153.1, 164.1; EIMS (*m*/*z*, I_rel_%): 504.2144 [M + 1]^+^ (40), 503.2110 [M]^+^ (100), 502.2027 (13), 252.1375 (11), 77.0386 (11); Calcd exact mass for C_34_H_25_N_5_ (503.2110). Calcd: C, 81.09, H, 5.00, N, 13.91%. Found: C, 81.55, H, 5.13, N, 13.32%.
5-(4-(9*H*-carbazol-9-yl)phenyl)-2-(*p*-tolyl)-[1,2,4]triazolo[1,5-*c*]quinazoline (**6f**). The general procedure was applied using 5-chloro-2-(*p*-tolyl)-[1,2,4]triazolo[1,5-*c*]quinazoline **5b** and 9*H*-carbazole-9-(4-phenyl) boronic acid pinacol ester as the starting materials. Reaction time is 21 h. Column chromatography: SiO_2_, hexane and CH_2_Cl_2_ (7:3). Colourless solid, yield: 70% (187 mg); mp = 210–212 °C; ^1^H NMR (CDCl_3_, 400 MHz): δ 2.46 (3H, s, CH_3_), 7.33–7.38 (4H, m, carbazolyl, H-3′, H-5′), 7.46–7.50 (2H, m, carbazolyl), 7.61–7.63 (2H, m, carbazolyl), 7.75–7.79 (1H, m, H-8), 7.87–7.92 (3H, m, H-9, H-3″, H-5″), 8.16–8.19 (3H, m, H-10, carbazolyl), 8.35 (d, ^3^*J* = 8.1, 2H, H-2′, H-6′), 8.67–8.69 (1H, m, H-7), 8.97–9.01 (2H, m, H-2″, H-6″); ^13^C {^1^H} NMR (CDCl_3_, 100 MHz): δ 21.7 (CH_3_), 110.1, 117.6, 120.6, 120.6, 123.9, 124.1, 126.3, 126.6, 127.6, 127.8, 128.5, 128.9, 129.7, 130.7, 132.3, 132.4, 140.6, 140.9, 141.0, 143.1, 145.7, 153.2, 164.5; EIMS (*m*/*z*, I_rel_%): 502.1987 [M + 1]^+^ (38), 501.1953 [M]^+^ (100), 500.1870 (13), 251.1297 (19); Calcd exact mass for C_34_H_23_N_5_ (501.1953). Calcd: C, 81.42, H, 4.62, N, 13.96%. Found: C, 81.29, H, 4.70, N, 13.83%.
5-(4-Diethylaminophenyl)-2-(4-(*tert*-butyl)phenyl)-[1,2,4]triazolo[1,5-*c*]quinazoline (**6g**). The general procedure was applied using 5-chloro-2-(4-(*tert*-butyl)phenyl)-[1,2,4]triazolo[1,5-*c*]quinazoline **5c** and 4-(diethylamino)phenyl boronic acid as the starting materials. Reaction time is 14 h. Column chromatography: SiO_2_, eluent: gradually from hexane/CH_2_Cl_2_ (8:2) to CH_2_Cl_2_. Yellow solid, yield: 20% (46 mg); mp = 135–137 °C; ^1^H NMR (CDCl_3_, 400 MHz): δ 1.27 (6H, t, ^3^*J* = 7.1, 2CH_3_), 1.39 (9H, s, *t*-Bu), 3.50 (4H, q, ^3^*J* = 7.1, 2CH_2_), 6.84 (2H, d, ^3^*J* = 9.1, H-3″, H-5″), 7.55 (2H, d, ^3^*J* = 8.4, H-3′, H-5′), 7.60–7.63 (1H, m, H-8), 7.76–7.80 (1H, m, H-9), 8.03–8.05 (1H, m, H-10), 8.35 (2H, d, ^3^*J* = 8.4, H-2′, H-6′), 8.59 (1H, d, ^3^*J* = 8.4, H-7), 8.74 (2H, d, ^3^*J* = 9.1, H-2″, H-6″); ^13^C {^1^H} NMR (CDCl_3_, 100 MHz): δ 12.7 (2CH_3_), 31.3 (C(CH_3_)_3_), 34.9 (C(CH_3_)_3_), 44.6 (2 CH_2_), 110.7, 116.8, 117.9, 123.7, 125.6, 126.8, 127.4, 127.9, 128.1, 131.7, 132.4, 143.5, 146.7, 150.1, 153.0, 153.5, 163.6; EIMS (*m*/*z*, I_rel_%): 450.2613 [M + 1]^+^ (26), 449.2579 [M]^+^ (72), 435.2423 (36), 434.2340 [M-CH_3_]^+^ (100), 390.1714 (14), 210.1283 (27), 196.1126 (14), 182.1096 (11); Calcd exact mass for C_29_H_31_N_5_ (449.2579). Calcd: C, 77.47, H, 6.95, N, 15.58%; Found: C, 77.38, H, 6.85, N, 15.44%.
5-(4-Diphenylaminophenyl)-2-(4-(*tert*-butyl)phenyl)-[1,2,4]triazolo[1,5-*c*]quinazoline (**6h**). The general procedure was applied using 5-chloro-2-(4-(*tert*-butyl)phenyl)-[1,2,4]triazolo[1,5-*c*]quinazoline **5c** and 4-(diphenylamino)phenylboronyc acid as the starting materials. Reaction time is 14 h. Column chromatography: SiO_2_, hexane and CH_2_Cl_2_ (7:3) and then EtOAc and hexane (1:9). Pale-yellow solid, yield: 61% (177 mg); mp = 223–225 °C; ^1^H NMR (CDCl_3_, 400 MHz): δ 1.38 (9H, s, *t*-Bu), 7.12–7.16 (2H, m, Phenyl), 7.20–7.24 (6H, m, Phenyl, H-3″, H-5″), 7.33–7.37 (4H, m, Phenyl), 7.54 (2H, d, ^3^*J* = 8.3, H-3′, H-5′), 7.66–7.69 (1H, m, H-8), 7.80–7.84 (1H, m, H-9), 8.06–8.08 (1H, m, H-10), 8.32 (2H, d, ^3^*J* = 8.3, H-2′, H-6′), 8.61–8.65 (3H, m, H-7, H-2″, H-6″); ^13^C {^1^H} NMR (CDCl_3_, 100 MHz): δ 31.4 (C(CH_3_)_3_), 35.0 (C(CH_3_)_3_), 117.3, 120.8, 123.9, 124.3, 124.4, 125.8, 125.9, 127.6, 127.7, 127.8, 128.6, 129.7, 131.9, 132.0, 143.3, 146.2, 147.0, 151.0, 153.1, 153.8, 164.1; EIMS (*m*/*z*, I_rel_%): 546.2613 [M+1]^+^ (43), 545.2579 [M]^+^ (100), 530.2340 [M-CH_3_]^+^ (15), 265,1699 (22), 251.1674 (11); Calcd exact mass for C_37_H_31_N_5_ (545.2579). Calcd: C, 81.44, H, 5.73, N, 12.83%. Found: C, 81.50, H, 5.82, N, 12.78%.
5-(4-(9*H*-Carbazol-9-yl)phenyl)-2-(4-(*tert*-butyl)phenyl)-[1,2,4]triazolo[1,5-*c*]quinazoline (**6i**). The general procedure was applied using 5-chloro-2-(4-(*tert*-butyl)phenyl)-[1,2,4]triazolo[1,5-*c*]quinazoline **5c** and 9*H*-carbazole-9-(4-phenyl) boronic acid pinacol ester as the starting materials. Reaction time is 21 h. Column chromatography: SiO_2_, hexane and CH_2_Cl_2_ (7:3) and then EtOAc and hexane (1:9). Colourless solid, yield: 58% (167 mg); mp = 228–230 °C; ^1^H NMR (CDCl_3_, 400 MHz): δ 1.40 (9H, s, *t*-Bu), 7.33–7.37 (2H, m, carbazolyl), 7.46–7.50 (2H, m, carbazolyl), 7.58–7.63 (4H, m, carbazolyl, H-3′, H-5′), 7.75–7.79 (1H, m, H-8), 7.86–7.92 (3H, m, H-9, H-3″, H-5″), 8.16–8.19 (3H, m, H-10, carbazolyl), 8.38 (2H, d, ^3^*J* = 8.5, H-2′, H-6′), 8.68–8.71 (1H, m, H-7), 9.00 (2H, m, ^3^*J* = 8.7, H-2″, H-6″); ^13^C {^1^H} NMR (CDCl_3_, 100 MHz): δ 31.4 (C(CH_3_)_3_), 35.1 (C(CH_3_)_3_), 110.1, 117.6, 120.6, 120.6, 123.9, 124.1, 125.9, 126.3, 126.6, 127.6, 127.7, 128.5, 128.9, 132.3, 132.4, 140.6, 140.9, 143.1, 145.7, 153.2, 154.1, 164.5; EIMS (*m*/*z*, I_rel_%): 544.2457 [M + 1]^+^ (44), 543.2423 [M]^+^ (100), 529.2266 (15), 528.2183 [M-CH_3_]^+^ (36), 268.1939 (13), 264.1626 (27), 250.1591 (19); Calcd exact mass for C_37_H_29_N_5_ (543.2423). Calcd: C, 81.74, H, 5.38, N, 12.88%. Found: C, 81.88, H, 5.46, N, 12.65%.
5-(9,9′-Spirobi[fluoren]-2-yl)-2-phenyl-[1,2,4]triazolo[1,5-*c*]quinazoline (**6j**). The general procedure was applied using 5-chloro-2-phenyl-[1,2,4]triazolo[1,5-*c*]quinazoline **5a** and 9,9′-spirobifluorene-2-boronic acid as the starting materials. Reaction time is 14 h. Column chromatography: SiO_2_, hexane and EtOAc (8:2) to pure EtOAc. Colourless solid, yield: 52% (155 mg); mp = 269–271 °C; ^1^H NMR (CDCl_3_, 400 MHz): δ 6.86–6.88 (3H, m, fluoren), 7.17–7.24 (3H, m), 7.42–7.47 (6H, m, H-3′, H-4′, H-5′), 7.64–7.68 (1H, m, H-8), 7.78–7.80 (1H, m, H-9), 7.88–7.90 (2H, m), 7.97–7.8.15 (5H, m), 8.15 (1H, d, ^4^*J* = 1.1, bisfluorenyl), 8.54–8.56 (2H, m, H-2′, H-6′); ^13^C {^1^H} NMR (CDCl_3_, 100 MHz): δ 117.3, 120.2, 120.3, 121.0, 123.9, 124.4, 124.5, 126.9, 127.6, 128.0, 128.1, 128.2, 128.8, 128.8, 129.0, 130.2, 130.4, 130.5, 131.0, 132.1, 141.1, 142.1, 143.1, 145.1, 146.5, 148.3, 148.8, 149.8, 152.8, 163.7; EIMS (*m*/*z*, I_rel_%): 561.2035 [M + 1]^+^ (48), 560.2001 [M]^+^ (100); Calcd exact mass for C_40_H_24_N_4_ (560.2001).Calcd: C, 85.69, H, 4.31, N, 9.99%. Found: C, 85.52, H, 4.45, N, 9.84%.
5-(4′-Diethylamino-[1,1′]-biphenyl-4-yl)-2-phenyl-[1,2,4]triazolo[1,5-*c*]quinazoline (**10**). The general procedure was applied using 5-(4-bromophenyl)-2-phenyl-[1,2,4]triazolo[1,5-*c*]quinazoline **8a** and 4-(diethylamino)phenyl boronic acid as the starting materials. Reaction time is 16 h. Column chromatography: SiO_2_, hexane and EtOAc from (1:9) to (1:1). Bright-yellow solid, yield: 36% (91 mg); mp = 190–192 °C; ^1^H NMR (CDCl_3_, 400 MHz): δ 1.23 (6H, t, ^3^*J* = 7.1, 2CH_3_), 3.44 (4H, q, ^3^*J* = 7.1, 2CH_2_) 6.80 (2H, d, ^3^*J* = 8.3, Et_2_NC_6_H_4_), 7.49–7.56 (3H, m, H-3′, H-4′, H-5′), 7.64 (2H, d, ^3^*J* = 8.3, 2H, Et_2_NC_6_H_4_), 7.70–7.73 (1H, m, H-8), 7.81–7.87 (3H, m, H-9, H-3″, H-5″), 8.12–8.14 (1H, m, H-10), 8.44 (2H, d, ^3^*J* = 7.1, H-2′, H-6′), 8.65 (1H, d, ^3^*J* = 8.3, H-7), 8.75 (2H, d, ^3^*J* = 8.3, H-2″, H-6″); ^13^C {^1^H} NMR (CDCl_3_, 100 MHz): δ 12.8 (2CH_3_), 44.6 (2CH_2_), 112.0, 117.4, 123.9, 125.8, 126.7, 127.9, 128.1, 128.3, 128.8, 128.9, 129.0, 130.5, 130.6, 131.1, 132.1, 143.3, 144.5, 146.6, 147.9, 153.1, 164.1; EIMS (*m*/*z*, I_rel_%): 470.2300 [M + 1]^+^ (34), 469.2266 [M]^+^ (88), 455.2110 (36), 454.2027 [M-CH_3_]^+^ (100); Calcd exact mass for C_31_H_27_N_5_ (469.2266). Calcd: C, 79.29; H, 5.80; N, 14.91%. Found: C, 79.21, H, 5.93, N, 14.85%.
5-(4′-Diethylamino-[1,1′]-biphenyl-4-yl)-3-phenyl-[1,2,4]triazolo[4,3-*c*]quinazoline (**11**). The general procedure was applied using 5-(4-bromophenyl)-3-phenyl- [1,2,4]triazolo[4,3-*c*]quinazoline **9** and 4-(diethylamino)phenyl boronic acid as the starting materials. Reaction time is 11 h. Column chromatography: SiO_2_, hexane and EtOAc (1:1) with addition of CF_3_COOH and then Et_3_N. Pale-yellow solid, yield: 20% (38 mg); mp = 175–177 °C; ^1^H NMR (CDCl_3_, 400 MHz): δ 1.21 (6H, t, ^3^*J* = 7.1, 2CH_3_), 3.41 (4H, q, ^3^*J* = 7.1, 2CH_2_) 6.74 (2H, d, ^3^*J* = 8.2, Et_2_NC_6_H_4_), 7.08–7.11 (2H, m), 7.19–7.24 (5H, m), 7.29–7.31 (2H, m), 7.35 (2H, d, ^3^*J* = 8.4), 8.72–8.76 (1H, m, H-8), 8.80–8.83 (1H, m, H-9), 8.04–8.06 (1H, m, H-10), 8.75 (1H, d, ^3^*J* = 8.2, H-7); ^13^C {^1^H} NMR (CDCl_3_, 100 MHz): δ 12.8 (2CH_3_), 44.6 (2CH_2_), 111.9, 116.5, 123.6, 125.3, 126.6, 127.9, 127.9, 128.1, 128.4, 129.1, 129.3, 129.5, 129.8, 131.9, 141.4, 143.6, 146.0, 147.8, 149.2, 150.2; EIMS (*m*/*z*, I_rel_%): 470.2300 [M + 1]^+^ (30), 469.2266 [M]^+^ (78), 455.2110 (37), 454.2027 [M-CH_3_]^+^ (100); Calcd exact mass for C_31_H_27_N_5_ (469.2266).Calcd: C, 79.29; H, 5.80; N, 14.91%. Found: C, 79.23, H, 5.91, N, 14.86%.
5-(4-(9,9′-Spirobi[fluoren]-2-yl)phenyl)-2-phenyl-[1,2,4]triazolo[1,5-*c*]quinazoline (**12a**). The general procedure was applied using 5-(4-bromophenyl)-2-phenyl- [1,2,4]triazolo[1,5-*c*]quinazoline **8a** and 9,9′-spirobifluorene-2-boronic acid as the starting materials. Reaction time is 14 h. Column chromatography: SiO_2_, EtOAc and petroleum ether from (2:8) to (3:1). Pale-yellow solid, yield: 83% (75 mg); mp = 175–177 °C; ^1^H NMR (DMSO-d_6_, 400 MHz): δ 6.62–6.64 (1H, m), 6.70–6.72 (2H, m), 7.02 (1H, d, ^4^*J* = 1.0, spirobifluorene) 7.16–7.20 (3H, m), 7.42–7.44 (3H, m), 7.56–7.58 (3H, m), 7.77–7.82 (3H, m), 7.94–7.97 (2H, m), 8.08–8.12 (4H, m), 8.20–8.22 (1H, m), 8.30–8.32 (3H, m), 8.53 (1H, d, ^3^*J* = 8.2, H-7), 8.63 (2H, d, ^3^*J* = 8.7, H-2″, H-6″); ^13^C {^1^H} NMR (CDCl_3_, 100 MHz): δ 117.5, 120.2,120.4, 120.6, 123.0, 123.9, 124.2, 124.3, 127.0, 127.2, 127.8, 128.0, 128.1, 128.2, 128.8, 128.9, 130.4, 130.5, 130.6, 131.0, 132.1, 140.0, 141.3, 142.0, 143.1, 144.0, 146.2, 148.8, 149.4, 149.9, 153.1, 164.1; EIMS (*m*/*z*, I_rel_%): 637.2348 [M + 1]^+^ (50), 636.2314 [M]^+^ (100); Calcd exact mass for C_37_H_29_N_5_ (636.2314). Calcd: C, 81.74, H, 5.38, N, 12.88%. Found: C, 81.86, H, 5.47, N, 12.63%.
5-(4-(9,9′-Spirobi[fluoren]-2-yl)phenyl)-2-ethyl-[1,2,4]triazolo[1,5-*c*]quinazoline (**12b**). The general procedure was applied using 5-(4-bromophenyl)-2-ethyl-[1,2,4]triazolo[1,5-*c*]quinazoline **8b** (0.12 g, 0.34 mmol) and 9,9′-spirobifluorene-2-boronic acid as the starting materials. Reaction time is 14 h. Column chromatography: SiO_2_, hexane and EtOAc (2:8). Colourless solid, yield: 33% (65 mg); mp = 289–291 °C; ^1^H NMR (CDCl_3_, 400 MHz): δ 1.46 (3H, t, ^3^*J* = 8.1, CH_3_), 3.03 (2H, q, ^3^*J* = 8.1, CH_2_), 6.75—6.77 (1H, m), 6.79–6.81 (2H, m), 7.05 (1H, d, ^4^*J* = 1.1, spirobifluorene) 7.12–7.16 (3H, m), 7.38–7.41 (3H, m), 7.64–7.69 (3H, m), 7.72–7.75 (1H, m), 7.79–7.83 (1H, m), 7.87–7.91 (3H, m), 7.95–7.97 (1H, m), 8.07–8.09 (1H, m), 8.52 (1H, d, ^3^*J* = 8.3, H-7), 8.56 (2H, d, ^3^*J* = 8.8, H-2″, H-6″); ^13^C {^1^H} NMR (CDCl_3_, 100 MHz): δ 12.8 (CH_3_), 22.5 (CH_2_), 117.3, 120.2, 120.3, 120.6, 123.0, 123.7, 124.3, 127.0, 127.2, 128.0, 128.1, 128.2, 128.8, 130.7, 130.9, 132.0, 140.0, 141.4, 142.0, 143.1, 144.0, 146.1, 148.7, 149.4, 149.9, 152.6, 168.5; EIMS (*m*/*z*, I_rel_%): 589.2348 [M + 1]^+^ (47), 588.2314 [M]^+^ (100), 294.0905 (31); Calcd exact mass for C_42_H_28_N_4_ (588.2314). Calcd: C, 85.69; H, 4.79; N, 9.52%. Found: C, 85.62, H, 4.89, N, 9.63%.

## 4. Conclusions

Cross-coupling reactions of 5-chloro-2-aryl-[1,2,4]triazolo[1,5-*c*]quinazolines were achieved for the first time, and a series of novel donor–acceptor 2-aryl[1,2,4]triazolo[1,5-*c*]quinazoline fluorophores were synthesized. Their photophysical characteristics were investigated in toluene, in MeCN and in the solid state. In toluene, 5-Aminoaryl-substituted 2-aryl[1,2,4]triazolo[1,5-*c*]quinazoline show long-wavelength absorption at 325–425 nm and emission at 420–459 nm; variation in the triazole-aryl substituent has little effect, whereas the electronic nature of the C-5 aryl donor strongly influences the photophysics. The compounds display strong blue emission with Φ_F_ > 95% in toluene and > 52% in MeCN, while introducing a 9,9′-spirobisfluorene at C-5 induces blue-shifted absorption/emission and a pronounced reduction in Φ_F_ across solvents. 5-Biphenyl-2-phenyl-[1,2,4]triazoloquinazoline derivatives bearing an additional phenylene π-spacer, prepared from 2-(4-bromophenyl)-4-hydrazinoquinazoline and orthoester, show a significant bathochromic shift in their emission bands. Furthermore, the [4,3-c]-annulated analogue exhibits a hypsochromic shift in its absorption maximum due to reduced molecular planarity and shorter conjugation length induced by steric hindrance from neighbouring substituents.

Several compounds displayed pronounced positive solvatochromism, which is indicative of ICT excited state. Additionally, responses to acid stimuli were assessed using trifluoroacetic acid as a protonating agent. Observed acidochromic shifts and fluorescence quenching patterns varied depending on the compound’s structure and could be attributed to differences in conjugation effects and preferred protonation sites.

Experimental observations were corroborated through DFT calculations of frontier molecular orbitals and electronic transitions. Computed HOMO–LUMO energy levels and electron density distributions provided insights into the nature of the ICT processes.

In summary, these triazoloquinazoline-based fluorophores exhibit tunable photophysical properties, environmental responsiveness, and structural flexibility. They are thus highly promising candidates for use as active materials in organic optoelectronics and as fluorescent sensors for monitoring parameters such as solvent polarity or pH. The 2-aryl[1,2,4]triazolo[1,5-*c*]quinazoline framework appears particularly attractive to modifications that can yield new classes of fluorescent chemosensors capable of detecting diverse analytes, ranging from charged species (both cations and anions) to small molecules and even biomolecules.

## Figures and Tables

**Table 1:** Photophysical properties of fluorophores **6a**–**j**, **10**, **11**, and **12a**,**b** in solution (c = 10^−5^ M) and in the solid state at r.t.

Comp.	Solvent/ Solid State	λ_abs_, nm, ε (10^4^ M^−1^cm^−1^)	λ_em_ ^b^, nm	Δν_st_ ^c^, cm^−1^	Φ_F_ ^d^,%
**6a**	Toluene	376 (2.64)	425	3070	>95
	MeCN	284 (2.35), 376 (3.53)	459	4810	>95
	Solid	-	511	-	*
**6b**	Toluene	383 (1.92)	459	4320	81
	MeCN	252 (4.21), 285 (2.30), 375 (2.61)	544	8280	51
	Solid	-	456	-	45
**6c**	Toluene	342 (1.47), 355 (1.48)	423	4530	>95
	MeCN ^a^	254, 277 sh, 340	507	9690	87
	Solid	-	459, 481 (sh)	-	20
**6d**	Toluene	369 (1.96)	421	3350	>95
	MeCN	253 (4.18), 284 (2.56), 367 (2.69)	457	5370	>95
	Solid	- *	433	-	*
**6e**	Toluene	289 (3.35), 382 (2.49)	459	4390	91
	MeCN	259 (1.23), 284 (0.87), 375 (0.73)	539	8110	52
	Solid	-	492	-	38
**6f**	Toluene	342 (0.95), 355 (0.92)	421	4420	>95
	MeCN ^a^	259, 278 sh, 340	506	9650	88
	Solid	-	434	-	28
**6g**	Toluene	376 (2.88)	425	3070	>95
	MeCN	254 (4.39), 286 (2.48), 376 (3.19)	460	4860	>95
	Solid	-	448, 474	-	*
**6h**	Toluene	292 (1.23), 381 (1.43)	457	4370	>95
	MeCN	259 (1.43), 284 (1.03) sh, 375 (0.77)	540	8150	53
	Solid	-	463	-	37
**6i**	Toluene	342 (0.92), 355 (0.89)	420	4360	>95
	MeCN ^a^	284, 278 sh, 340	506	9650	84
	Solid	-	432	-	*
**6j**	Toluene	296 (2.48), 309 (2.73), 339 (2.58)	376, 396, 417 sh	4250 ^e^	7
	MeCN	252 (1.90), 270 (1.48), 307 (1.09), 330 (1.0)	400	5300	4
	Solid		403, 422	-	*
**10**	Toluene	384 (1.88)	481	5250	>95
	MeCN	222 (0.53), 262 (1.71), 377 (0.83)	601	9890	35
	Solid	-	513	-	6
**11**	Toluene	363 (1.21)	493	8324	17
	MeCN	271 (7.34), 356 (1.46)	616	11,860	34
	Solid	-	492	-	*
**12a**	Toluene	284(9,08), 309(6,00)	398, 416, 440 sh	8324	19
	MeCN	296 (1,54), 308(1,87), 335(2,26)	442	7226	77
	Solid	-	431, 449, 482 sh	-	*
**12b**	Toluene	309 (2.87), 337 (3.04)	392, 412, 437 sh	5402 ^e^	60
	MeCN ^a^	308, 331	433	7120	>95
	Solid	-	417, 439, 469 sh	-	*

^a^ Poor soluble. ^b^ Excitation at the lowest energy absorption peak. ^c^ Stokes shift was calculated relative to the lowest energy absorption peak and rounded to tens. ^d^ Absolute fluorescence quantum yield was measured according to a reported procedure [17] using Horiba-Fluoromax-4 spectrofluorometer equipped with integrating sphere. ^e^ Relative to middle peak of emission. * Data was not measured.

**Table 2:** Solvent cavity (Onsager) radius and data from Ravi plot for quinazolines **6b**, **6c**, and **10**.

Comp.	*a*, ^a^ A	Slope (m)	R^2^	Δμ, D	μ(S_0_)^DFT^, D	μ*(S_1_)^DFT^, D	∆μ^DFT^, D
**6b**	5.79	9539	0.98	7.46	1.6670	7.1272	5.46
**6c**	5.78	13,735	0.95	8.93	0.9471	9.9854	9.04
**10**	5.71	11,343	0.97	7.97	3.9203	12.7510	8.83

^a^ Solvent cavity radius, calculated from Suppan’s equation [28]: *a* = $\sqrt[{3}]{\frac{3M}{4N\pi \delta }}$, where with δ is the density of the solute (δ is assumed to 1.0 g/cm^3^), *M* is the molecular weight of the solute, and *N* the Avogadro number.

## Data Availability

The data are available on request from the corresponding authors.

## Supplementary Materials

The following supporting information can be downloaded at: https://www.mdpi.com/article/10.3390/molecules30224420/s1, Figures S1 and S2. Proposed reaction mechanisms; Figures S3–S12. NMR and mass spectra of intermediates; Figure S13. Molecular structure of **6**, **10**, **11** and atoms numbering; Figures S14–S27. NMR and mass spectra of target products **6a**–**j**, **10**, **11**, and **12a**,**b**; Tables S1–S8: Selected bond lengths and angles of compounds **6g**, **10**, and **12a**; Figure S28. Absorption and emission spectra of compounds **6a**–**j**, **10**, **11**, and **12a**,**b** in toluene and MeCN; Table S9. Photophysical properties of previously reported fluorophores of series **F** and **E**, and compounds **10** and **11**, in solution. Figure S29. Combined solid-state emission spectra of compounds **6a**–**j**, **10**, **11**, and **12a**,**b**; Figure S30. Changes in emission colour of compounds **6a**–**c**, **10**, and **11** in different solvents; Figure S31. Combined emission spectra (non-normalized and normalized) of compounds **6a**, **6b**, **6c**, and **10** in different solvents; Tables S10–S12. Empirical solvent polarity parameter (*E^N^_T_*), absorption and emission maxima (λ_abs_, λ_em_, nm) and Stokes shift (nm, cm^−1^) of **6b**, **6c**, and **10** in different solvents; Figure S32. Plots of Stokes shift in compounds **6b**, **6c**, and **10** versus Reichardt solvent polarity functions; Figure S33. Emission colour changes in compounds **6a** and **10** in toluene upon sequential addition of trifluoroacetic acid (TFA) and triethylamine (TEA). Figure S34. Absorption spectra of compounds **6a** (a) and **10** (b) in pure toluene and upon addition of excess of CF_3_COOH (TFA). Figures S35 and S36. Absorption and emission spectra changes of 6a and 10 in toluene upon sequential addition of CF_3_COOH (TFA) and Et_3_N (TEA). Figure S37. ^1^H NMR spectra of compounds **6a** and **10** in pure DMSO-d_6_ and upon addition of excess of TFA-d_1_. Table S13. The electronic distribution in HOMO/LUMO of **6a**–**c**,**e**,**h**,**j**, **10**, **11**, and **12a**,**b** for gas phase; Table S14. The optimized geometries of **6a**–**c**,**e**,**h**,**j**, **10**, **11** and **12a**,**b** in toluene and MeCN; Table S15. Selected dihedral angles (α), and bond lengths (L) in the optimized geometries of compounds **6a**–**c**,**e**,**h**,**j**, **10**, **11**, and **12a**,**b**, in ground (S_0_) and excited (S_1_) states. References are cited in [23,24,38,39,40]
