# Supplementary material for: Novel 5-Aryl-[1,2,4]triazoloquinazoline Fluorophores: Synthesis, Comparative Studies of the Optical Properties and ICT-Based Sensing Application
Source: Molecules. 2025 Nov 15;30(22):4420. doi: 10.3390/molecules30224420 (PMC12655076; doi:10.3390/molecules30224420)
Supplement: Supplementary file 1 [file molecules-30-04420-s001.zip › molecules-3966064-supplementary.pdf]

Supporting Information

Novel 5-aryl-[1,2,4]triazoloquinazoline fluorophores: synthesis, comparative studies of the optical properties and ICT-based sensing application

Alexandra E. Kopotilova, Julia V. Permyakova, Ekaterina S. Starnovskaya, Tatyana N. Moshkina, Alexander S. Novikov, Pavel A. Slepukhin and Emiliya V. Nosova

CONTENTS

1. The proposed reaction mechanisms 2
2. NMR and mass spectra of intermediates 3
3. NMR and mass spectra of target products **6a-j**, **10**, **11** and **12a,b**........ 21
4. Crystallographic data of triazoloquinazolines 51
5. Absorption and emission spectra of compounds **6a-j**, **10**, **11** and **12a,b**........ 58
6. Solvatochromic properties of compounds **6**, **10** and **11** 64
7. Acid induced spectroscopic changes of compounds **6a-c**, **7** and **8**........ 70
8. Theoretical studies 75

1. The proposed reaction mechanisms

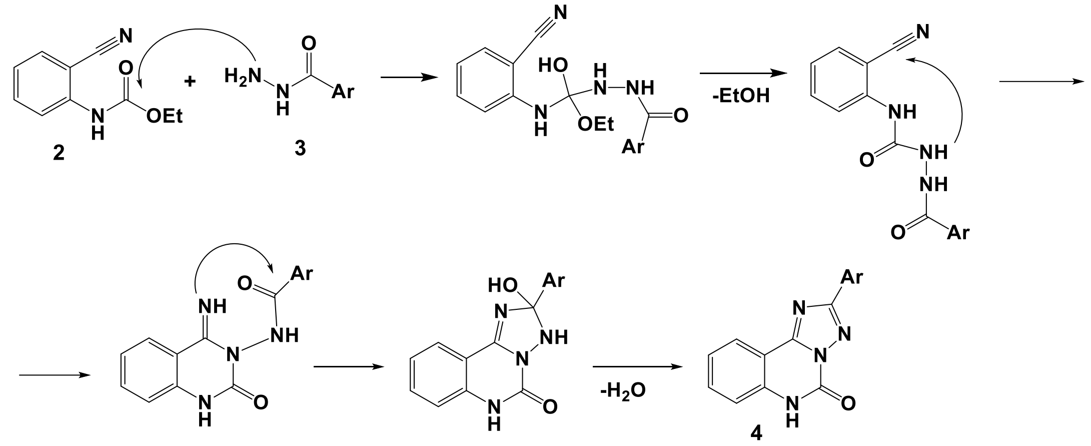

Figure S1. Proposed mechanism for the formation of 2-aryl-[1,2,4]triazolo[1,5-*c*]quinazolin-5-ones **4** from *N*-(2-cyanophenyl)carbamate **2** and arylhydrazides **3.** The mechanism based on literature data for similar precoursors [1,2].

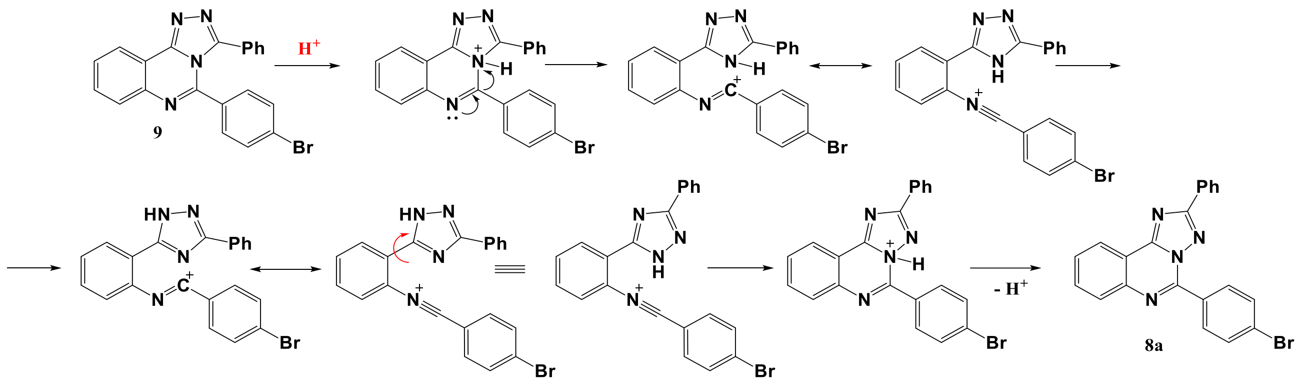

Figure S2. Proposed mechanism for Dimroth rearrangement of 3-phenyl-[1,2,4]triazolo[4,3-*c*]quinazoline **9** into 2-phenyl-[1,2,4]triazolo[1,5-*c*]quinazoline **8a** under reflux in acetic acid for 20 h [2].

2. NMR and mass spectra of intermediates

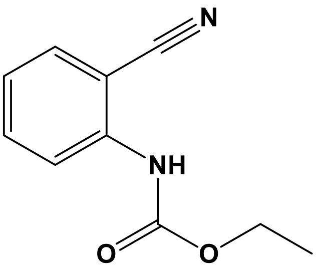

2

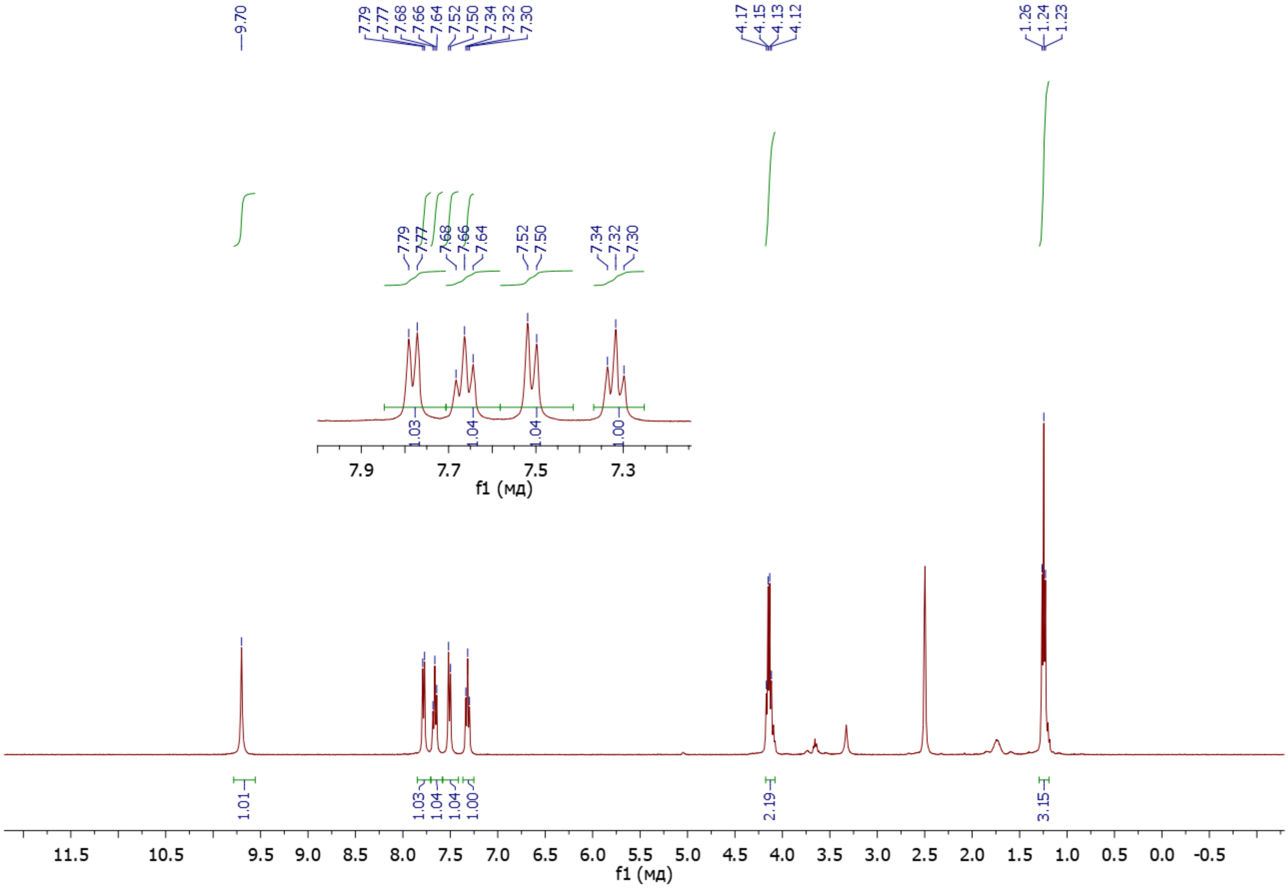

a

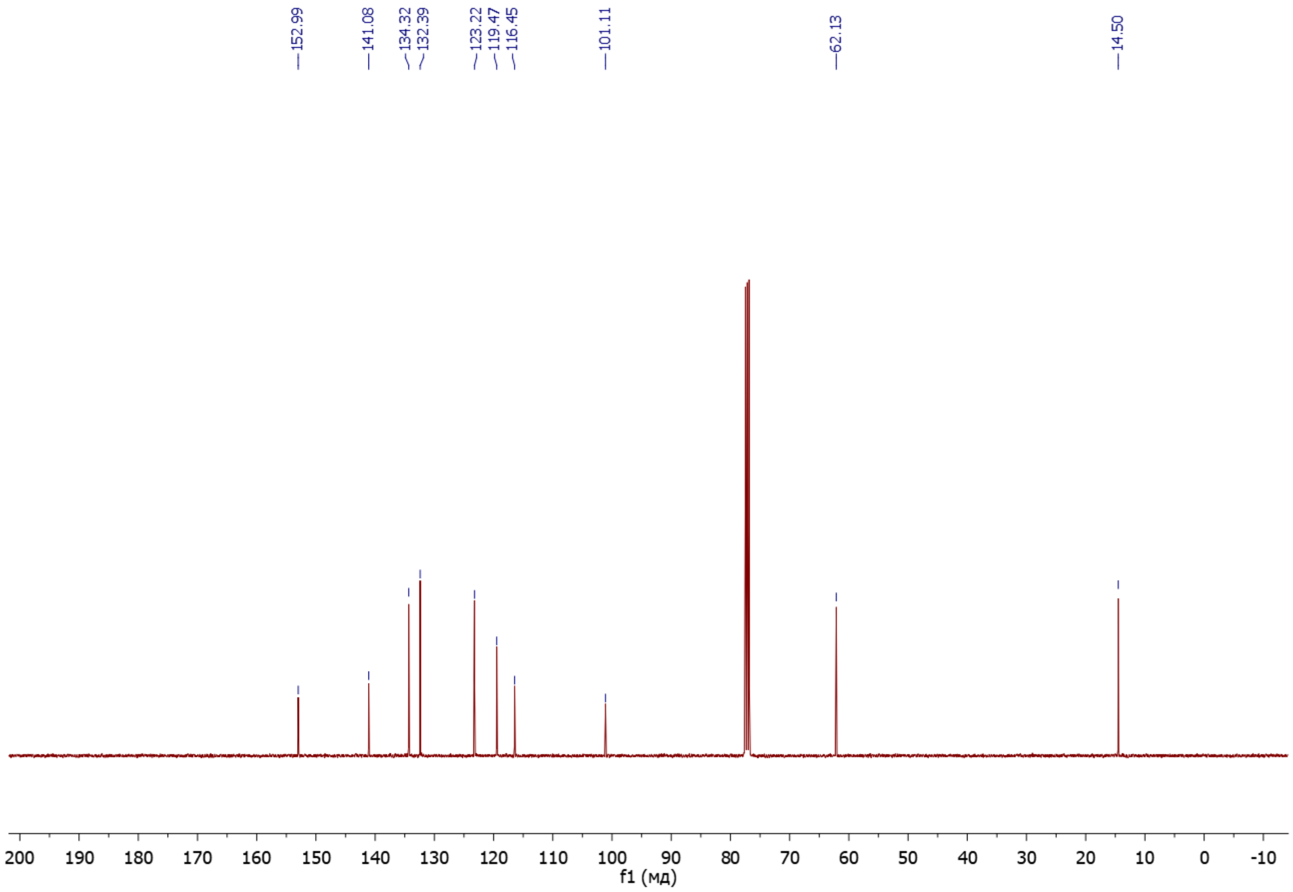

b

Figure S3. ^{1}H NMR (a) and ^{13}C NMR (b) spectra of **2**.

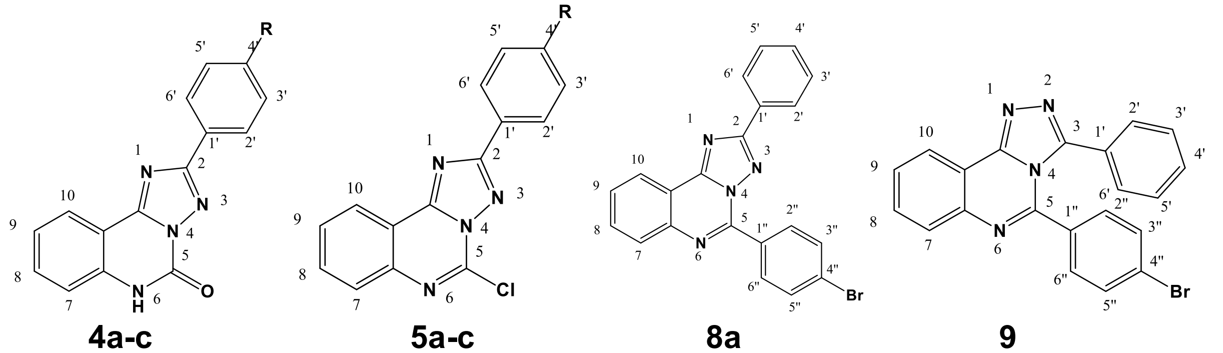

4a-c **5a-c** **8a** **9**

Figure S4. Molecular structure of compounds **4-9** and atoms numbering.

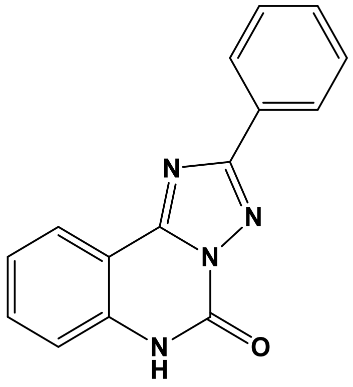

4a

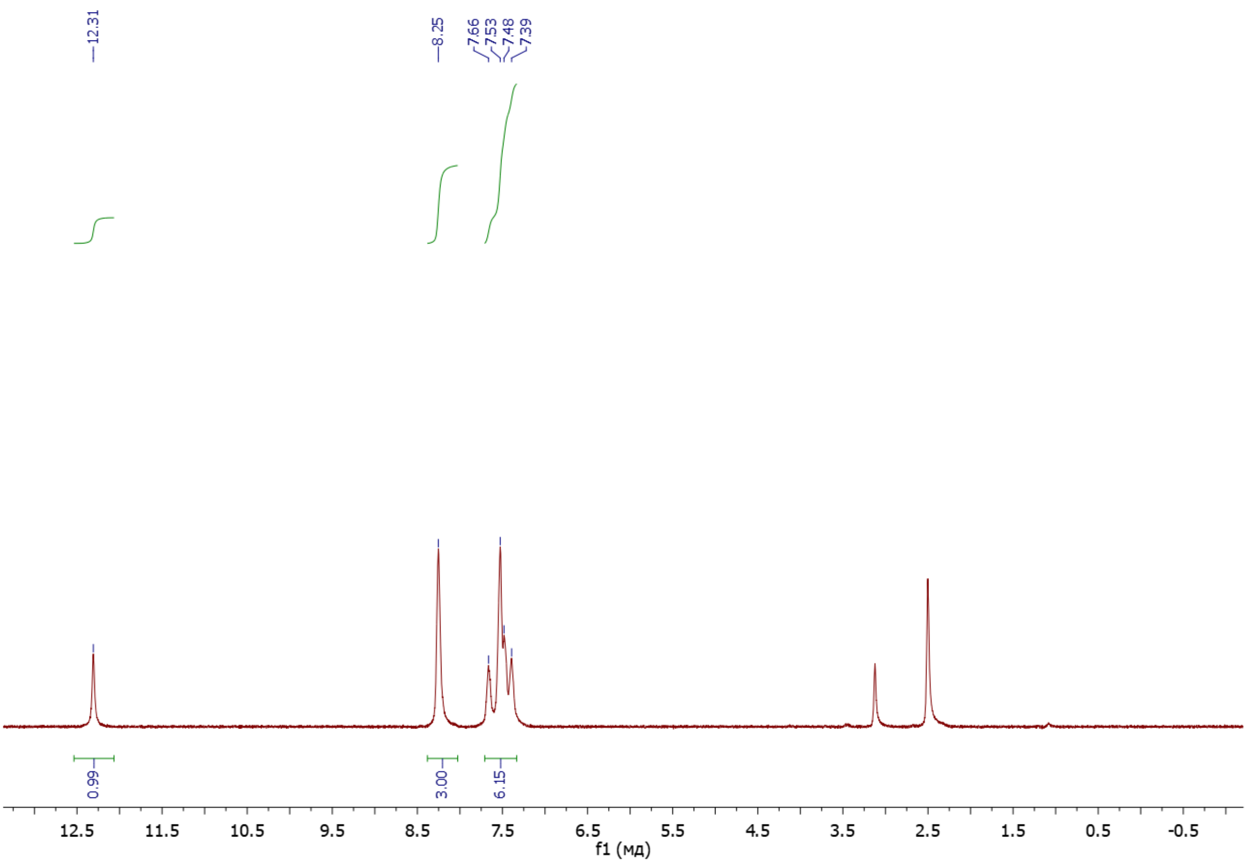

a

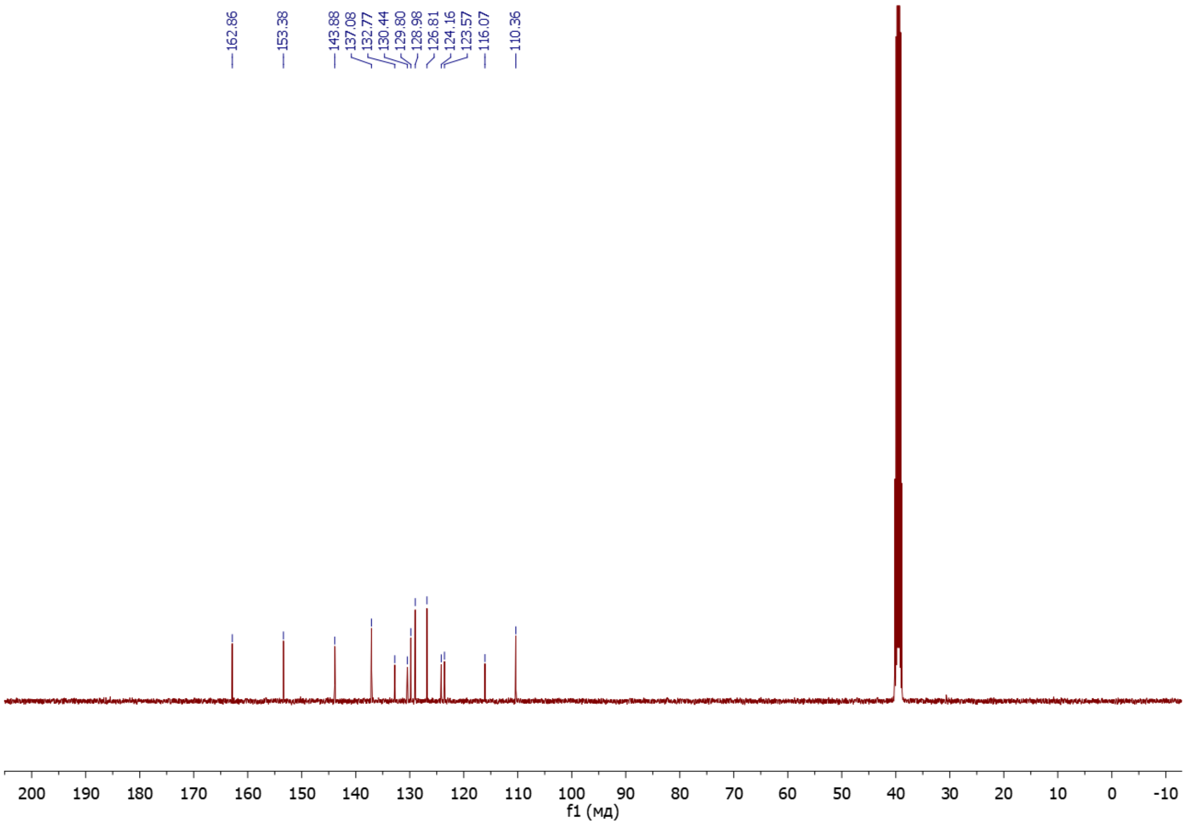

b

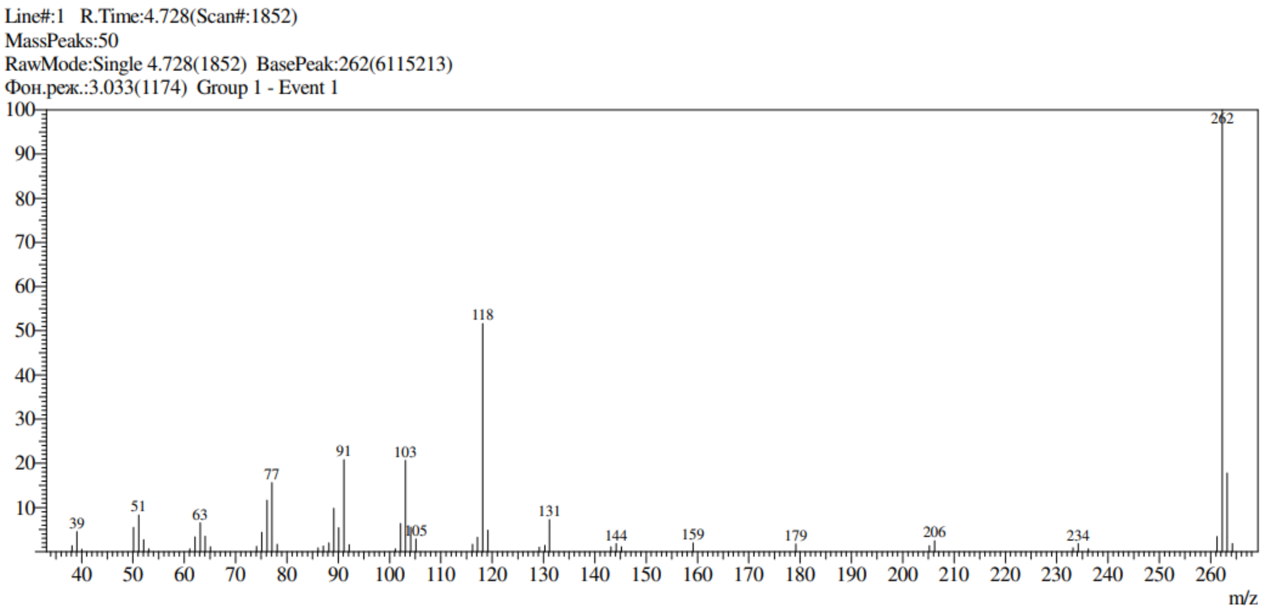

c

Figure S5. ^{1}H NMR (a) and ^{13}C NMR (b) and mass spectra (EI) (c) of **4a**.

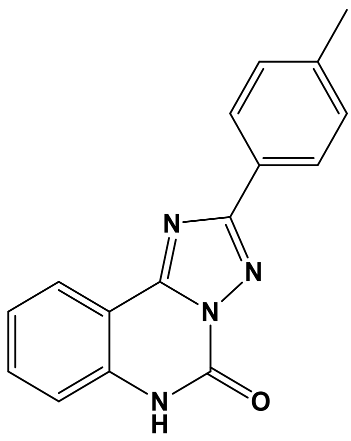

4b

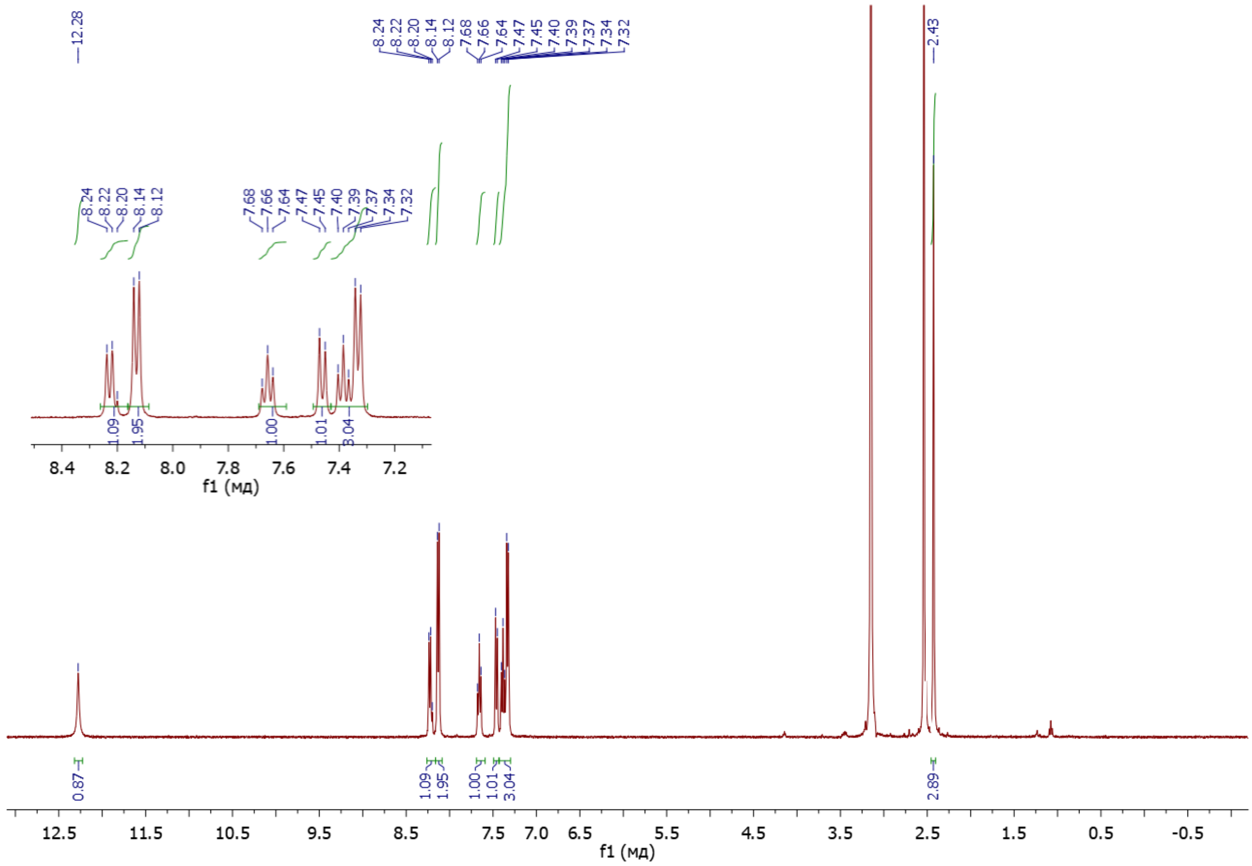

12.28
8.24
8.22
8.20
8.14
8.12
7.68
7.66
7.64
7.47
7.45
7.40
7.39
7.37
7.34
7.32
2.43
0.87
1.09
1.95
1.00
1.01
3.04
2.89
12.5
11.5
10.5
9.5
8.5
7.5
7.0
6.5
5.5
4.5
3.5
2.5
1.5
0.5
-0.5
f1 (мд)
8.4
8.2
8.0
7.8
7.6
7.4
7.2

a

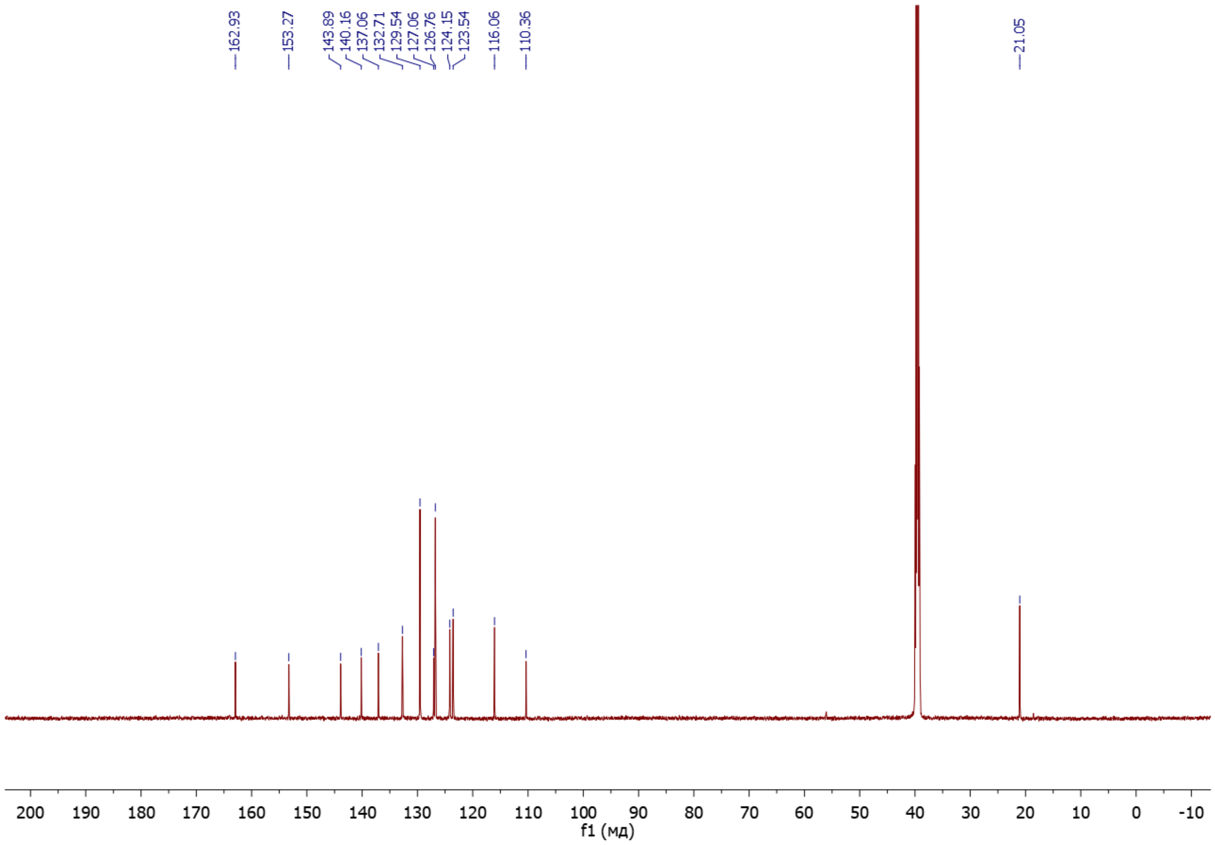

b

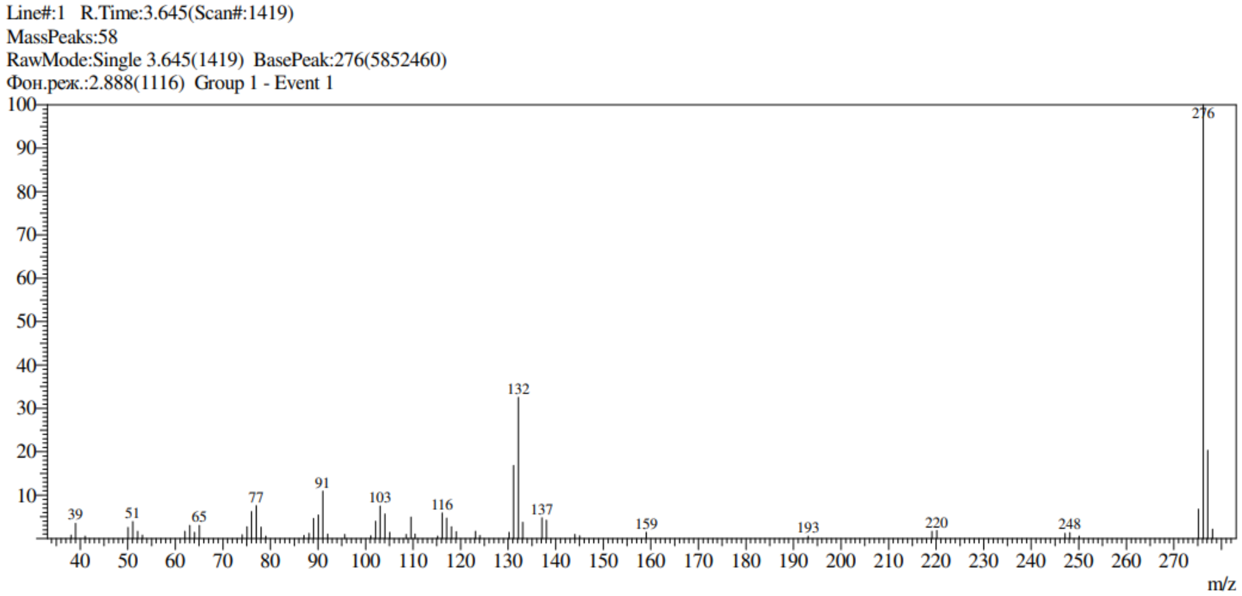

c

Figure S6. ^{1}H NMR (a) and ^{13}C NMR (b) and mass spectra (EI) (c) of **4b**.

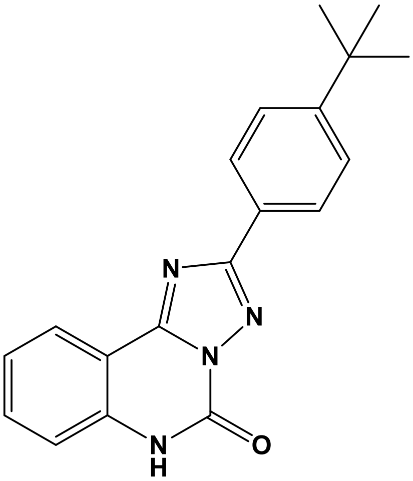
N
N
N
N
H
O

4c

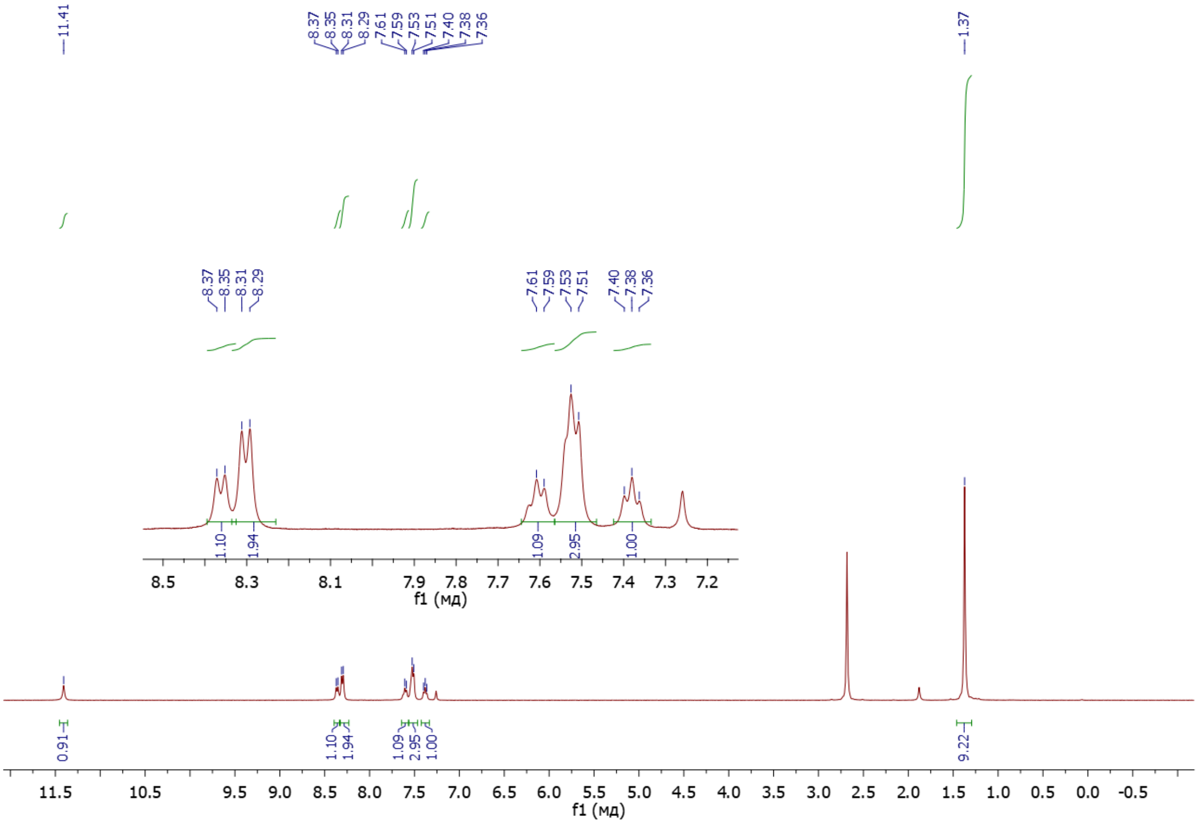
11.41
8.37
8.35
8.31
8.29
7.61
7.59
7.53
7.51
7.40
7.38
7.36
1.37
1.10
1.94
1.09
2.95
1.00
0.91
9.22
f1 (мд)
f1 (мд)

a

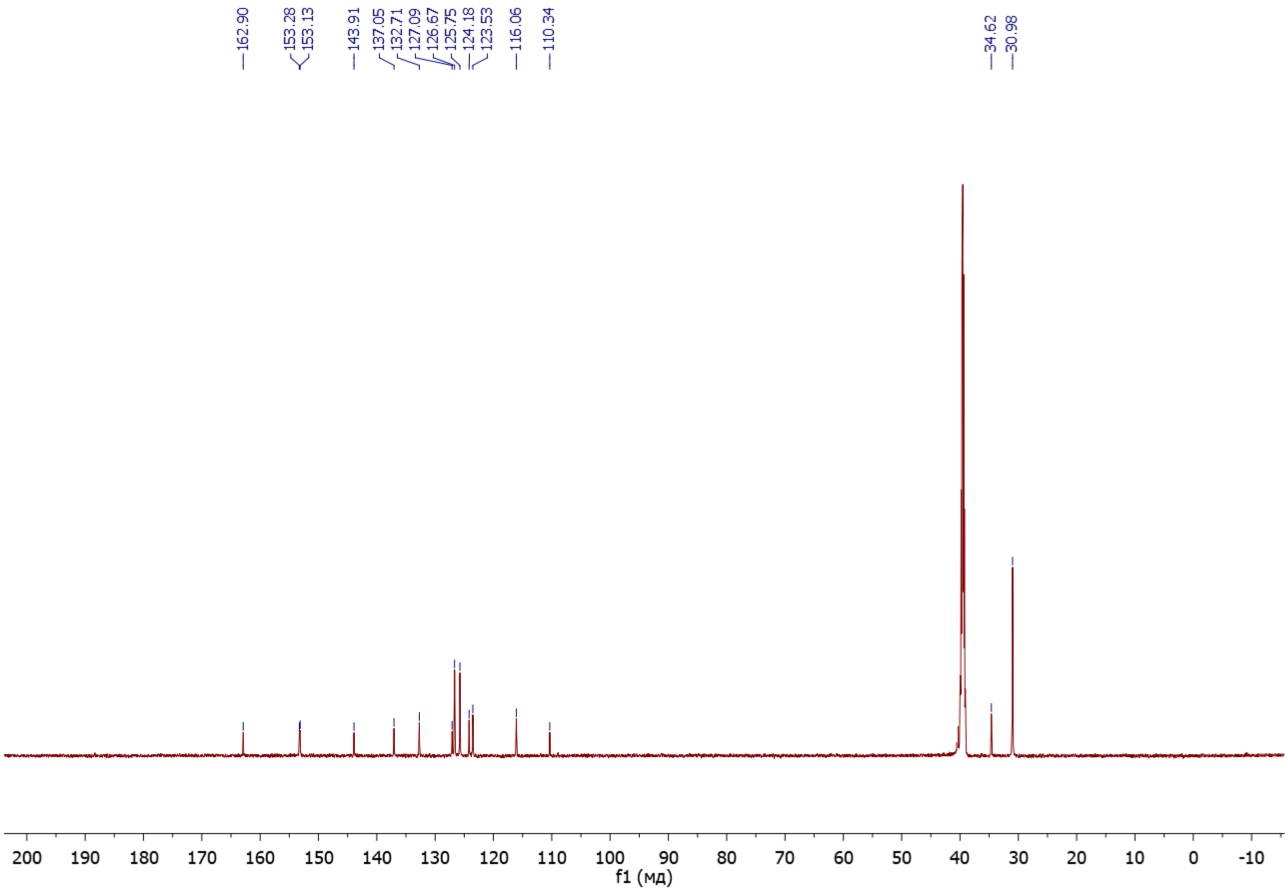

b

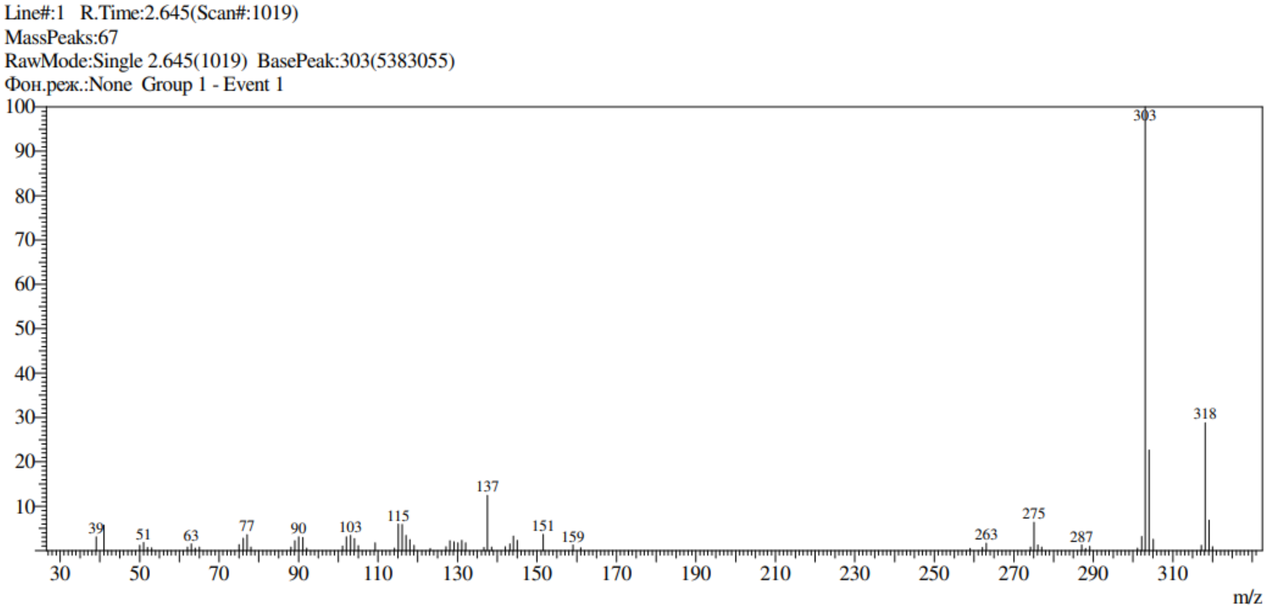

c

Figure S7. ^{1}H NMR (a) and ^{13}C NMR (b) and mass spectra (EI) (c) of **4c**.

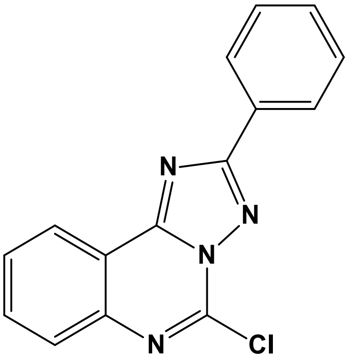
N
N
N
N
Cl

5a

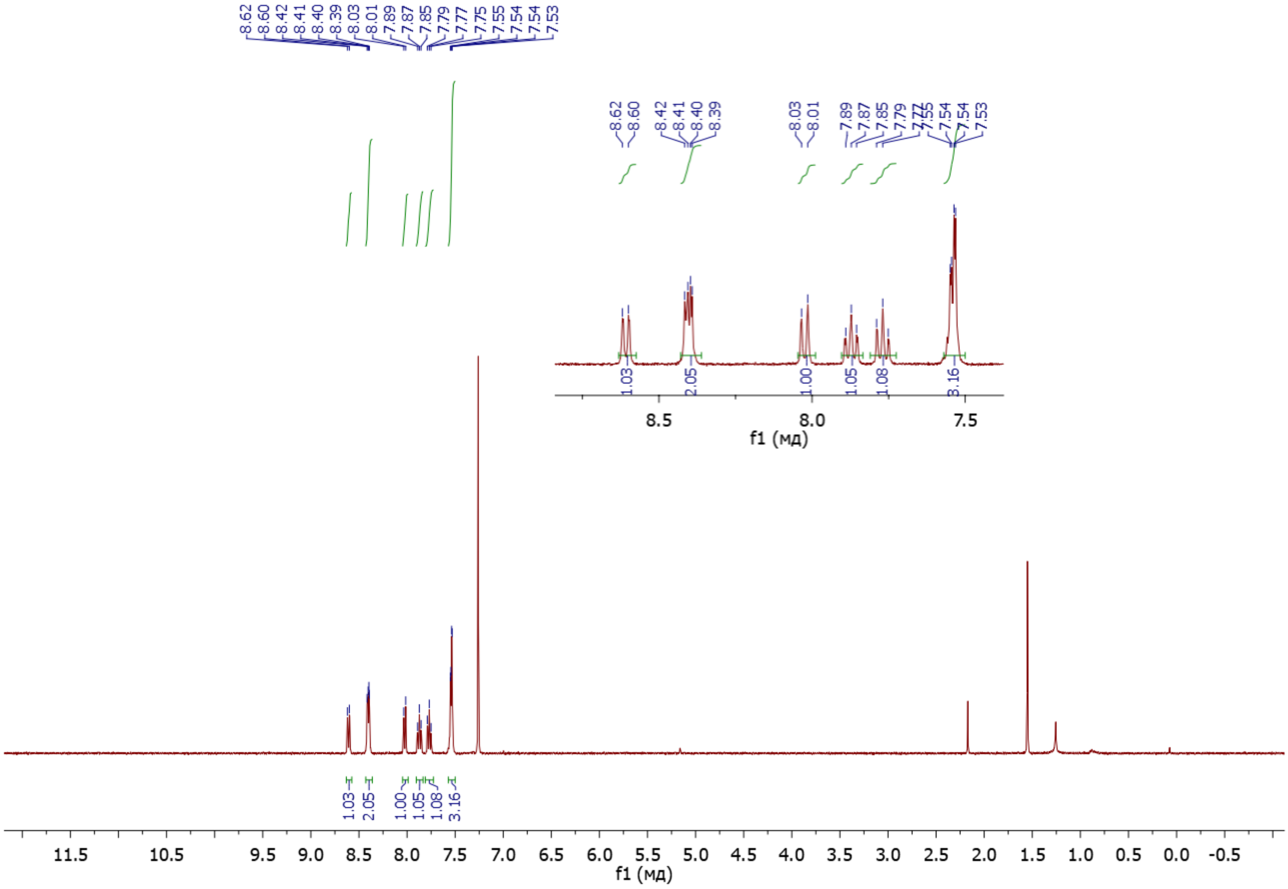
8.62
8.60
8.42
8.41
8.40
8.39
8.03
8.01
7.89
7.87
7.85
7.79
7.77
7.75
7.55
7.54
7.54
7.53
1.03
2.05
1.00
1.05
1.08
3.16
8.5
8.0
7.5
f1 (мд)
11.5
10.5
9.5
9.0
8.5
8.0
7.5
7.0
6.5
6.0
5.5
5.0
4.5
4.0
3.5
3.0
2.5
2.0
1.5
1.0
0.5
0.0
-0.5
f1 (мд)

a

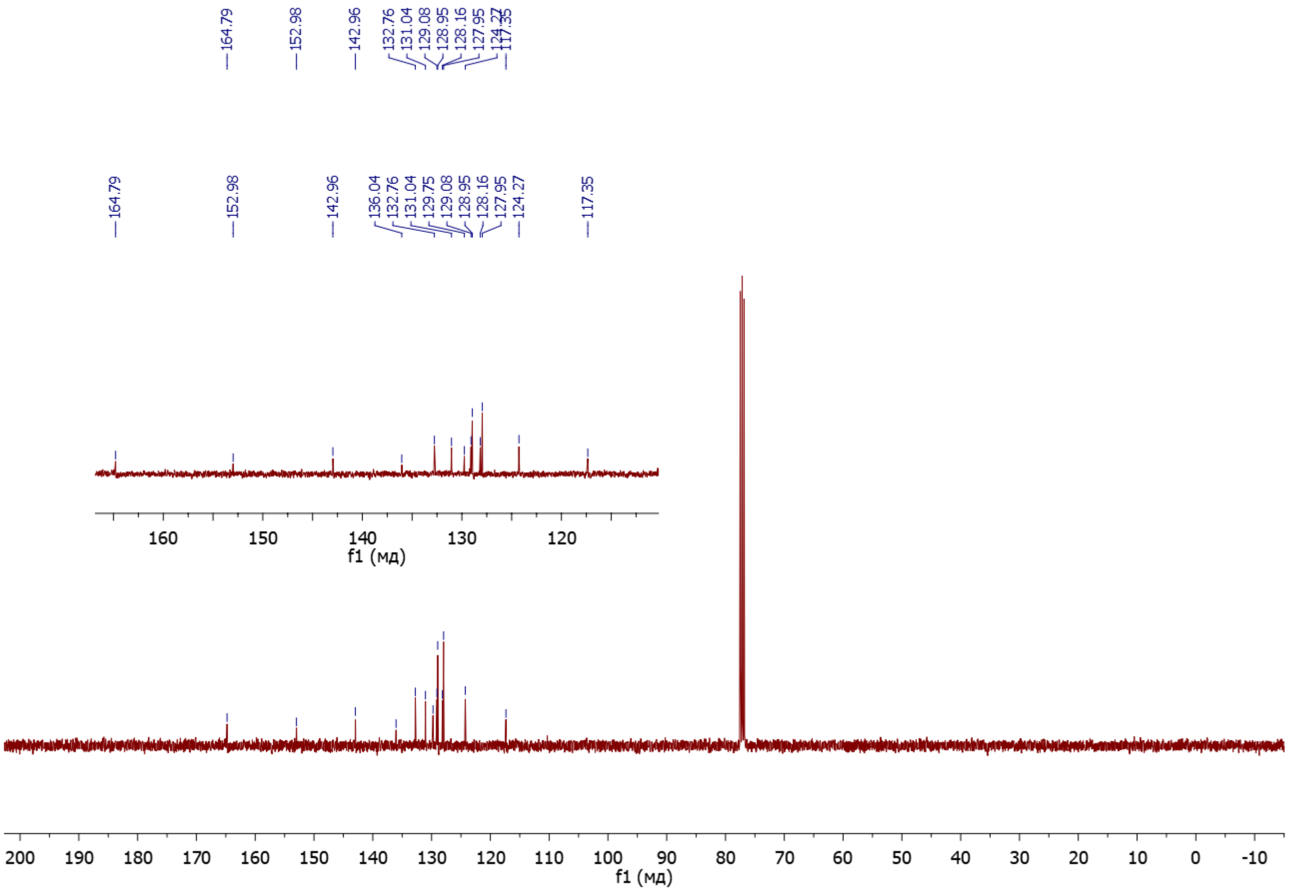

b

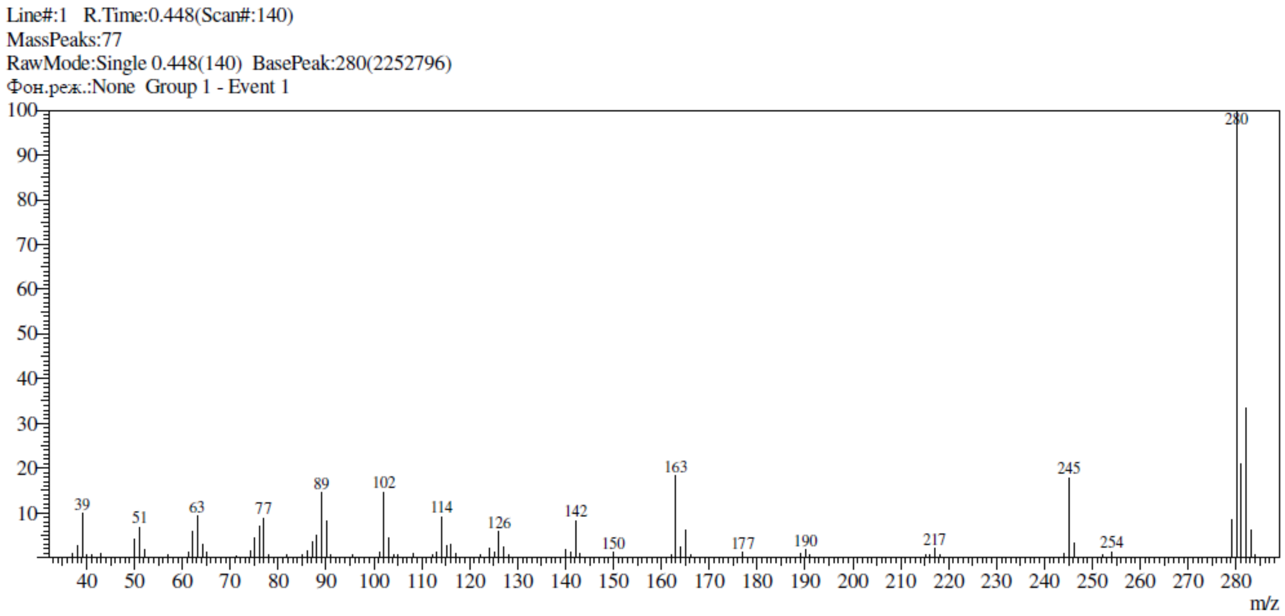

c

Figure S8. ^{1}H NMR (a) and ^{13}C NMR (b) and mass spectra (EI) (c) of **5a**.

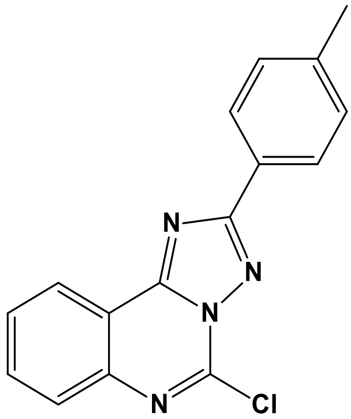

5b

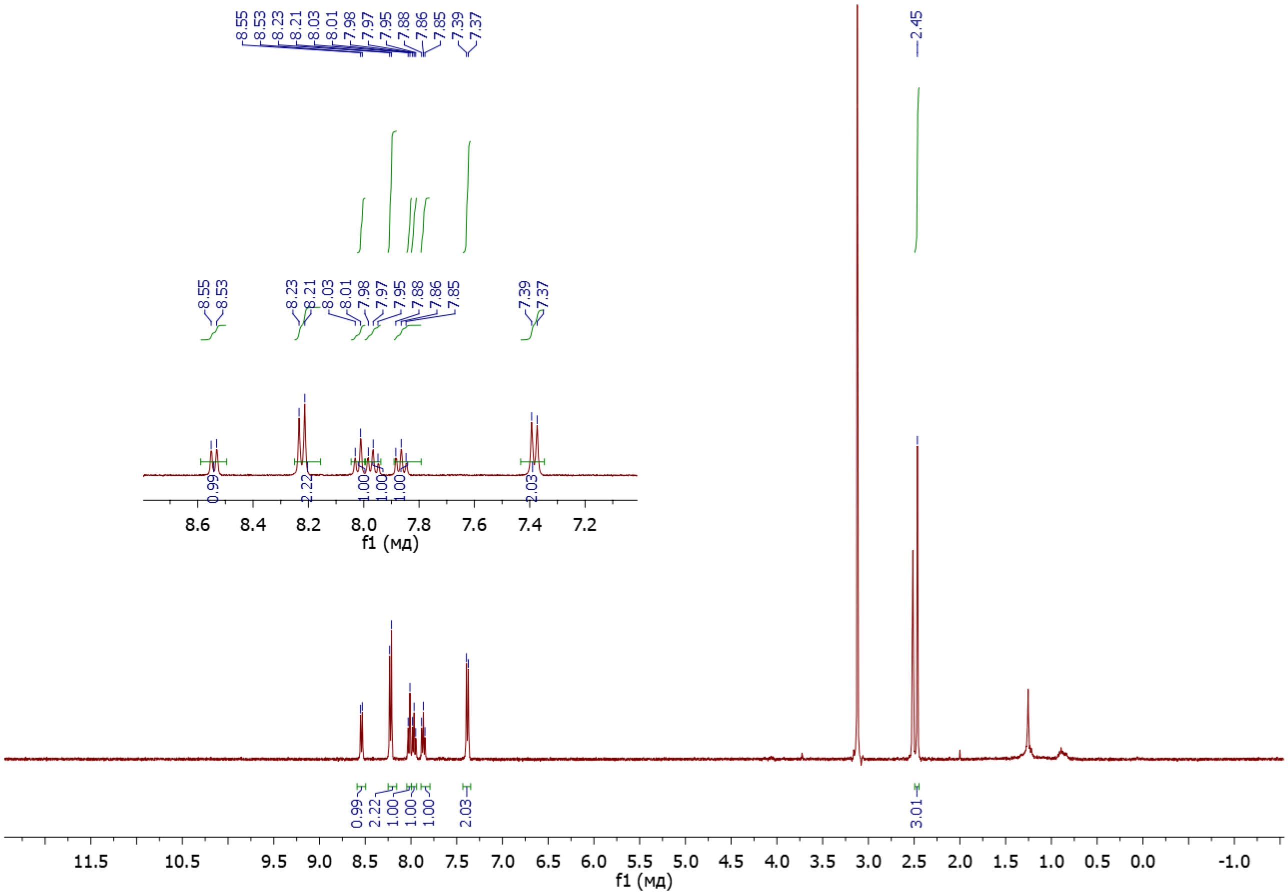
8.55
8.53
8.23
8.21
8.03
8.01
7.98
7.97
7.95
7.88
7.86
7.85
7.39
7.37
2.45
0.99
2.22
1.00
1.00
1.00
2.03
3.01
f1 (мд)

a

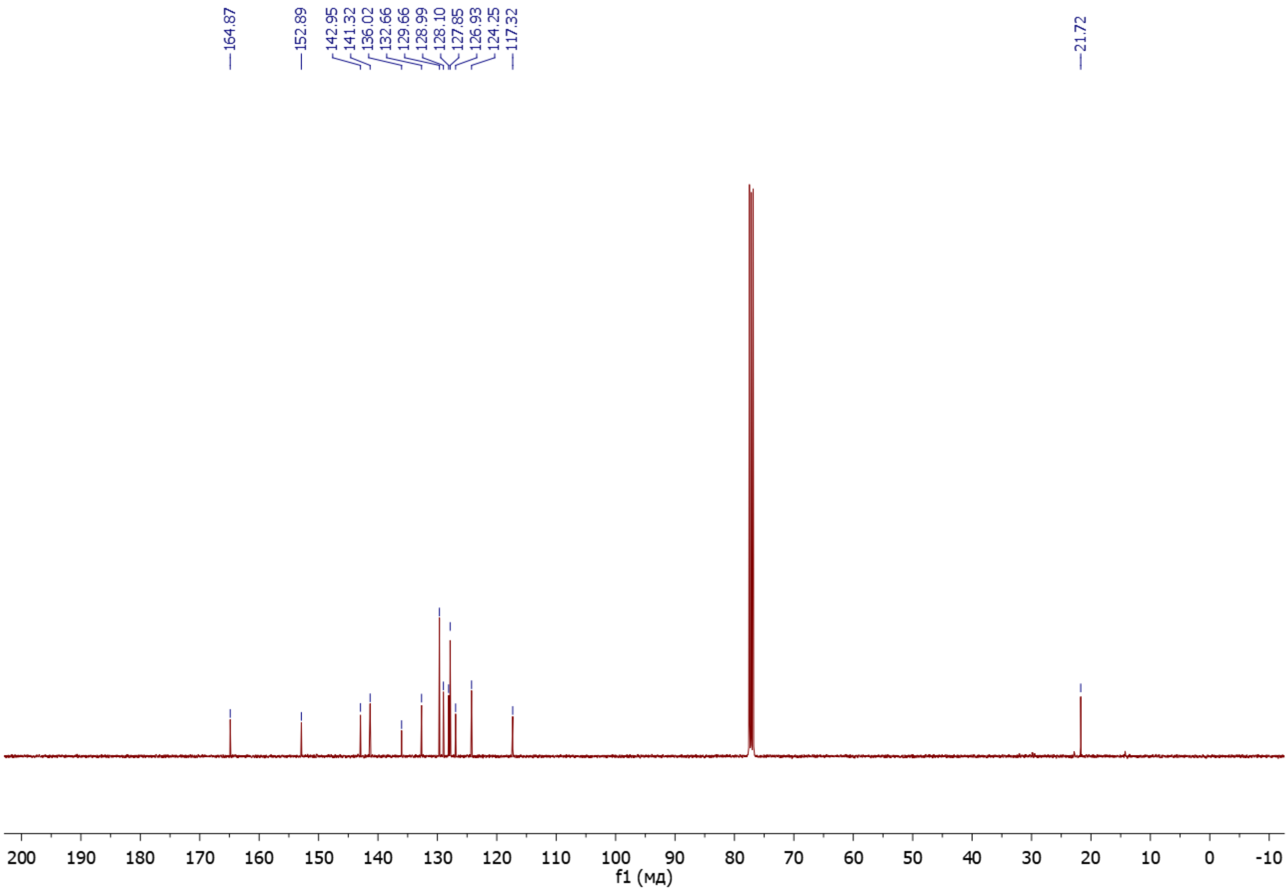

b

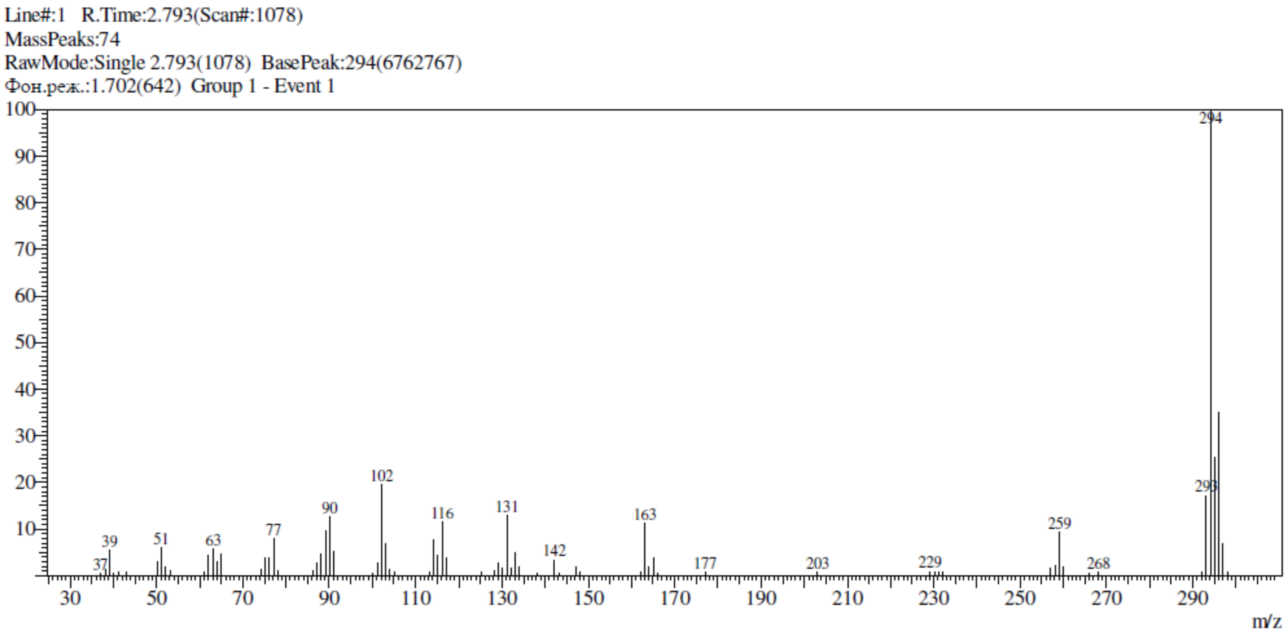

c

Figure S9. ^{1}H NMR (a) and ^{13}C NMR (b) and mass spectra (EI) (c) of **5b**.

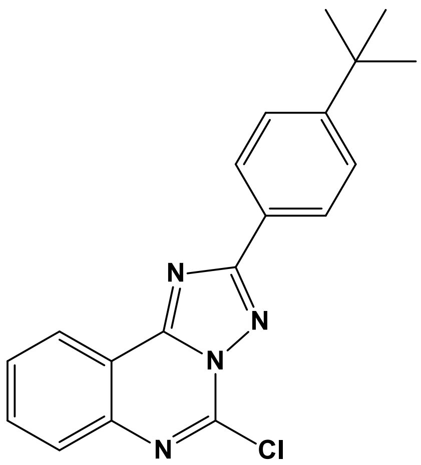

5c

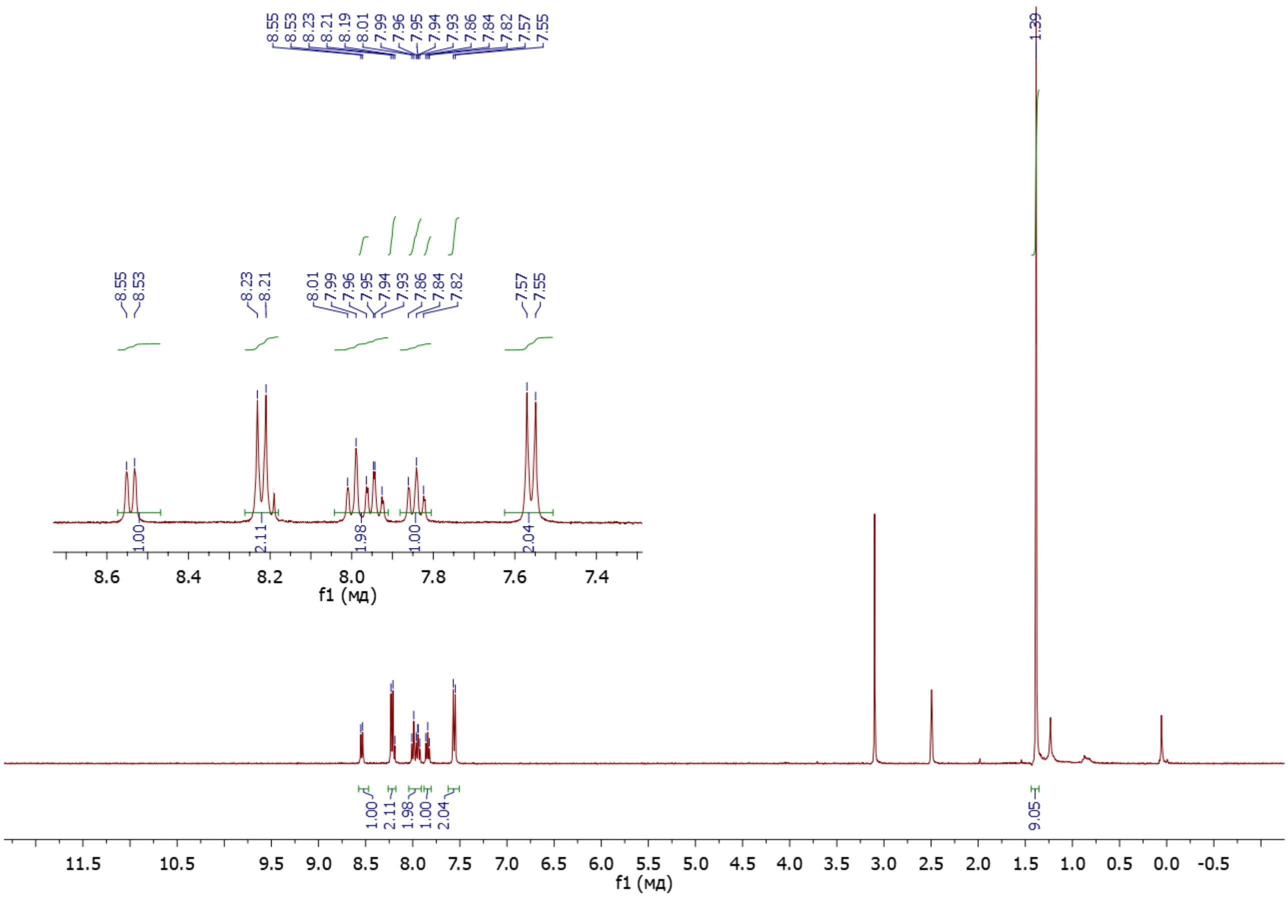
8.55 8.53 8.23 8.21 8.19 8.01 7.99 7.96 7.95 7.94 7.93 7.86 7.84 7.82 7.57 7.55
1.39
1.00 2.11 1.98 1.00 2.04
9.05
f1 (мд)

a

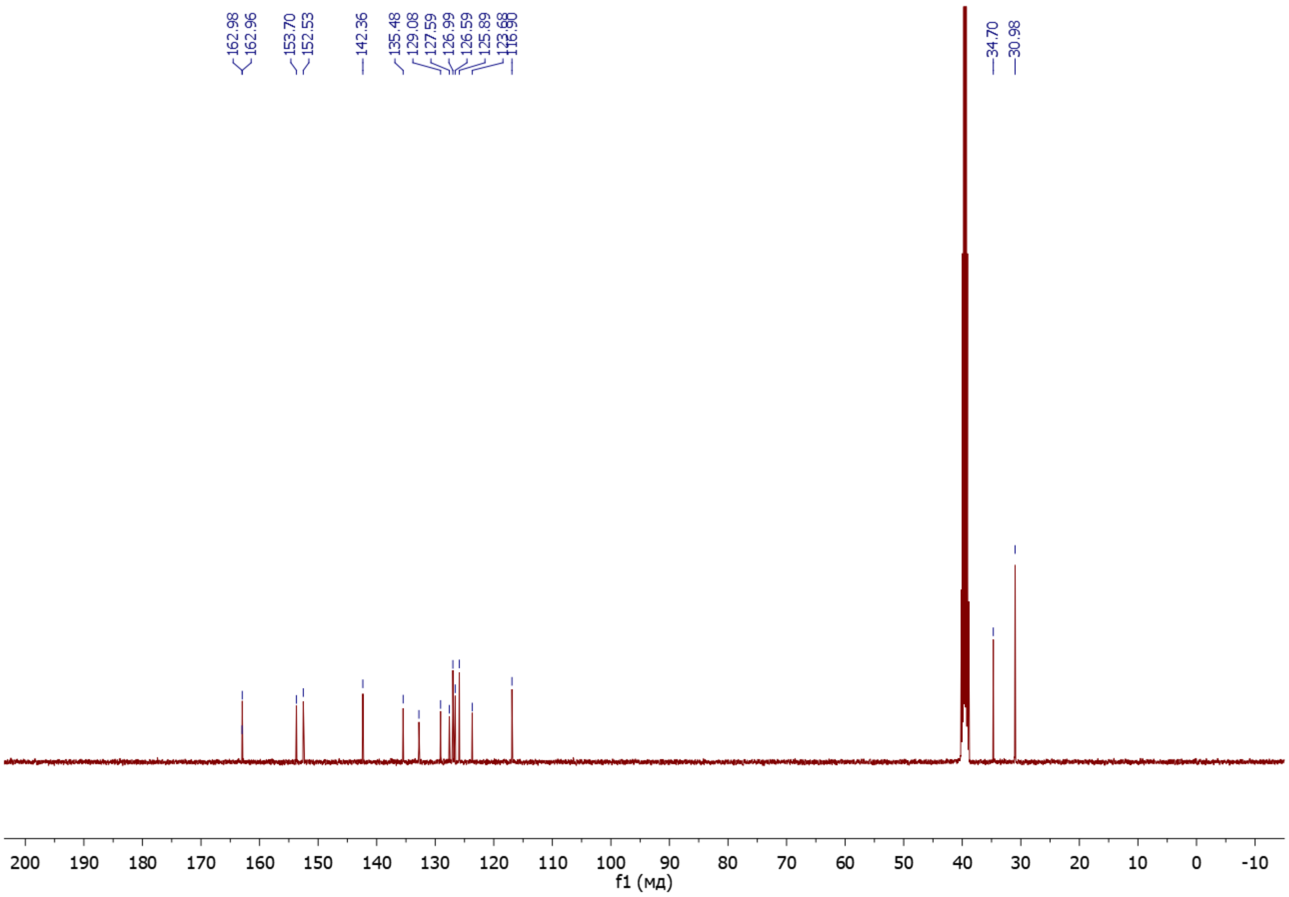

b

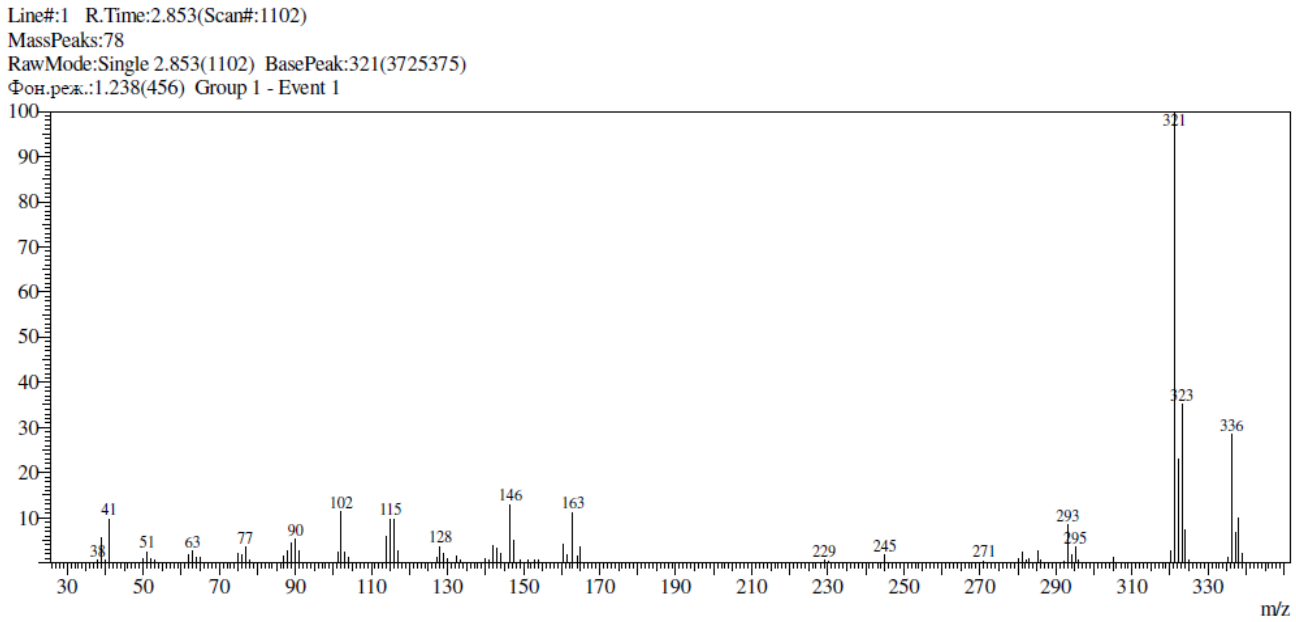

c

Figure S10. ^{1}H NMR (a) and ^{13}C NMR (b) and mass spectra (EI) (c) of of **5c**.

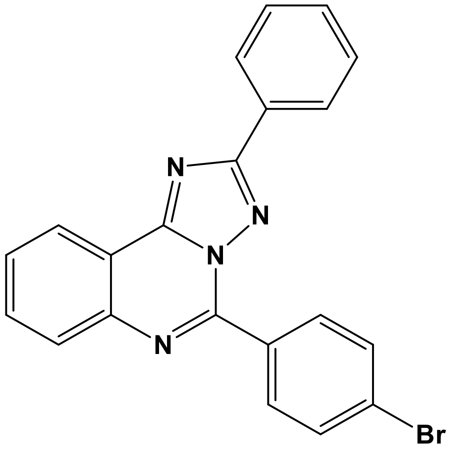

8a

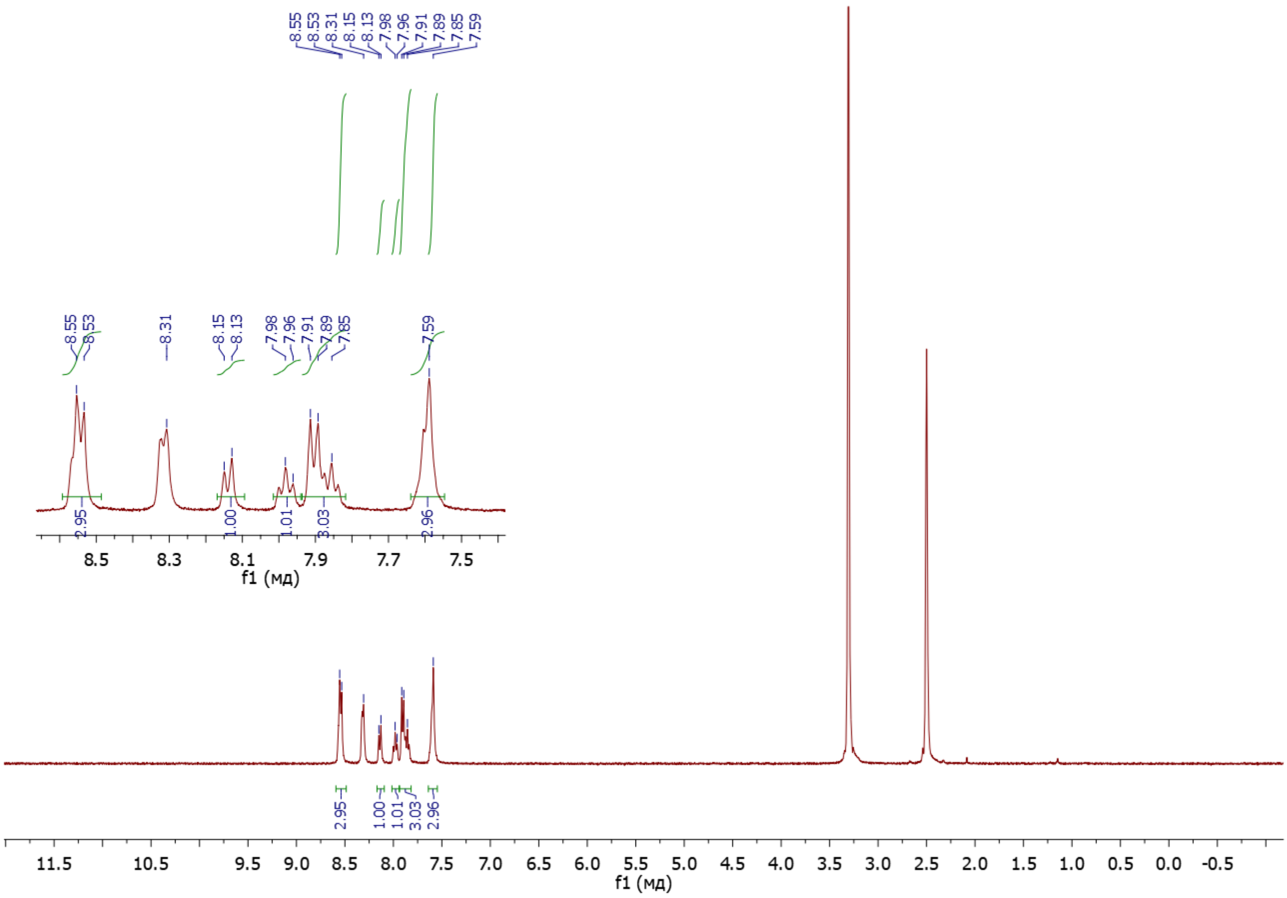
8.55
8.53
8.31
8.15
8.13
7.98
7.96
7.91
7.89
7.85
7.59
2.95
1.00
1.01
3.03
2.96
f1 (мд)

a

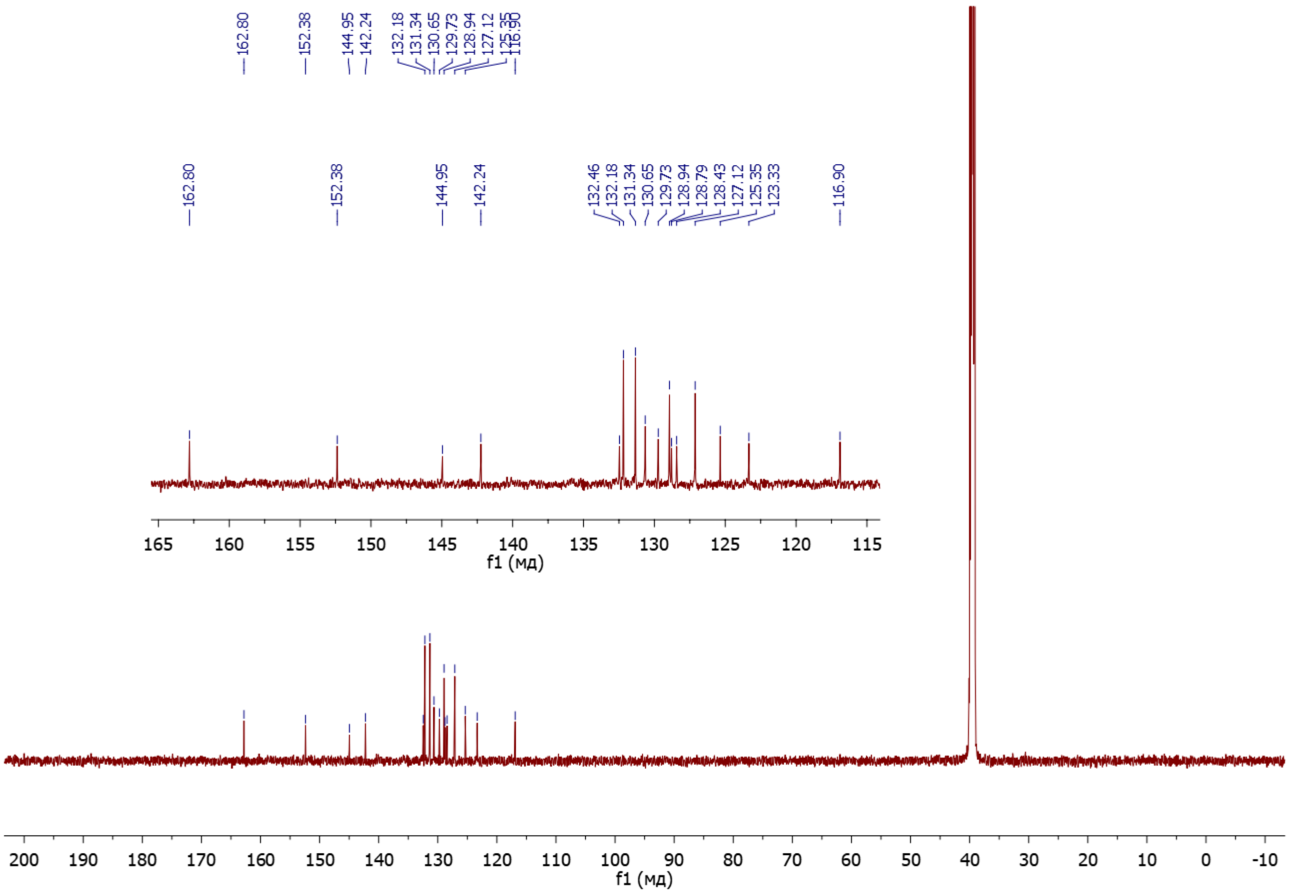

b

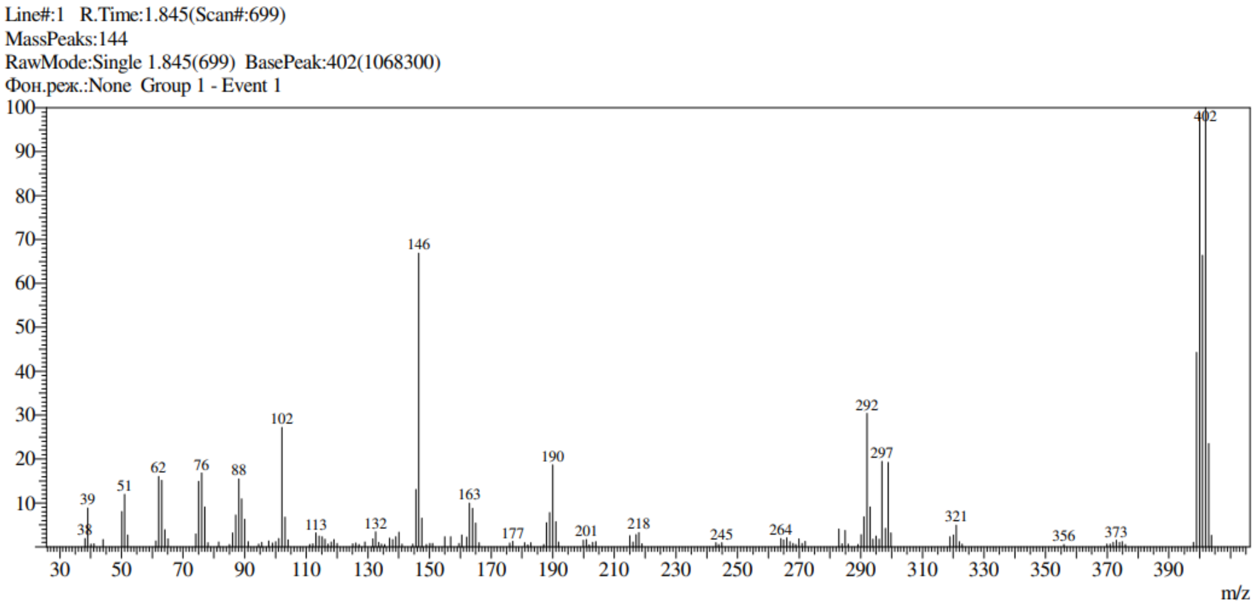

c

Figure S11. ^{1}H (a) ^{13}C (b) NMR and mass spectra (EI) (c) of **8a**.

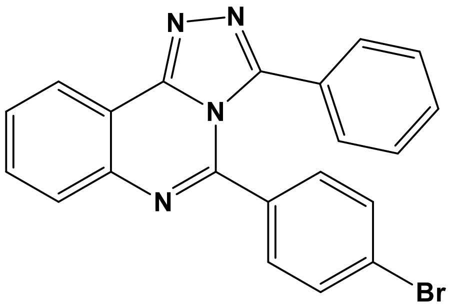

8b

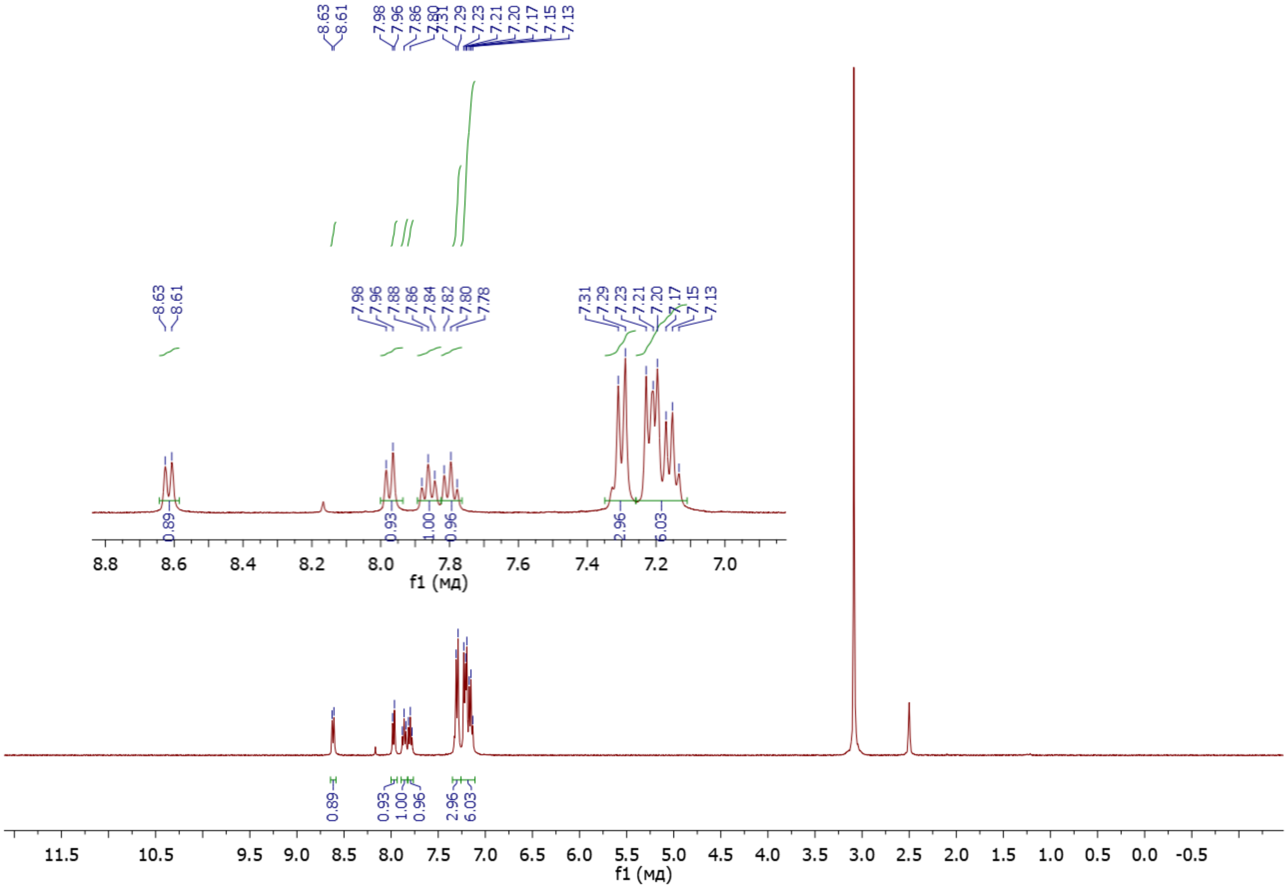

a

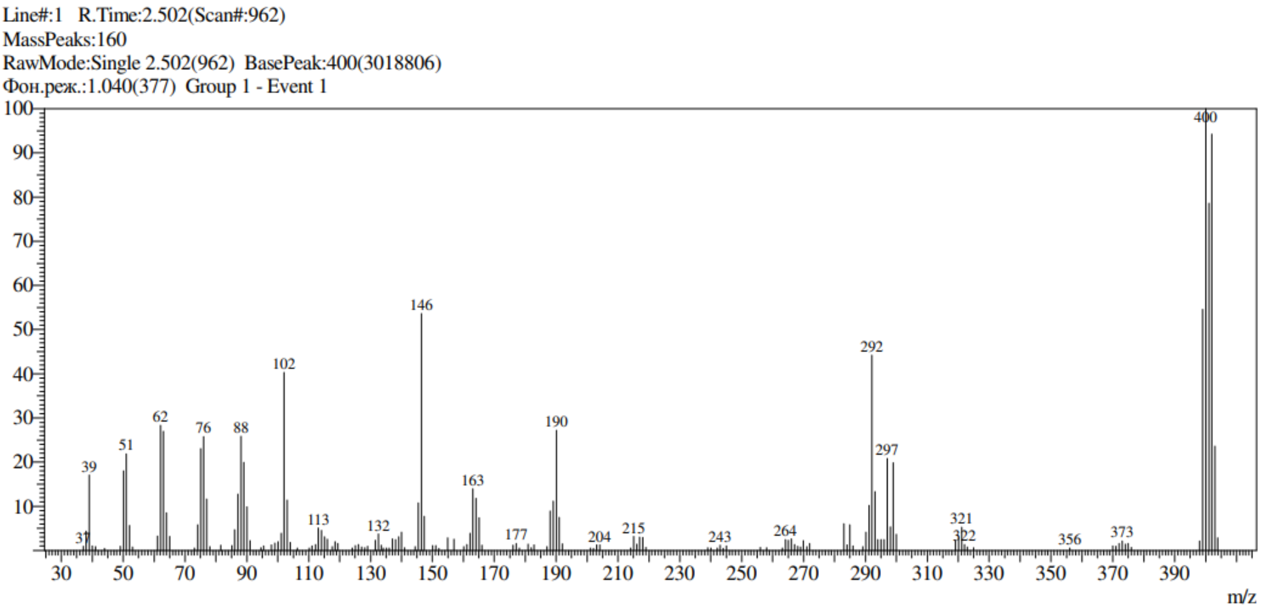

b

Figure S12. ^{1}H NMR (a) and mass spectra (EI) (b) of **8b**.

3. NMR and mass spectra of target products 6a-j, 10, 11 and 12a,b

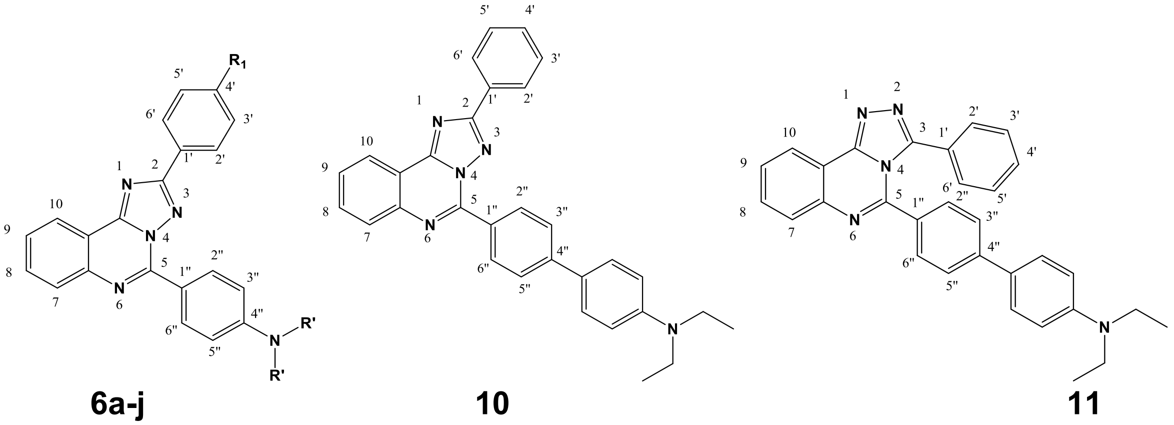

6a-j **10** **11**

Figure S13. Molecular structure of **6**, **10**, **11** and atoms numbering

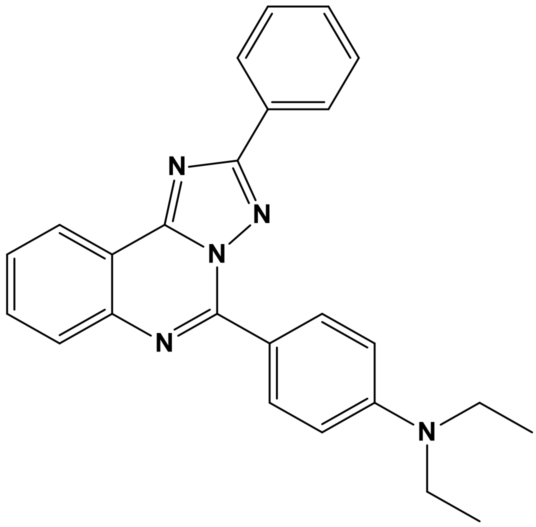

6a

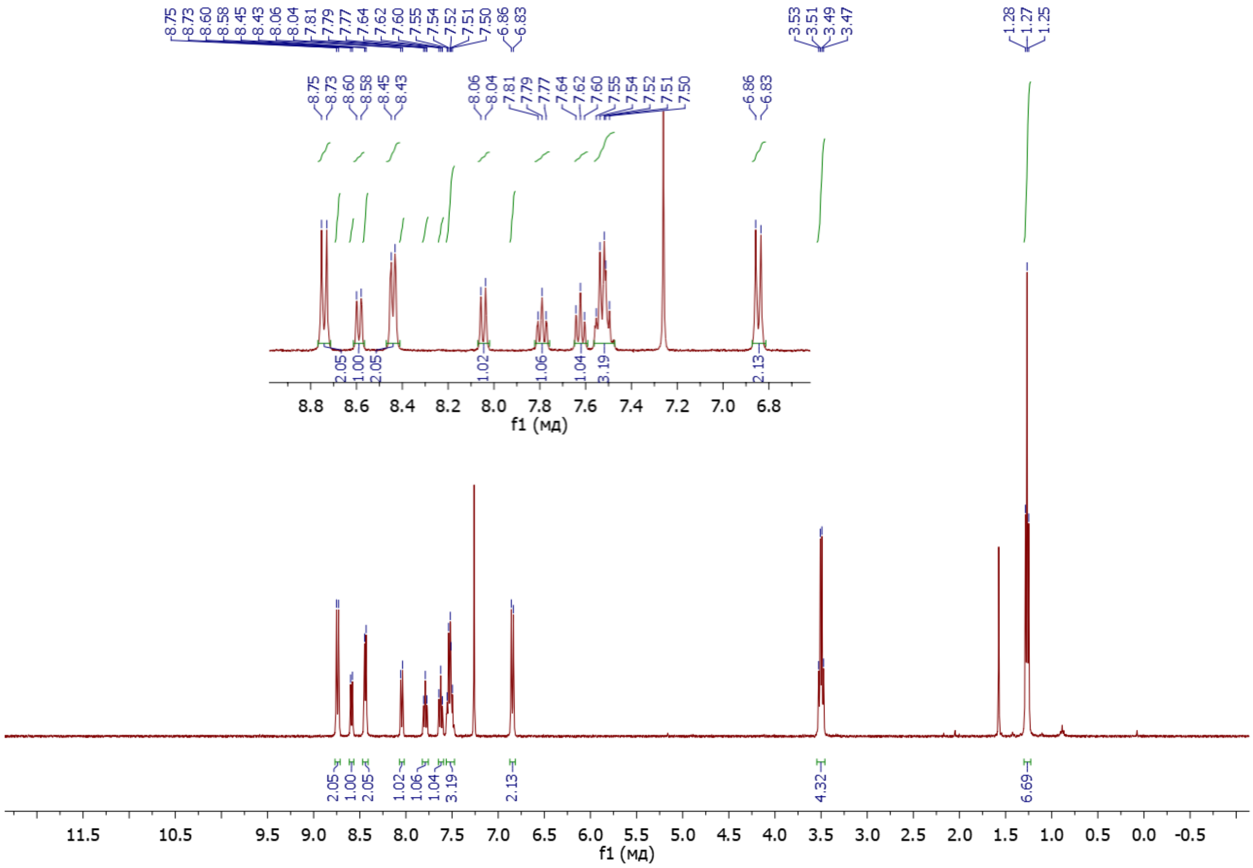
8.75 8.73 8.60 8.58 8.45 8.43 8.06 8.04 7.81 7.79 7.77 7.64 7.62 7.60 7.55 7.54 7.52 7.51 7.50 6.86 6.83
3.53 3.51 3.49 3.47
1.28 1.27 1.25
2.05 1.00 2.05 1.02 1.06 1.04 3.19 2.13
4.32
6.69
f1 (мд)

a

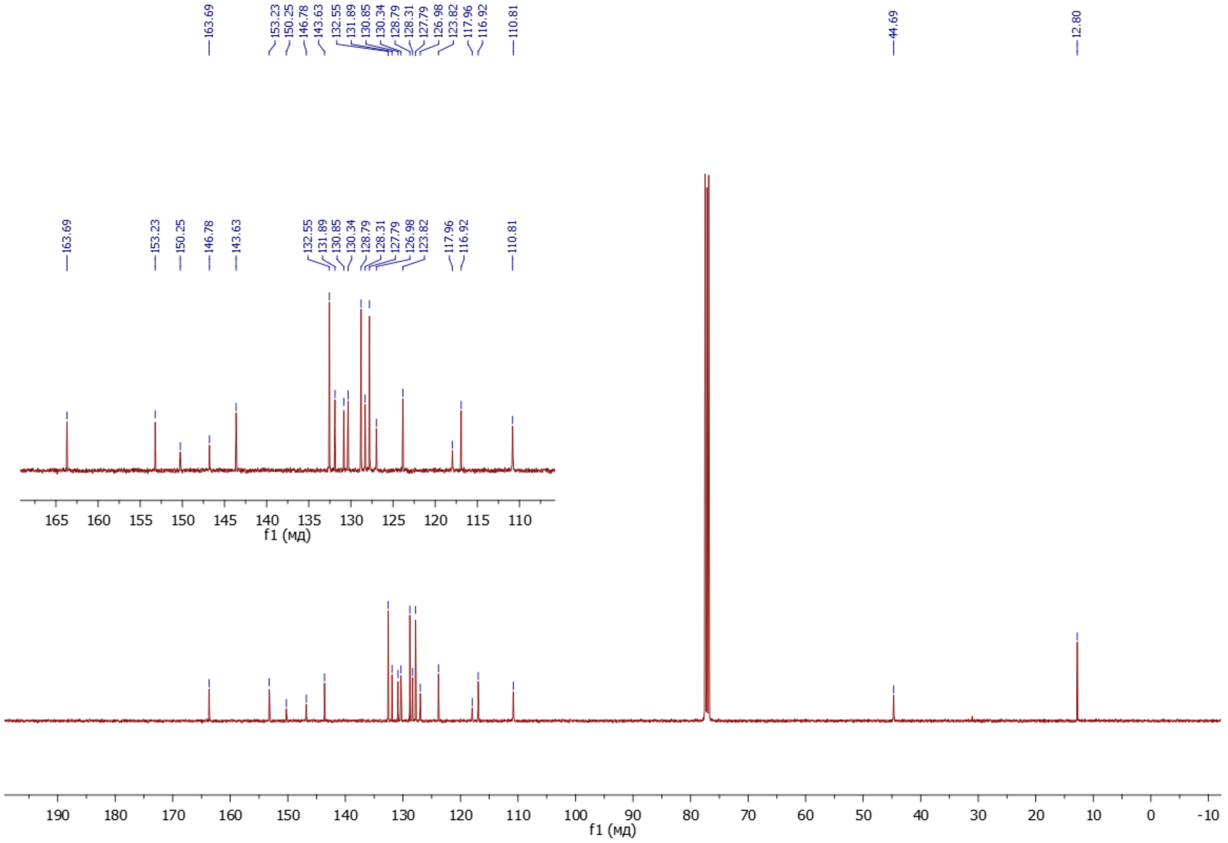
163.69
153.23 150.25 146.78 143.63 132.55 131.89 130.85 130.34 128.79 128.31 127.79 126.98 123.82 117.96 116.92 110.81
44.69
12.80
f1 (мд)

b

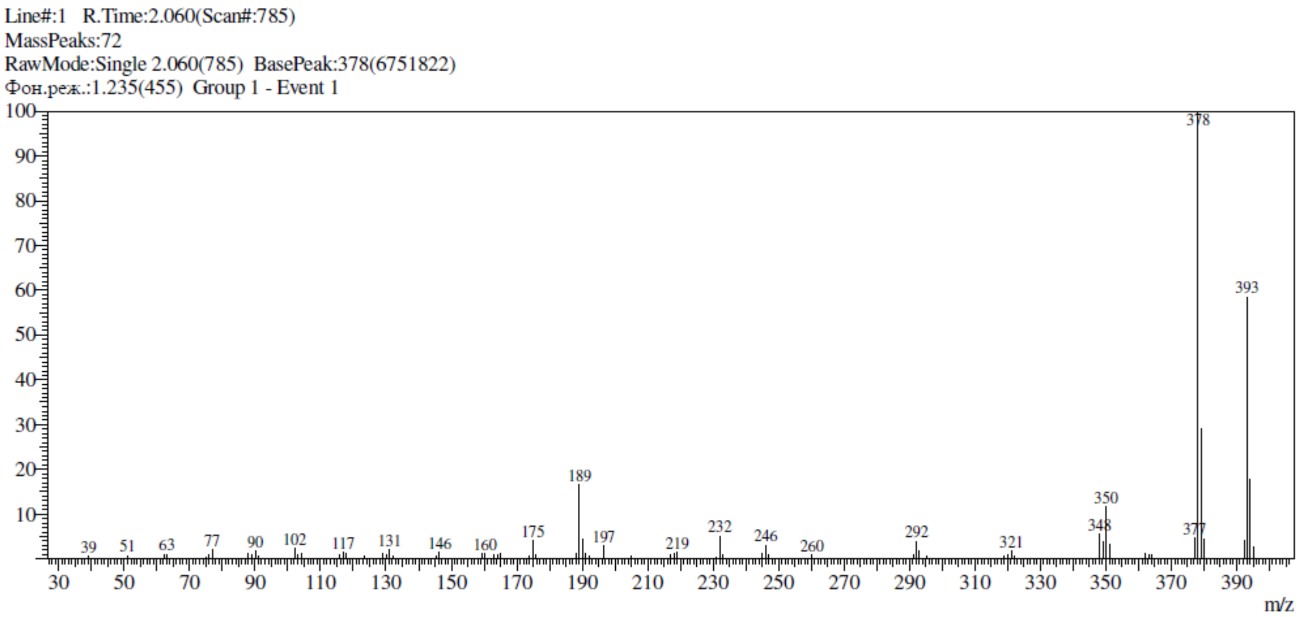

c

Figure S14. ^{1}H NMR (a), ^{13}C NMR (b) and mass (EI) (c) spectra of **6a**.

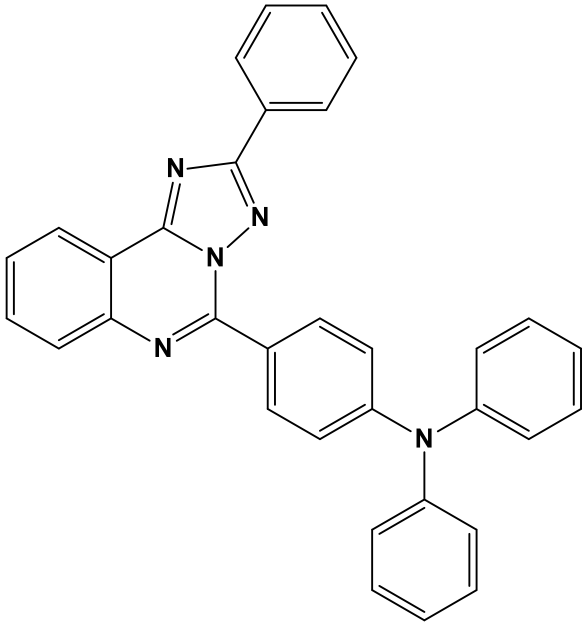

6b

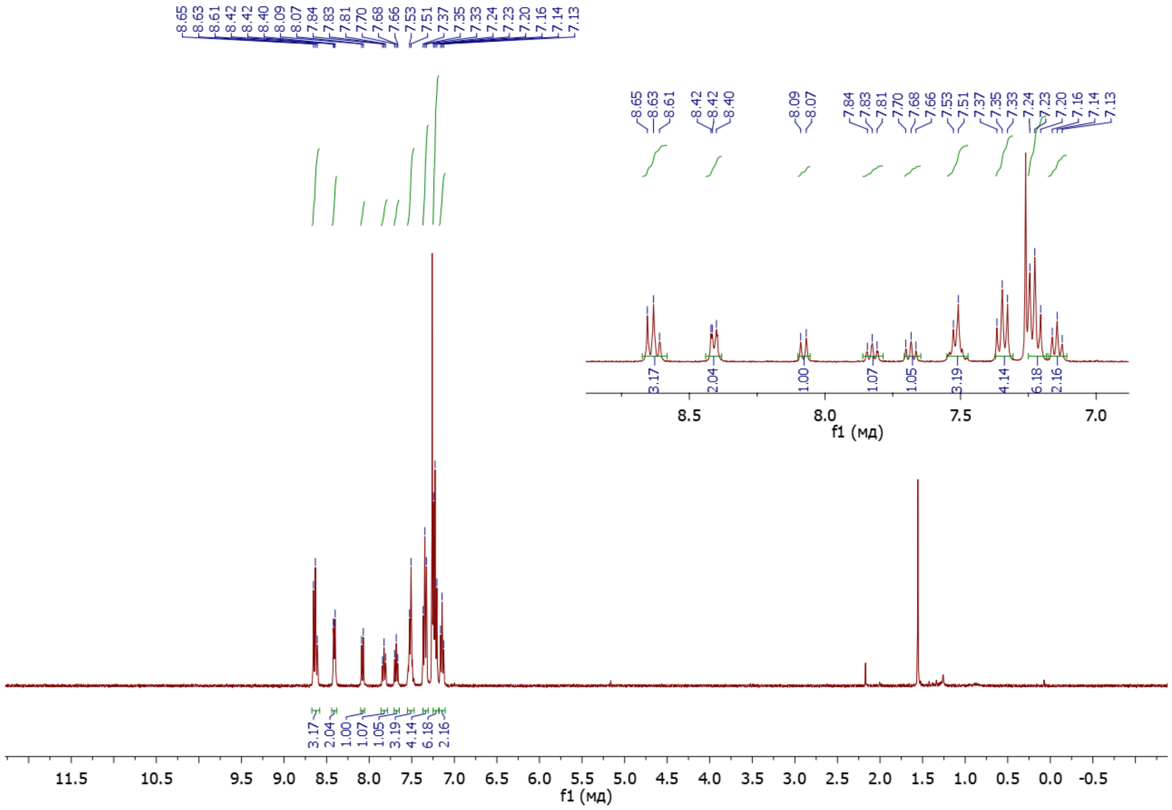

a

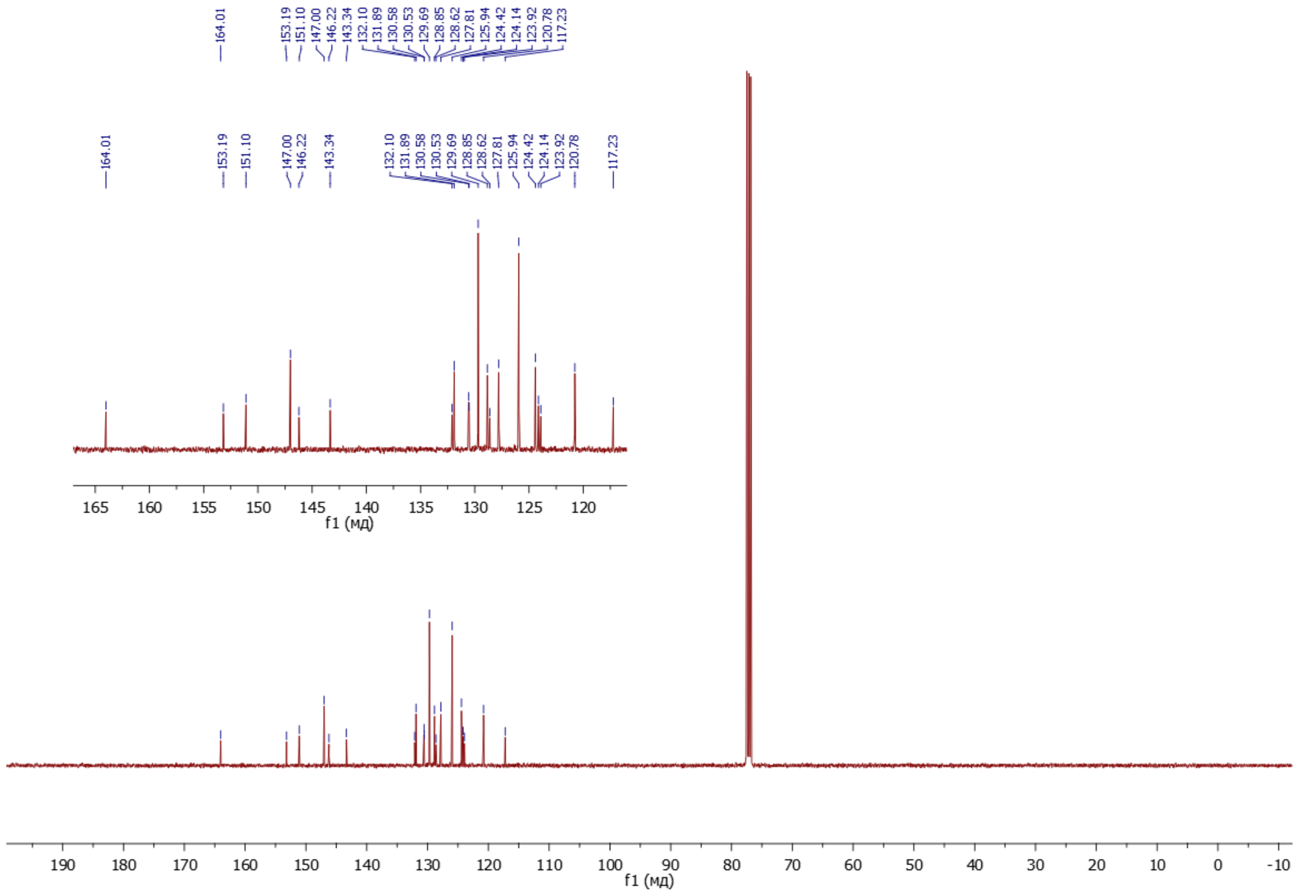

b

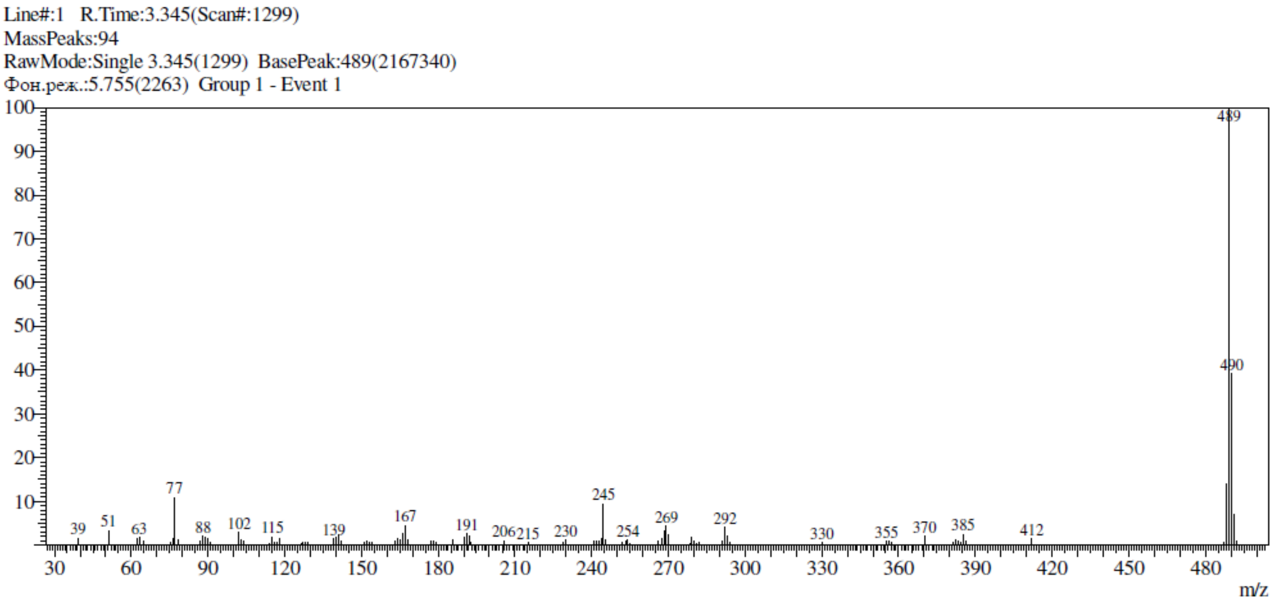

c

Figure S15. ^{1}H NMR (a), ^{13}C NMR (b) and mass (EI) (c) spectra of **6b**.

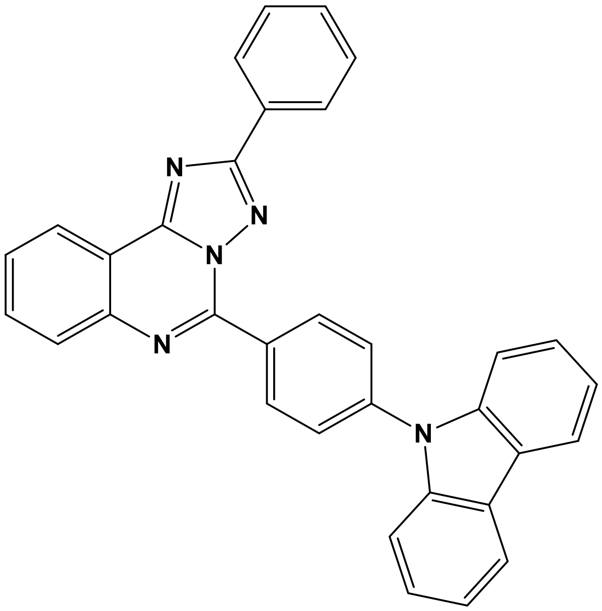

6c

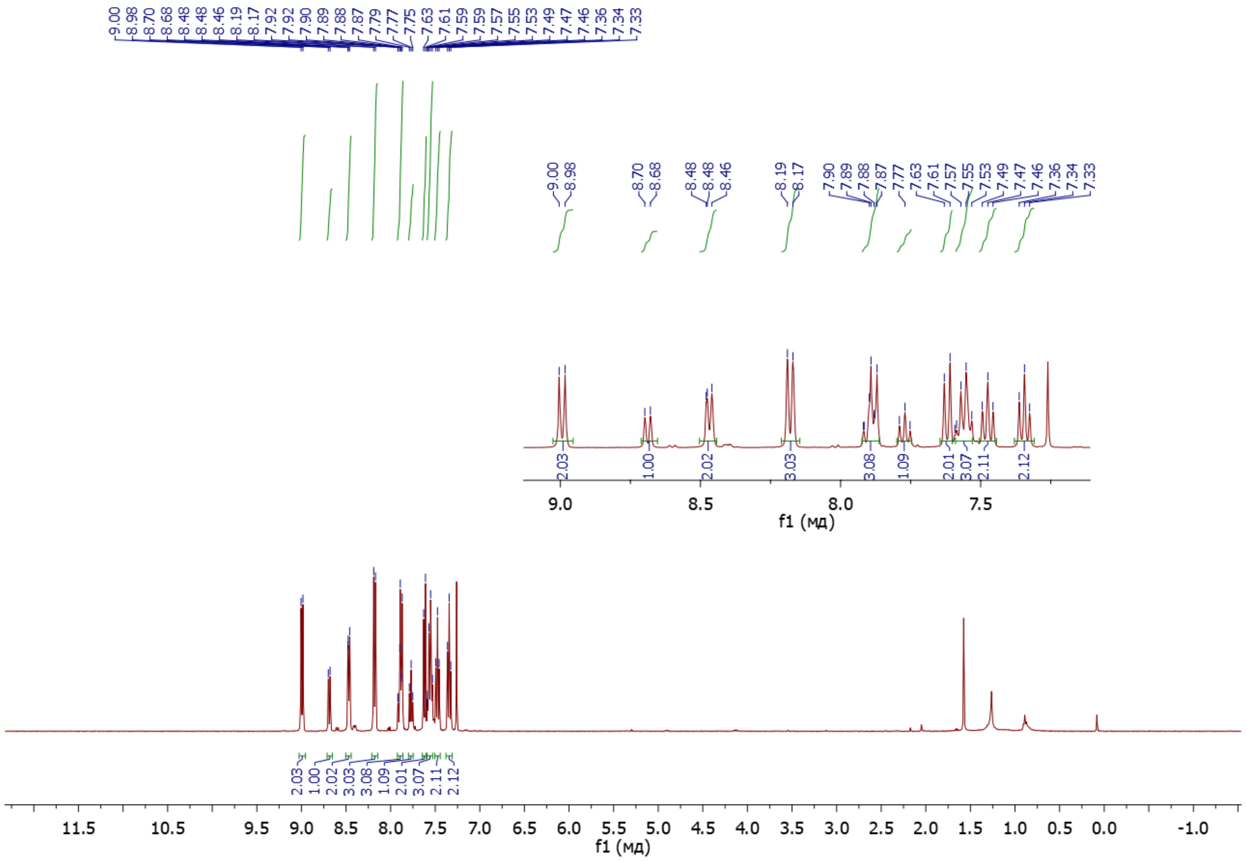

a

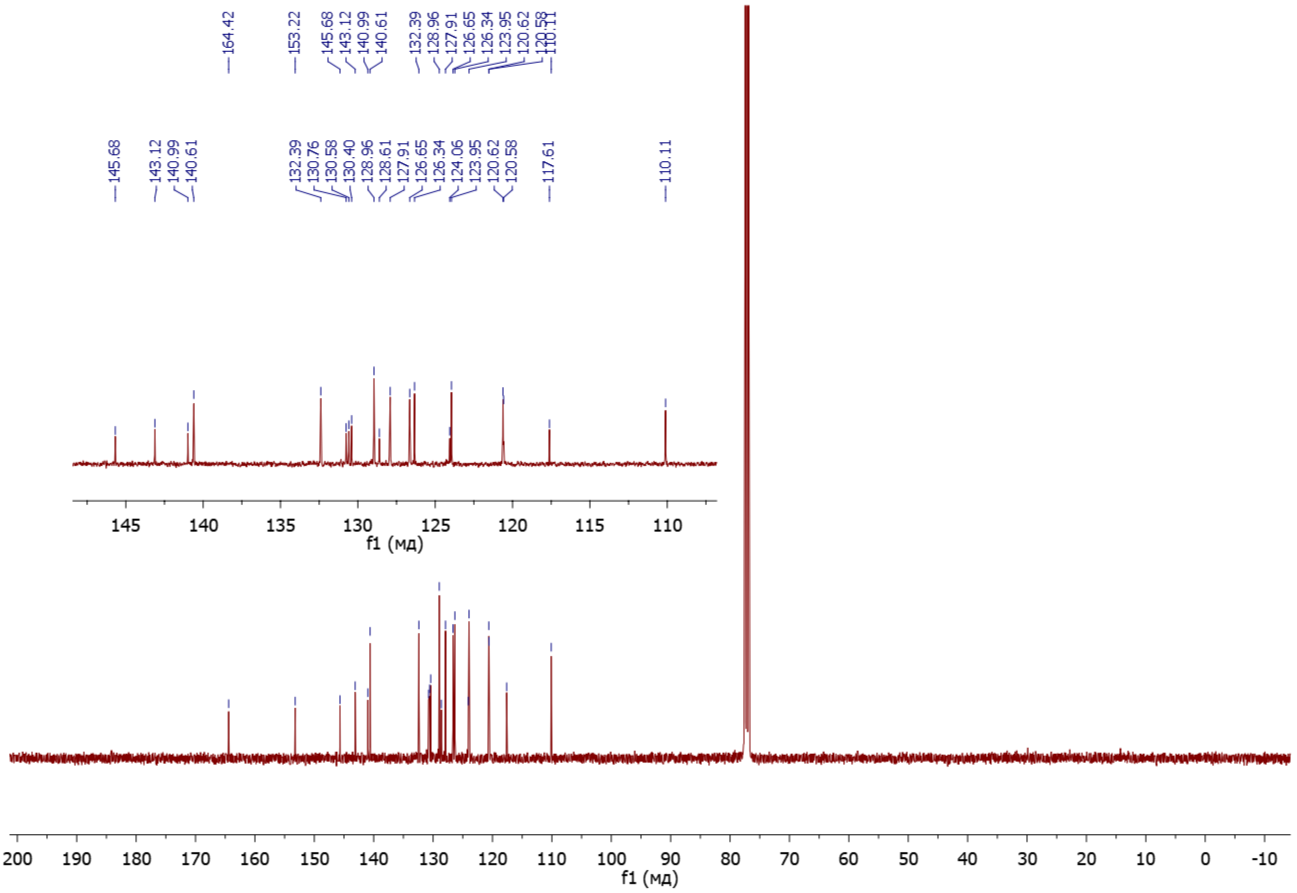

b

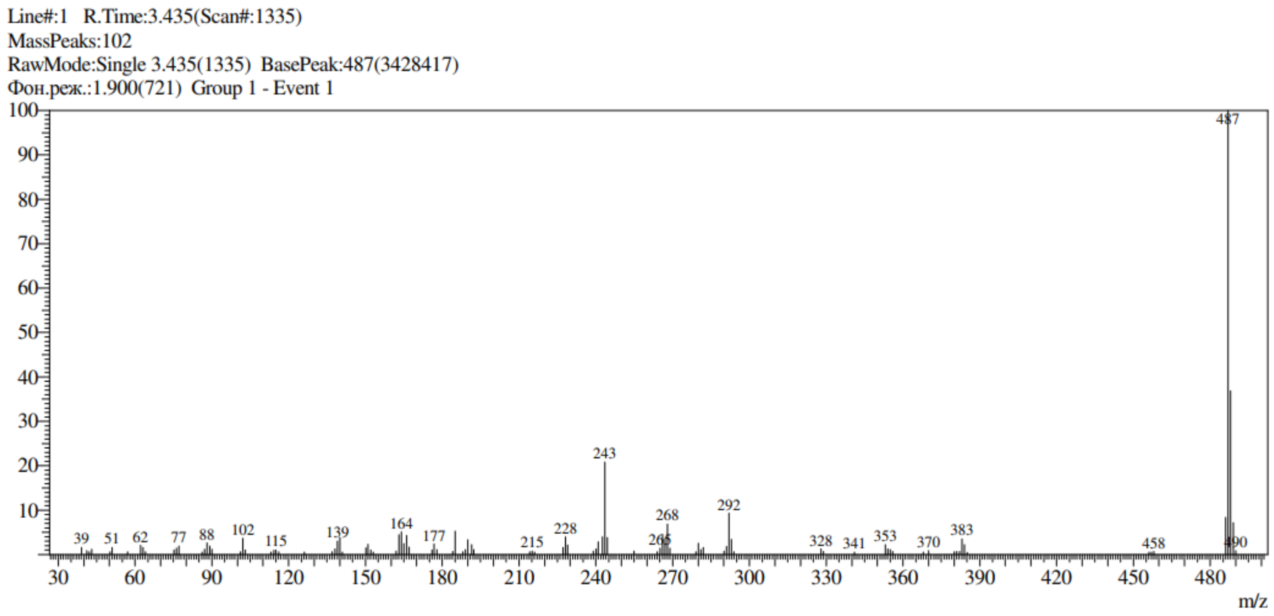

c

Figure S16. ^{1}H NMR (a), ^{13}C NMR (b) and mass (EI) (c) spectra of **6c**.

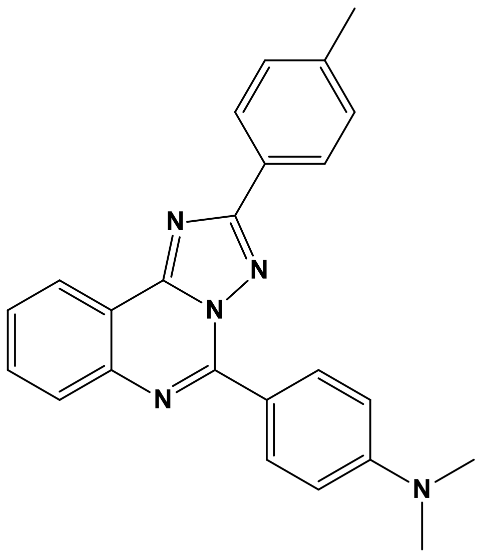

6d

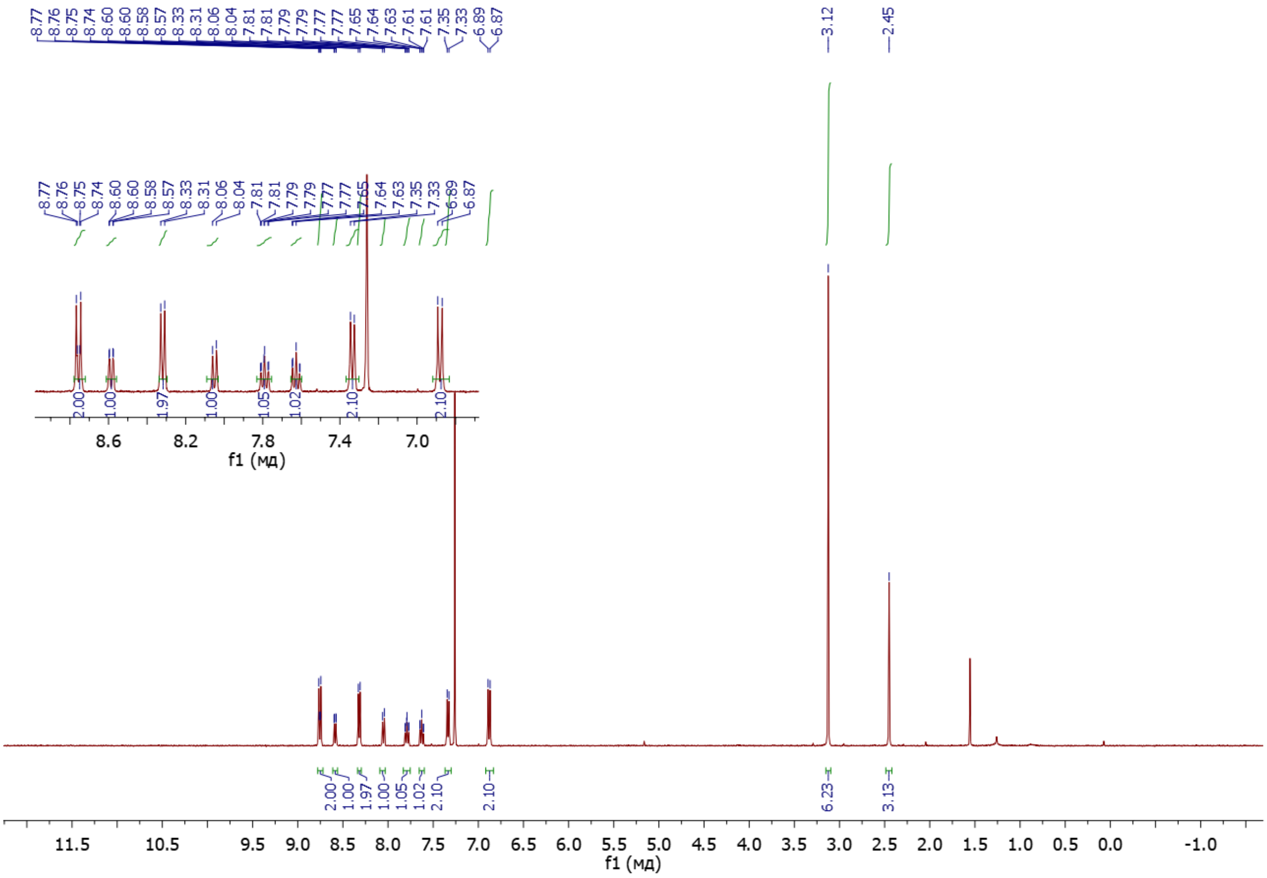
8.77
8.76
8.75
8.74
8.60
8.60
8.58
8.57
8.33
8.31
8.06
8.04
7.81
7.81
7.79
7.79
7.77
7.77
7.65
7.64
7.63
7.61
7.61
7.35
7.33
6.89
6.87
3.12
2.45
2.00
1.00
1.97
1.00
1.05
1.02
2.10
2.10
6.23
3.13
f1 (мд)

a

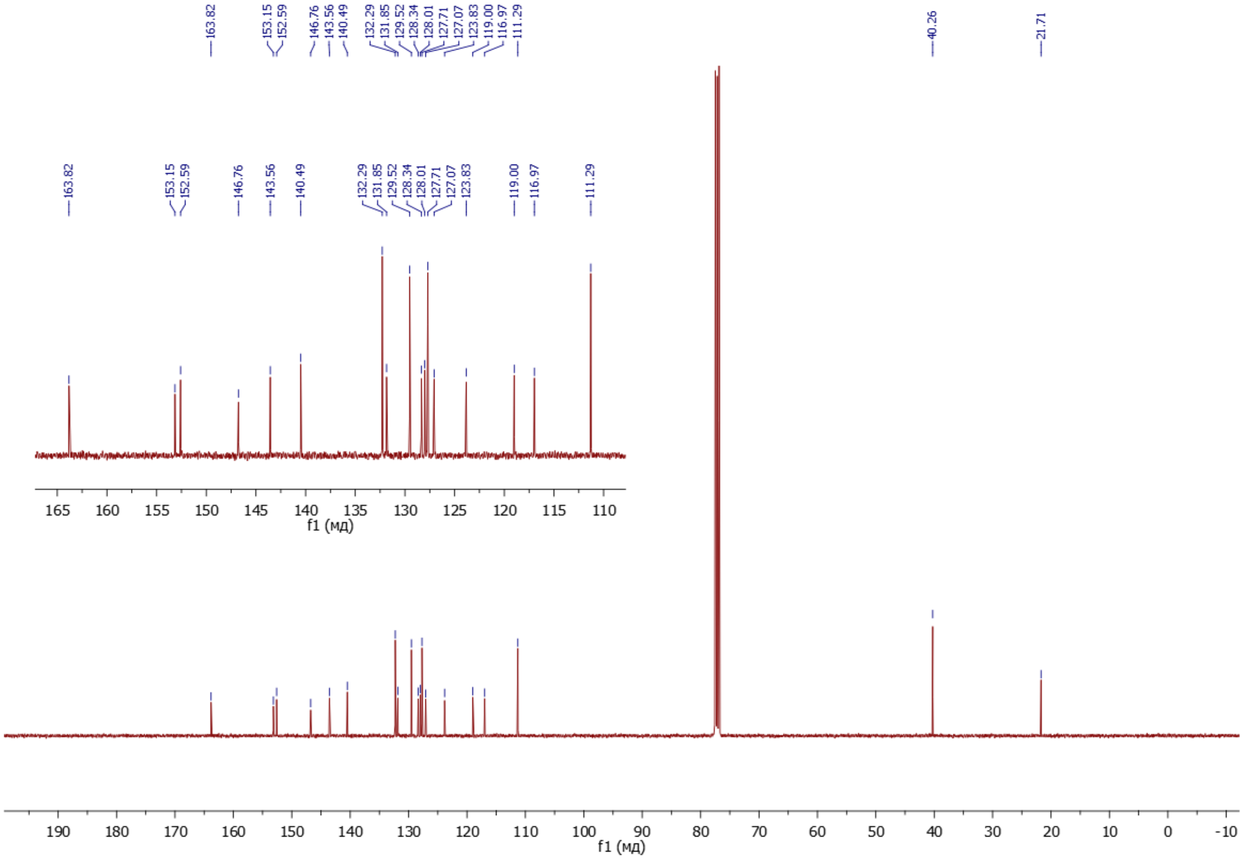
163.82
153.15
152.59
146.76
143.56
140.49
132.29
131.85
129.52
128.34
128.01
127.71
127.07
123.83
119.00
116.97
111.29
40.26
21.71
f1 (мд)

b

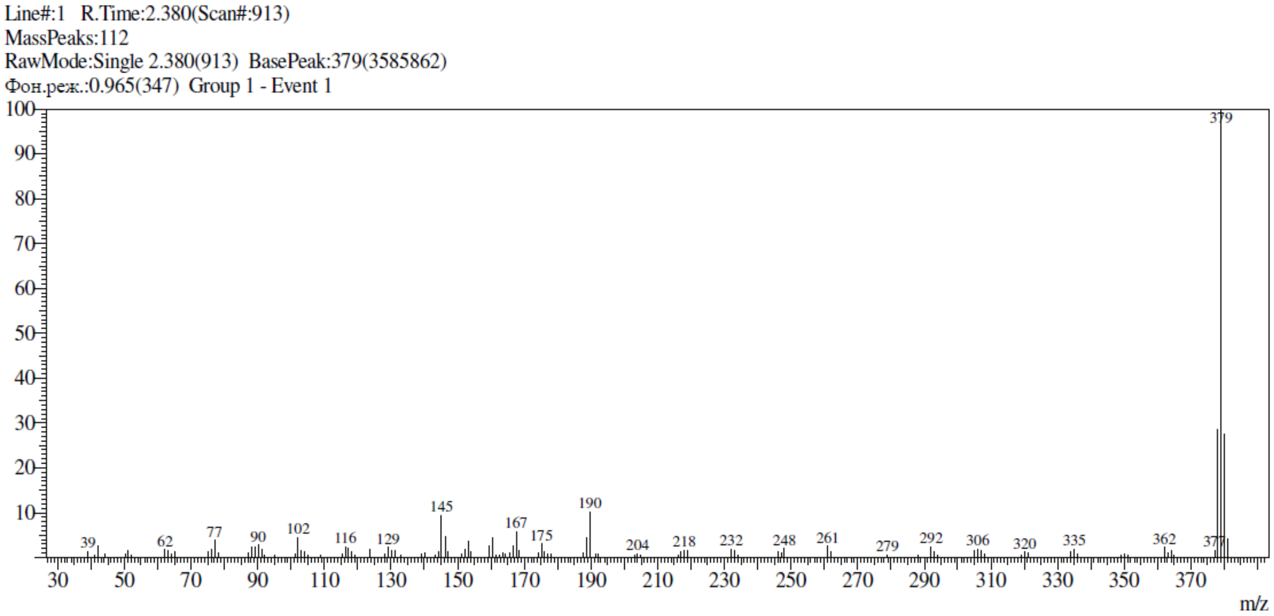

c

Figure S17. ^{1}H NMR (a), ^{13}C NMR (b) and mass (EI) (c) spectra of **6d**.

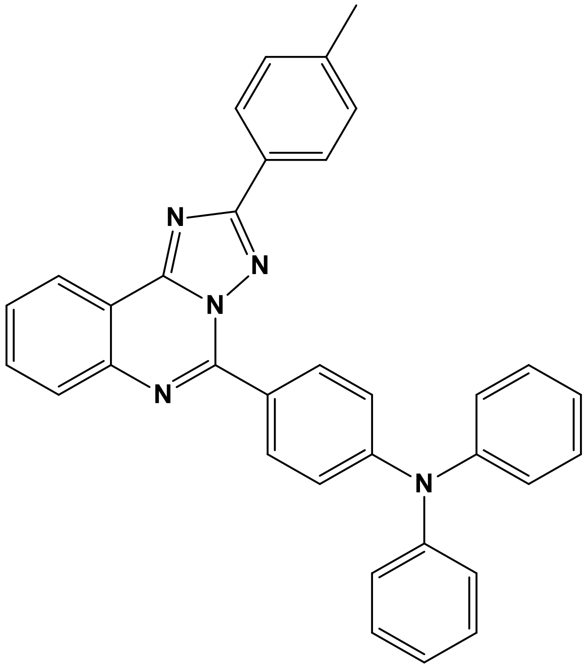

6e

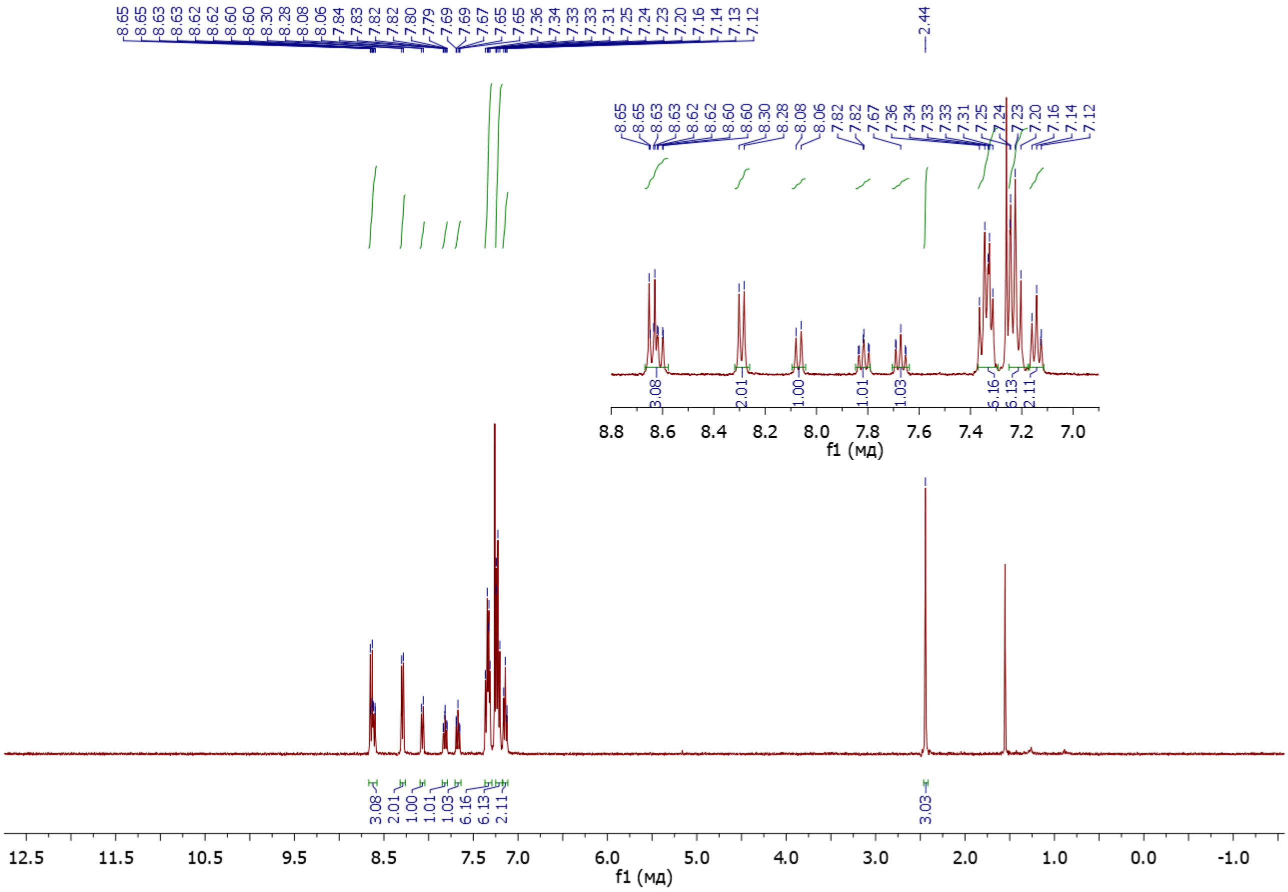
8.65 8.65 8.63 8.63 8.62 8.62 8.60 8.60 8.30 8.28 8.08 8.06 7.84 7.83 7.82 7.82 7.80 7.79 7.69 7.69 7.67 7.65 7.65 7.36 7.34 7.33 7.33 7.31 7.25 7.24 7.23 7.20 7.16 7.14 7.13 7.12
2.44
8.65 8.65 8.63 8.63 8.62 8.62 8.60 8.60 8.30 8.28 8.08 8.06 7.82 7.82 7.67 7.36 7.34 7.33 7.33 7.31 7.25 7.24 7.23 7.20 7.16 7.14 7.12
3.08 2.01 1.00 1.01 1.03 6.16 6.13 2.11
8.8 8.6 8.4 8.2 8.0 7.8 7.6 7.4 7.2 7.0
f1 (мд)
3.08 2.01 1.00 1.01 1.03 6.16 6.13 2.11 3.03
12.5 11.5 10.5 9.5 8.5 7.5 7.0 6.0 5.0 4.0 3.0 2.0 1.0 0.0 -1.0
f1 (мд)

a

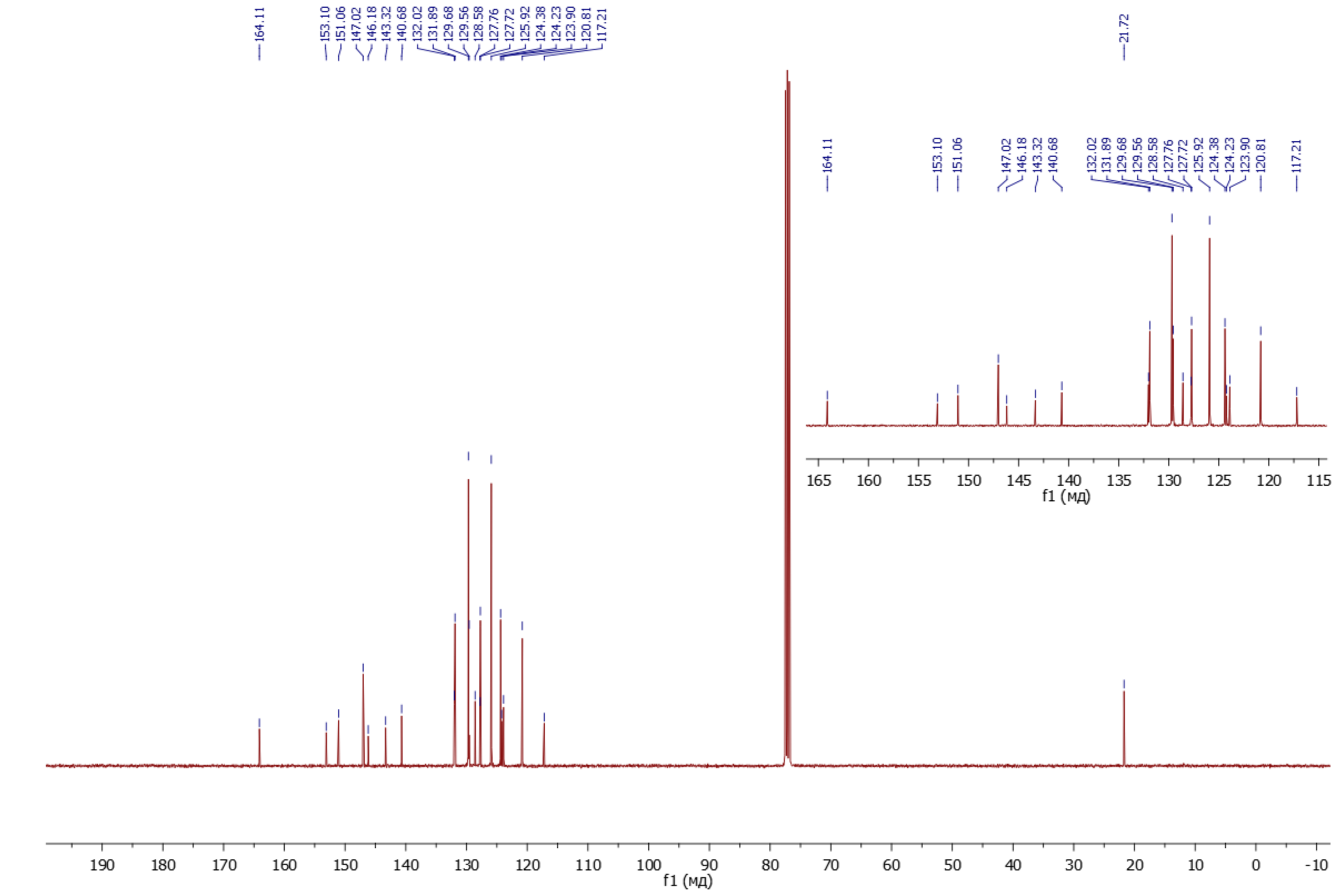

b

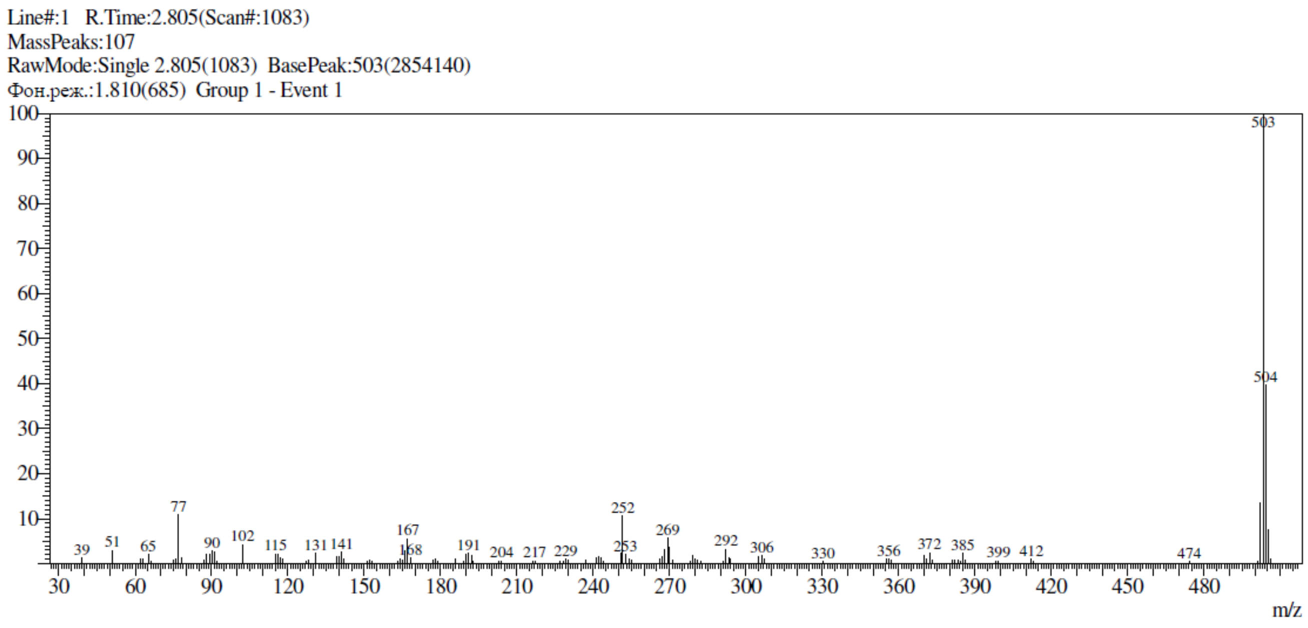

c

Figure S18. ^{1}H NMR (a), ^{13}C NMR (b) and mass (EI) (c) spectra of **6e**.

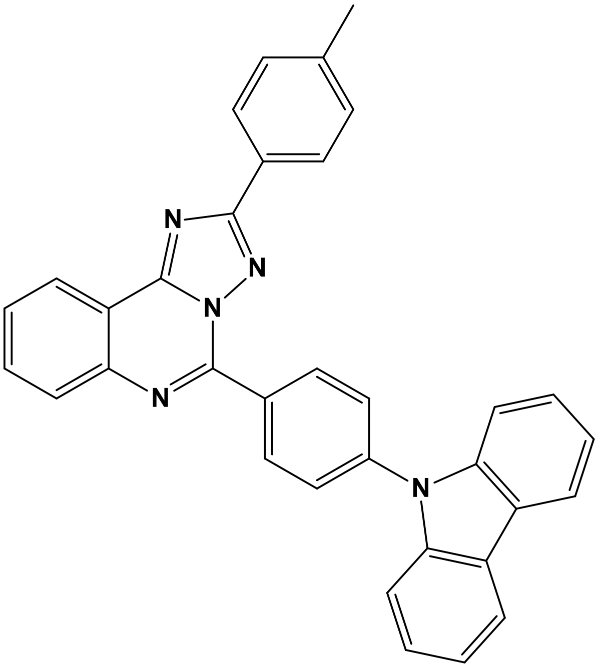

6f

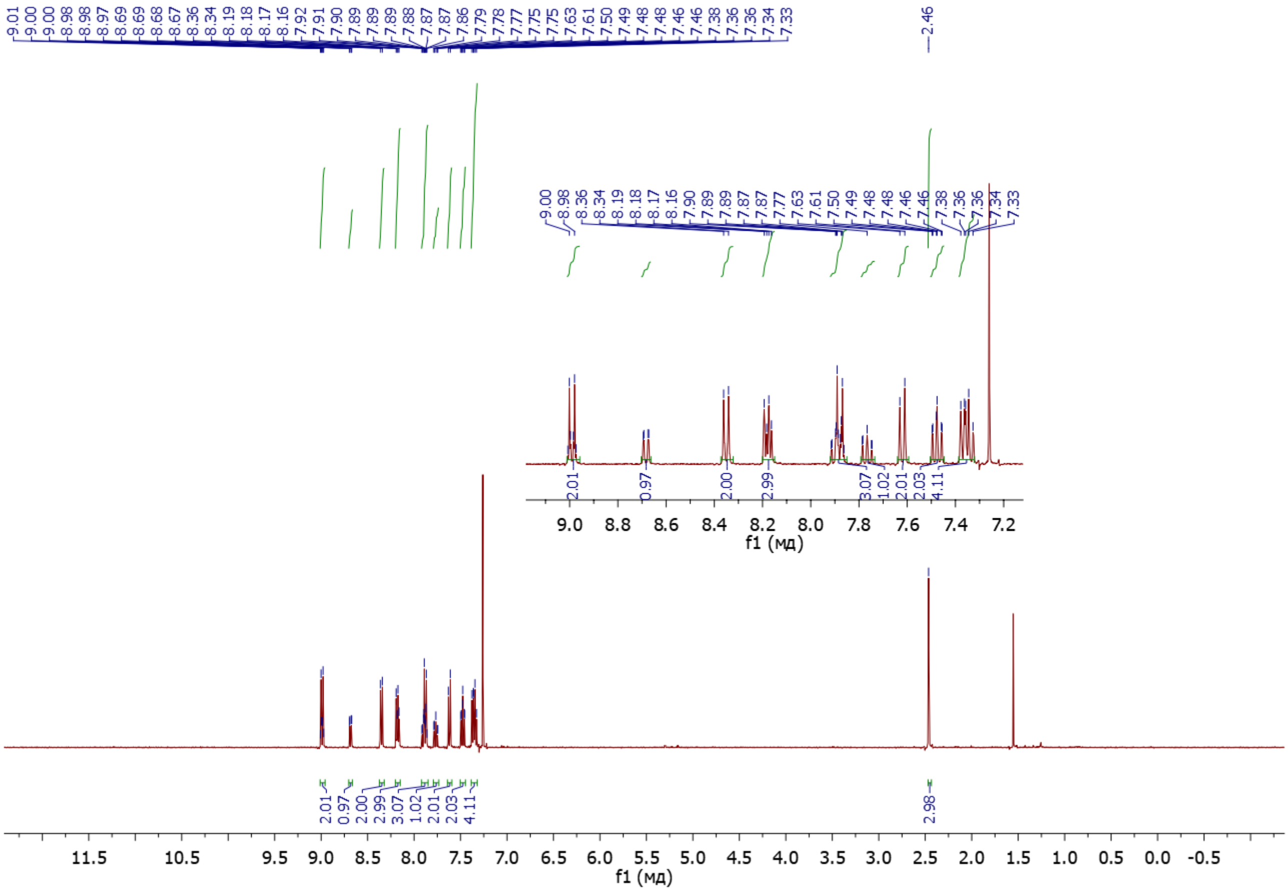

2.46
f1 (мд)
11.5 10.5 9.5 9.0 8.5 8.0 7.5 7.0 6.5 6.0 5.5 5.0 4.5 4.0 3.5 3.0 2.5 2.0 1.5 1.0 0.5 0.0 -0.5
9.0 8.8 8.6 8.4 8.2 8.0 7.8 7.6 7.4 7.2
2.01 0.97 2.00 2.99 3.07 1.02 2.01 2.03 4.11
2.98

a

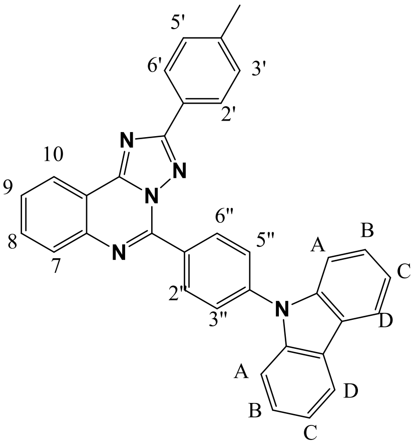

5'
6'
3'
2'
10
9
8
7
N
N
N
N
6''
5''
2''
3''
A
B
C
D

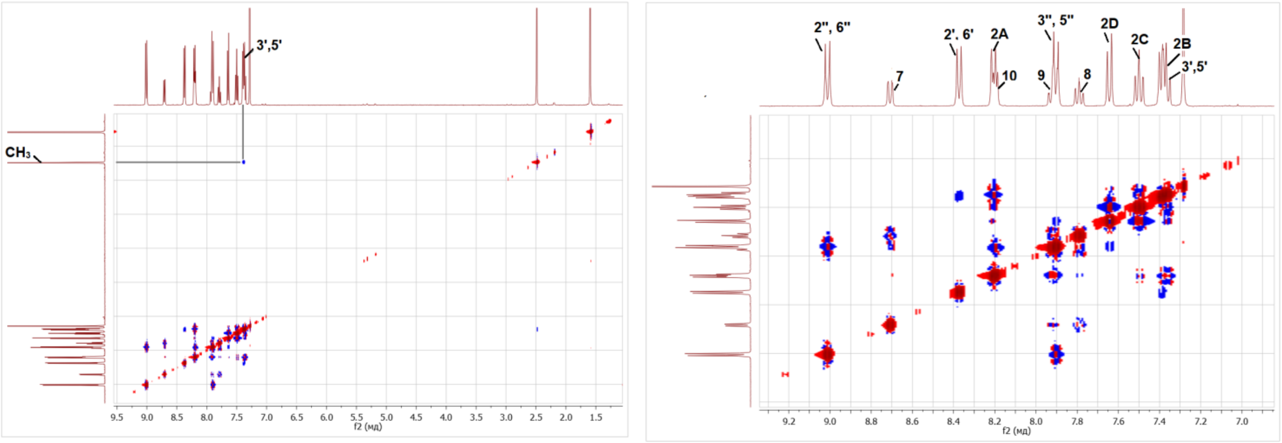

3',5'
CH3
2'', 6''
2', 6'
2A
3'', 5''
2D
2C
2B
7
10
9
8
f2 (мд)

b

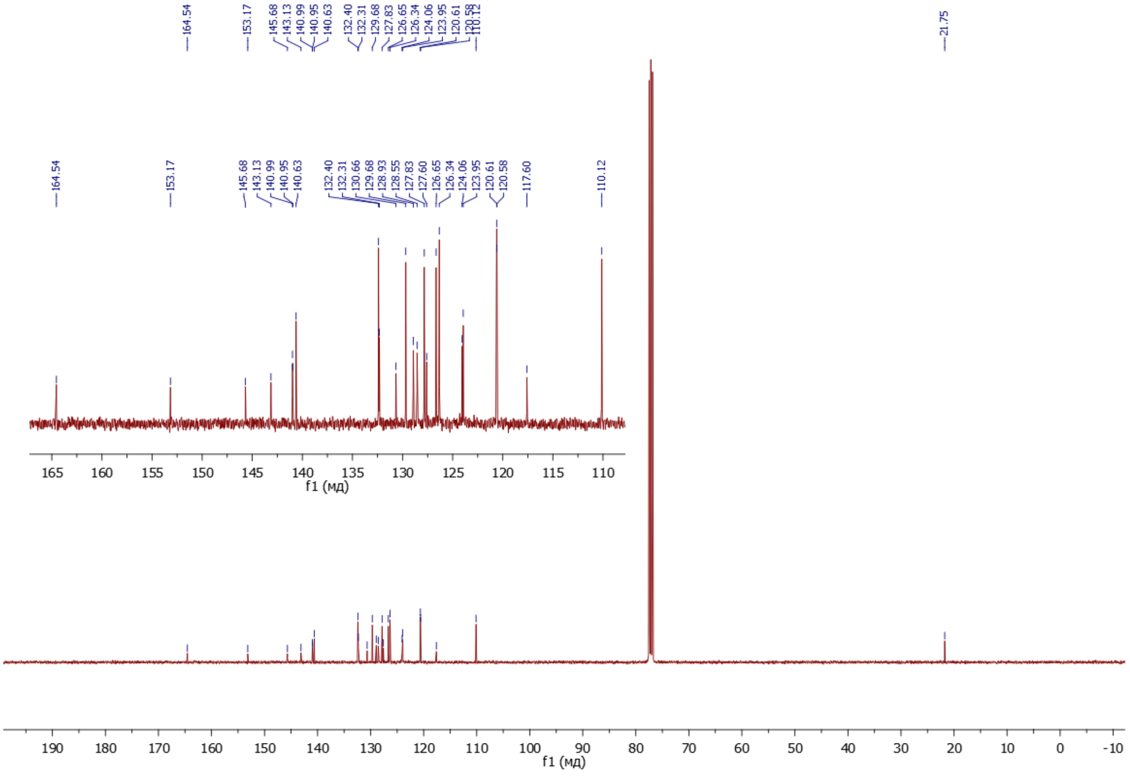

164.54
153.17
145.68
143.13
140.99
140.95
140.63
132.40
132.31
130.66
129.68
128.93
128.55
127.83
127.60
126.65
126.34
124.06
123.95
120.61
120.58
117.60
110.12
21.75
f1 (мд)

c

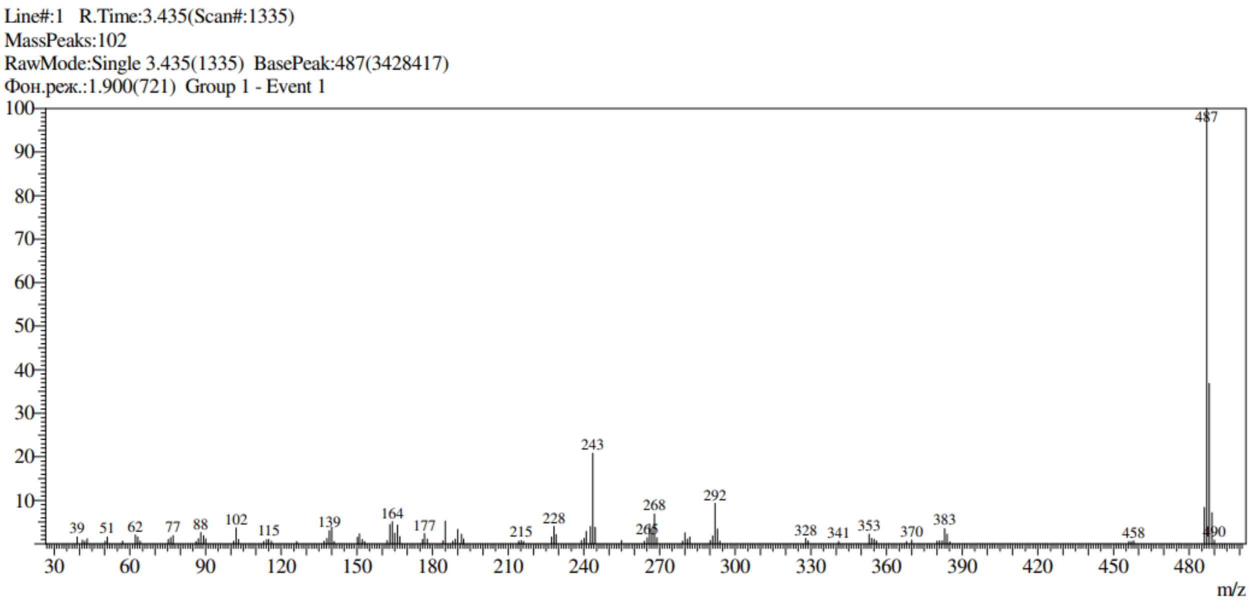

d

Figure S19. ^{1}H NMR spectrum (a), ^{1}H-^{1}H NOESY correlation spectrum (b), ^{13}C NMR spectrum (c) and mass spectrum (EI) (d) spectra of compound **6f**.

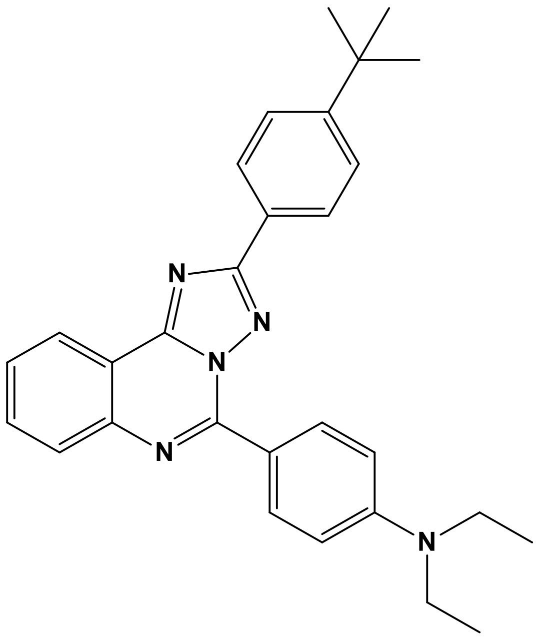

6g

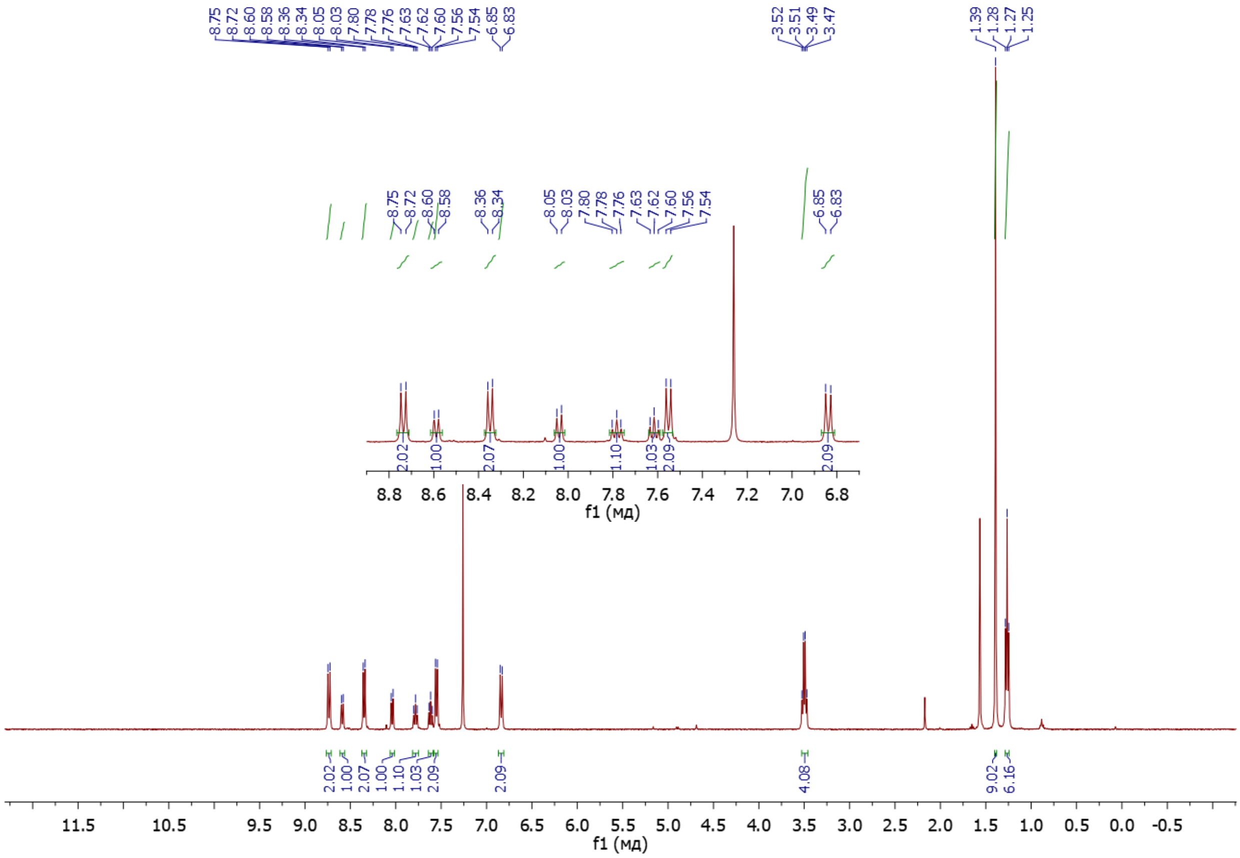
8.75 8.72 8.60 8.58 8.36 8.34 8.05 8.03 7.80 7.78 7.76 7.63 7.62 7.60 7.56 7.54 6.85 6.83
3.52 3.51 3.49 3.47
1.39 1.28 1.27 1.25
2.02 1.00 2.07 1.00 1.10 1.03 2.09 2.09
4.08
9.02 6.16
f1 (мд)

a

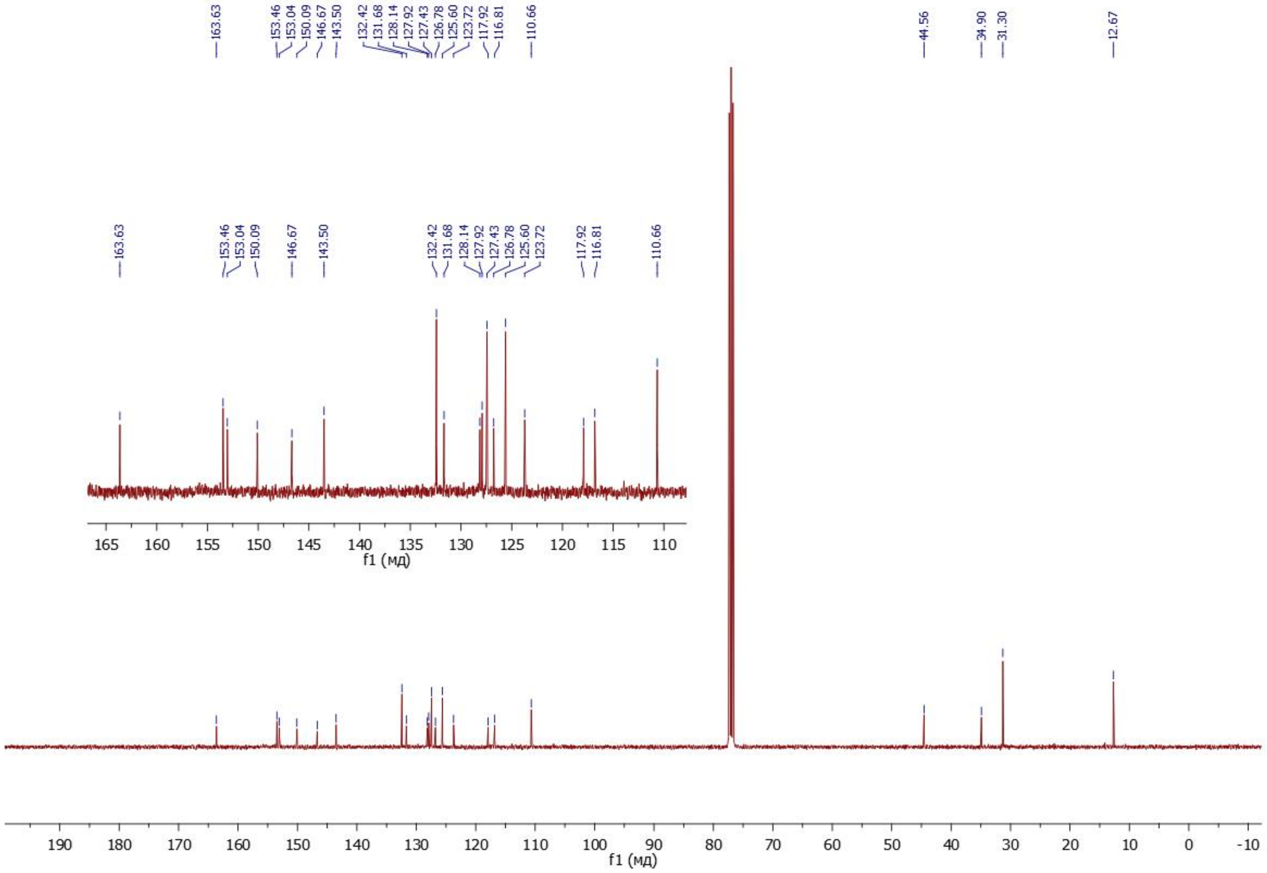
163.63 153.46 153.04 150.09 146.67 143.50 132.42 131.68 128.14 127.92 127.43 126.78 125.60 123.72 117.92 116.81 110.66
44.56 34.90 31.30 12.67
f1 (мд)

b

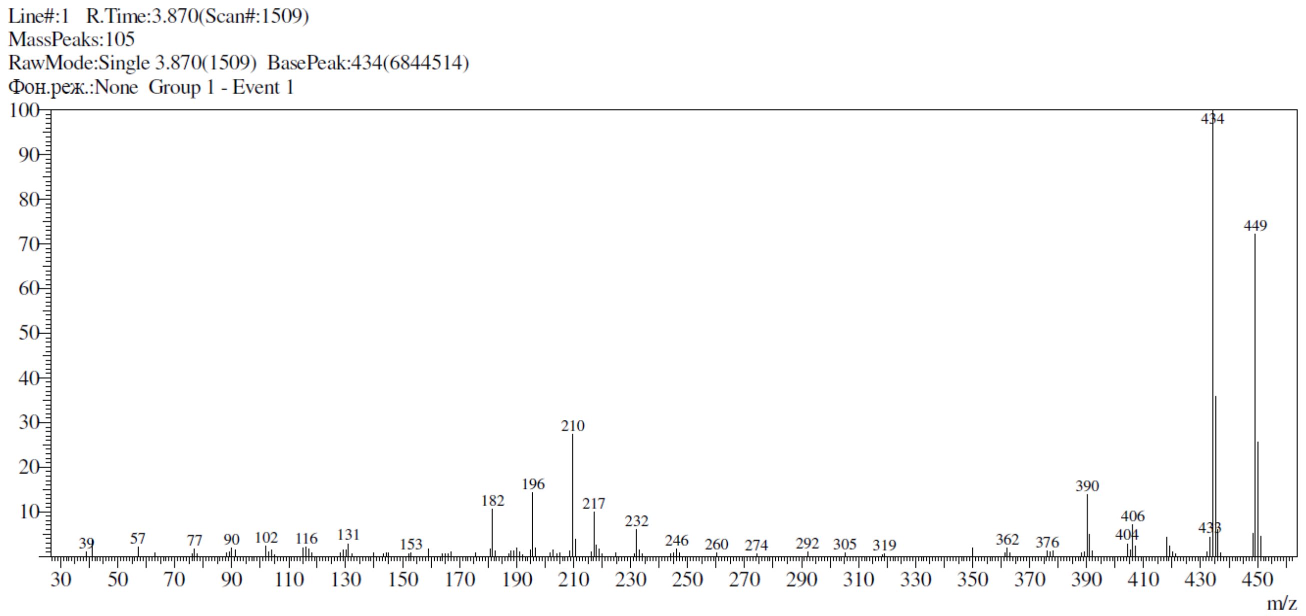

c

Figure S20. ^{1}H NMR (a), ^{13}C NMR (b) and mass (EI) (c) spectra of **6g**.

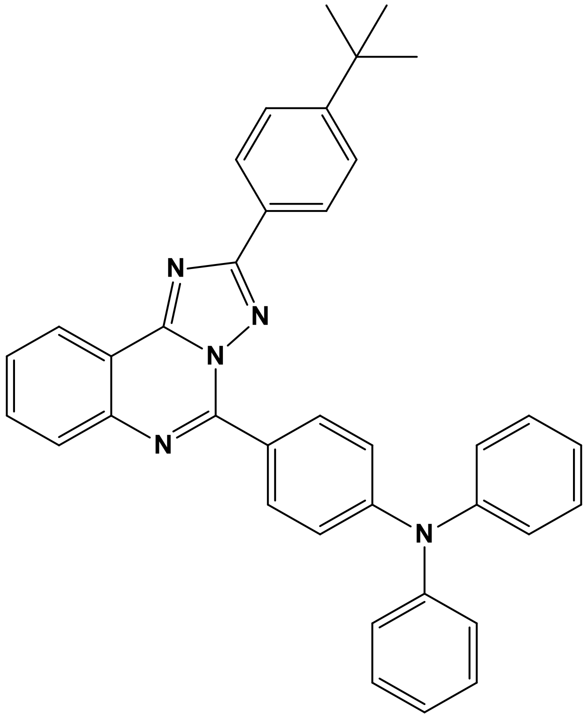

6h

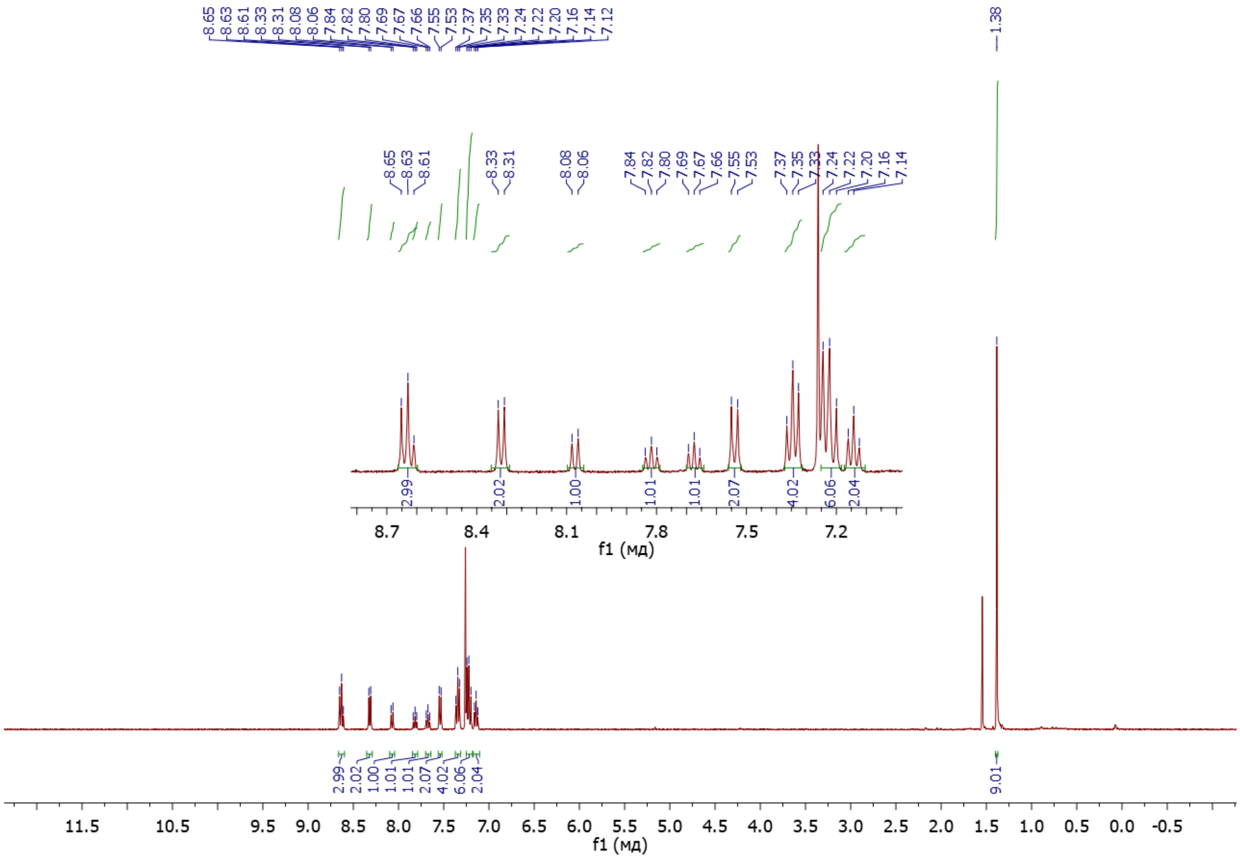

a

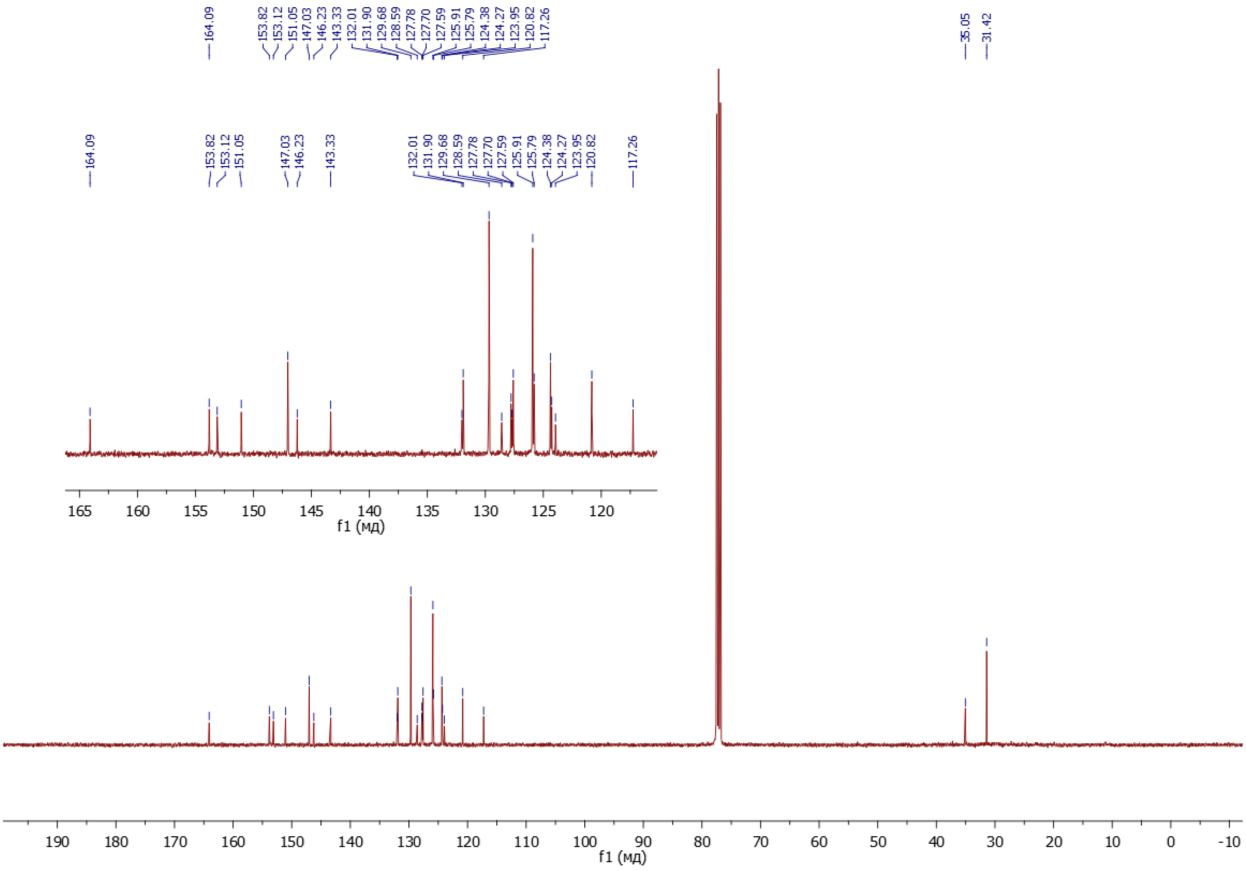

b

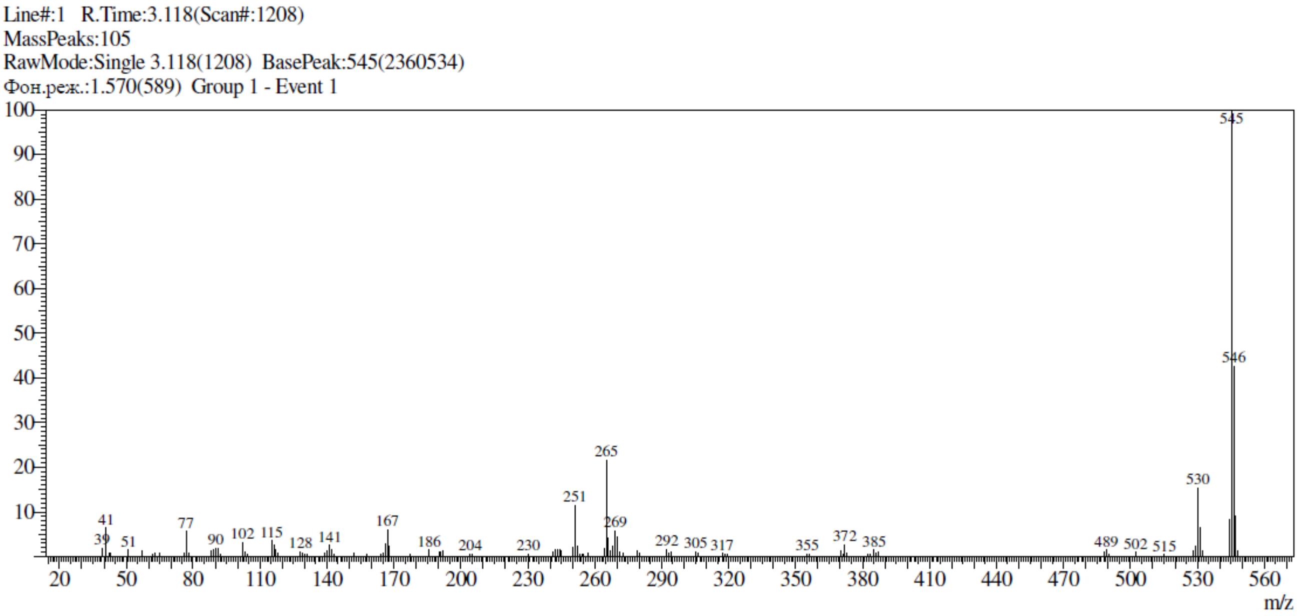

c

Figure S21. ^{1}H NMR (a), ^{13}C NMR (b) and mass (EI) (c) spectra of **6i**.

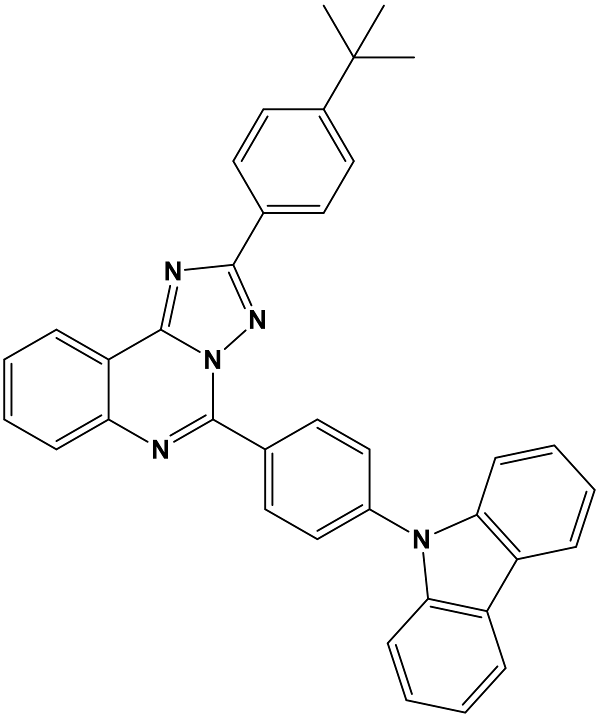

6i

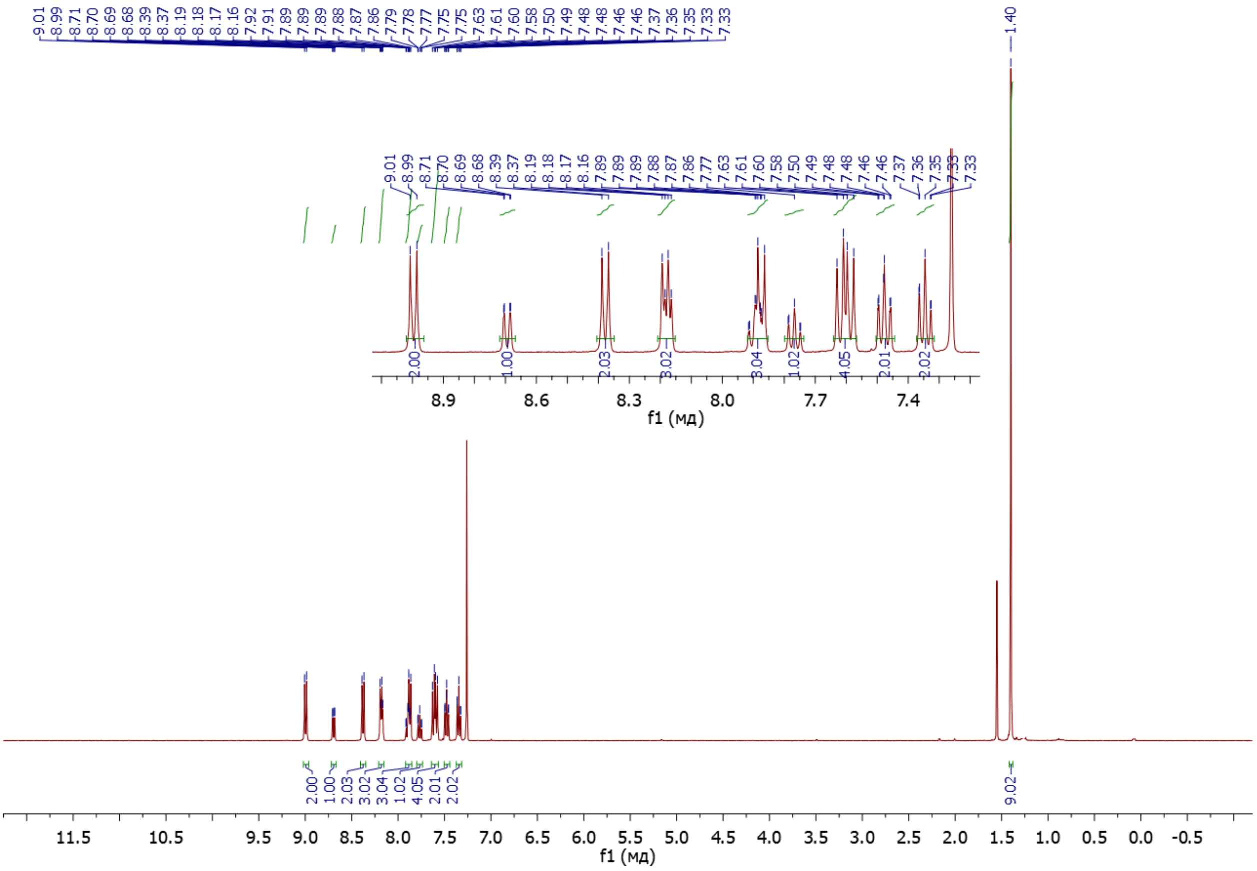
9.01 8.99 8.71 8.70 8.69 8.68 8.39 8.37 8.19 8.18 8.17 8.16 7.92 7.91 7.89 7.89 7.88 7.87 7.86 7.79 7.78 7.77 7.75 7.75 7.63 7.61 7.60 7.58 7.50 7.49 7.48 7.46 7.46 7.37 7.36 7.35 7.33 7.33
1.40
2.00 1.00 2.03 3.02 3.04 1.02 4.05 2.01 2.02
9.02
f1 (мд)

a

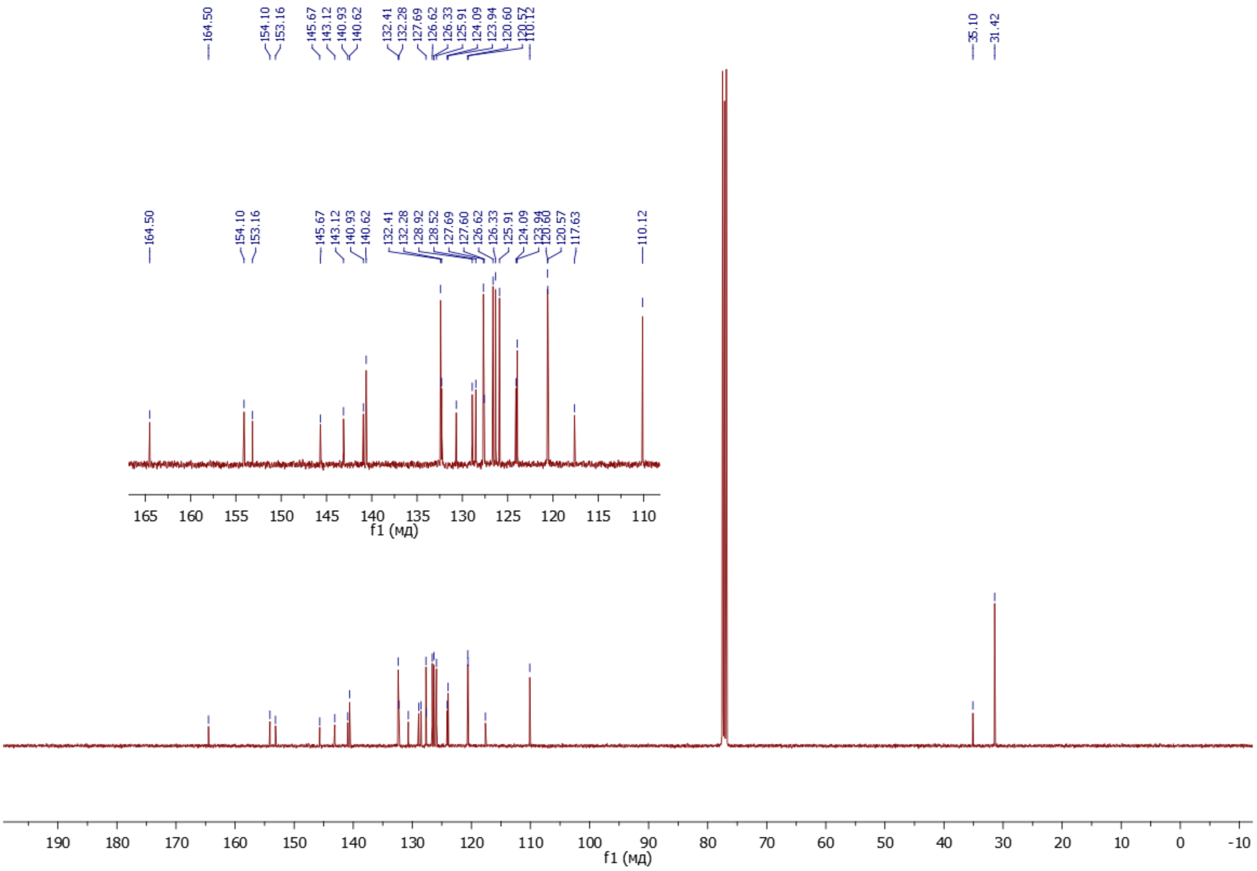
164.50
154.10 153.16
145.67 143.12 140.93 140.62
132.41 132.28 128.92 128.52 127.69 127.60 126.62 126.33 125.91 124.09 123.94 120.60 120.57 117.63
110.12
35.10
31.42
f1 (мд)

b

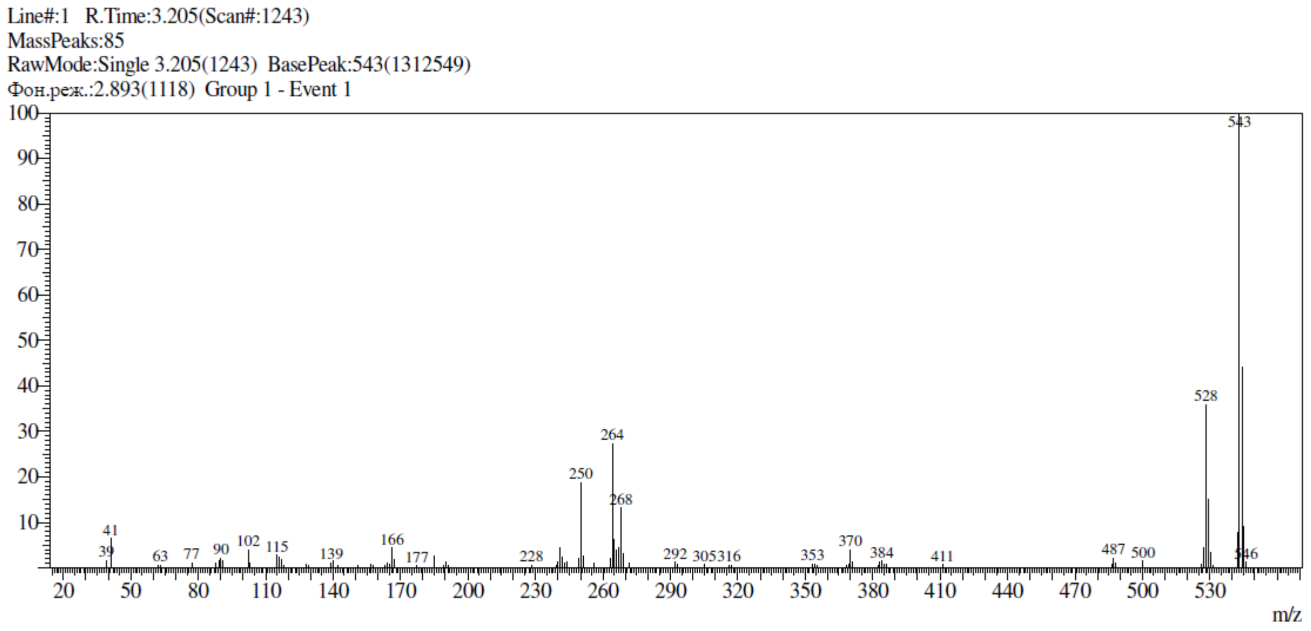

c

Figure S22. ^{1}H NMR (a), ^{13}C NMR (b) and mass (EI) (c) spectra of **6j**.

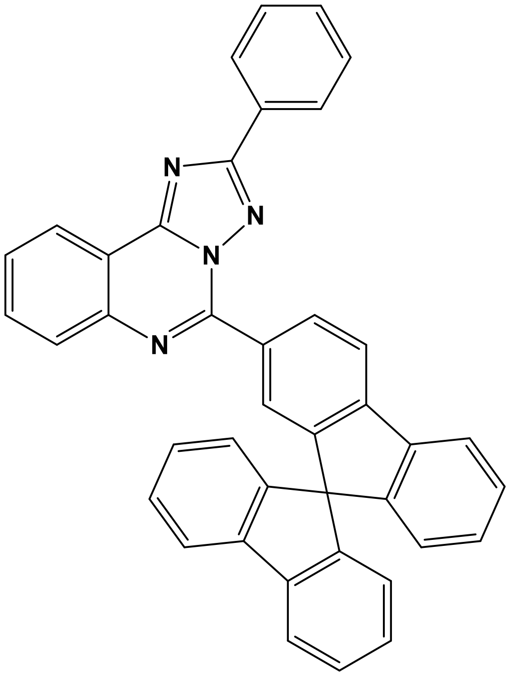

6j

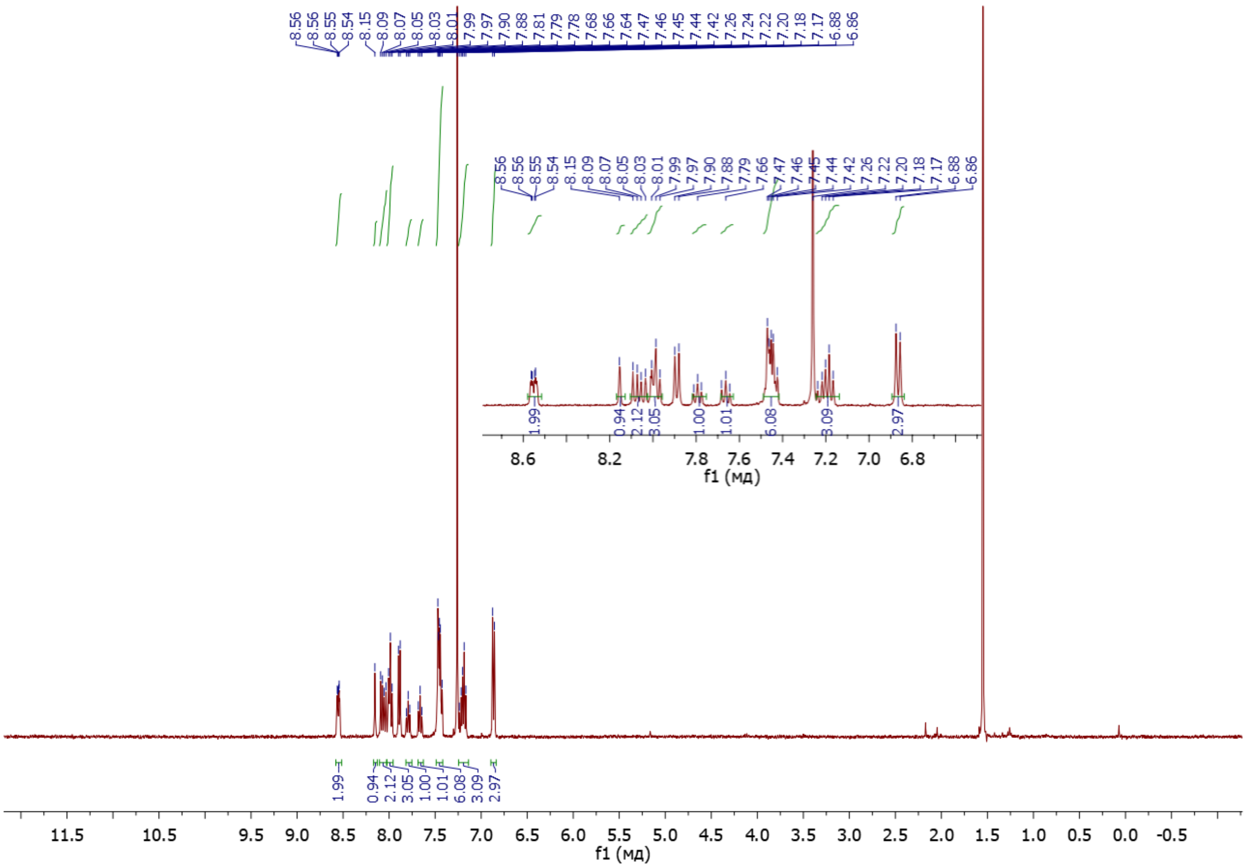
8.56
8.56
8.55
8.54
8.15
8.09
8.07
8.05
8.03
8.01
7.99
7.97
7.90
7.88
7.81
7.79
7.78
7.68
7.66
7.64
7.47
7.46
7.45
7.44
7.42
7.26
7.24
7.22
7.20
7.18
7.17
6.88
6.86
1.99
0.94
2.12
3.05
1.00
1.01
6.08
3.09
2.97
f1 (мд)

a

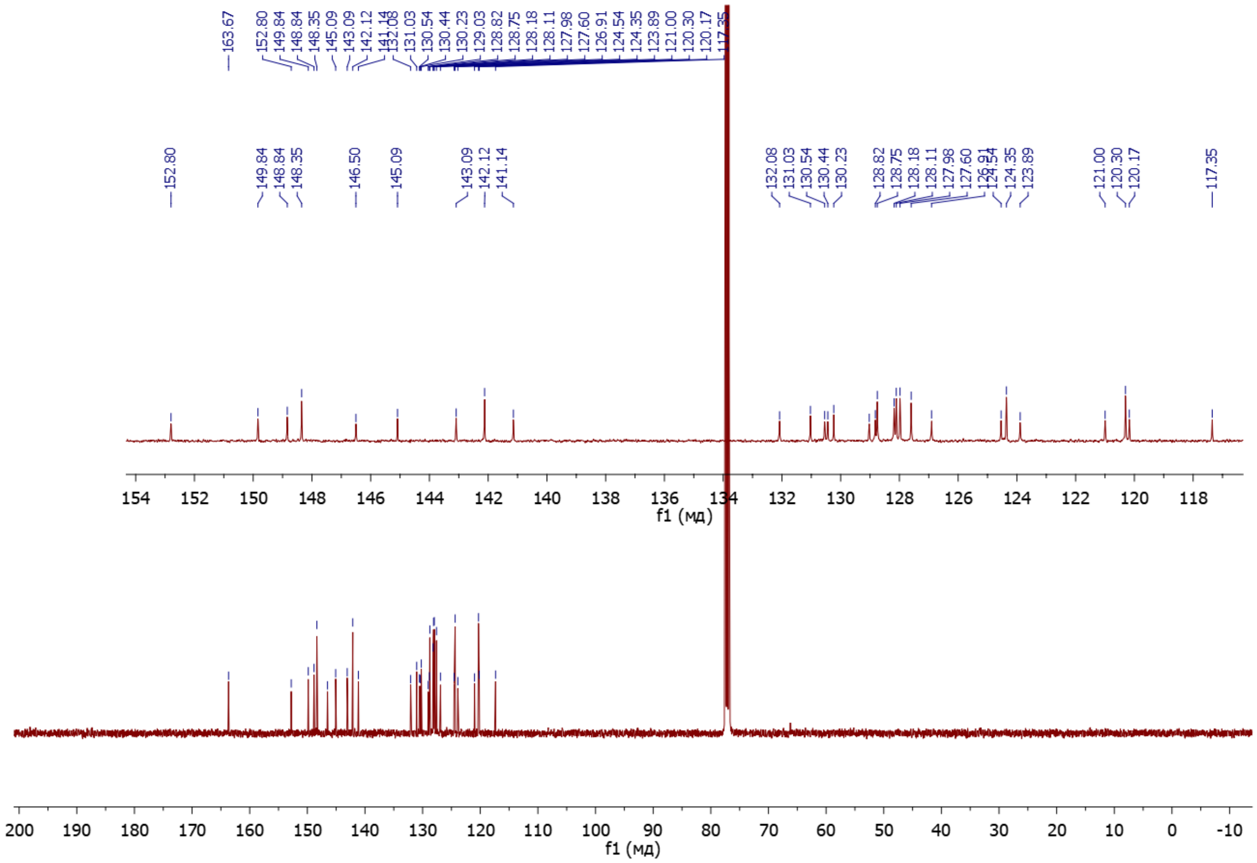
163.67
152.80
149.84
148.84
148.35
146.50
145.09
143.09
142.12
141.14
132.08
131.03
130.54
130.44
130.23
129.03
128.82
128.75
128.18
128.11
127.98
127.60
126.91
124.54
124.35
123.89
121.00
120.30
120.17
117.35
f1 (мд)

b

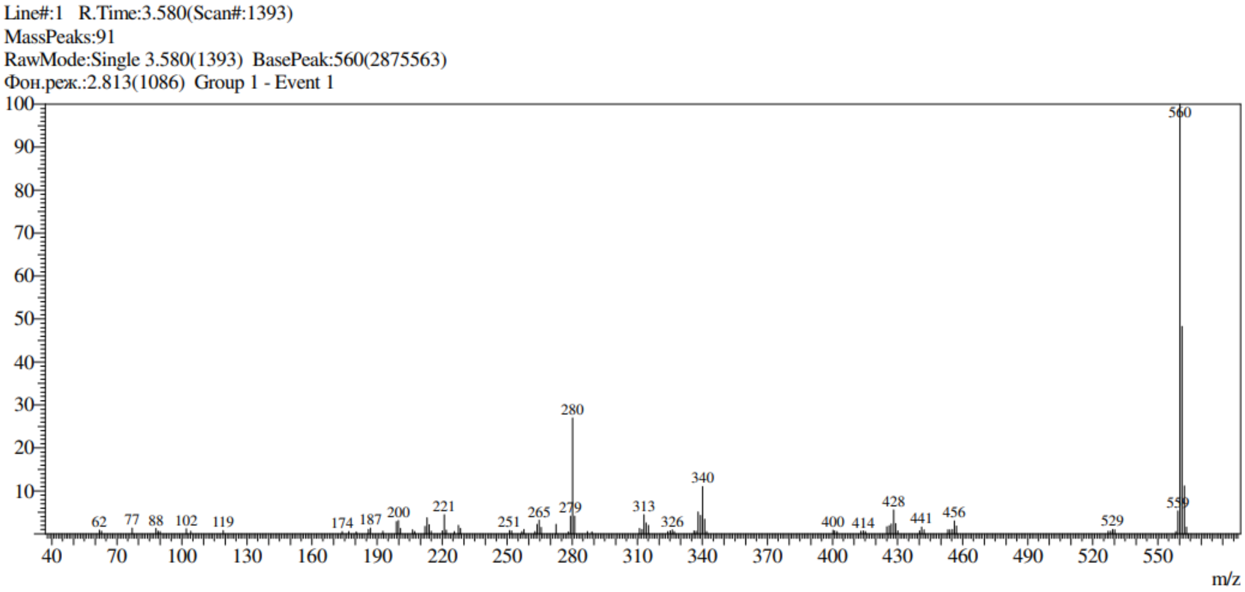

c

Figure S23. ^{1}H NMR (a), ^{13}C NMR (b) and mass (EI) (c) spectra of **6j**.

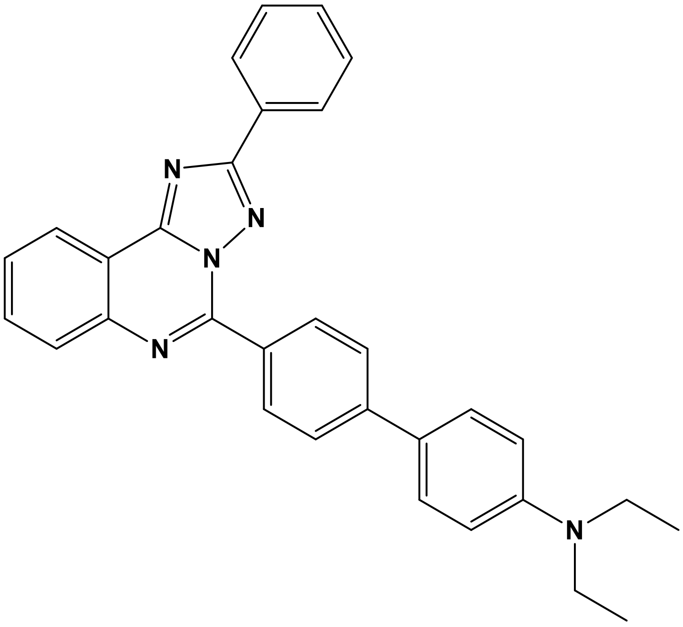

10

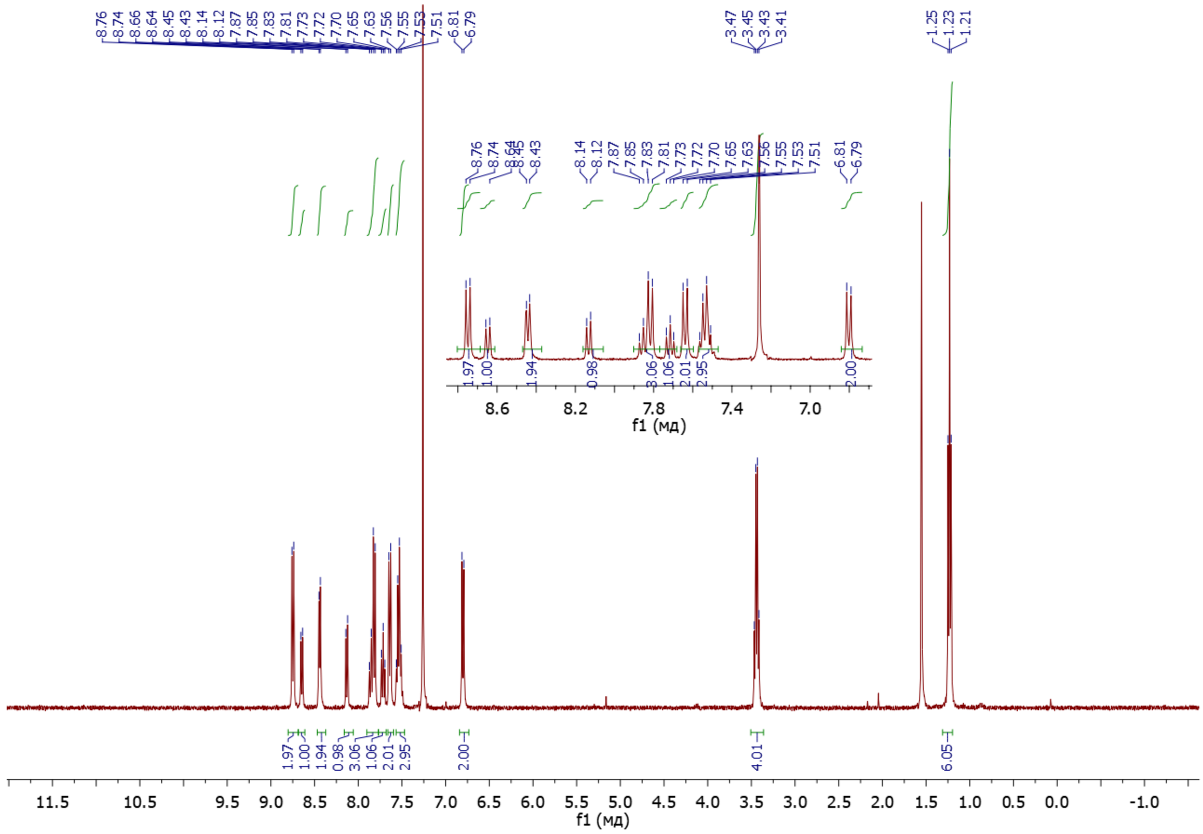

a

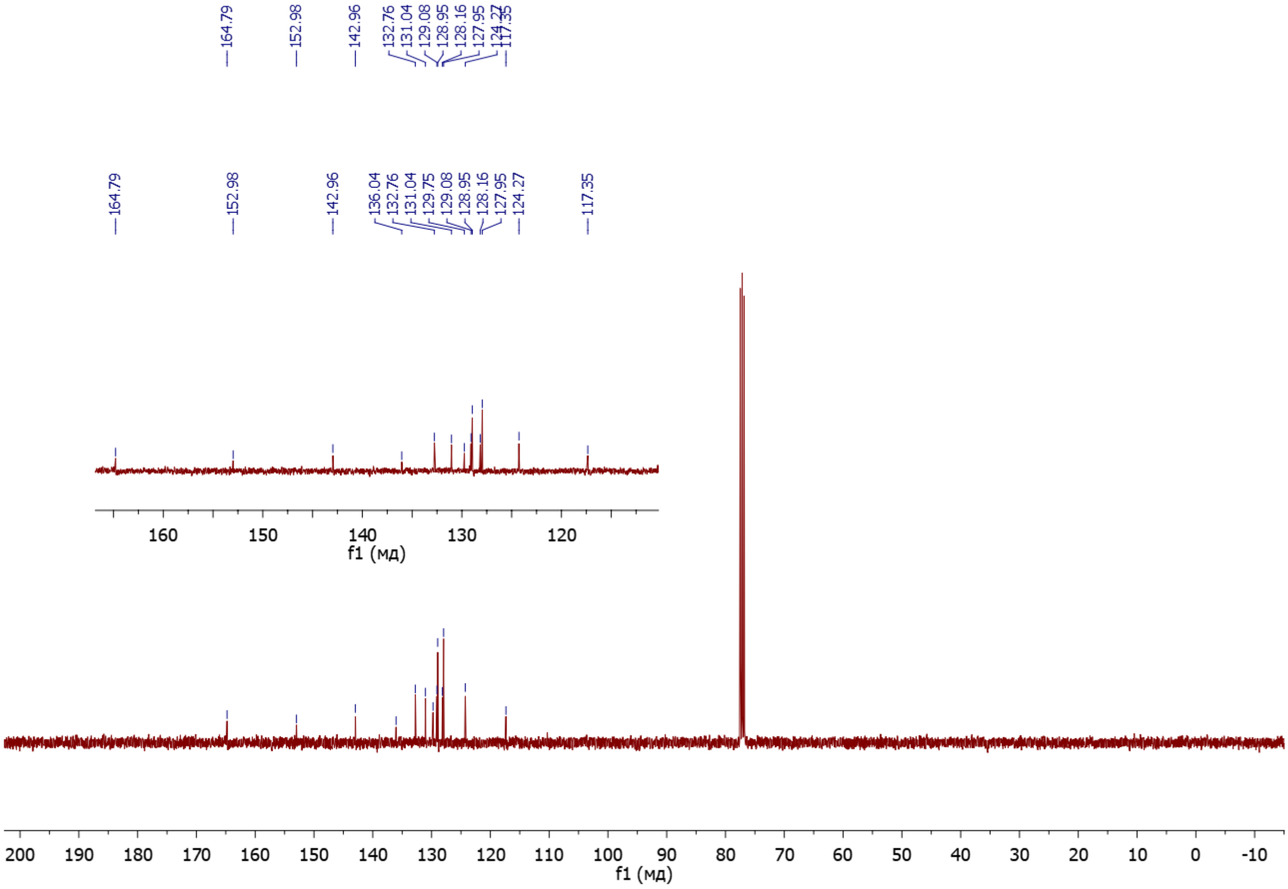

b

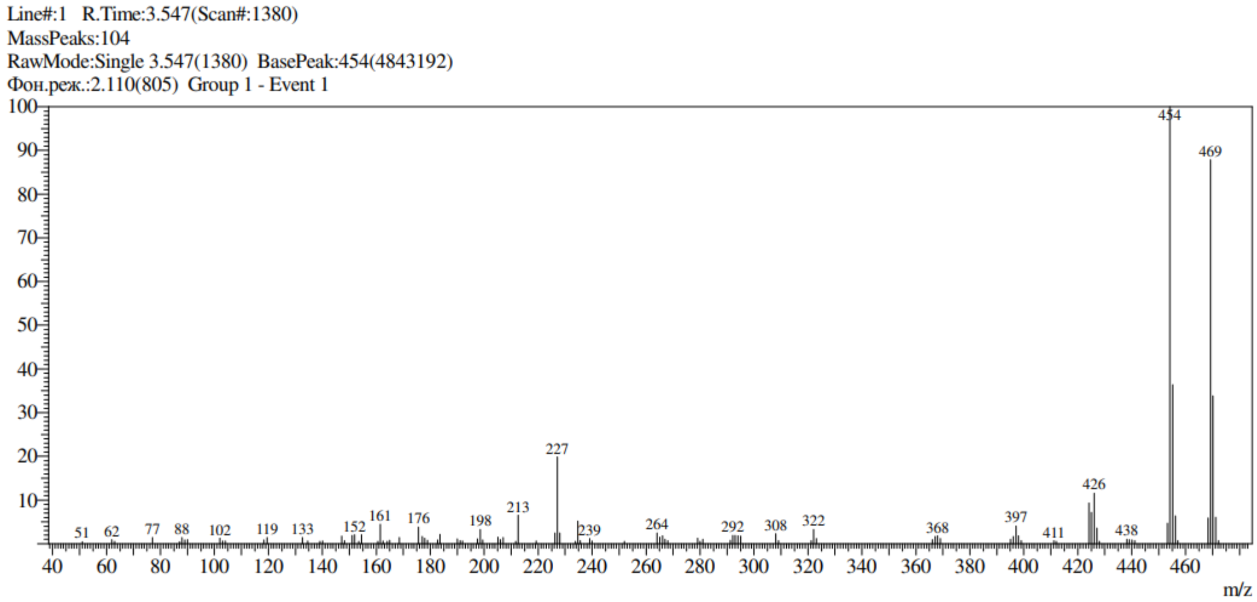

c

Figure S24. ^{1}H NMR (a), ^{13}C NMR (b) and mass (EI) (c) spectra of **10**.

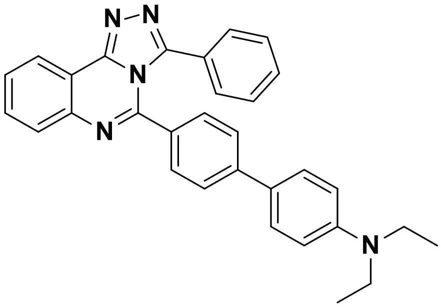

11

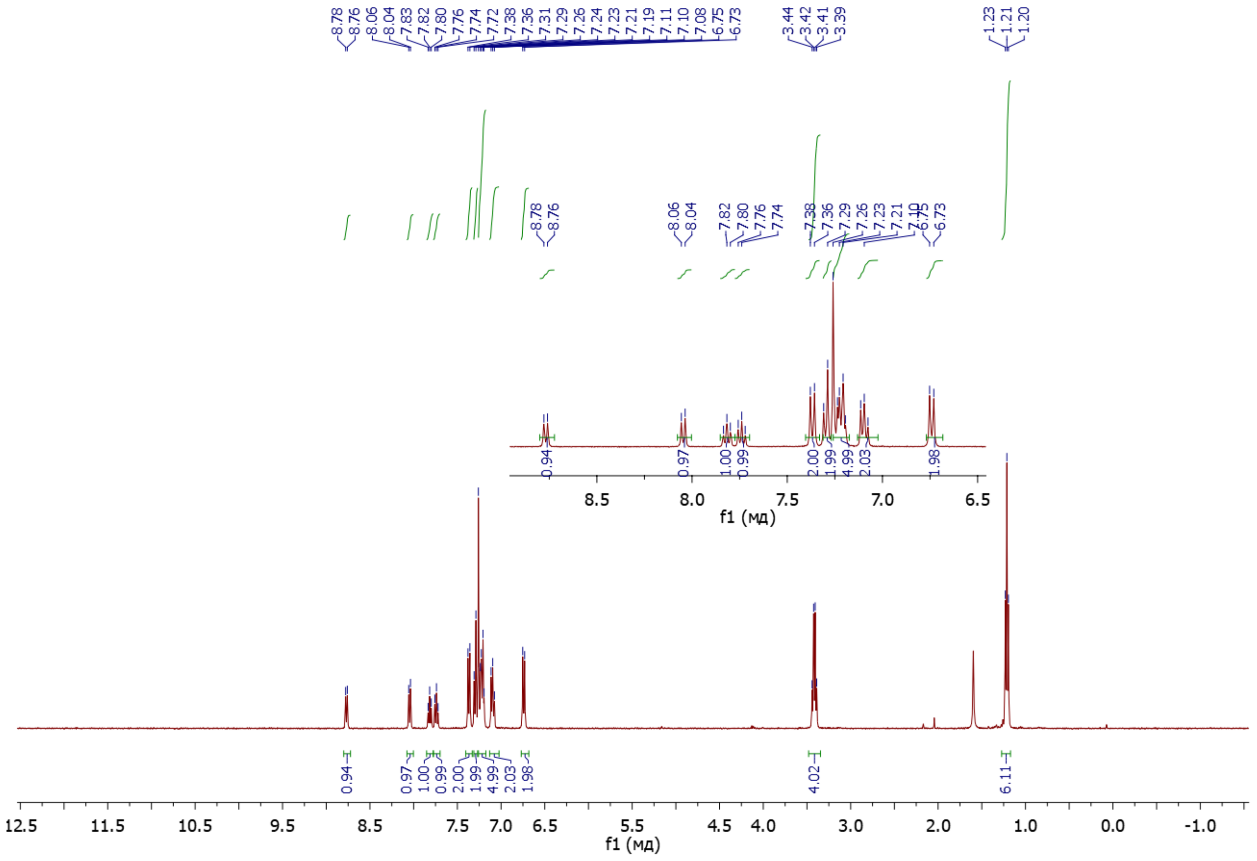

a

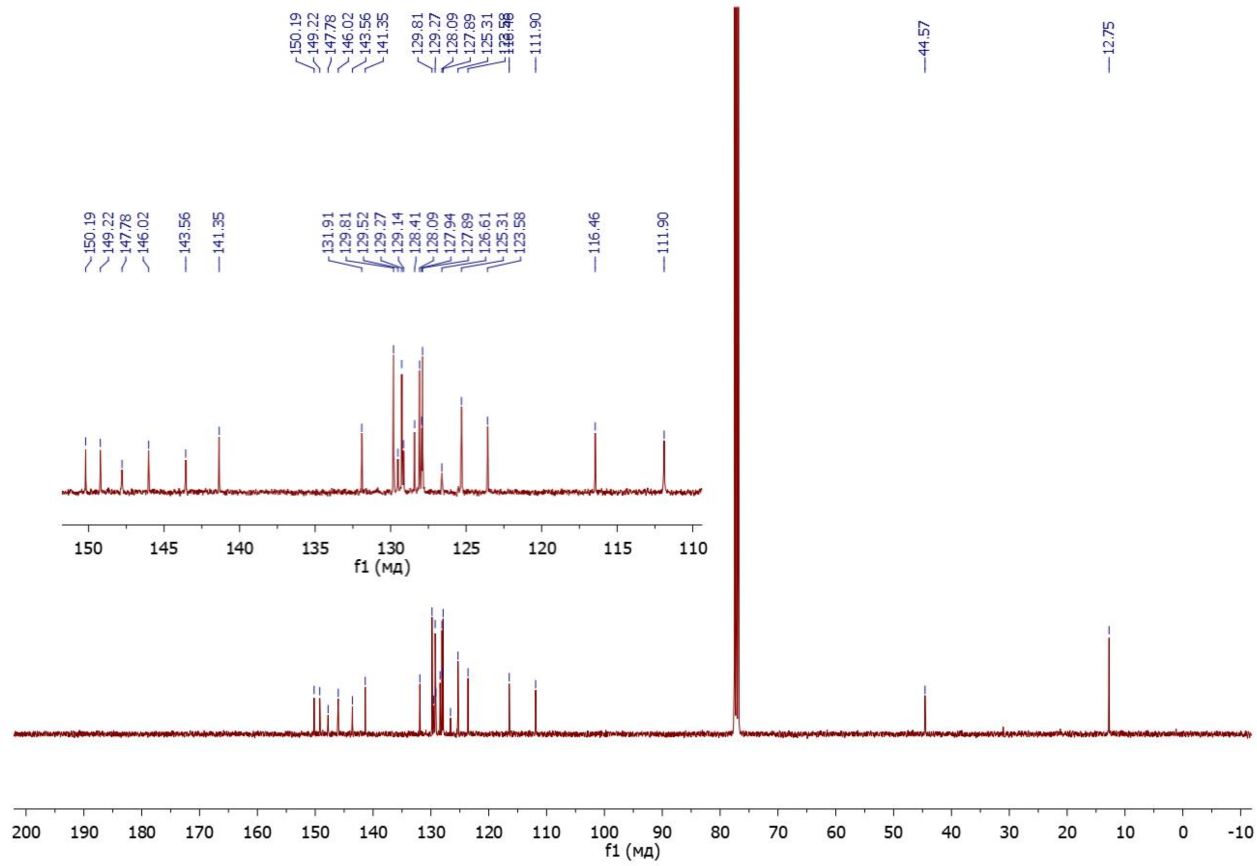

b

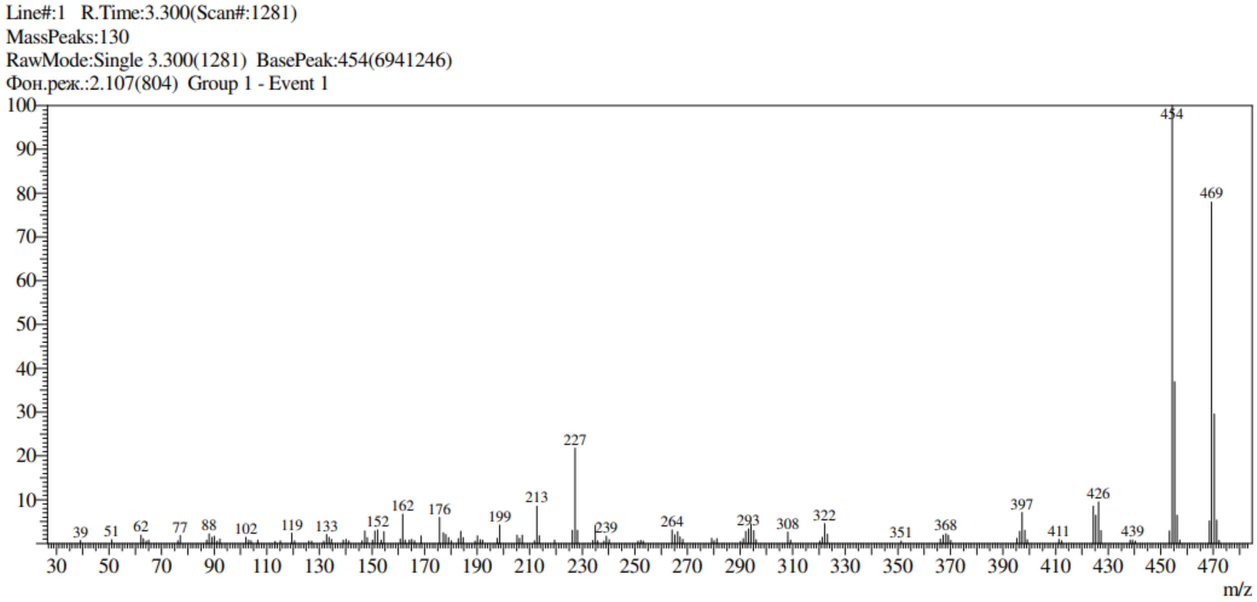

c

Figure S25. ^{1}H NMR (a), ^{13}C NMR (b) and mass (EI) (c) spectra of **11**.

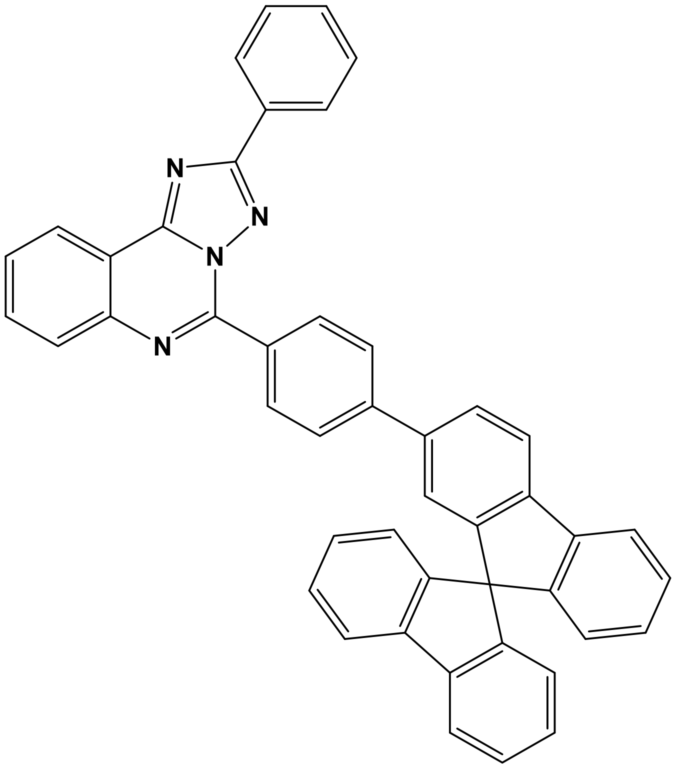

12a

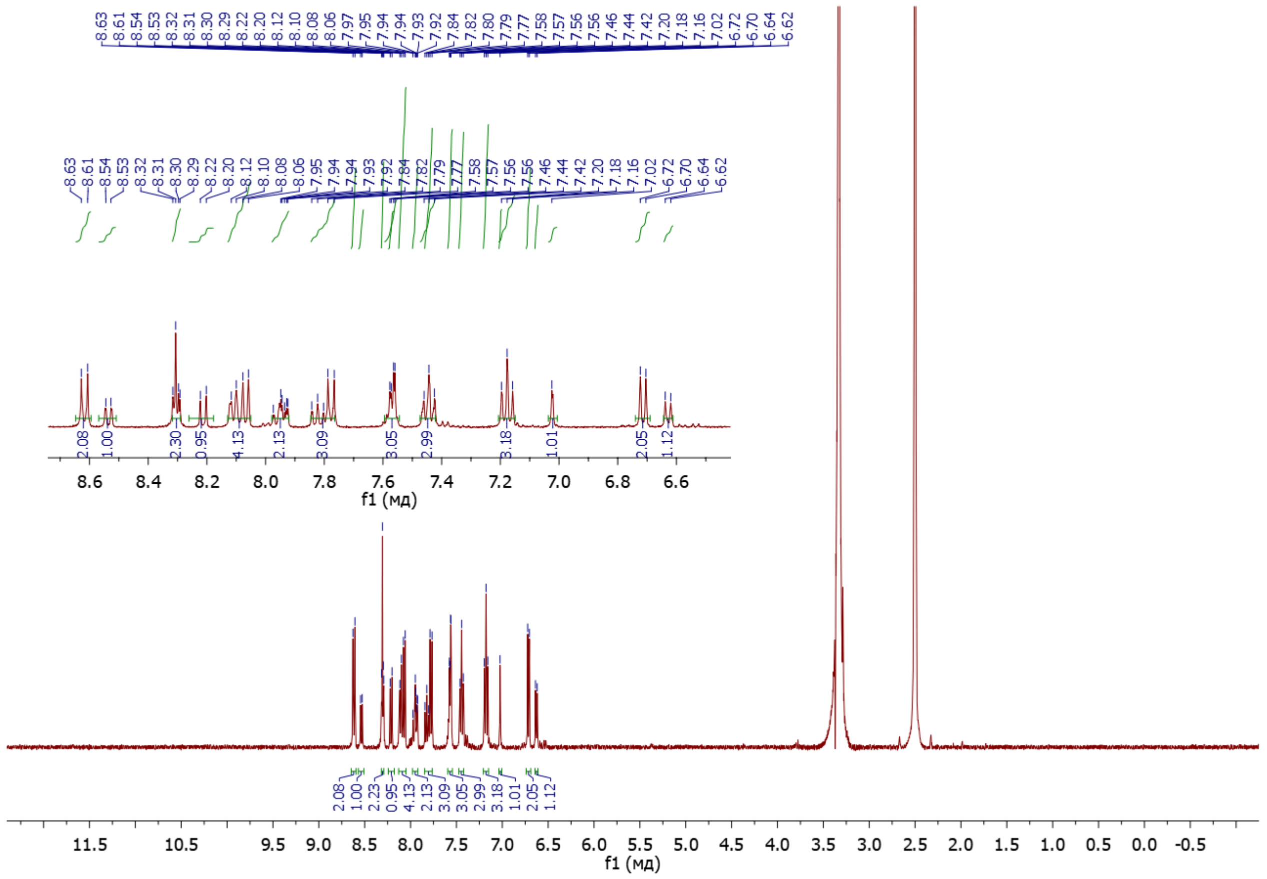

a

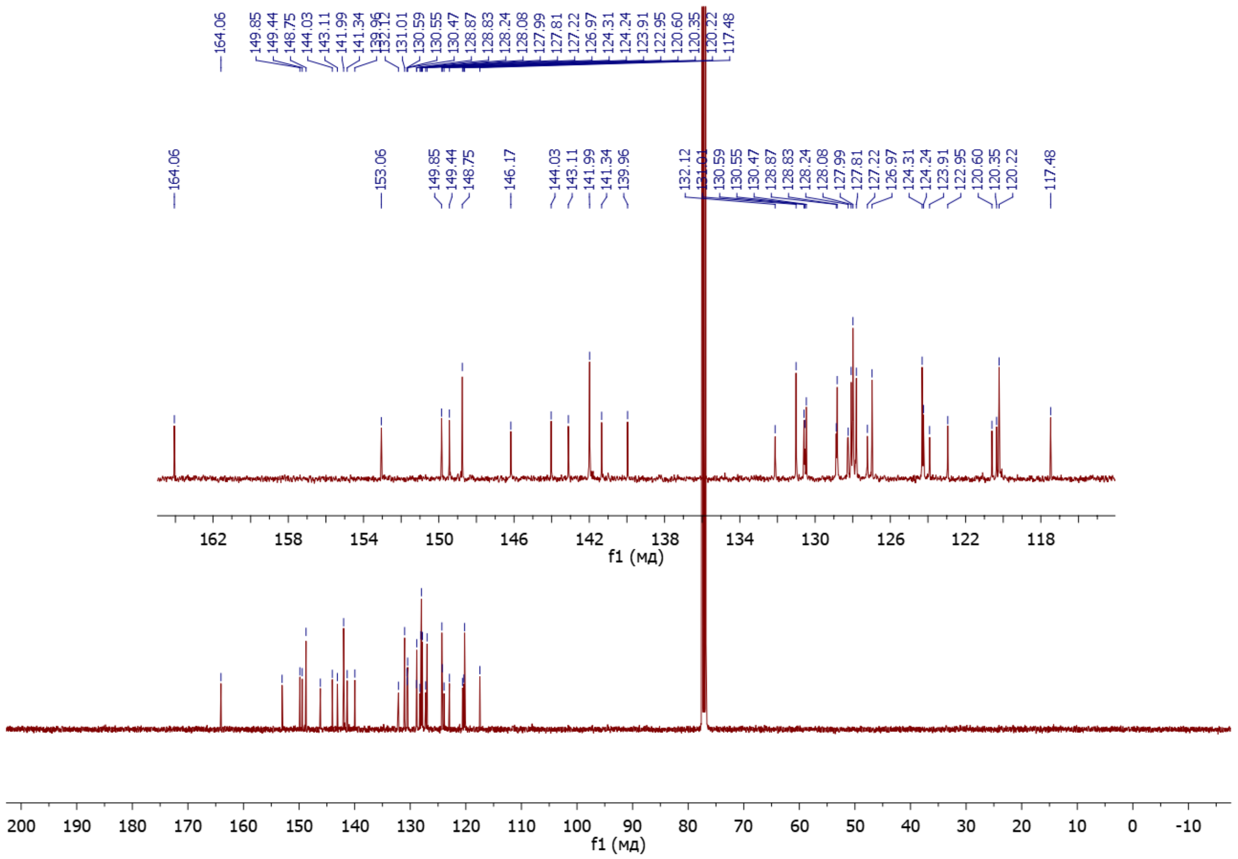

b

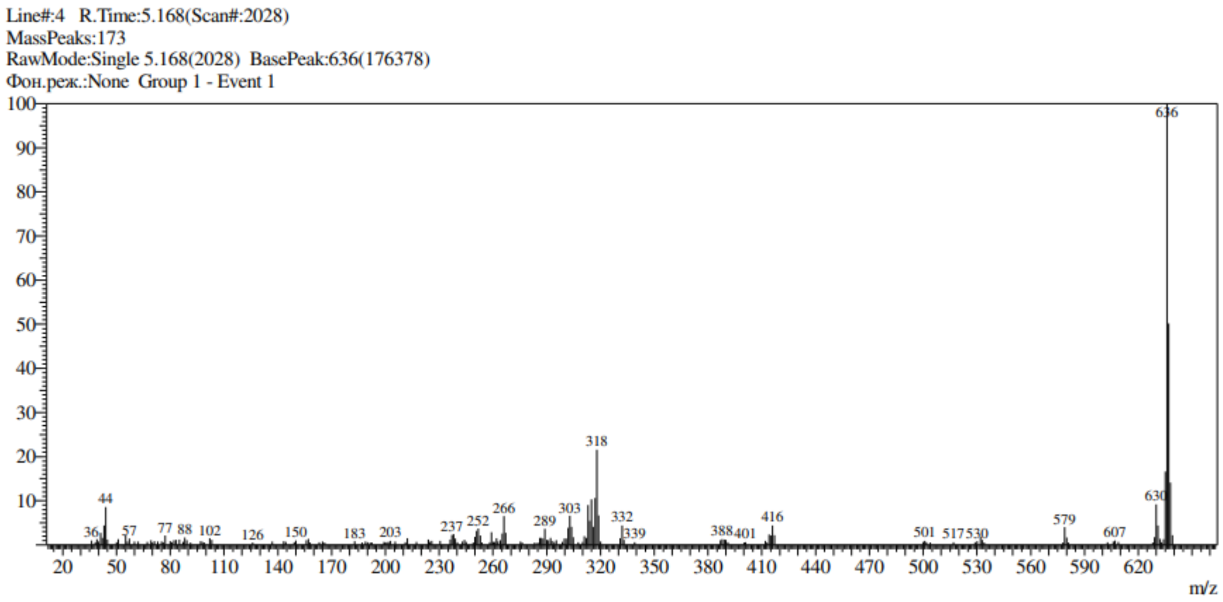

c

Figure S26. ^{1}H NMR (a), ^{13}C NMR (b) and mass (EI) (c) spectra of **12a**.

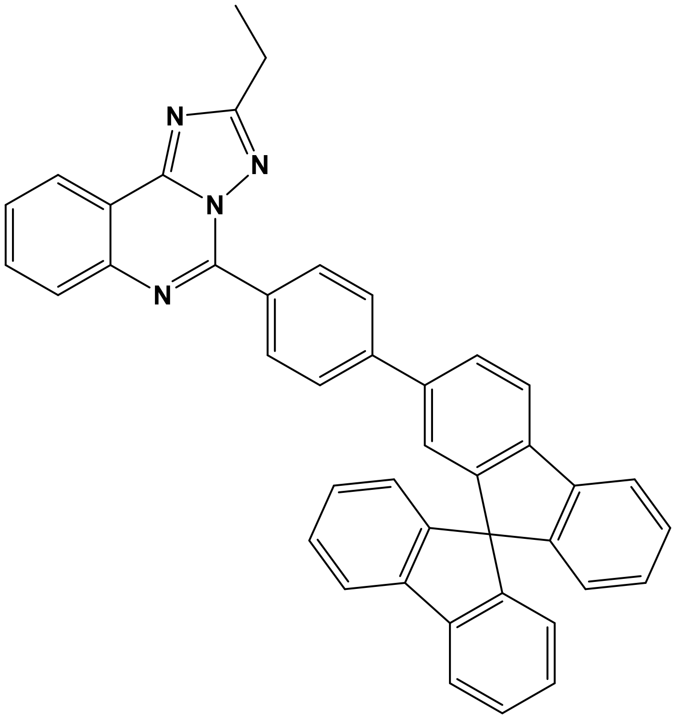

12b

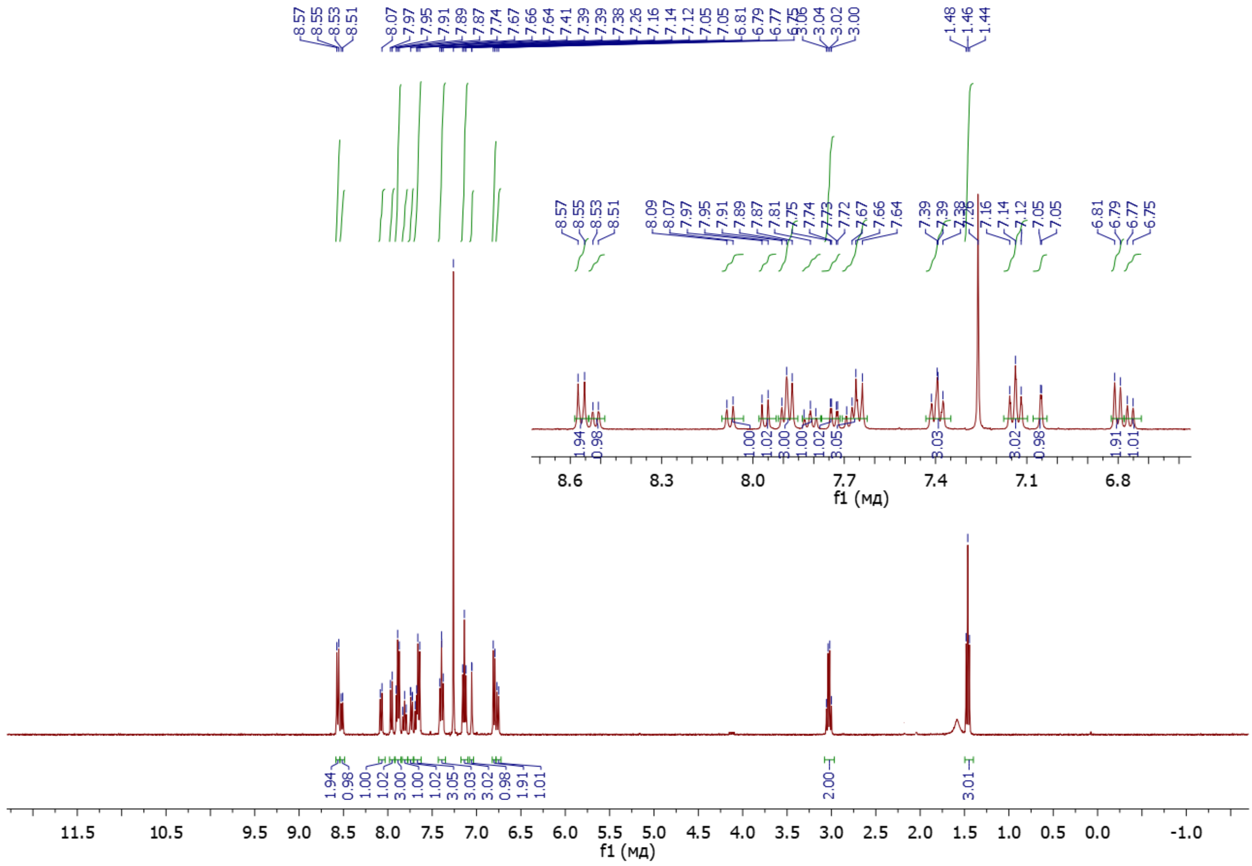

a

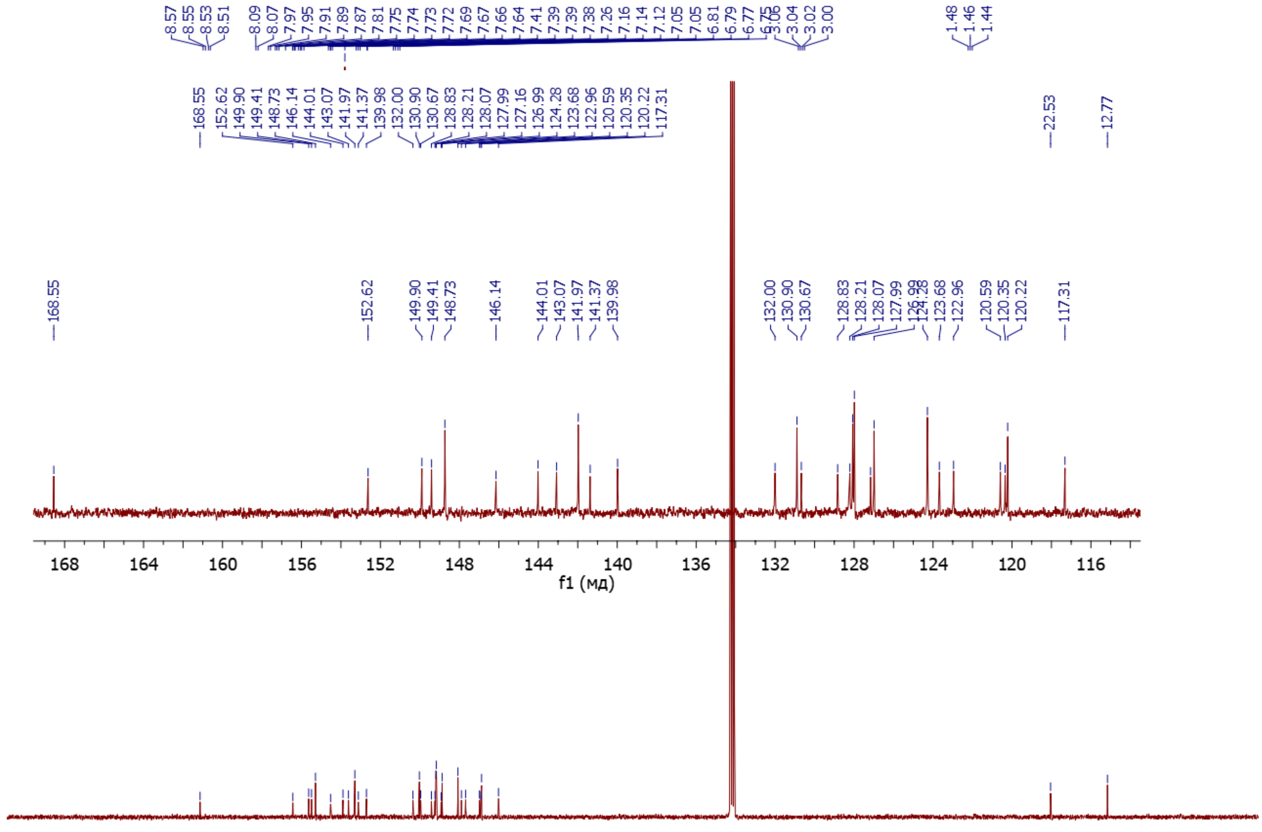

b

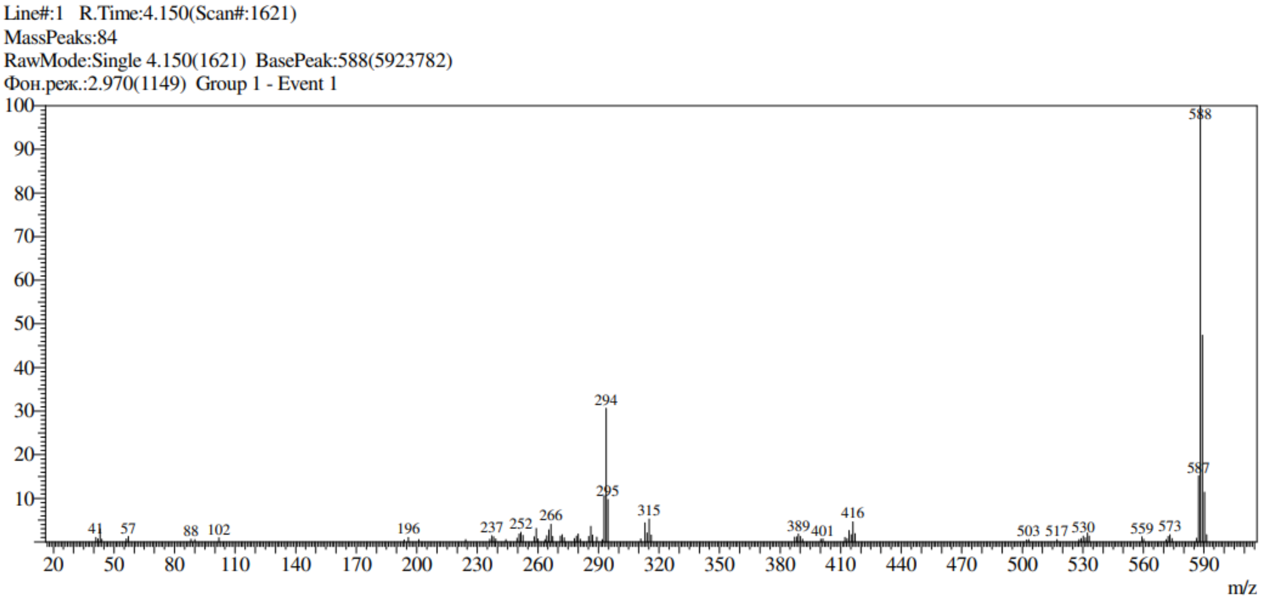

c

Figure S27. ^{1}H NMR (a), ^{13}C NMR (b) and mass (EI) (c) spectra of **12b**.

4. Crystallographic data of triazoloquinazolines

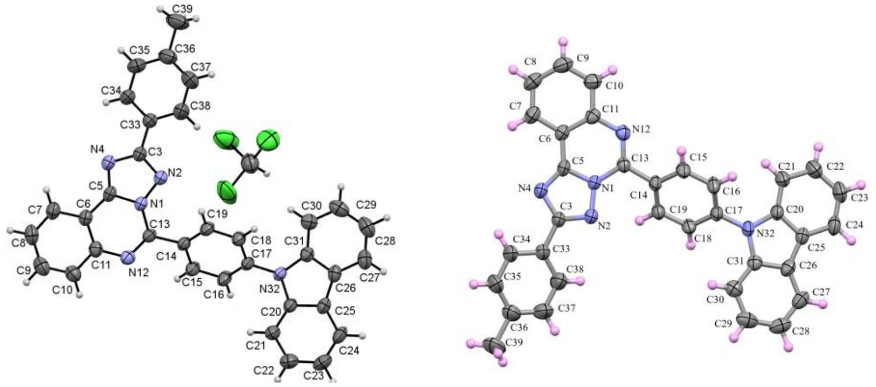

Table S1. Selected bond lengths of compound **6f**.

Bond	Bond length (Å)	Bond	Bond length (Å)
N(1) – N(2)	1.371(4)	C(16) – C(17)	1.387(5)
N(1) – C(5)	1.377(4)	C(17) – C(18)	1.386(5)
N(1) – C(13)	1.392(4)	C(18) – C(19)	1.392(5)
N(2) – C(3)	1.328(4)	C(20) – C(21)	1.379(5)
N(4) – C(3)	1.371(4)	C(20) – C(25)	1.410(5)
N(4) – C(5)	1.314(4)	C(21) – C(22)	1.388(5)
N(12) – C(11)	1.396(4)	C(22) – C(23)	1.392(6)
N(12) – C(13)	1.293(4)	C(23) – C(24)	1.376(6)
N(32) – C(17)	1.420(4)	C(24) – C(25)	1.406(5)
N(32) – C(20)	1.398(4)	C(25) – C(26)	1.432(5)
N(32) – C(31)	1.405(4)	C(26) – C(27)	1.398(5)
C(3) – C(33)	1.476(5)	C(26) – C(31)	1.404(5)
C(5) – C(6)	1.430(5)	C(27) – C(28)	1.371(6)
C(6) – C(7)	1.404(5)	C(28) – C(29)	1.401(6)
C(6) – C(11)	1.402(5)	C(29) – C(30)	1.372(6)
C(7) – C(8)	1.358(6)	C(30) – C(31)	1.388(5)
C(8) – C(9)	1.393(6)	C(33) – C(34)	1.370(5)
C(9) – C(10)	1.374(6)	C(33) – C(38)	1.375(6)
C(10) – C(11)	1.404(5)	C(34) – C(35)	1.379(6)
C(13) – C(14)	1.486(4)	C(35) – C(36)	1.361(6)
C(14) – C(15)	1.394(5)	C(36) – C(37)	1.365(7)
C(14) – C(19)	1.376(5)	C(36) – C(39)	1.522(6)
C(15) – C(16)	1.385(5)	C(37) – C(38)	1.380(6)

Table S2. Selected bond angles of compound **6f**.

Angle	(°)	Angle	(°)
N(2) – N(1) – C(5)	109.6(3)	C(18) – C(17) – N(32)	119.8(3)
N(2) – N(1) – C(13)	127.5(3)	C(18) – C(17) – C(16)	119.6(3)
C(5) – N(1) – C(13)	122.9(3)	C(17) – C(18) – C(19)	119.7(3)
C(3) – N(2) – N(1)	101.7(3)	C(14) – C(19) – C(18)	120.9(3)
C(5) – N(4) – C(3)	103.0(3)	N(32) – C(20) – C(25)	108.4(3)
C(13) – N(12) – C(11)	119.8(3)	C(21) – C(20) – N(32)	129.8(3)

C(20) – N(32) – C(17)	125.5(3)	C(21) – C(20) – C(25)	121.8(3)
C(20) – N(32) – C(31)	108.3(3)	C(20) – C(21) – C(22)	117.8(4)
C(31) – N(32) – C(17)	126.1(3)	C(21) – C(22) – C(23)	121.9(4)
N(2) – C(3) – N(4)	115.7(3)	C(24) – C(23) – C(22)	120.0(4)
N(2) – C(3) – C(33)	122.3(3)	C(23) – C(24) – C(25)	119.7(4)
N(4) – C(3) – C(33)	122.0(3)	C(20) – C(25) – C(26)	107.3(3)
N(1) – C(5) – C(6)	117.6(3)	C(24) – C(25) – C(20)	118.8(3)
N(4) – C(5) – N(1)	110.0(3)	C(24) – C(25) – C(26)	133.9(3)
N(4) – C(5) – C(6)	132.4(3)	C(27) – C(26) – C(25)	133.5(4)
C(7) – C(6) – C(5)	123.1(3)	C(27) – C(26) – C(31)	119.1(4)
C(11) – C(6) – C(5)	116.3(3)	C(31) – C(26) – C(25)	107.4(3)
C(11) – C(6) – C(7)	120.6(3)	C(28) – C(27) – C(26)	119.0(4)
C(8) – C(7) – C(6)	119.7(4)	C(27) – C(28) – C(29)	121.1(4)
C(7) – C(8) – C(9)	120.2(4)	C(30) – C(29) – C(28)	120.9(4)
C(10) – C(9) – C(8)	121.4(4)	C(29) – C(30) – C(31)	118.1(4)
C(9) – C(10) – C(11)	119.5(4)	C(26) – C(31) – N(32)	108.5(3)
N(12) – C(11) – C(6)	122.8(3)	C(30) – C(31) – N(32)	129.7(3)
N(12) – C(11) – C(10)	118.5(3)	C(30) – C(31) – C(26)	121.7(3)
C(6) – C(11) – C(10)	118.7(3)	C(34) – C(33) – C(3)	120.0(3)
N(1) – C(13) – C(14)	118.6(3)	C(34) – C(33) – C(38)	117.6(4)
N(12) – C(13) – N(1)	120.5(3)	C(38) – C(33) – C(3)	122.4(3)
N(12) – C(13) – C(14)	120.9(3)	C(33) – C(34) – C(35)	120.6(4)
C(15) – C(14) – C(13)	118.3(3)	C(36) – C(35) – C(34)	122.0(4)
C(19) – C(14) – C(13)	122.3(3)	C(35) – C(36) – C(37)	117.3(4)
C(19) – C(14) – C(15)	119.3(3)	C(35) – C(36) – C(39)	121.5(5)
C(16) – C(15) – C(14)	120.1(3)	C(37) – C(36) – C(39)	121.1(4)
C(15) – C(16) – C(17)	120.4(3)	C(36) – C(37) – C(38)	121.4(4)
C(16) – C(17) – N(32)	120.6(3)	C(33) – C(38) – C(37)	120.9(4)

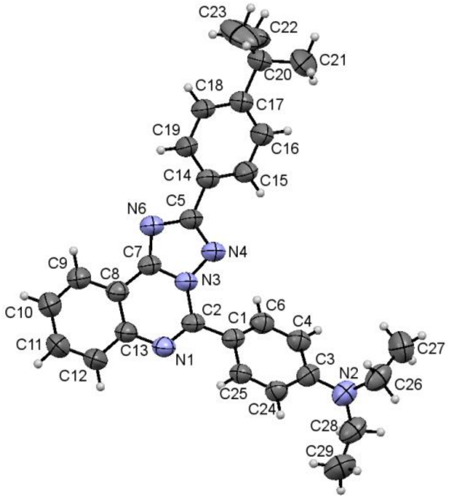

Table S3. Selected bond lengths of compound **6g**.

Bond	Bond length (Å)	Bond	Bond length (Å)
N(1) – C(2)	1.288(3)	C(8) – C(13)	1.395(4)
N(1) – C(13)	1.383(3)	C(9) – C(10)	1.356(4)
N(2) – C(3)	1.374(4)	C(10) – C(11)	1.391(5)
N(2) – C(26)	1.552(7)	C(11) – C(12)	1.365(5)
N(2) – C(28)	1.395(7)	C(12) – C(13)	1.402(4)

N(3) – N(4)	1.375(3)	C(14) – C(15)	1.370(4)
N(3) – C(2)	1.400(3)	C(14) – C(19)	1.376(4)
N(3) – C(7)	1.367(3)	C(15) – C(16)	1.370(4)
N(4) – C(5)	1.331(3)	C(16) – C(17)	1.382(4)
N(6) – C(5)	1.365(3)	C(17) – C(18)	1.372(4)
N(6) – C(7)	1.320(3)	C(17) – C(20)	1.525(4)
C(1) – C(2)	1.461(4)	C(18) – C(19)	1.381(4)
C(1) – C(6)	1.346(6)	C(20) – C(21)	1.505(5)
C(1) – C(25)	1.406(6)	C(20) – C(22)	1.552(6)
C(3) – C(4)	1.428(6)	C(20) – C(23)	1.511(5)
C(3) – C(24)	1.409(6)	C(27) – C(26)	1.543(8)
C(5) – C(14)	1.464(4)	C(29) – C(28)	1.524(8)
C(7) – C(8)	1.421(4)	C(4) – C(6)	1.389(7)
C(8) – C(9)	1.398(4)	C(24) – C(25)	1.353(7)

Table S4. Selected bond angles of compound **6g**.

**Angle**	**(°)**	**Angle**	**(°)**
C(2) – N(1) – C(13)	120.9(2)	C(12) – C(11) – C(10)	121.0(3)
C(3) – N(2) – C(26)	120.2(3)	C(11) – C(12) – C(13)	120.3(3)
C(3) – N(2) – C(28)	124.8(4)	N(1) – C(13) – C(8)	122.9(2)
C(28) – N(2) – C(26)	113.8(4)	N(1) – C(13) – C(12)	119.0(3)
N(4) – N(3) – C(2)	127.1(2)	C(8) – C(13) – C(12)	118.1(3)
C(7) – N(3) – N(4)	109.6(2)	C(15) – C(14) – C(5)	122.3(3)
C(7) – N(3) – C(2)	123.2(2)	C(15) – C(14) – C(19)	117.5(3)
C(5) – N(4) – N(3)	101.7(2)	C(19) – C(14) – C(5)	120.1(3)
C(7) – N(6) – C(5)	103.0(2)	C(16) – C(15) – C(14)	121.2(3)
C(6) – C(1) – C(2)	124.8(3)	C(15) – C(16) – C(17)	122.2(3)
C(6) – C(1) – C(25)	118.6(4)	C(16) – C(17) – C(20)	122.3(3)
C(25) – C(1) – C(2)	116.4(3)	C(18) – C(17) – C(16)	116.0(3)
N(1) – C(2) – N(3)	118.9(2)	C(18) – C(17) – C(20)	121.6(3)
N(1) – C(2) – C(1)	120.7(2)	C(17) – C(18) – C(19)	122.2(3)
N(3) – C(2) – C(1)	120.4(2)	C(14) – C(19) – C(18)	120.7(3)
N(2) – C(3) – C(4)	123.2(3)	C(17) – C(20) – C(22)	106.9(3)
N(2) – C(3) – C(24)	122.5(3)	C(21) – C(20) – C(17)	111.0(3)
C(24) – C(3) – C(4)	114.1(4)	C(21) – C(20) – C(22)	106.7(4)
N(4) – C(5) – N(6)	115.7(2)	C(21) – C(20) – C(23)	114.4(4)
N(4) – C(5) – C(14)	121.7(3)	C(23) – C(20) – C(17)	112.6(3)
N(6) – C(5) – C(14)	122.5(2)	C(23) – C(20) – C(22)	104.6(4)
N(3) – C(7) – C(8)	118.1(2)	C(6) – C(4) – C(3)	121.5(5)
N(6) – C(7) – N(3)	110.1(2)	C(1) – C(6) – C(4)	121.6(5)
N(6) – C(7) – C(8)	131.8(3)	C(25) – C(24) – C(3)	123.5(5)
C(9) – C(8) – C(7)	123.4(3)	C(24) – C(25) – C(1)	120.3(5)
C(13) – C(8) – C(7)	115.9(3)	C(27) – C(26) – N(2)	105.9(4)
C(13) – C(8) – C(9)	120.7(3)	N(2) – C(28) – C(29)	116.3(5)
C(10) – C(9) – C(8)	120.1(3)		
C(9) – C(10) – C(11)	119.9(3)		

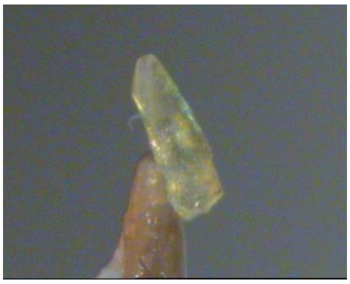

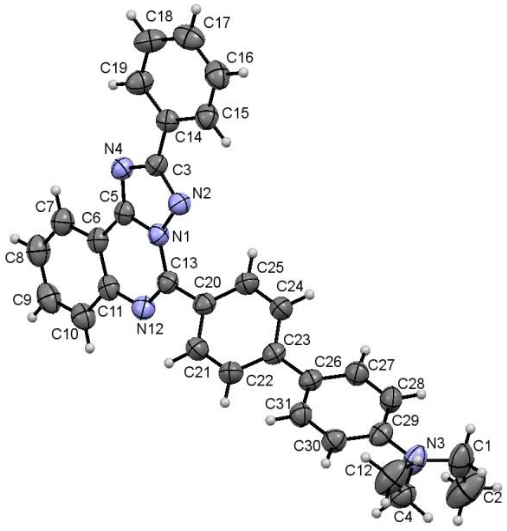

Crystal of **10** **10**

Table S5. Selected bond lengths of compound **10**.

**Bond**	**Bond length (Å)**	**Bond**	**Bond length (Å)**
N(1) – N(2)	1.375(2)	C(26) – C(27)	1.398(3)
N(1) – C(5)	1.367(2)	C(26) – C(31)	1.396(3)
N(1) – C(13)	1.391(2)	C(29) – C(30)	1.405(3)
N(4) – C(5)	1.322(2)	C(29) – C(28)	1.404(3)
N(4) – C(3)	1.370(2)	C(6) – C(11)	1.401(3)
N(2) – C(3)	1.325(2)	C(6) – C(7)	1.397(3)
N(12) – C(13)	1.302(2)	C(11) – C(10)	1.404(3)
N(12) – C(11)	1.388(2)	C(24) – C(25)	1.383(3)
N(3) – C(29)	1.385(2)	C(27) – C(28)	1.376(3)
N(3) – C(4)	1.449(3)	C(30) – C(31)	1.374(3)
N(3) – C(1)	1.457(3)	C(22) – C(21)	1.372(3)
C(5) – C(6)	1.439(3)	C(15) – C(16)	1.378(3)
C(23) – C(26)	1.474(3)	C(10) – C(9)	1.363(3)
C(23) – C(24)	1.399(3)	C(19) – C(18)	1.382(3)
C(23) – C(22)	1.393(3)	C(4) – C(12)	1.500(3)
C(20) – C(13)	1.474(3)	C(7) – C(8)	1.369(3)
C(20) – C(25)	1.394(3)	C(9) – C(8)	1.392(3)
C(20) – C(21)	1.388(3)	C(18) – C(17)	1.371(3)
C(3) – C(14)	1.470(2)	C(16) – C(17)	1.369(3)
C(14) – C(15)	1.390(3)	C(1) – C(2)	1.490(4)
C(14) – C(19)	1.383(3)		

Table S6. Selected bond angles of compound **10**.

**Angle**	**(°)**	**Angle**	**(°)**
N(2) – N(1) – C(13)	126.86(15)	N(3) – C(29) – C(30)	121.95(19)
C(5) – N(1) – N(2)	109.45(15)	N(3) – C(29) – C(28)	122.24(19)
C(5) – N(1) – C(13)	123.57(15)	C(28) – C(29) – C(30)	115.81(18)
C(5) – N(4) – C(3)	102.56(15)	C(11) – C(6) – C(5)	115.96(17)
C(3) – N(2) – N(1)	101.78(14)	C(7) – C(6) – C(5)	123.58(19)
C(13) – N(12) – C(11)	120.09(16)	C(7) – C(6) – C(11)	120.46(18)

C(29) – N(3) – C(4)	120.23(18)	N(12) – C(11) – C(6)	123.05(16)
C(29) – N(3) – C(1)	120.23(18)	N(12) – C(11) – C(10)	118.49(18)
C(4) – N(3) – C(1)	117.65(18)	C(6) – C(11) – C(10)	118.46(18)
N(1) – C(5) – C(6)	117.61(17)	C(25) – C(24) – C(23)	122.13(18)
N(4) – C(5) – N(1)	110.30(15)	C(28) – C(27) – C(26)	122.34(19)
N(4) – C(5) – C(6)	132.08(18)	C(24) – C(25) – C(20)	120.06(19)
C(24) – C(23) – C(26)	122.00(17)	C(31) – C(30) – C(29)	121.78(19)
C(22) – C(23) – C(26)	121.37(18)	C(30) – C(31) – C(26)	122.46(18)
C(22) – C(23) – C(24)	116.62(18)	C(21) – C(22) – C(23)	121.65(19)
C(25) – C(20) – C(13)	123.83(18)	C(16) – C(15) – C(14)	120.5(2)
C(21) – C(20) – C(13)	117.84(17)	C(27) – C(28) – C(29)	121.83(19)
C(21) – C(20) – C(25)	118.14(18)	C(22) – C(21) – C(20)	121.37(19)
N(4) – C(3) – C(14)	122.66(17)	C(9) – C(10) – C(11)	120.3(2)
N(2) – C(3) – N(4)	115.90(16)	C(18) – C(19) – C(14)	120.4(2)
N(2) – C(3) – C(14)	121.43(17)	N(3) – C(4) – C(12)	113.93(19)
N(1) – C(13) – C(20)	120.70(16)	C(8) – C(7) – C(6)	119.7(2)
N(12) – C(13) – N(1)	119.66(17)	C(10) – C(9) – C(8)	120.9(2)
N(12) – C(13) – C(20)	119.63(17)	C(17) – C(18) – C(19)	120.4(2)
C(15) – C(14) – C(3)	121.15(17)	C(17) – C(16) – C(15)	120.4(2)
C(19) – C(14) – C(3)	120.26(17)	C(16) – C(17) – C(18)	119.8(2)
C(19) – C(14) – C(15)	118.59(18)	C(7) – C(8) – C(9)	120.1(2)
C(27) – C(26) – C(23)	122.58(18)	N(3) – C(1) – C(2)	113.5(2)
C(31) – C(26) – C(27)	115.77(18)		

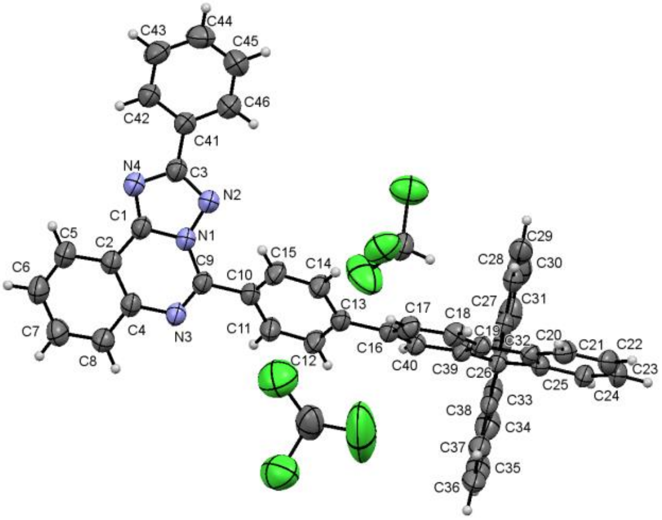

12a

Table S7. Selected bond lengths of compound **12a**.

**Bond**	**Bond length (Å)**	**Angle**	**(°)**
N(1) – N(2)	1.365(3)	C(20) – C(21)	1.396(4)
N(1) – C(1)	1.370(3)	C(20) – C(25)	1.392(4)
N(1) – C(9)	1.397(4)	C(21) – C(22)	1.388(4)
N(2) – C(3)	1.327(4)	C(22) – C(23)	1.382(5)
N(3) – C(4)	1.385(3)	C(23) – C(24)	1.383(4)
N(3) – C(9)	1.287(3)	C(24) – C(25)	1.382(3)
N(4) – C(1)	1.319(4)	C(25) – C(26)	1.529(3)
N(4) – C(3)	1.367(4)	C(26) – C(27)	1.521(4)

C(1) – C(2)	1.428(4)	C(26) – C(38)	1.524(4)
C(2) – C(4)	1.404(4)	C(26) – C(39)	1.533(3)
C(2) – C(5)	1.399(4)	C(27) – C(28)	1.371(4)
C(3) – C(41)	1.463(4)	C(27) – C(32)	1.402(4)
C(4) – C(8)	1.402(4)	C(28) – C(29)	1.392(4)
C(5) – C(6)	1.371(5)	C(29) – C(30)	1.374(5)
C(6) – C(7)	1.391(5)	C(30) – C(31)	1.379(5)
C(7) – C(8)	1.373(4)	C(31) – C(32)	1.395(5)
C(9) – C(10)	1.486(4)	C(32) – C(33)	1.461(5)
C(10) – C(11)	1.394(4)	C(33) – C(34)	1.393(4)
C(10) – C(15)	1.383(4)	C(33) – C(38)	1.392(4)
C(11) – C(12)	1.374(4)	C(34) – C(35)	1.370(6)
C(12) – C(13)	1.401(4)	C(35) – C(36)	1.384(6)
C(13) – C(14)	1.396(4)	C(36) – C(37)	1.404(5)
C(13) – C(16)	1.486(3)	C(37) – C(38)	1.375(4)
C(14) – C(15)	1.381(4)	C(39) – C(40)	1.371(3)
C(16) – C(17)	1.383(4)	C(41) – C(42)	1.386(4)
C(16) – C(40)	1.410(3)	C(41) – C(46)	1.393(4)
C(17) – C(18)	1.389(4)	C(42) – C(43)	1.377(5)
C(18) – C(19)	1.387(4)	C(43) – C(44)	1.369(5)
C(19) – C(20)	1.468(3)	C(44) – C(45)	1.363(5)
C(19) – C(39)	1.396(4)	C(45) – C(46)	1.378(5)

Table S8. Selected bond angles of compound **12a**.

**Bond**	**Bond length (Å)**	**Angle**	**(°)**
N(2) – N(1) – C(1)	109.2(2)	C(22) – C(21) – C(20)	117.9(3)
N(2) – N(1) – C(9)	128.5(2)	C(23) – C(22) – C(21)	121.4(3)
C(1) – N(1) – C(9)	122.3(2)	C(22) – C(23) – C(24)	120.7(3)
C(3) – N(2) – N(1)	102.1(2)	C(25) – C(24) – C(23)	118.6(3)
C(9) – N(3) – C(4)	120.3(2)	C(20) – C(25) – C(26)	110.8(2)
C(1) – N(4) – C(3)	102.4(2)	C(24) – C(25) – C(20)	121.1(2)
N(1) – C(1) – C(2)	118.3(2)	C(24) – C(25) – C(26)	128.0(2)
N(4) – C(1) – N(1)	110.5(3)	C(25) – C(26) – C(39)	101.1(2)
N(4) – C(1) – C(2)	131.2(2)	C(27) – C(26) – C(25)	110.2(2)
C(4) – C(2) – C(1)	115.9(2)	C(27) – C(26) – C(38)	101.3(2)
C(5) – C(2) – C(1)	123.2(3)	C(27) – C(26) – C(39)	113.7(2)
C(5) – C(2) – C(4)	121.0(3)	C(38) – C(26) – C(25)	114.8(2)
N(2) – C(3) – N(4)	115.7(3)	C(38) – C(26) – C(39)	116.2(2)
N(2) – C(3) – C(41)	122.3(3)	C(28) – C(27) – C(26)	128.4(2)
N(4) – C(3) – C(41)	122.0(2)	C(28) – C(27) – C(32)	120.6(3)
N(3) – C(4) – C(2)	122.8(3)	C(32) – C(27) – C(26)	110.8(2)
N(3) – C(4) – C(8)	118.4(3)	C(27) – C(28) – C(29)	119.9(3)
C(8) – C(4) – C(2)	118.7(2)	C(30) – C(29) – C(28)	119.5(3)
C(6) – C(5) – C(2)	119.0(3)	C(29) – C(30) – C(31)	121.5(3)
C(5) – C(6) – C(7)	120.6(3)	C(30) – C(31) – C(32)	119.1(3)
C(8) – C(7) – C(6)	121.1(3)	C(27) – C(32) – C(33)	108.0(3)
C(7) – C(8) – C(4)	119.7(3)	C(31) – C(32) – C(27)	119.3(3)
N(1) – C(9) – C(10)	120.1(2)	C(31) – C(32) – C(33)	132.7(3)
N(3) – C(9) – N(1)	120.3(2)	C(34) – C(33) – C(32)	130.4(3)
N(3) – C(9) – C(10)	119.5(2)	C(38) – C(33) – C(32)	109.4(2)
C(11) – C(10) – C(9)	117.4(3)	C(38) – C(33) – C(34)	120.2(3)
C(15) – C(10) – C(9)	124.0(2)	C(35) – C(34) – C(33)	118.7(4)

C(15) – C(10) – C(11)	118.5(2)	C(34) – C(35) – C(36)	121.2(3)
C(12) – C(11) – C(10)	120.9(3)	C(35) – C(36) – C(37)	120.6(4)
C(11) – C(12) – C(13)	121.3(3)	C(38) – C(37) – C(36)	117.9(4)
C(12) – C(13) – C(16)	122.0(2)	C(33) – C(38) – C(26)	110.3(3)
C(14) – C(13) – C(12)	117.1(2)	C(37) – C(38) – C(26)	128.3(3)
C(14) – C(13) – C(16)	120.9(3)	C(37) – C(38) – C(33)	121.4(3)
C(15) – C(14) – C(13)	121.7(3)	C(19) – C(39) – C(26)	110.4(2)
C(14) – C(15) – C(10)	120.6(3)	C(40) – C(39) – C(19)	121.2(2)
C(17) – C(16) – C(13)	120.9(2)	C(40) – C(39) – C(26)	128.3(2)
C(17) – C(16) – C(40)	118.9(2)	C(39) – C(40) – C(16)	119.3(2)
C(40) – C(16) – C(13)	120.1(2)	C(42) – C(41) – C(3)	120.0(3)
C(16) – C(17) – C(18)	121.9(2)	C(42) – C(41) – C(46)	118.8(3)
C(19) – C(18) – C(17)	118.6(2)	C(46) – C(41) – C(3)	121.2(3)
C(18) – C(19) – C(20)	131.1(2)	C(43) – C(42) – C(41)	120.5(3)
C(18) – C(19) – C(39)	120.0(2)	C(44) – C(43) – C(42)	119.8(3)
C(39) – C(19) – C(20)	108.8(2)	C(45) – C(44) – C(43)	120.5(3)
C(21) – C(20) – C(19)	131.0(3)	C(44) – C(45) – C(46)	120.5(3)
C(25) – C(20) – C(19)	108.6(2)	C(45) – C(46) – C(41)	119.8(3)
C(25) – C(20) – C(21)	120.3(2)		

5. Absorption and emission spectra of compounds 6a-j, 10, 11 and 12a,b

	In toluene	In MeCN
**6a**	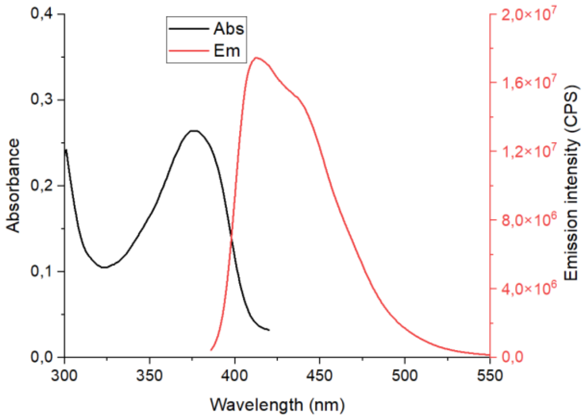	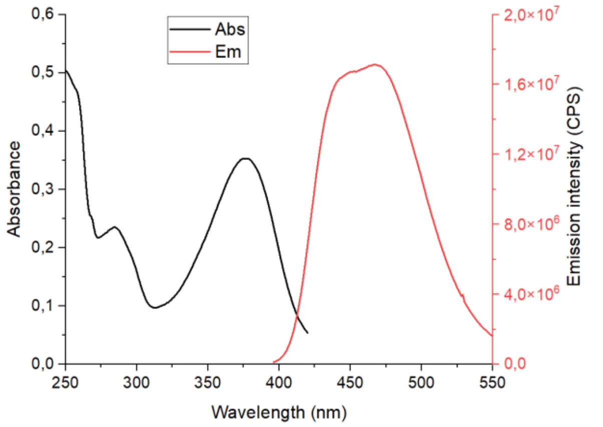
**6b**	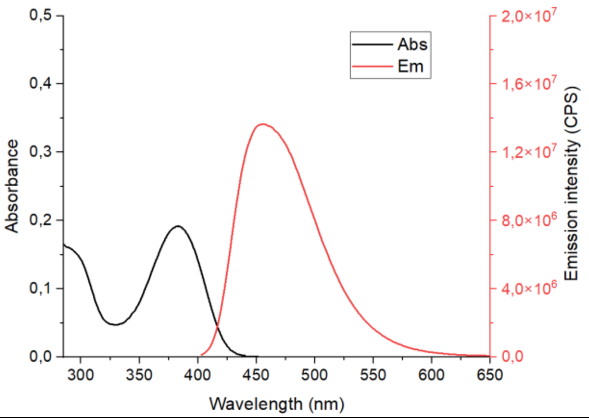	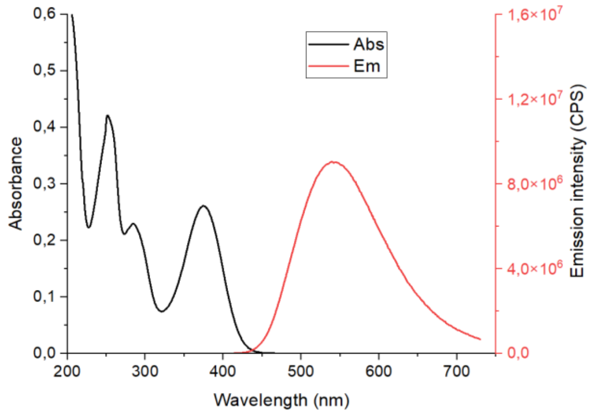
**6c**	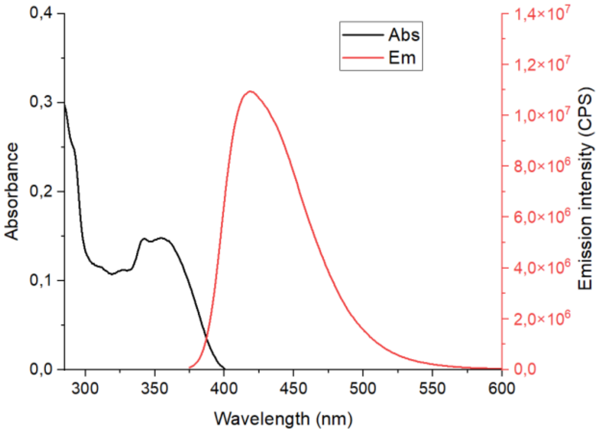	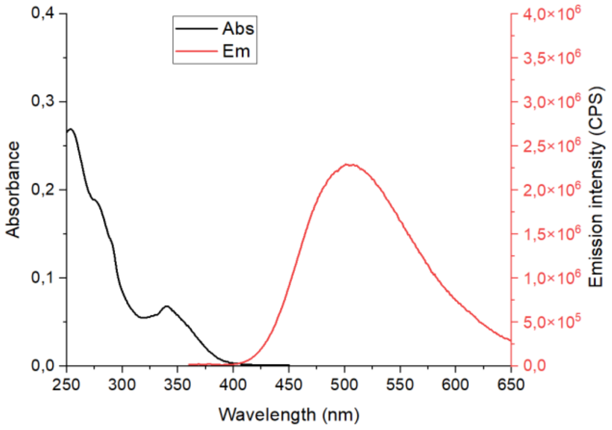

6d

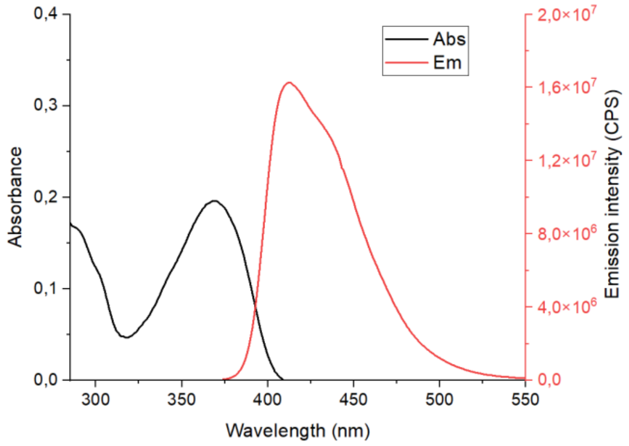
Abs
Em
Absorbance
0,0 0,1 0,2 0,3 0,4
Emission intensity (CPS)
0,0 $4,0\times10^6$ $8,0\times10^6$ $1,2\times10^7$ $1,6\times10^7$ $2,0\times10^7$
300 350 400 450 500 550
Wavelength (nm)

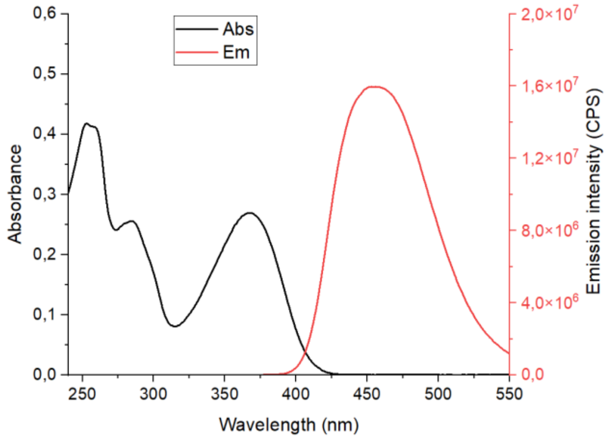
Abs
Em
Absorbance
0,0 0,1 0,2 0,3 0,4 0,5 0,6
Emission intensity (CPS)
0,0 $4,0\times10^6$ $8,0\times10^6$ $1,2\times10^7$ $1,6\times10^7$ $2,0\times10^7$
250 300 350 400 450 500 550
Wavelength (nm)

6e

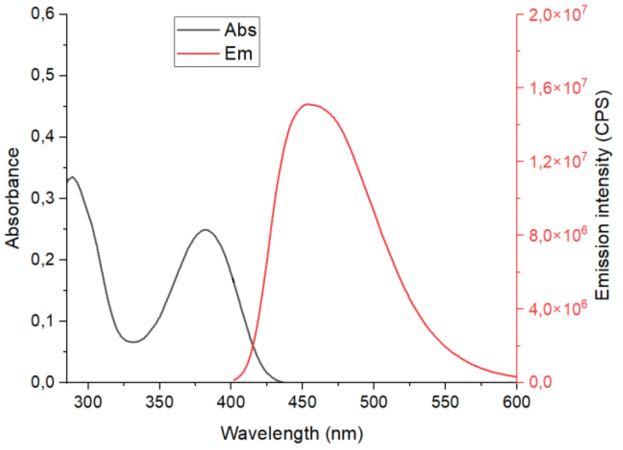
Abs
Em
Absorbance
0,0 0,1 0,2 0,3 0,4 0,5 0,6
Emission intensity (CPS)
0,0 $4,0\times10^6$ $8,0\times10^6$ $1,2\times10^7$ $1,6\times10^7$ $2,0\times10^7$
300 350 400 450 500 550 600
Wavelength (nm)

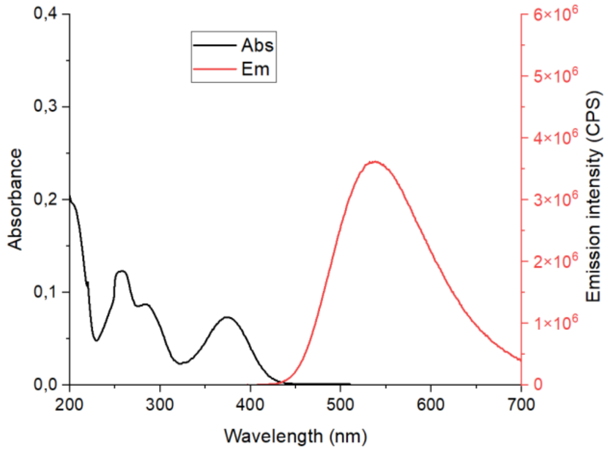
Abs
Em
Absorbance
0,0 0,1 0,2 0,3 0,4
Emission intensity (CPS)
0 1×10^6 2×10^6 3×10^6 4×10^6 5×10^6 6×10^6
200 300 400 500 600 700
Wavelength (nm)

6f

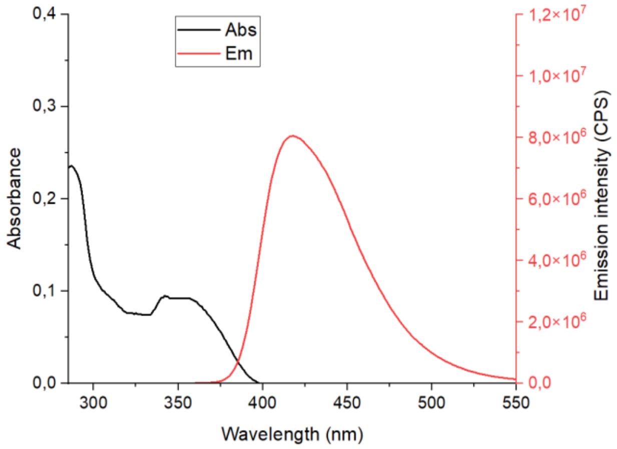
Abs
Em
Absorbance
0,0 0,1 0,2 0,3 0,4
Emission intensity (CPS)
0,0 $2,0\times10^6$ $4,0\times10^6$ $6,0\times10^6$ $8,0\times10^6$ $1,0\times10^7$ $1,2\times10^7$
300 350 400 450 500 550
Wavelength (nm)

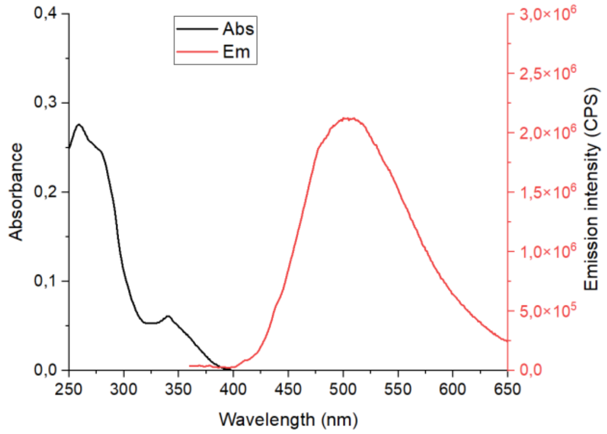
Abs
Em
Absorbance
0,0 0,1 0,2 0,3 0,4
Emission intensity (CPS)
0,0 $5,0\times10^5$ $1,0\times10^6$ $1,5\times10^6$ $2,0\times10^6$ $2,5\times10^6$ $3,0\times10^6$
250 300 350 400 450 500 550 600 650
Wavelength (nm)

6g

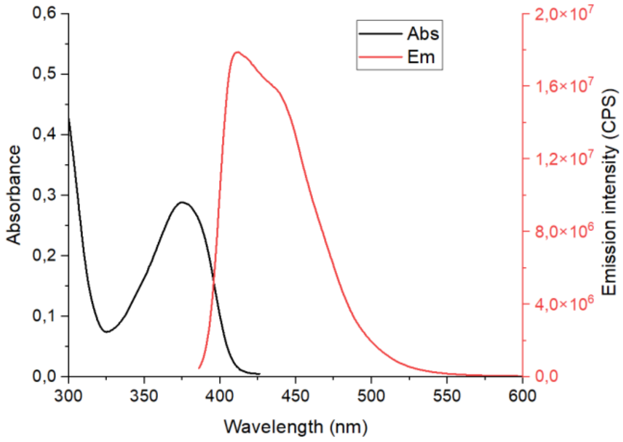
Abs
Em
Absorbance
0,0 0,1 0,2 0,3 0,4 0,5 0,6
Emission intensity (CPS)
0,0 $4,0\times10^6$ $8,0\times10^6$ $1,2\times10^7$ $1,6\times10^7$ $2,0\times10^7$
300 350 400 450 500 550 600
Wavelength (nm)

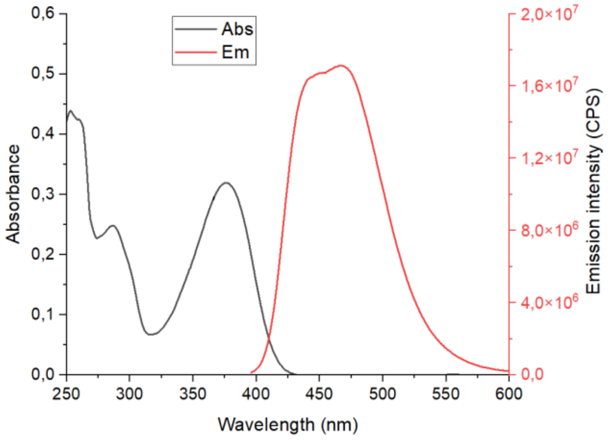
Abs
Em
Absorbance
0,0 0,1 0,2 0,3 0,4 0,5 0,6
Emission intensity (CPS)
0,0 $4,0\times10^6$ $8,0\times10^6$ $1,2\times10^7$ $1,6\times10^7$ $2,0\times10^7$
250 300 350 400 450 500 550 600
Wavelength (nm)

<table>
<tr><td></td><td></td><td></td></tr>
<tr><td>6h</td><td>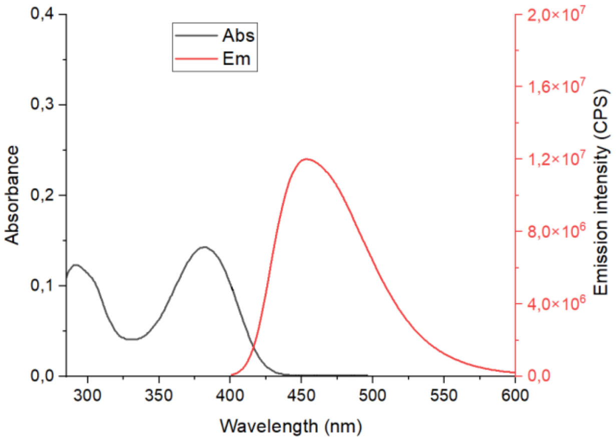
</td><td>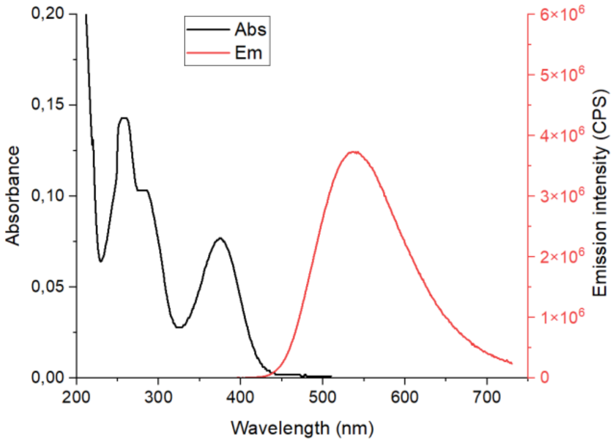
</td></tr>
<tr><td>6i</td><td>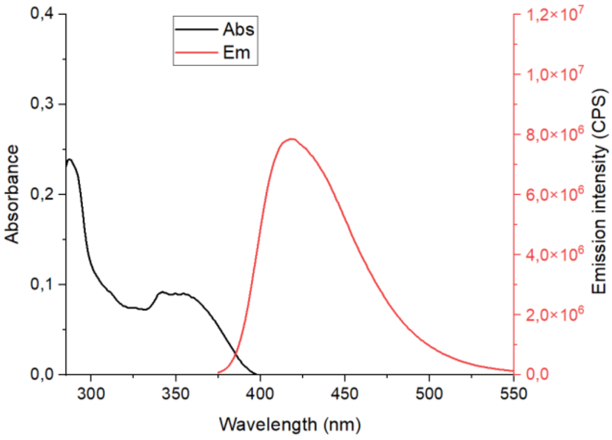
</td><td>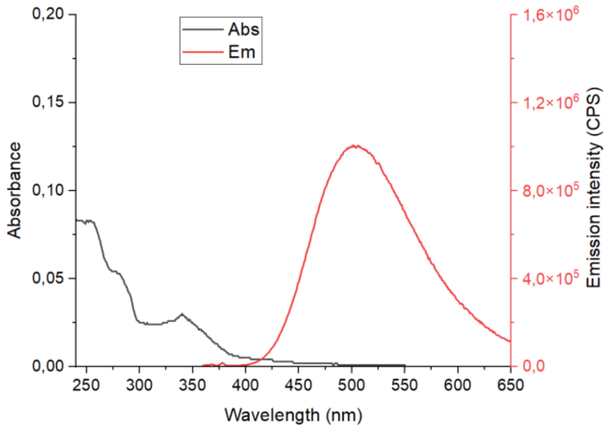
</td></tr>
<tr><td>6j</td><td>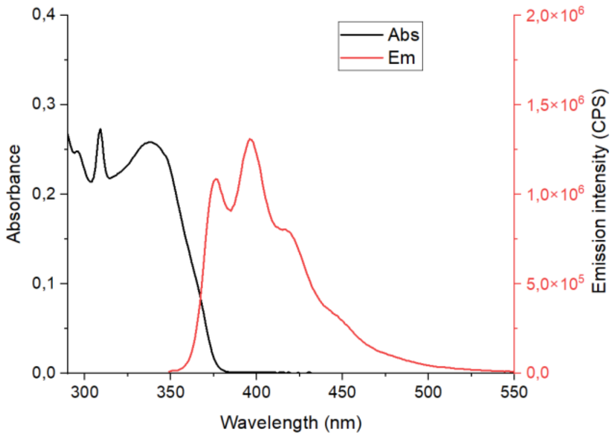
</td><td>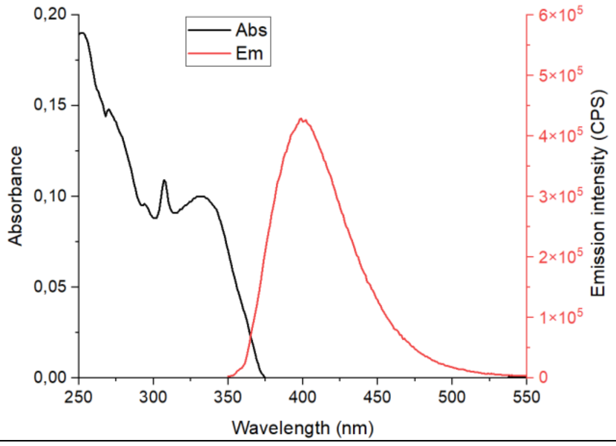
</td></tr>
<tr><td>10</td><td>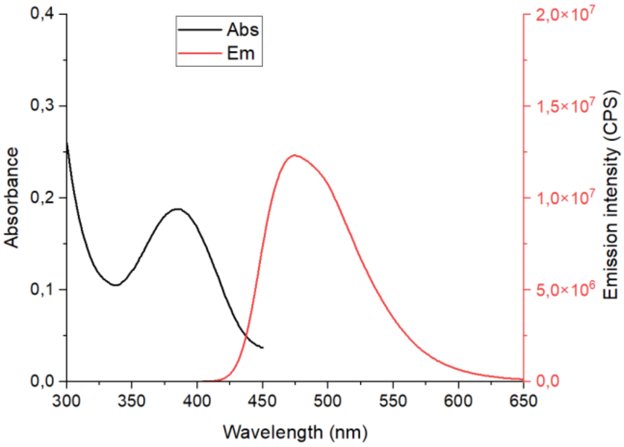
</td><td>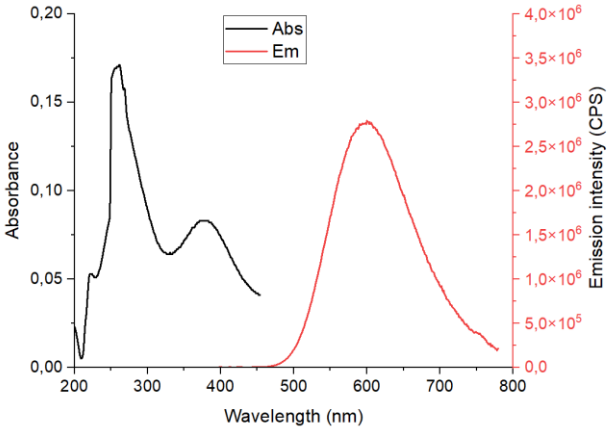
</td></tr>
</table>

11

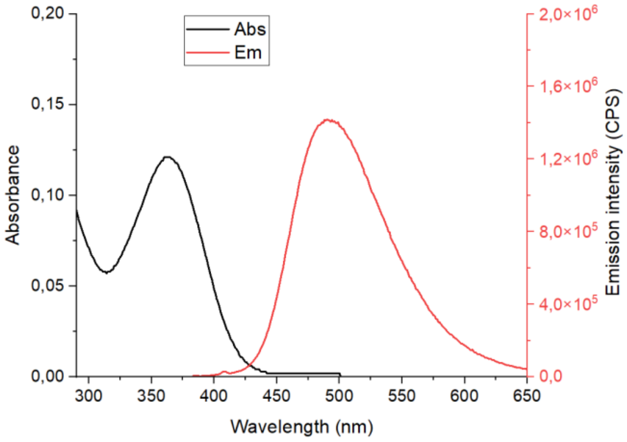

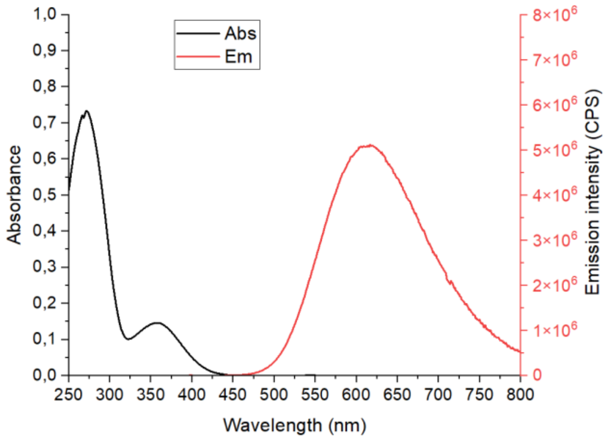

12a

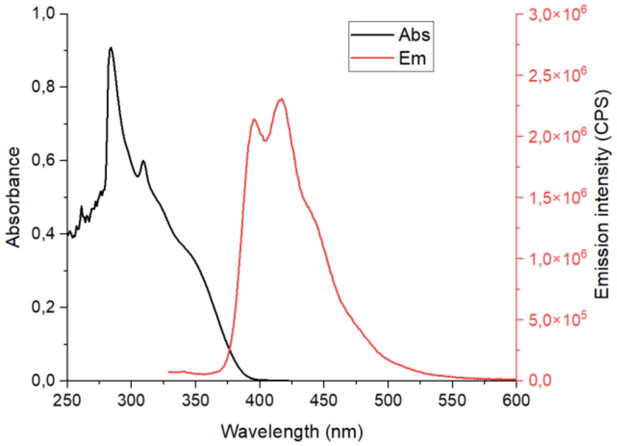

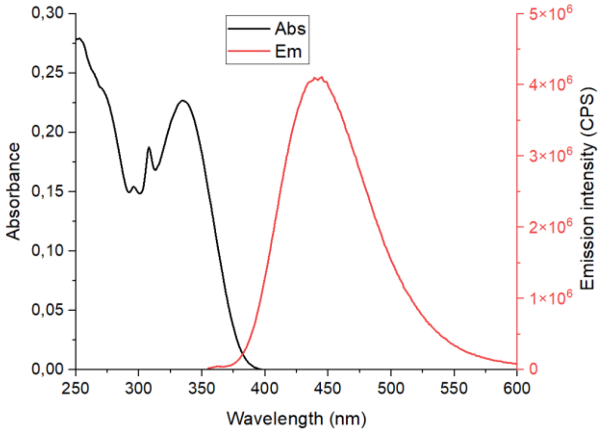

12b

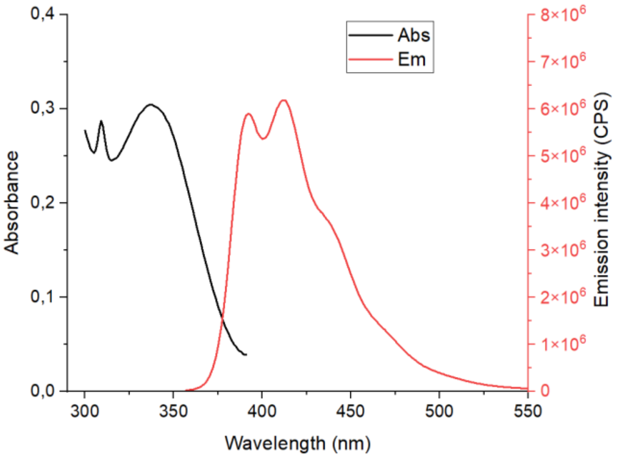

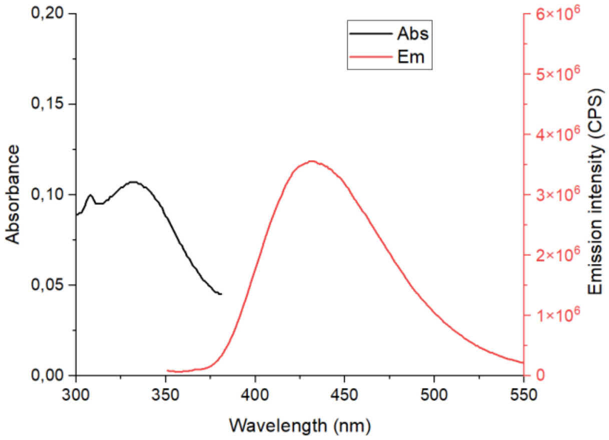

Figure S28. Absorption and emission spectra of compounds **6a-j, 10, 11** and **12a,b.**

Table S9. Photophysical properties of previously reported fluorophores of series **F** and **E**, and compounds **10** and **11**, in solution (c = 10^{-5} M) and the solid state at r.t.

Comp.		Solvent/ solid state	λ_{abs}, nm, ε (10^4 $M^{-1}cm^{-1}$)	λ_{em} [a], nm	$\Delta\nu_{st}$ [b], cm^{-1}	Φ_F [c], %
**Fa**	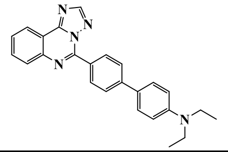	Toluene	383 (1.47)	479	5233	90
		MeCN	375 (3.01)	598	9944	34
		Solid	-	510	-	3
**Fb**	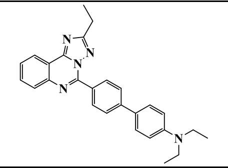	Toluene	380 (-)	486	5740	>98
		MeCN	374 (-)	579	9467	90
		Solid	-	517	-	8
**10**	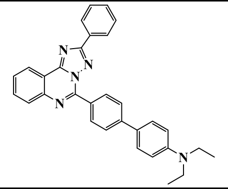	Toluene	384 (1.88)	481	5250	>95
		MeCN	377 (0.83)	601	9890	35
		Solid	-	513	-	6
**Ea**	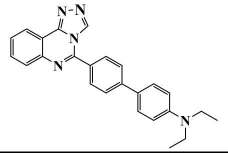	Toluene	371 (1.93)	476	5946	90
		MeCN	366 (2.41)	605	10,793	29
		Solid	-	500	-	8
**Eb**	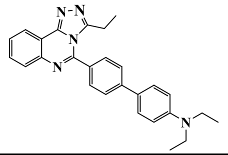	Toluene	343 (2.82)	481	8364	16
		MeCN	340 (4.26)	608	12,964	41
		Solid	-	455	-	32
**11**	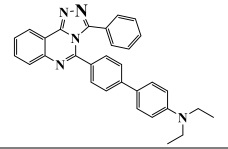	Toluene	363 (1.21)	493	8324	17
		MeCN	356 (1.46)	616	11860	34
		Solid	-	492	-	*

[a] Excitation at the lowest energy absorption peak. [b] Stokes shift was calculated relative to the lowest energy absorption peak and rounded to tens. [c] Absolute fluorescence quantum yield was measured according to a reported procedure [3] using Horiba-Fluoromax-4 spectrofluorometer equipped with integrating sphere. [e]Relative to middle peak of emission. * Data was not measured.

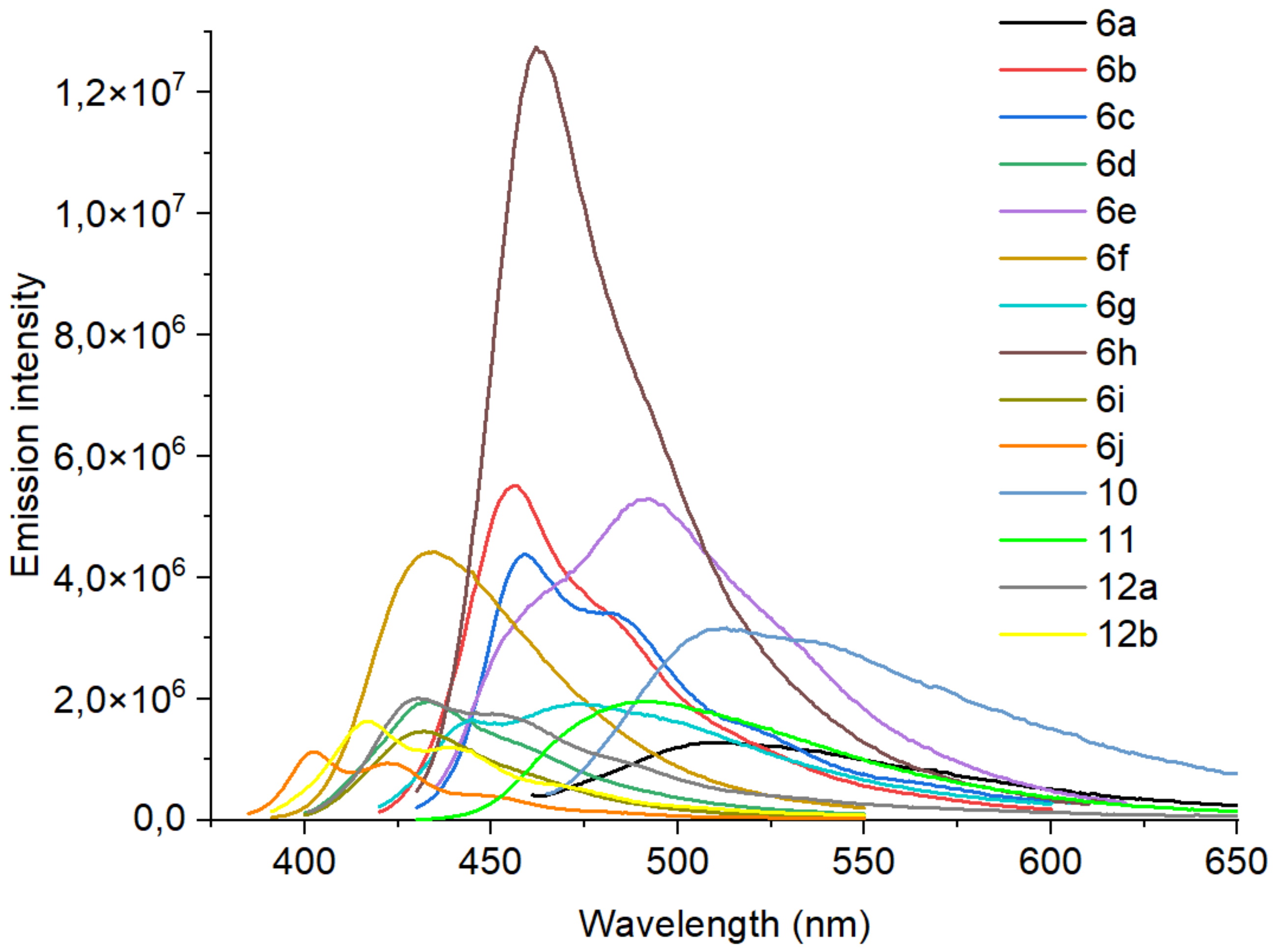

Figure S29. Combined solid-state emission spectra of compounds **6a-j, 10, 11** and **12a,b**

6. Solvatochromic properties of compounds 6, 10 and 11

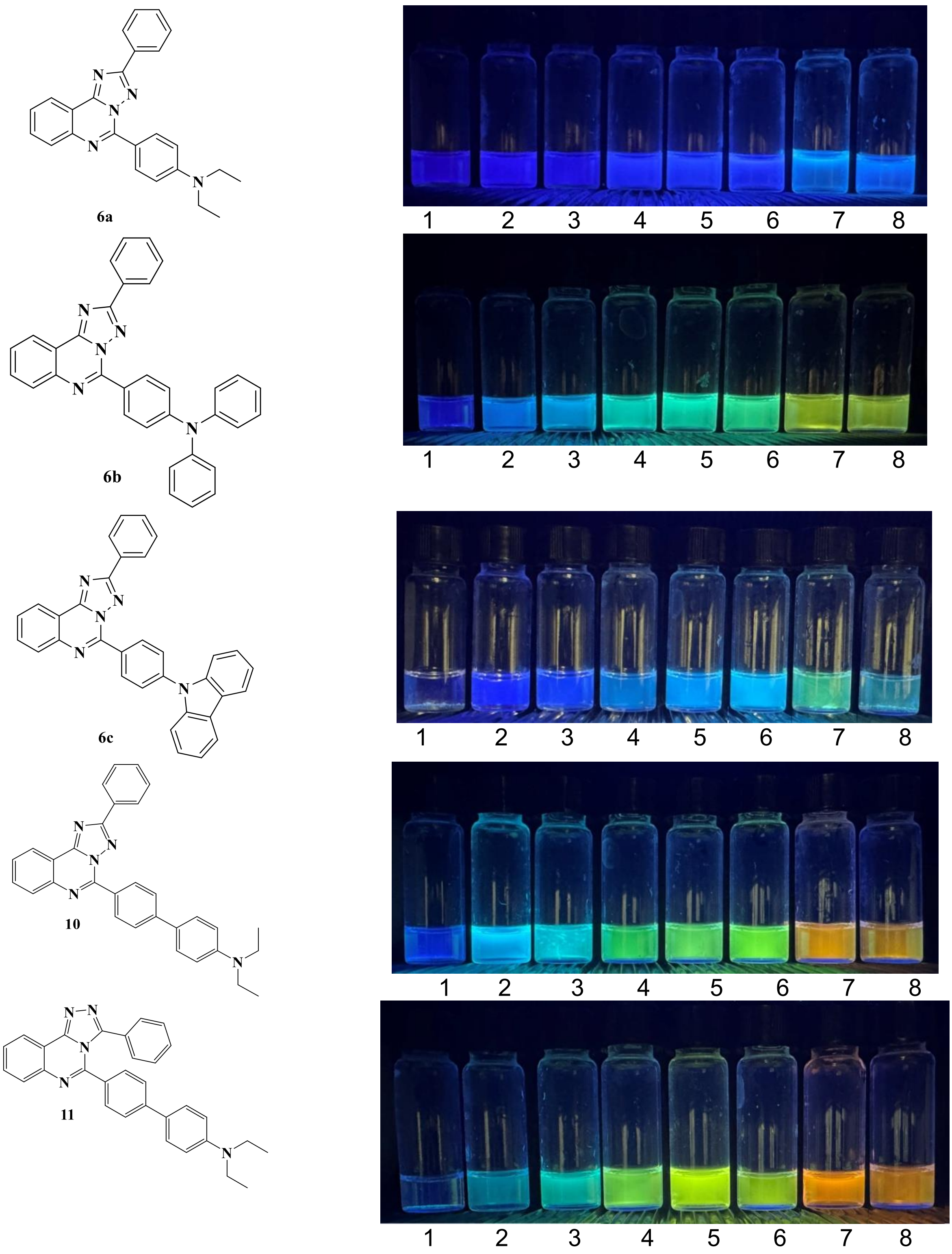

Figure S30. Changes in emission color of compounds **6a-c**, **10** and **11** in different solvents (1 – cyclohexane, 2 – toluene, 3 – 1,4-dioxane, 4 – EtOAc, 5 – THF, 6 – CH_2Cl_2, 7 – DMSO, 8 – MeCN).

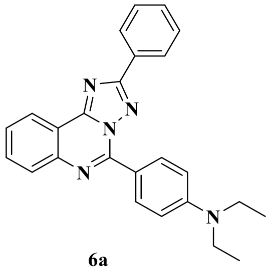

6a

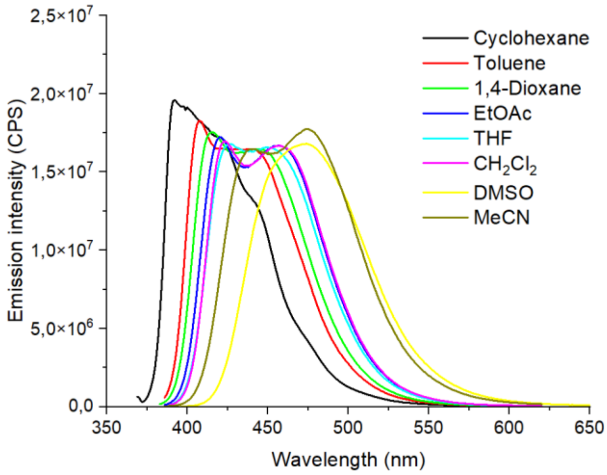

Emission intensity (CPS)
Wavelength (nm)
Cyclohexane
Toluene
1,4-Dioxane
EtOAc
THF
CH_2Cl_2
DMSO
MeCN

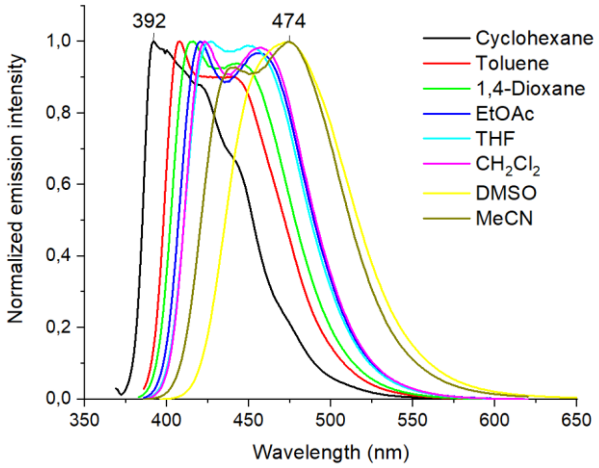

392
474
Normalized emission intensity
Wavelength (nm)
Cyclohexane
Toluene
1,4-Dioxane
EtOAc
THF
CH_2Cl_2
DMSO
MeCN

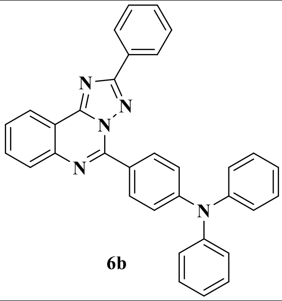

6b

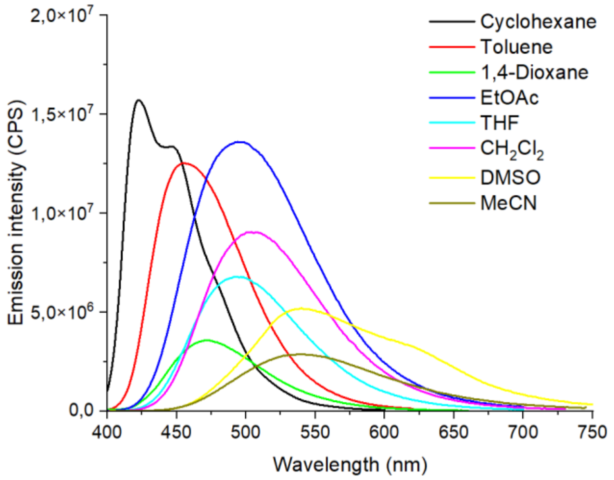

Emission intensity (CPS)
Wavelength (nm)
Cyclohexane
Toluene
1,4-Dioxane
EtOAc
THF
CH_2Cl_2
DMSO
MeCN

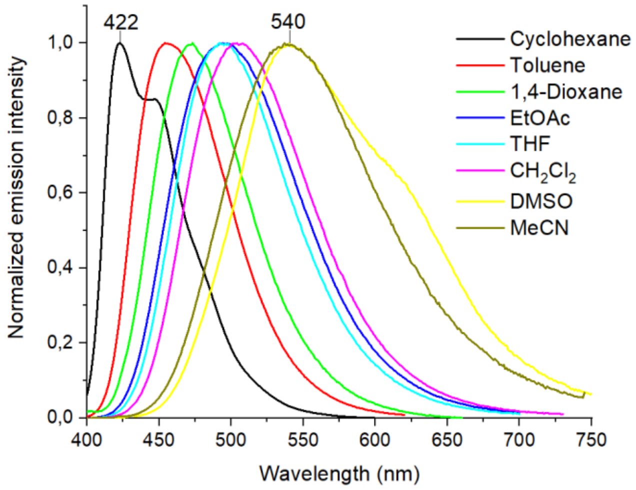

422
540
Normalized emission intensity
Wavelength (nm)
Cyclohexane
Toluene
1,4-Dioxane
EtOAc
THF
CH_2Cl_2
DMSO
MeCN

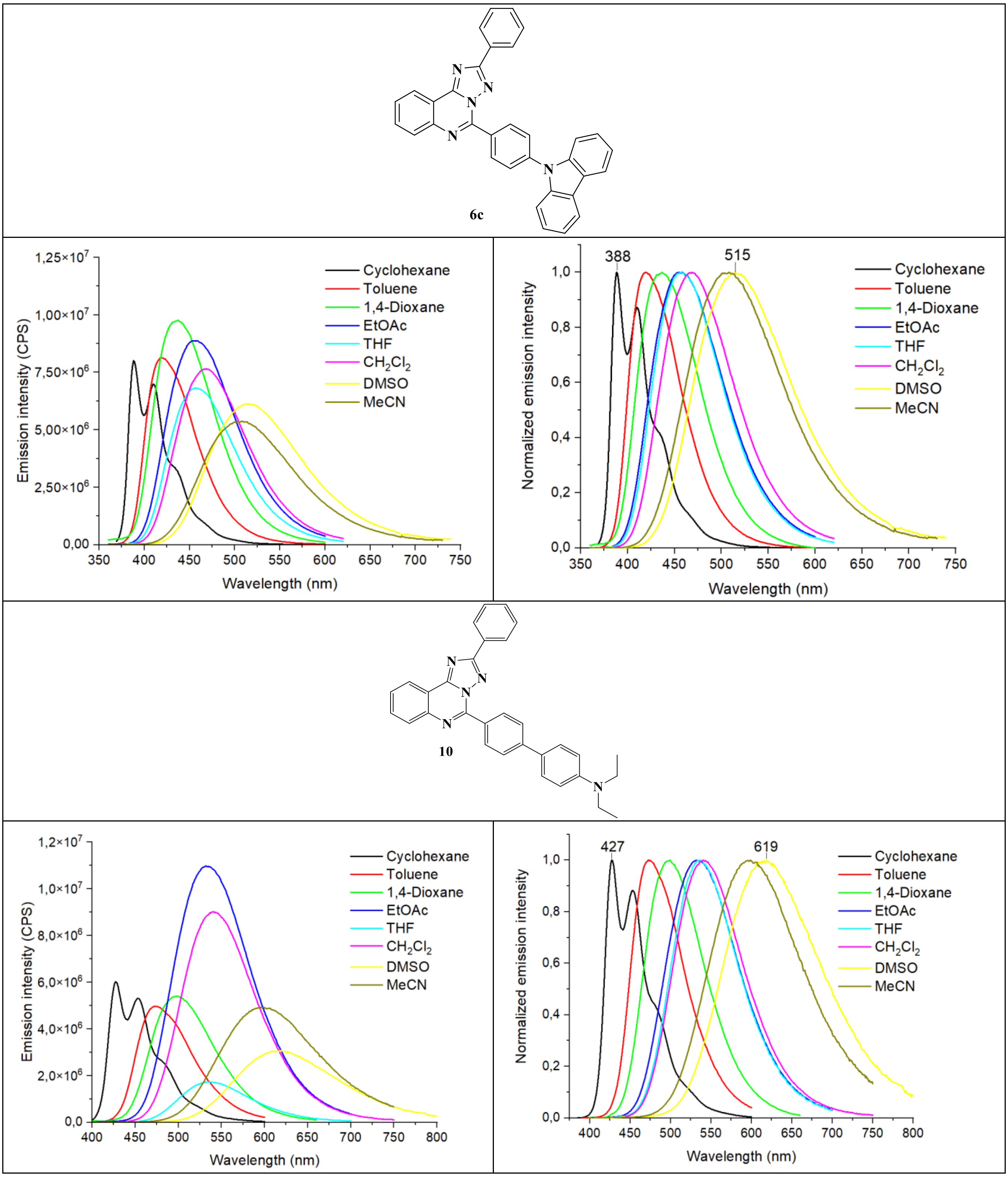

Figure S31. Combined emission spectrum (non-normalized and normalized) of compounds **6a**, **6b**, **6c** and **10** in different solvents.

Table S10. Empirical solvent polarity parameter ($E^N{}_T$), absorption and emission maxima (λ_{abs}, λ_{em}, nm) and Stokes shift (nm, cm^{-1}) of **6b** in different solvents.

**Solvent**	$E^N{}_T$	λ_{abs}, [nm]	λ_{em}, [nm]	$\Delta\nu_{st}$, [cm^{-1}]
Cyclohexane	0.01	382	446	3756
Toluene	0.10	383	459	4323
1,4-Dioxane	0.16	378	473	5313
EtOAc	0.23	379	494	6142
THF	0.21	381	492	5922
CH_2Cl_2	0.31	381	504	6405
DMSO	0.44	382	540	7659
MeCN	0.46	375	544	8284

[a] the red-shifted maxima was used for calculation (in bold).

Table S11. Empirical solvent polarity parameter ($E^N{}_T$), absorption and emission maxima (λ_{abs}, λ_{em}, nm) and Stokes shift (nm, cm^{-1}) of **6c** in different solvents.

**Solvent**	$E^N{}_T$	λ_{abs}, [nm]	λ_{em}, [nm]	$\Delta\nu_{st}$, [cm^{-1}]
Cyclohexane	0.01	359	410	3465
Toluene	0.10	355	423	4528
1,4-Dioxane	0.16	340	437	6528
EtOAc	0.23	340	455	7434
THF	0.21	341	456	7396
CH_2Cl_2	0.31	342	469	7918
DMSO	0.44	342	515	9822
MeCN	0.46	340	507	9688

[a] the red-shifted maxima was used for calculation (in bold).

Table S12. Empirical solvent polarity parameter ($E^N{}_T$), absorption and emission maxima (λ_{abs}, λ_{em}, nm) and Stokes shift (nm, cm^{-1}) of **10** in different solvents.

**Solvent**	$E^N{}_T$	λ_{abs}, [nm]	λ_{em}, [nm]	$\Delta\nu_{st}$, [cm^{-1}]
Cyclohexane	0.01	374	**427**, 454	4712
Toluene	0.10	384	481	5252
1,4-Dioxane	0.16	380	499	6276
EtOAc	0.23	380	532	7519
THF	0.21	386	536	7250
CH_2Cl_2	0.31	384	540	7523
DMSO	0.44	390	619	9486
MeCN	0.46	377	601	9886

[a] the red-shifted maxima was used for calculation (in bold).

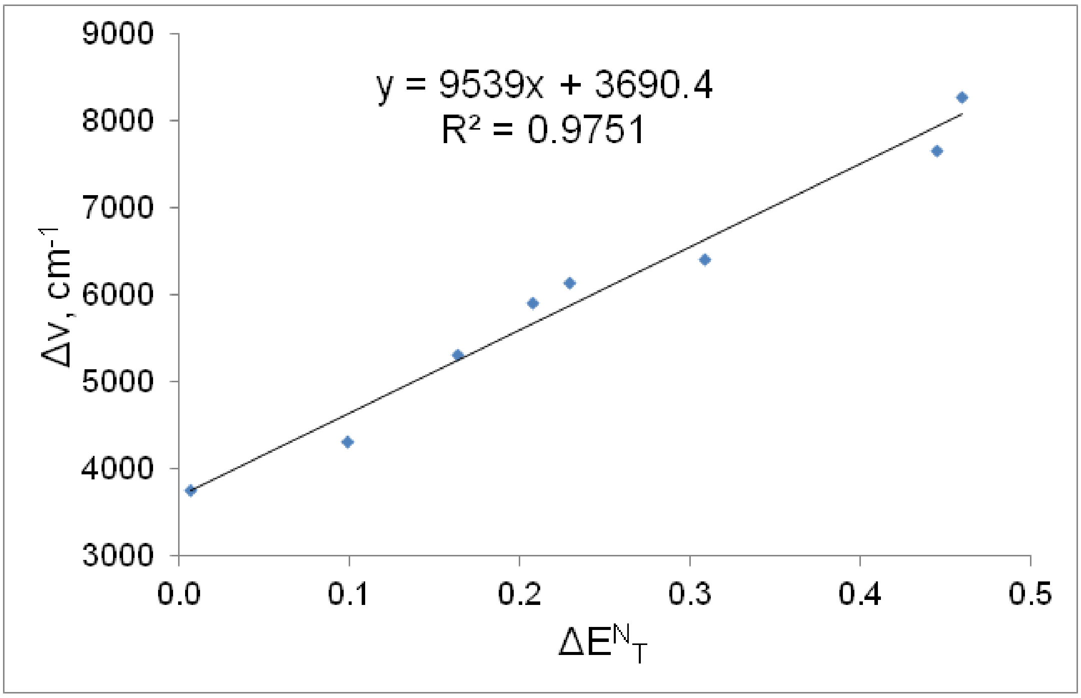
y = 9539x + 3690.4
R² = 0.9751
9000
8000
7000
6000
5000
4000
3000
Δv, cm-1
0.0
0.1
0.2
0.3
0.4
0.5
ΔENT

a

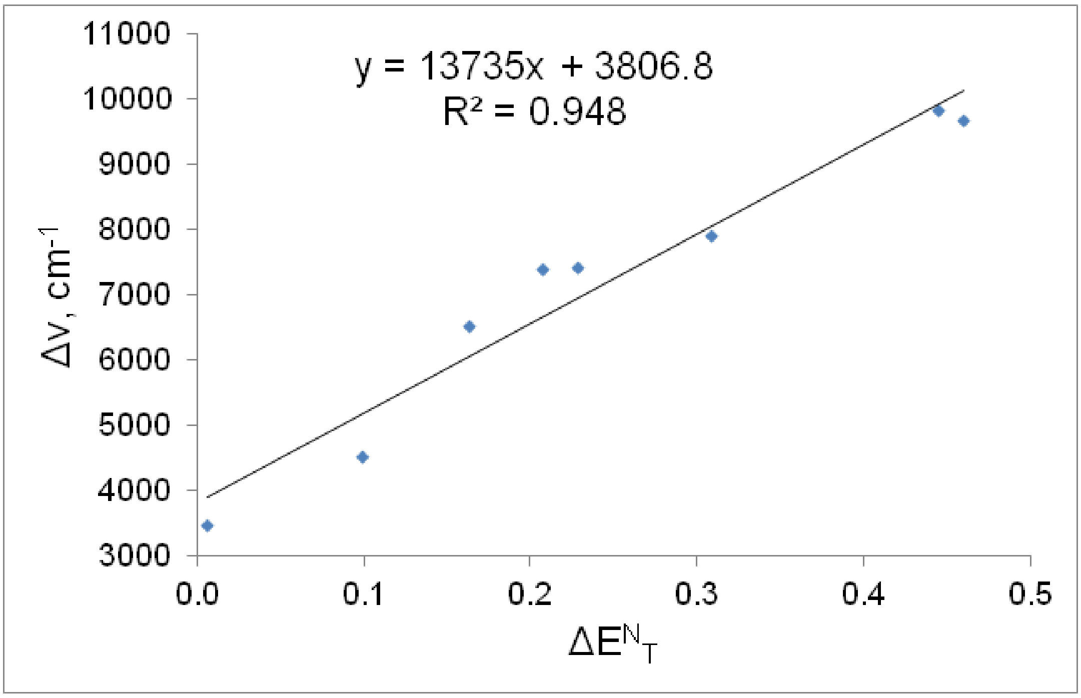
y = 13735x + 3806.8
R² = 0.948
11000
10000
9000
8000
7000
6000
5000
4000
3000
Δv, cm-1
0.0
0.1
0.2
0.3
0.4
0.5
ΔENT

b

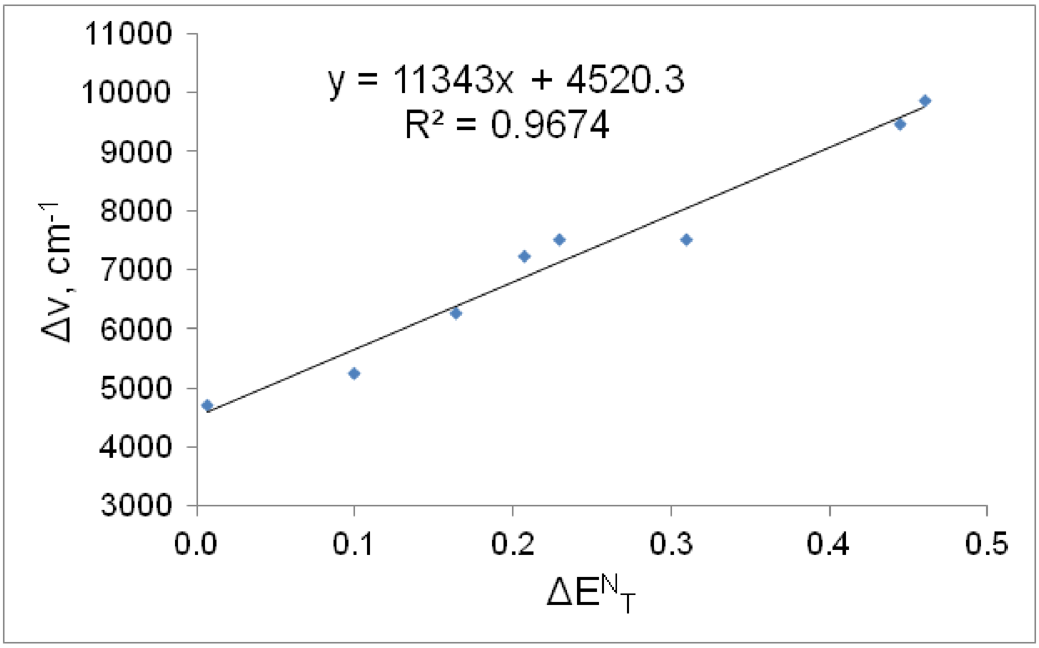

c

Figure S32. Plots of Stokes shift of compounds **6b** (a), **6c** (b) and **10** (c) versus Reichardt solvent polarity functions.

Estimation of Ground–Excited State Dipole Moment Difference by the Ravi Method [4].

The method relies on the empirical solvent polarity scale proposed by Reichardt [5]. In accordance with the method, the linear correlation is written as

$$v_a - v_f = 11307.6 \times E_T^N \left(\frac{\Delta\mu}{\Delta\mu_B}\right)^2 \left(\frac{a_B}{a}\right)^3 + \text{constant}, \quad (1)$$

In equation (1) $v_a - v_f$ is the Stokes shift (in cm^{-1}), E_T^N is the empirical solvent polarity parameter (Reichardt's normalized $E_T(30)$ scale) [5], $\Delta\mu$ and $\Delta\mu_B$ – the difference between the dipole moments of the ground and excited states for the target fluorophore and for the standard betaine dye ($\text{in D}, \Delta\mu_B = 9\text{D}$ [6]), a and a_B the Onsager cavity radius of the fluorophore and of the standard betaine dye ($\text{in Å}, a_B = 6.2\text{Å}$ [5]).

The slope coefficient (m) in the plot of $v_a - v_f$ *versus* E_T^N is:

$$\text{m} = 11307.6 \left(\frac{\Delta\mu}{\Delta\mu_B}\right)^2 \left(\frac{a_B}{a}\right)^3 \quad (2)$$

Where:

$$\Delta\mu = \sqrt{\frac{m \times 81}{11307.6 \times \left(\frac{6.2}{a}\right)^3}} \quad (3)$$

7. Acid induced spectroscopic changes of compounds 6a-c, 7 and 8

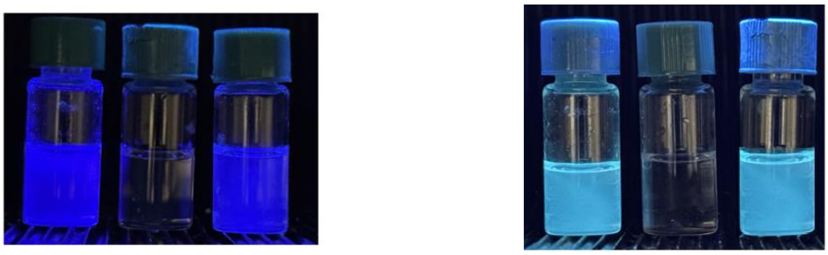

6a +TFA +TEA **10** +TFA +TEA

Figure S33. Emission color changes of compounds **6a** and **10** in toluene upon sequential addition of trifluoroacetic acid (TFA) and triethylamine (TEA).

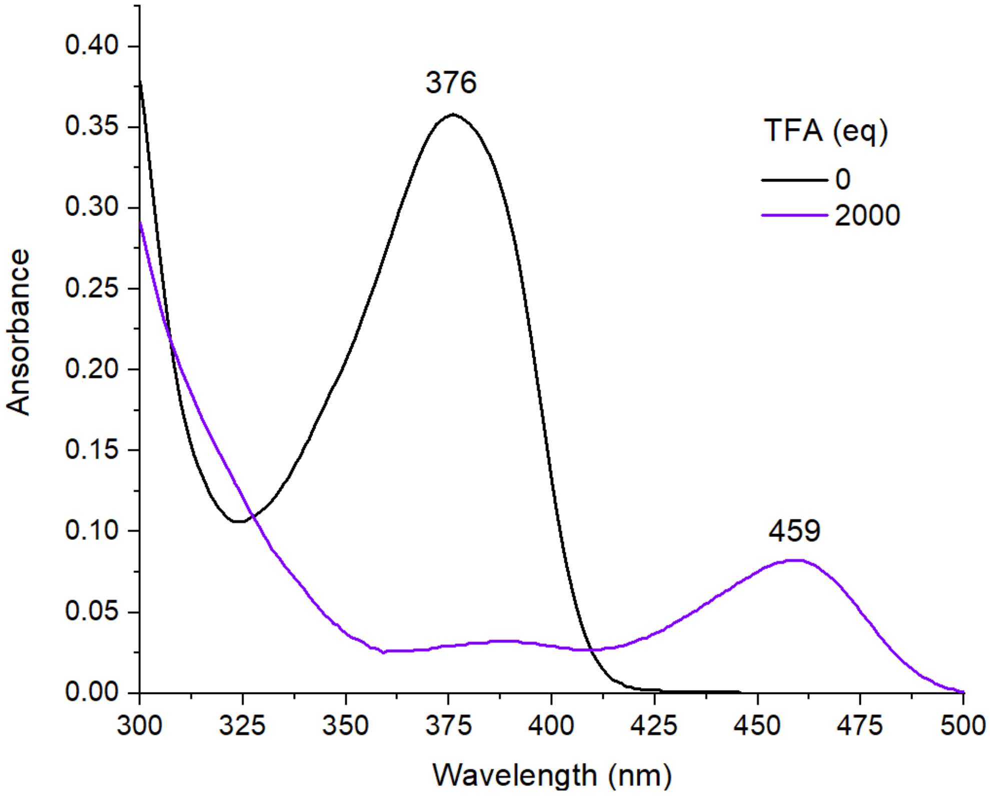

a

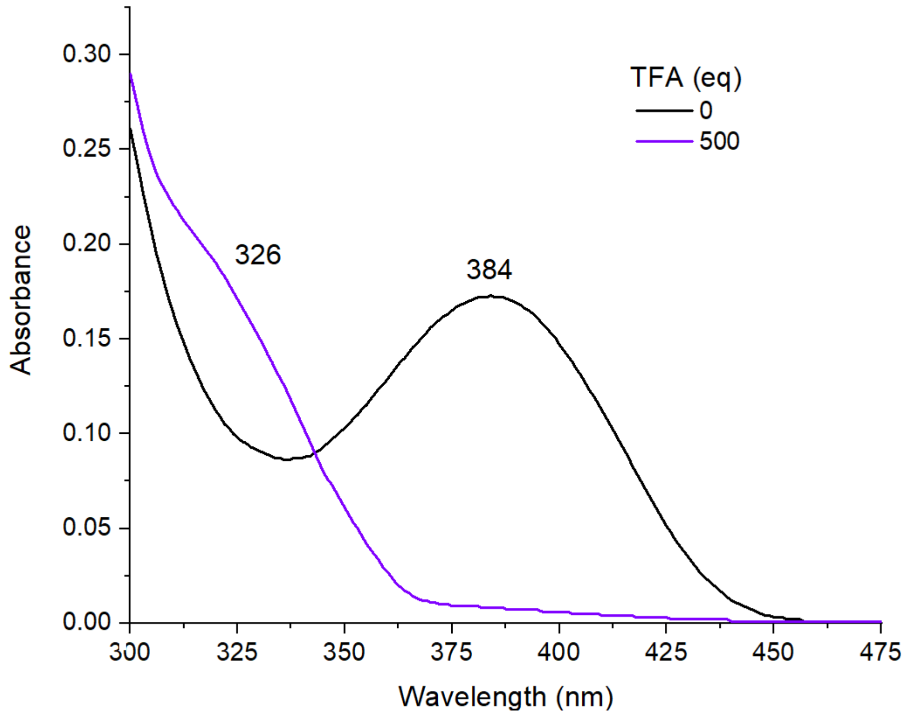

b

Figure S34. Absorption spectra of compounds **6a** (a) and **10** (b) in pure toluene (C = 2×10^{-6} M) and upon addition of excess of **CF_3COOH (TFA)**.

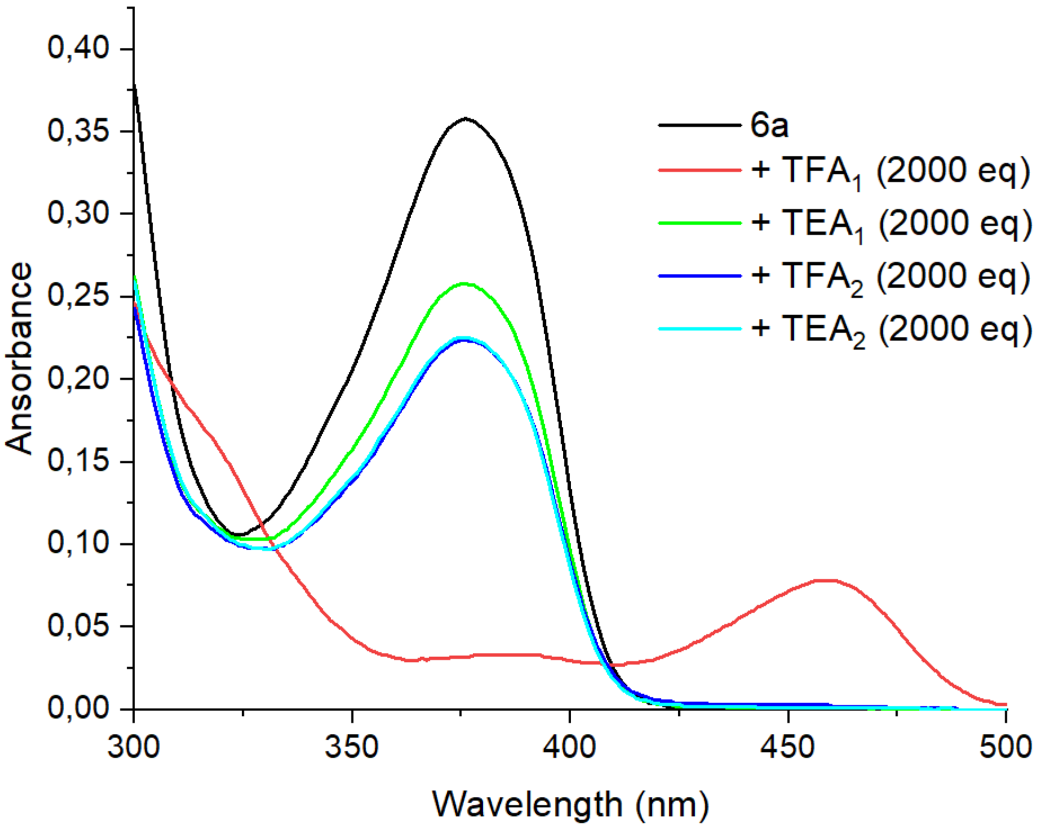

a

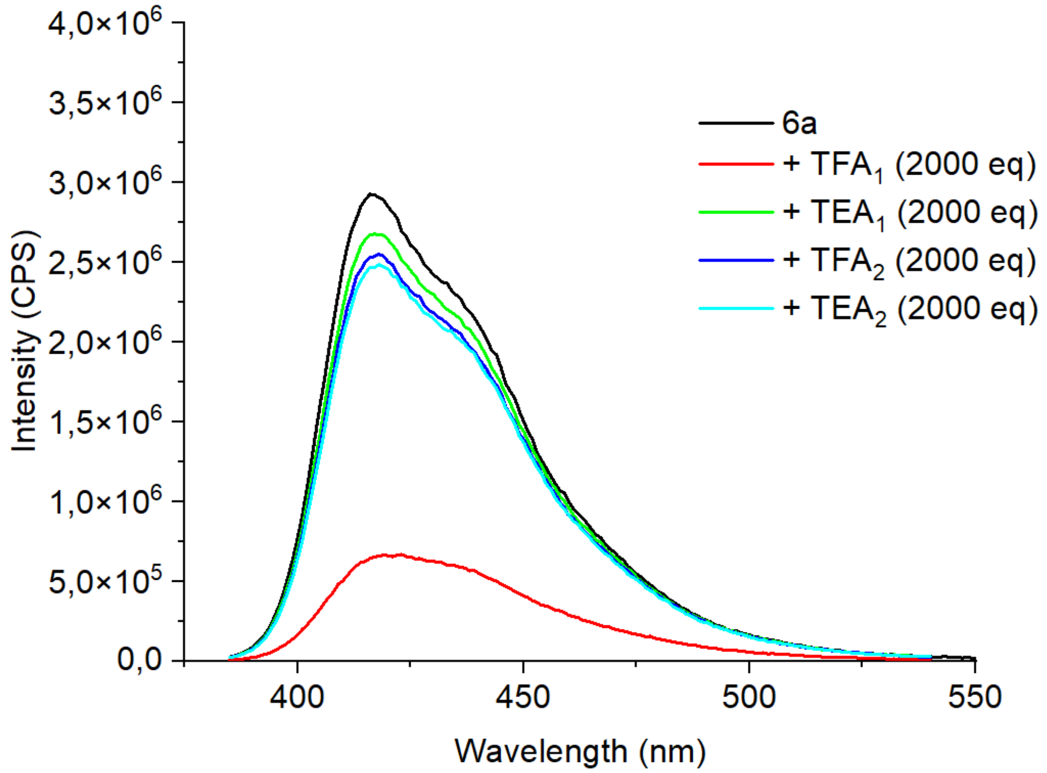

b

Figure S35. Absorption (a) and emission (b) spectra changes of **6a** in toluene (C = 2×10^{-6} M) upon sequential addition of **CF_3COOH (TFA)** and **Et_3N** (**TEA**).

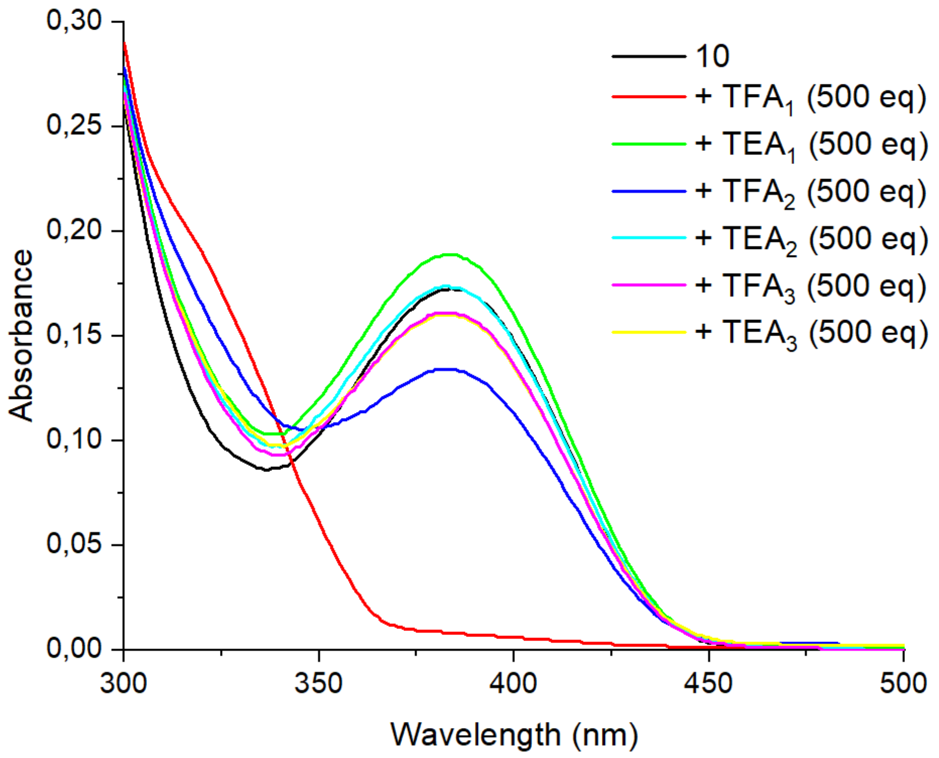

a

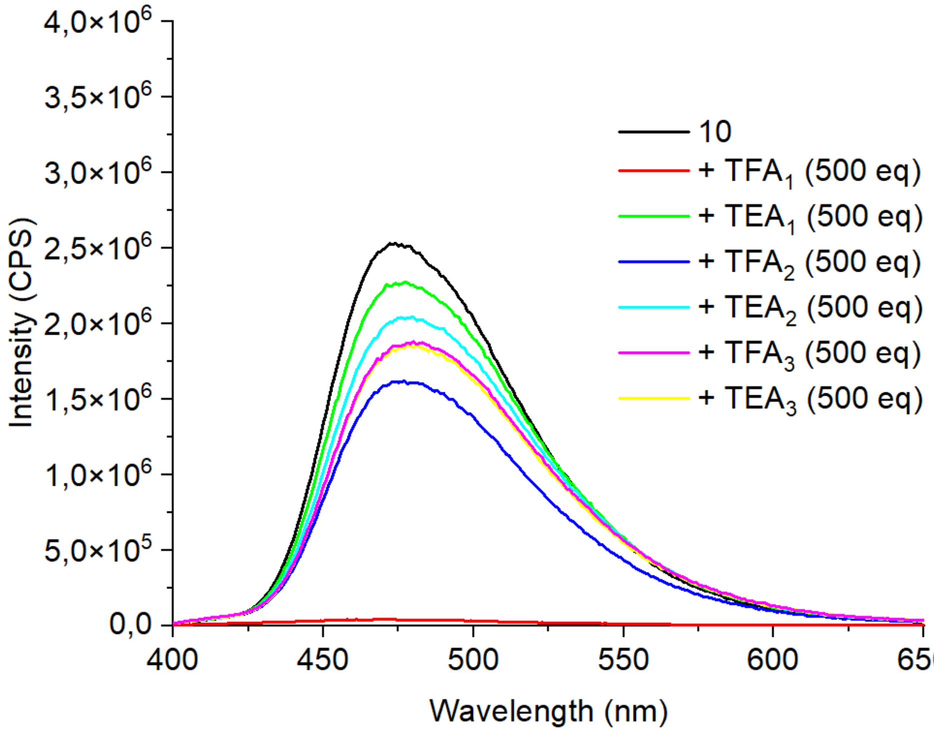

b

Figure S36. Absorption (a) and emission (b) spectra changes of **6a** in toluene (C = 2×10^{-6} M) upon sequential addition of **CF_3COOH (TFA)** and **Et_3N** (**TEA)**.

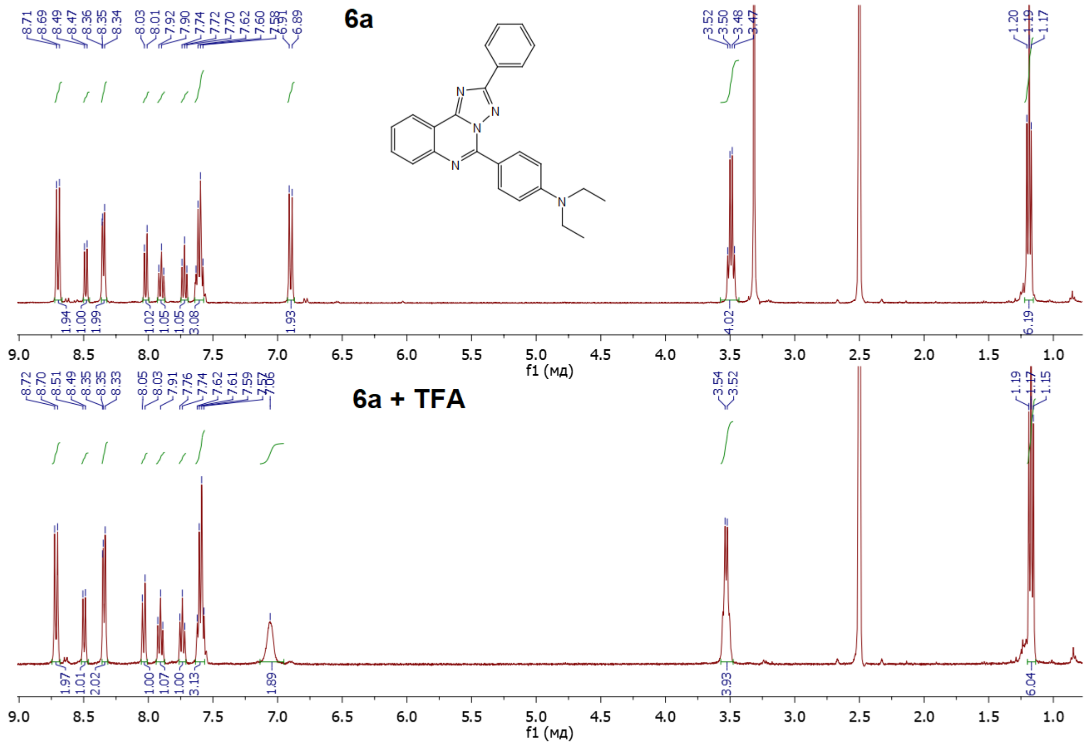

a

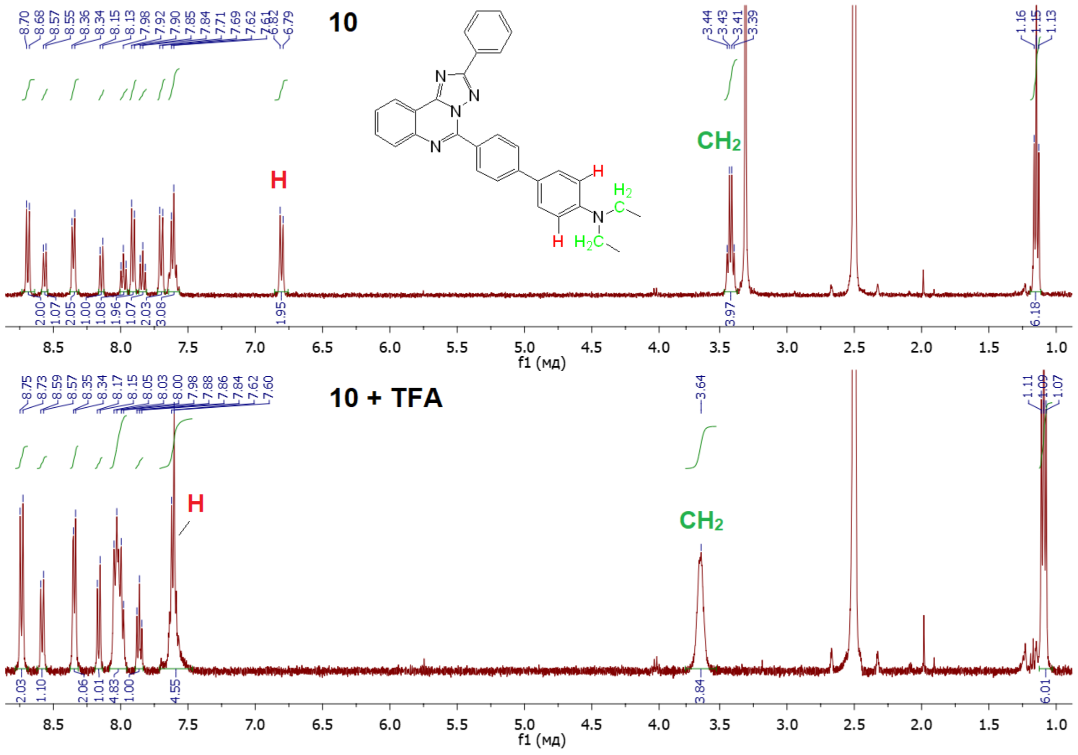

b

Figure S37. ^{1}H NMR spectra of compounds **6a** (a) and **10** (b) in pure DMSO-d_6 and upon addition of excess of TFA-d_1.

8. Theoretical studies

Table S13. The electronic distribution in HOMO/LUMO of **6a-c,e,h,j**, **10**, **11** and **12a,b** for gas phase.

Model structure	HOMO, eV	LUMO, eV	Eg
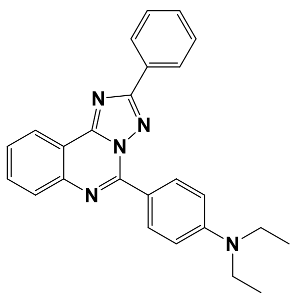 **6a**	-5.23 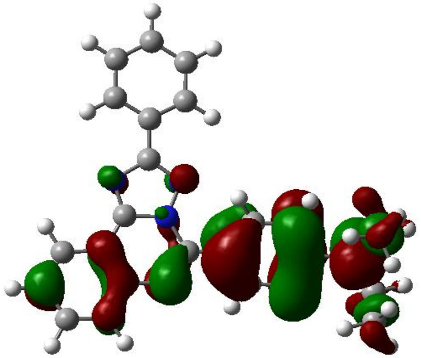	-1.48 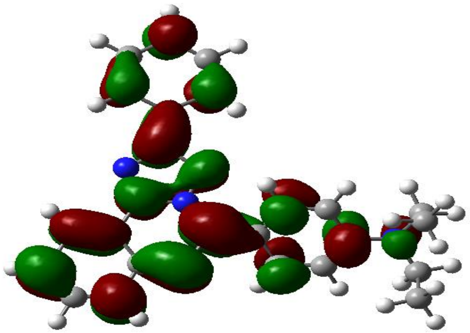	3.75
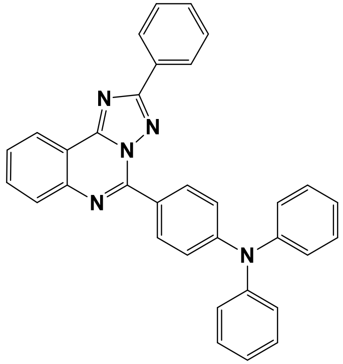 **6b**	-5.09 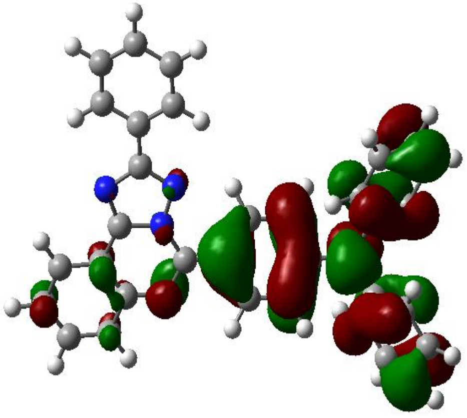	-1.63 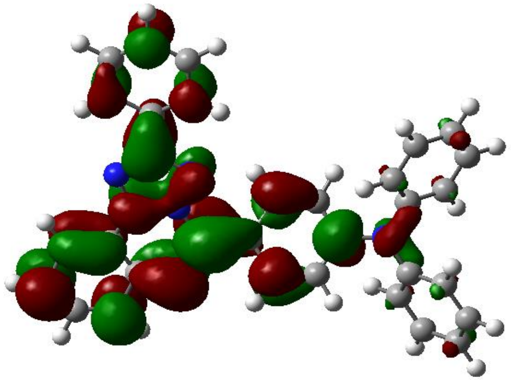	3.46

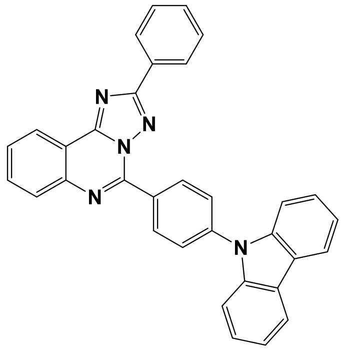 **6c**	-5.42 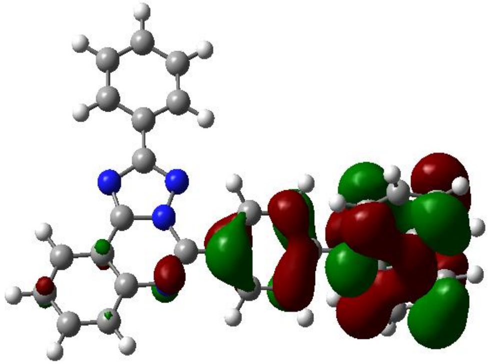	-1.84 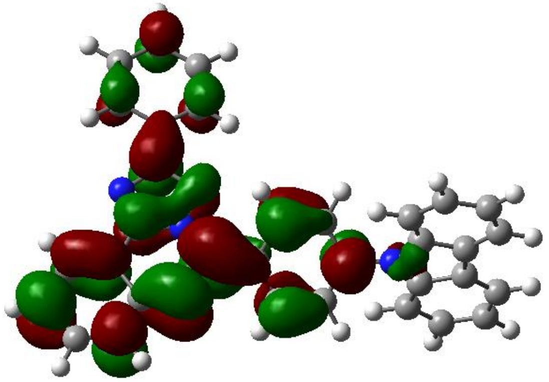	3.58
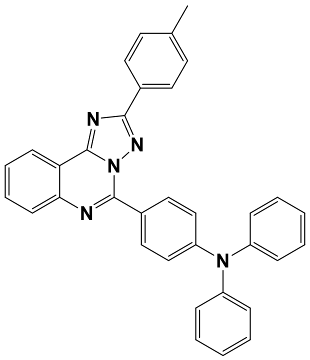 **6d**	-5.07 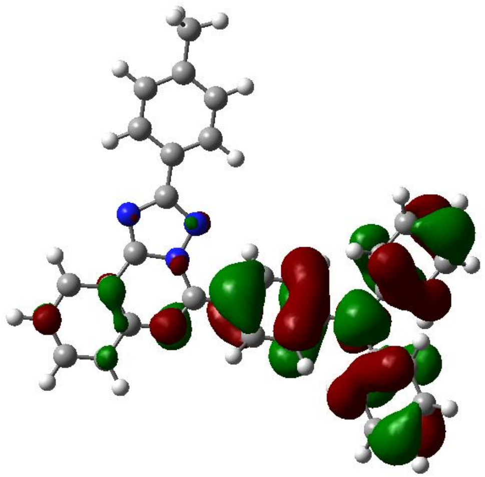	-1.59 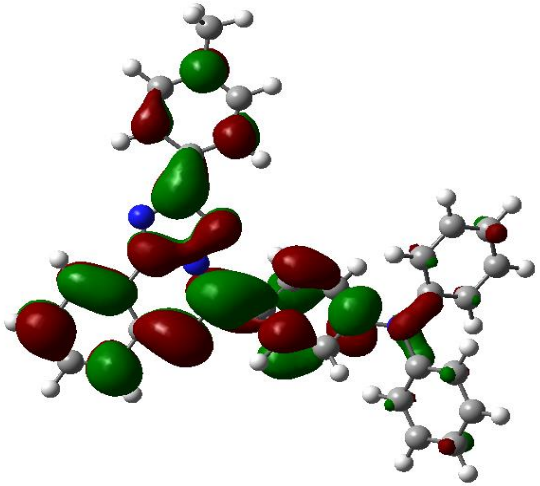	3.48

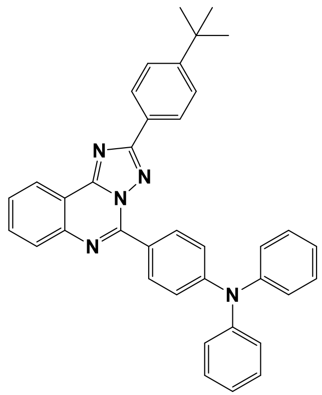 **6g**	-5.07 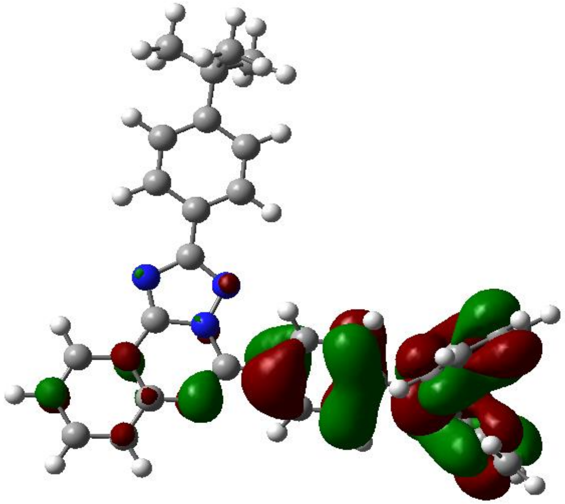	-1.58 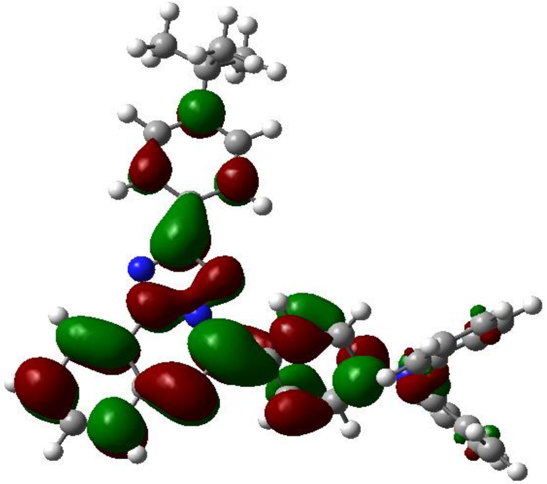	3.48
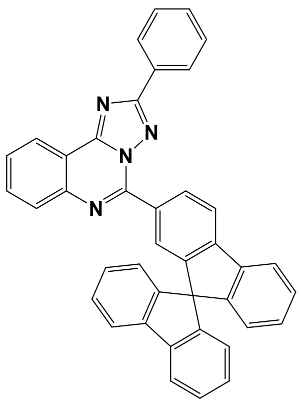 **6k**	-5.68 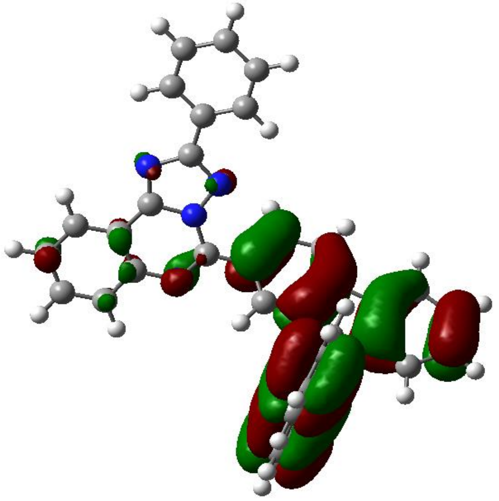	-1.75 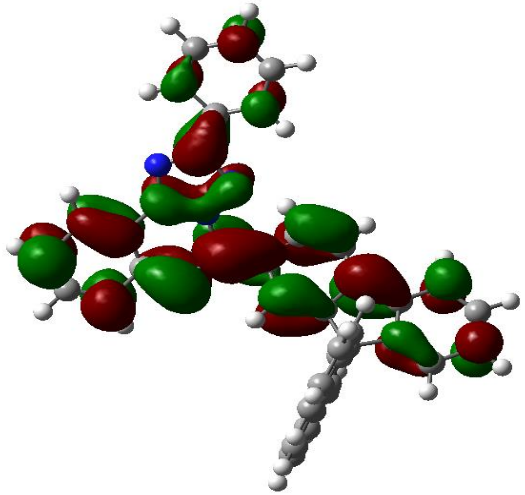	3.93

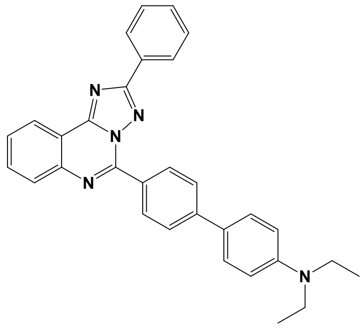 **10**	-4.92 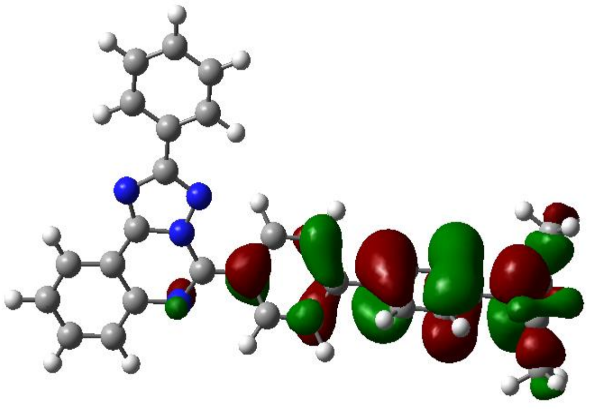	-1.60 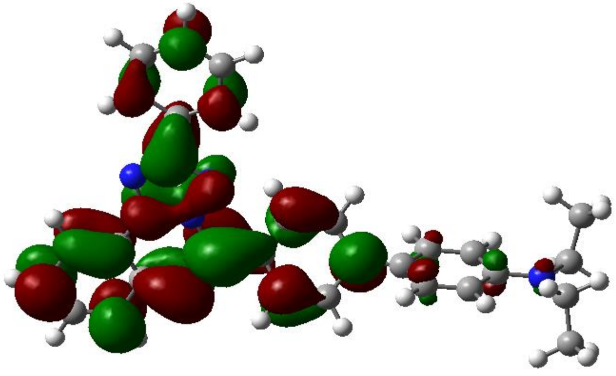	3.32
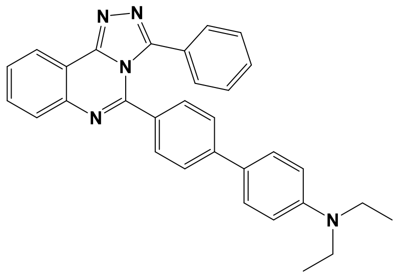 **11**	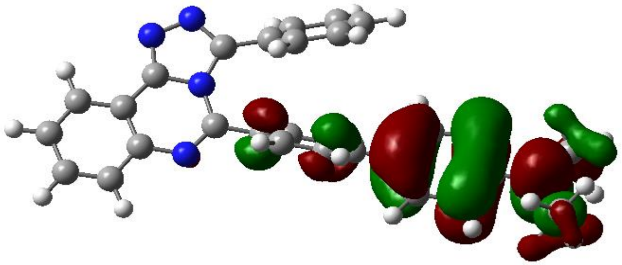 -5.01	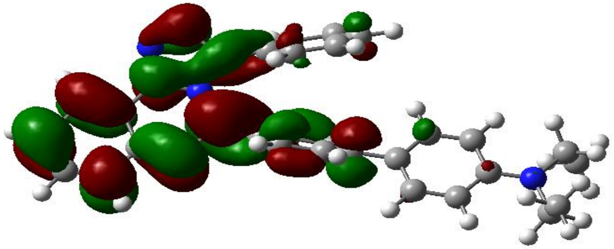 -1.47	3.54

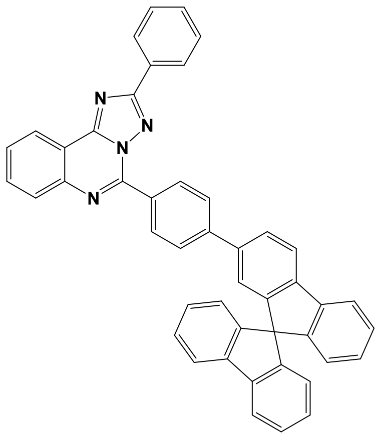 **12a**	-5.62 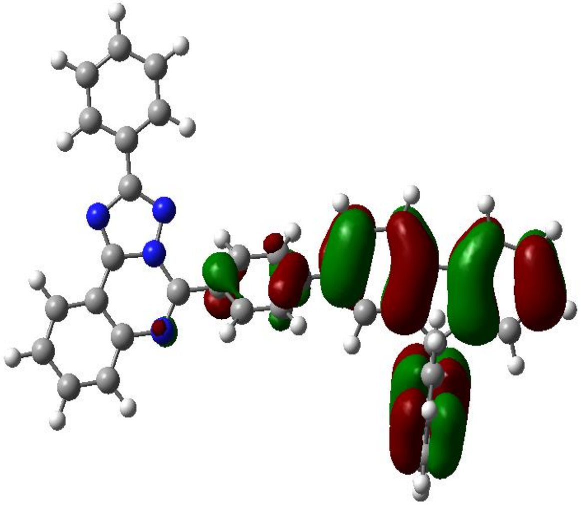	-1.73 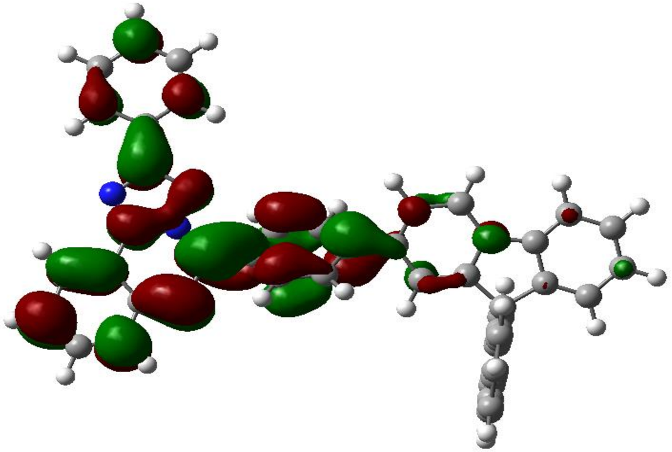	3.89
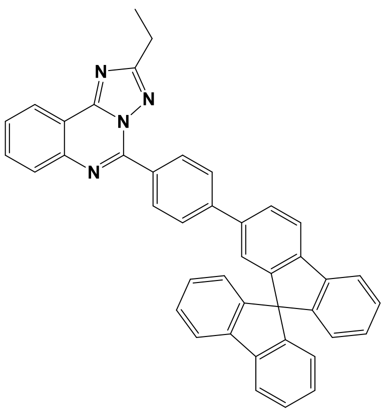 **12b**	-5.61 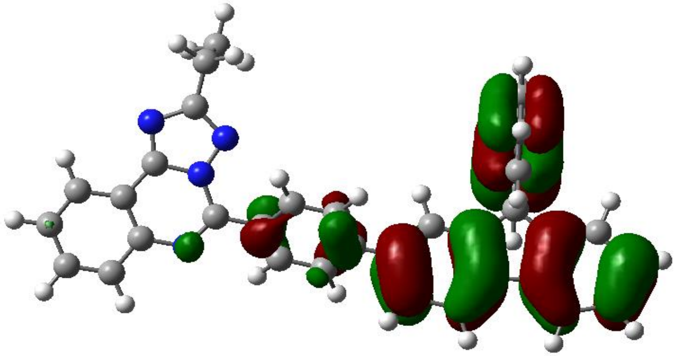	-1.63 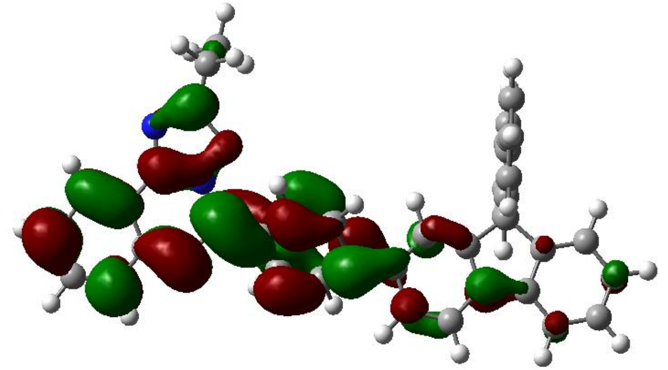	3.98

Table S14. The optimized geometries of **6a-c,e,h,j**, **10**, **11** and **12a,b** in toluene and MeCN

#		S_0	S_1
**6a**	Toluene	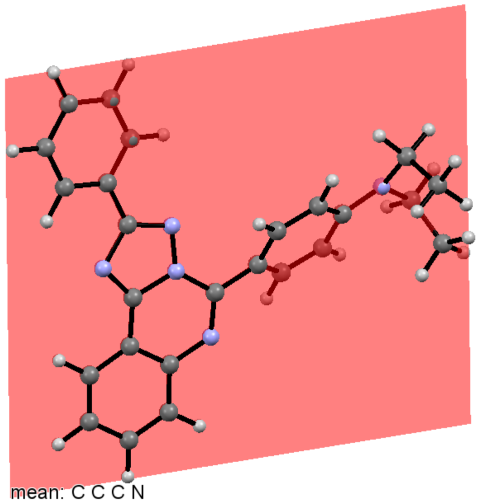	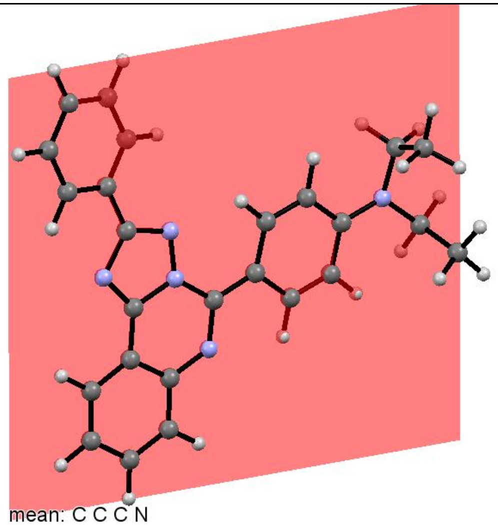
	MeCN	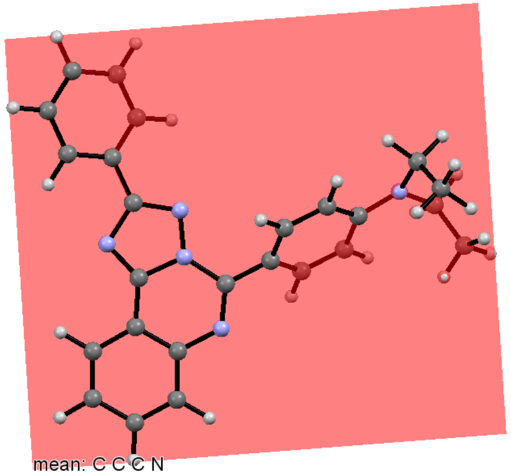	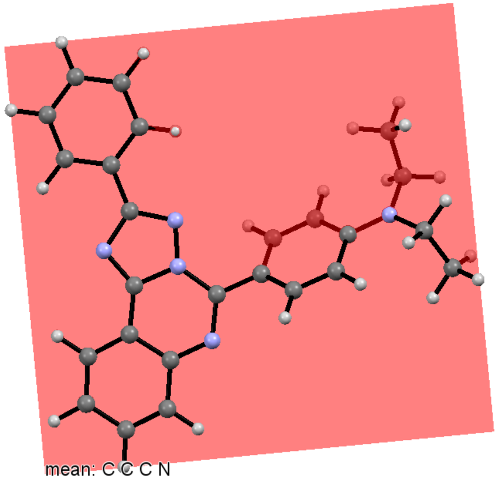

**6b**	Toluene	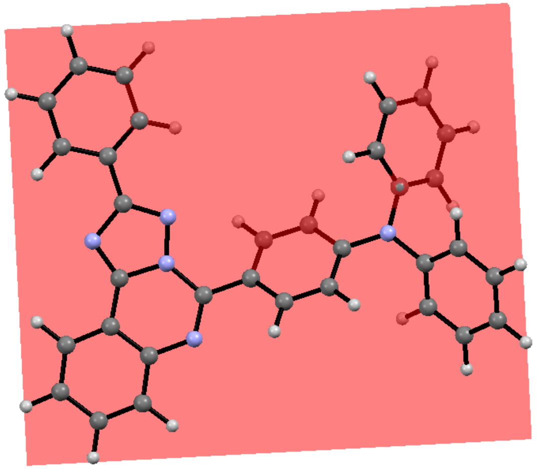 mean: C C C N	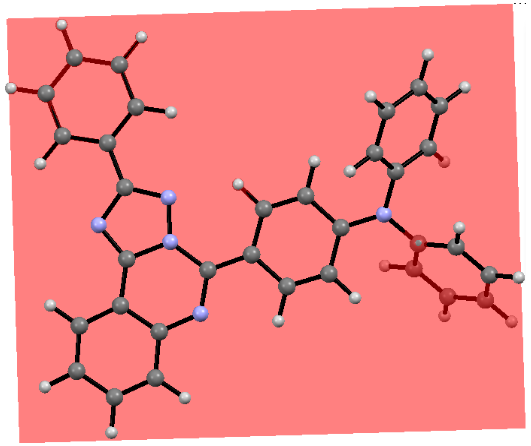 mean: C C C N
	MeCN	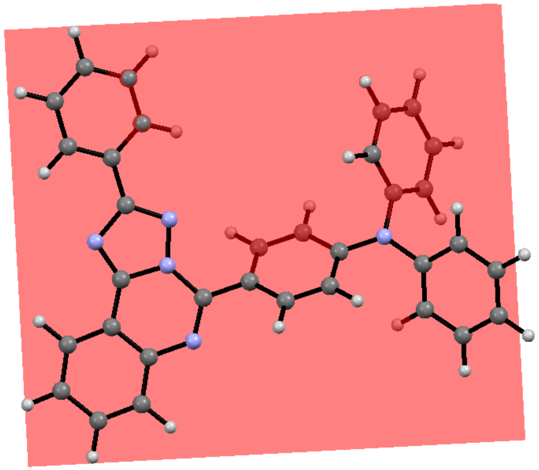 mean: C C C N	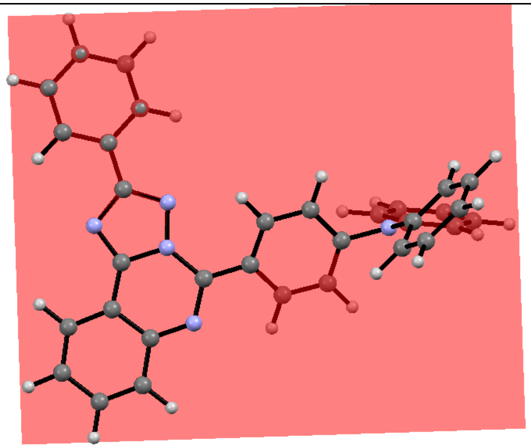 mean: C C C N

**6c**	Toluene	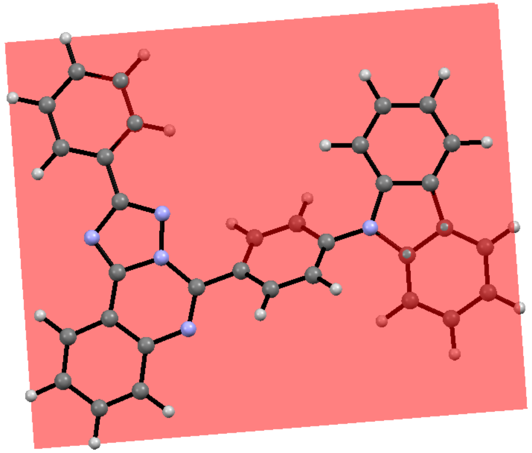 mean: C C C N	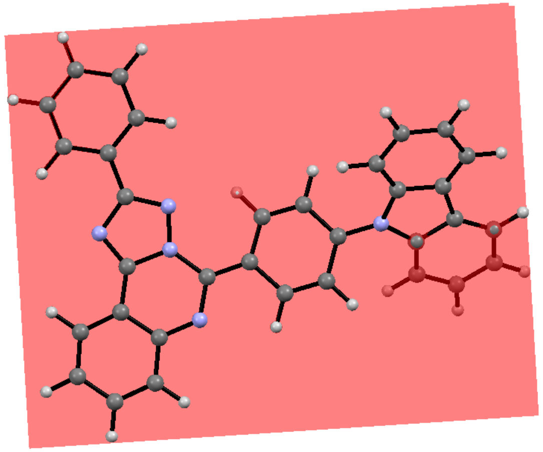 mean: C C C N
	MeCN	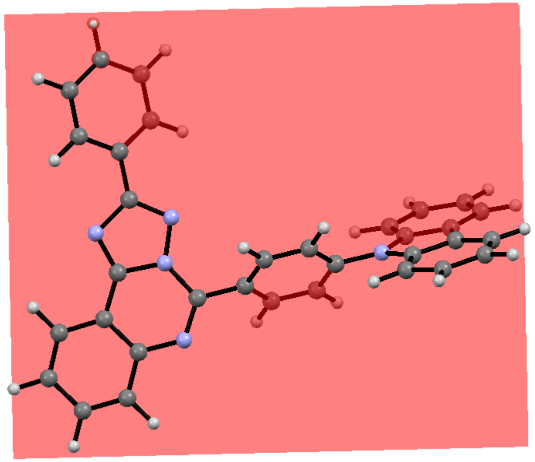 mean: C C C N	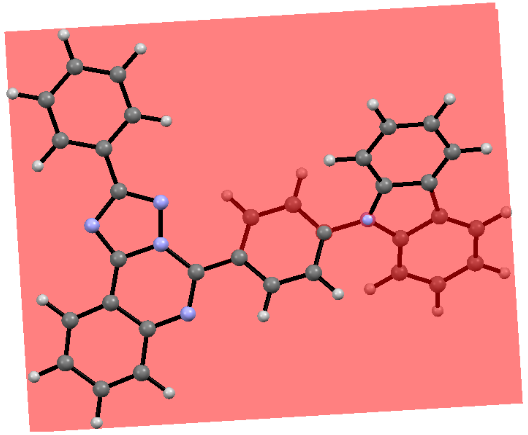 mean: C C C N

<table>
<tr><td rowspan="2">6d</td><td>Toluene</td><td>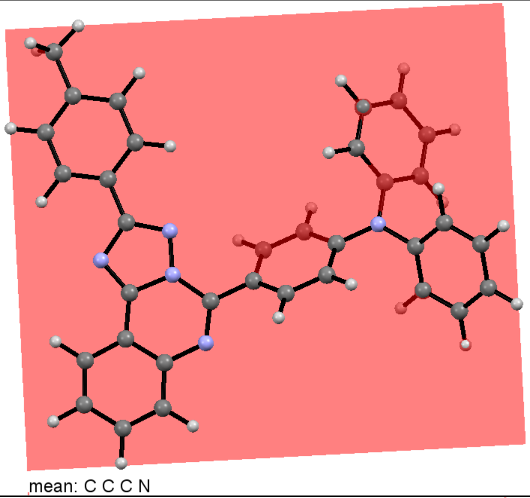</td><td>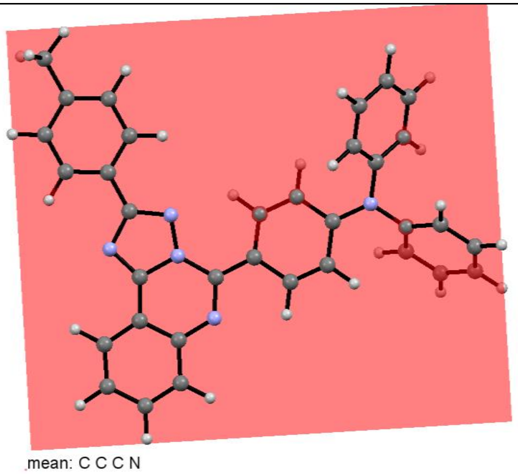</td></tr>
<tr><td>MeCN</td><td>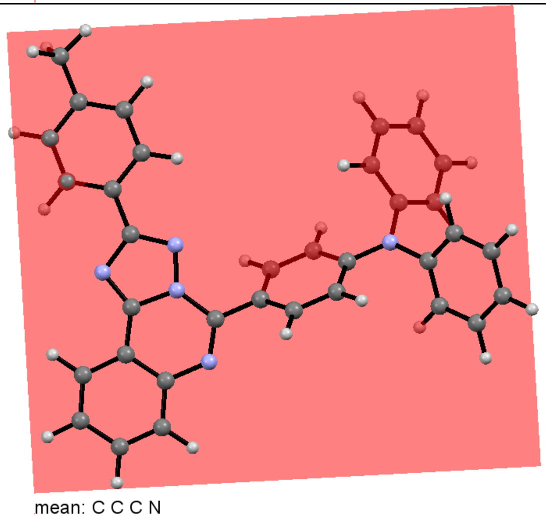</td><td>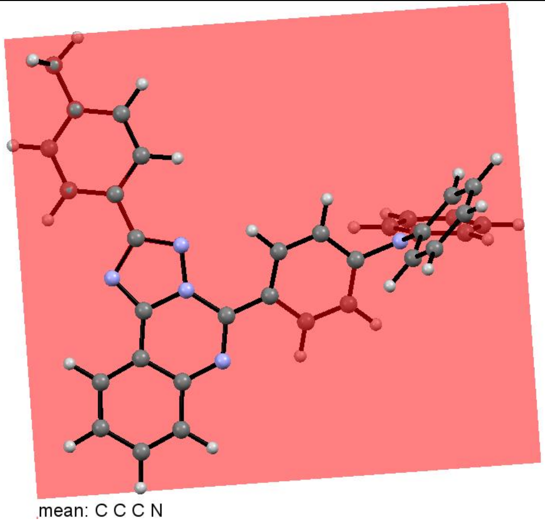</td></tr>
</table>

<table>
<tr><td rowspan="2">6h</td><td>Toluene</td><td>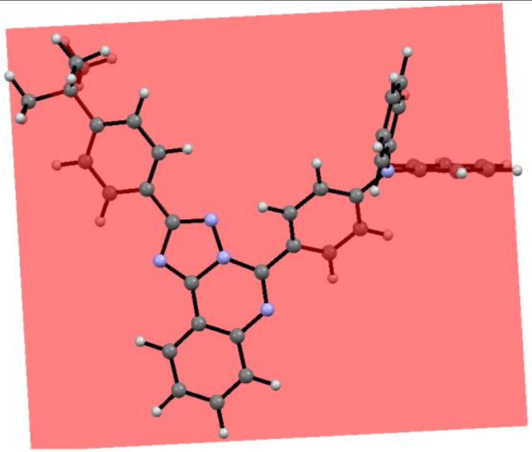
mean: C C C N</td><td>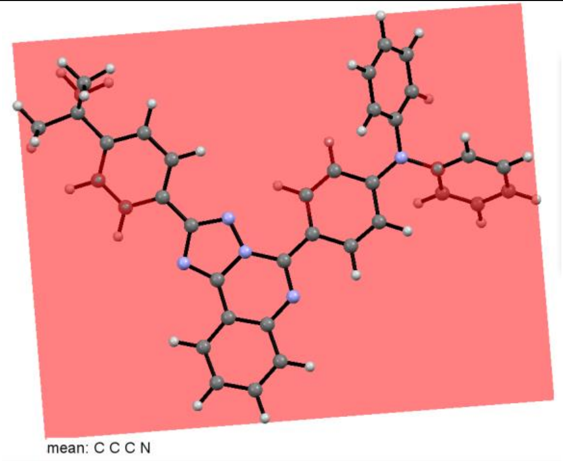
mean: C C C N</td></tr>
<tr><td>MeCN</td><td>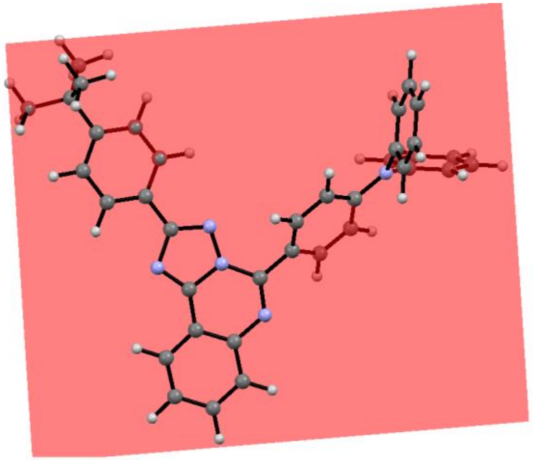
mean: C C C N</td><td>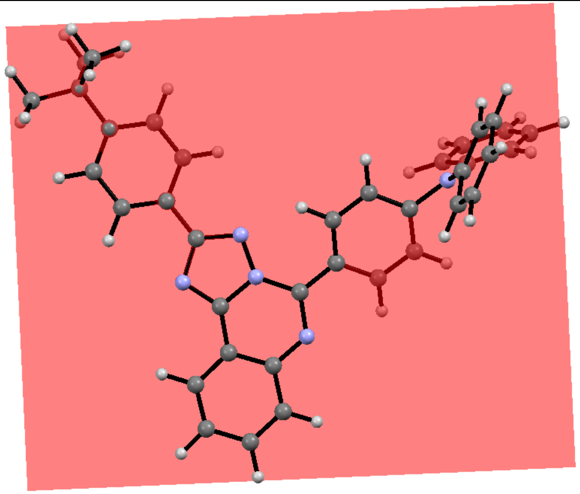
mean: C C C N</td></tr>
</table>

**6j**	Toluene	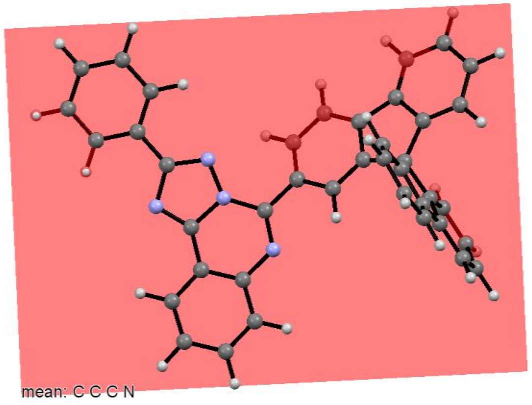	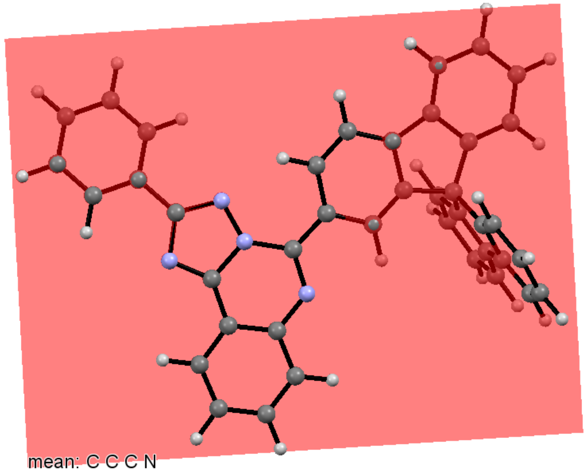
	MeCN	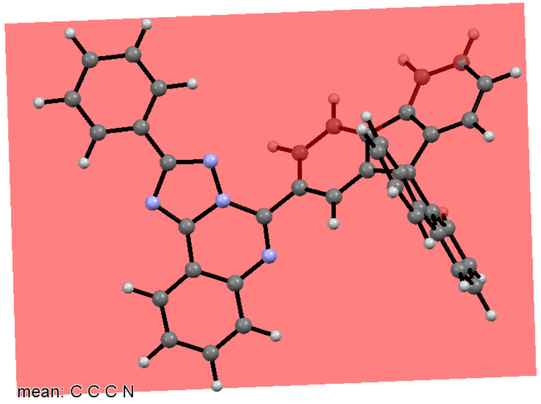	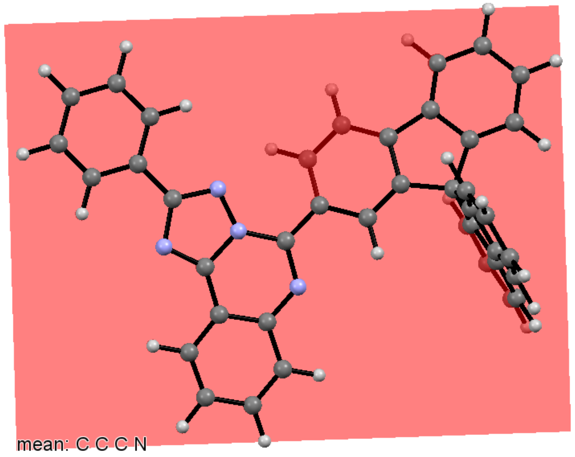

**10**	Toluene	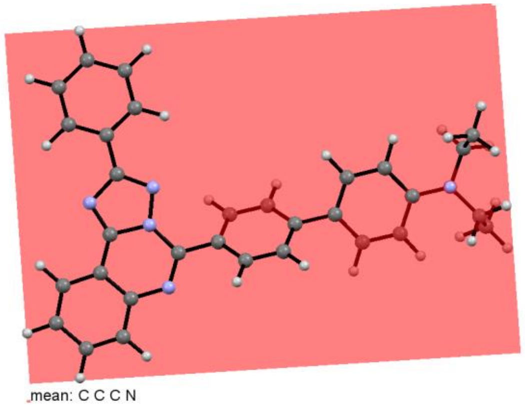	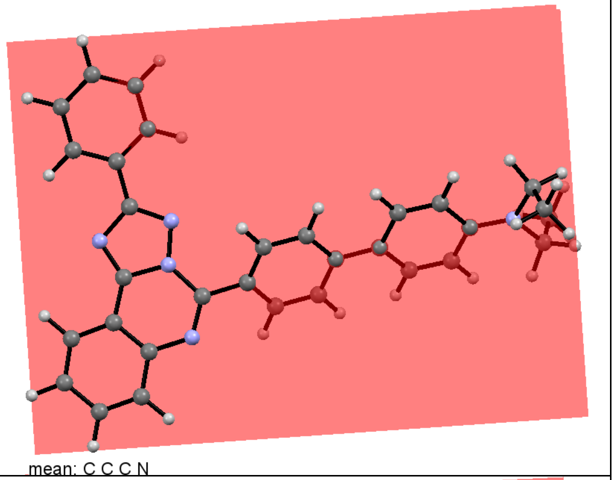
	MeCN	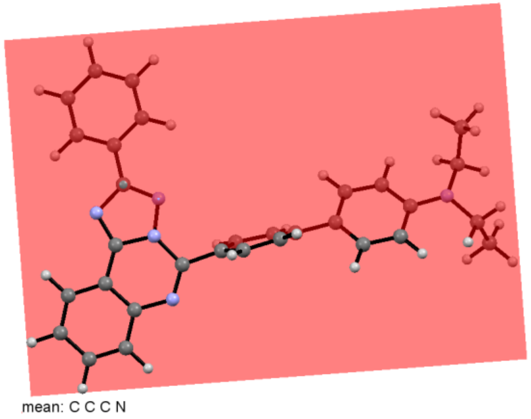	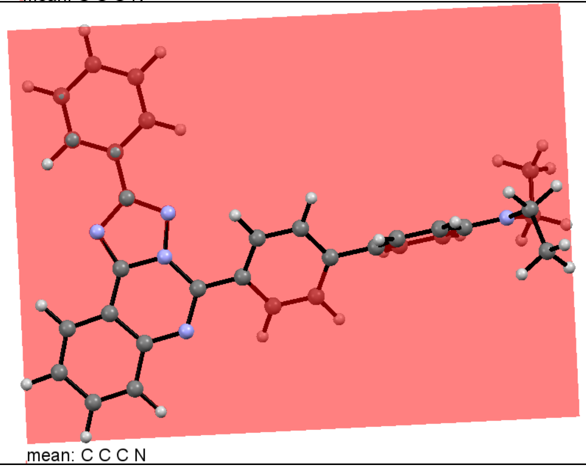

**11**	Toluene	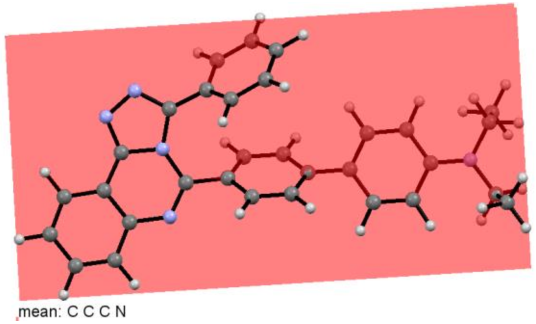	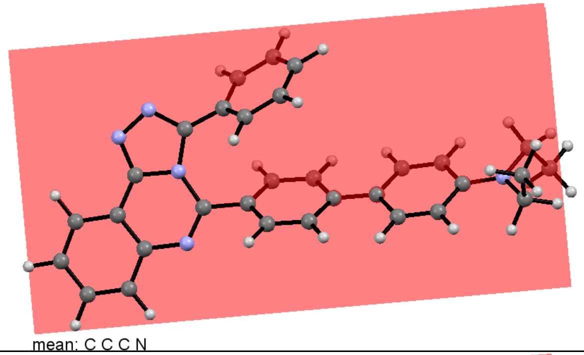
	MeCN	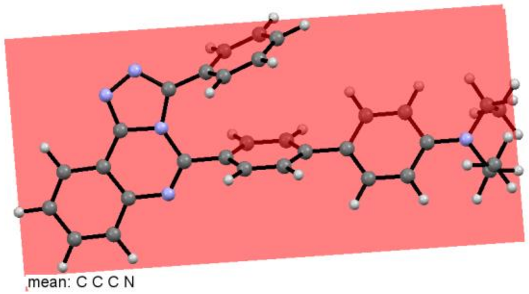	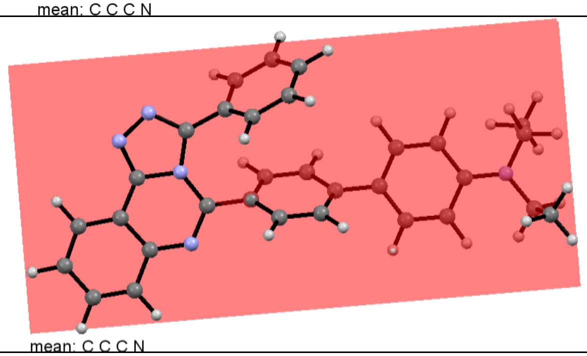
**12a**	Toluene	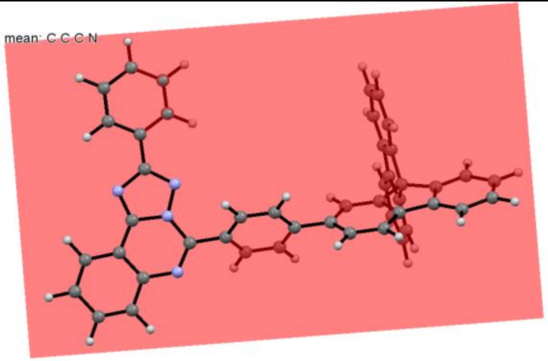	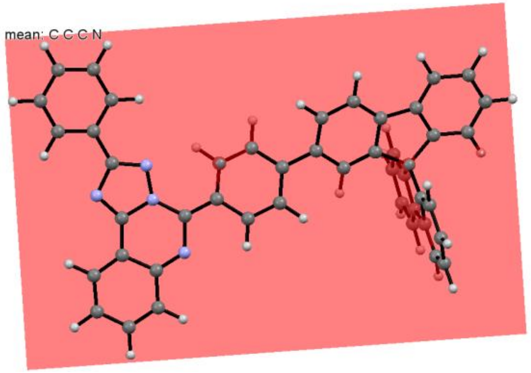

	MeCN	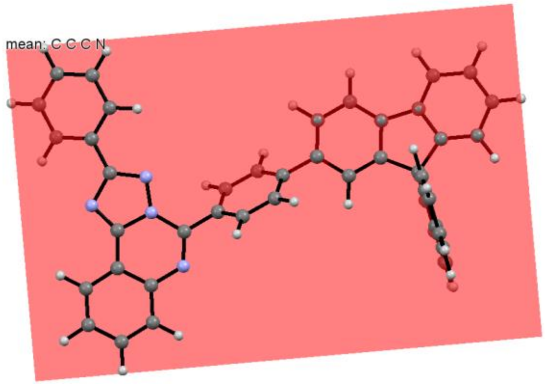	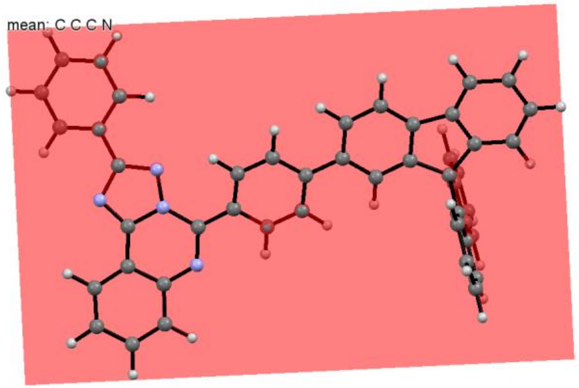
**12b**	Toluene	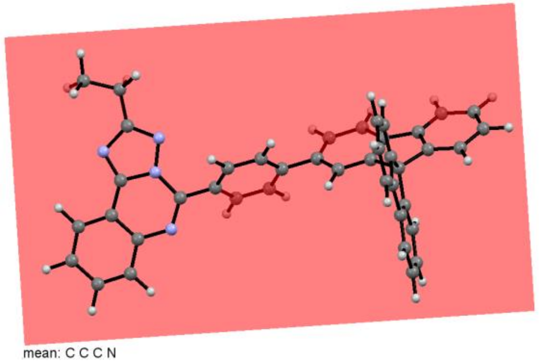	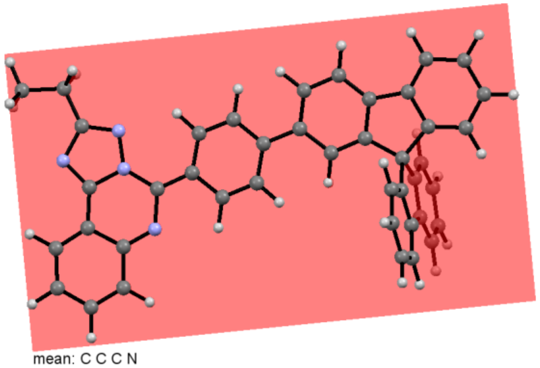
	MeCN	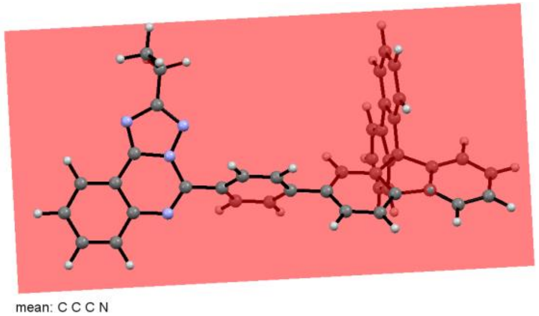	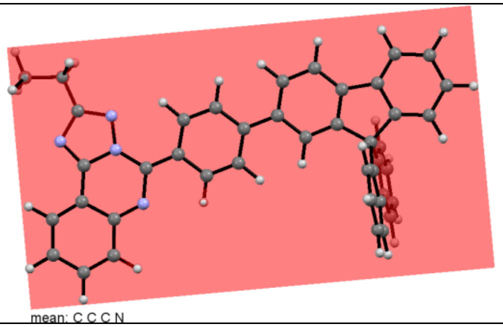

Table S15. Selected dihedral angles (α), and bond lengths (L) in the optimized geometries of compounds **6a-c,e,h,j**, **10**, **11** and **12a,b**, in ground (S_0) and excited (S_1) states. The dihedral angles (α) are given in the 0–90° range as absolute values to illustrate deviations from planarity.

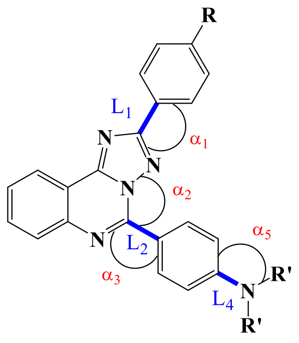

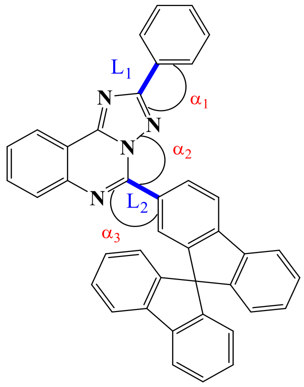

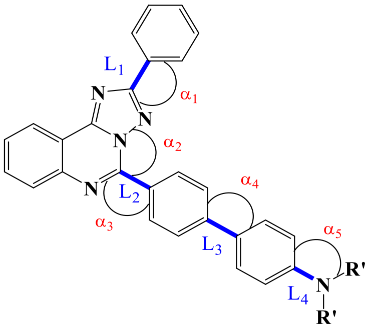

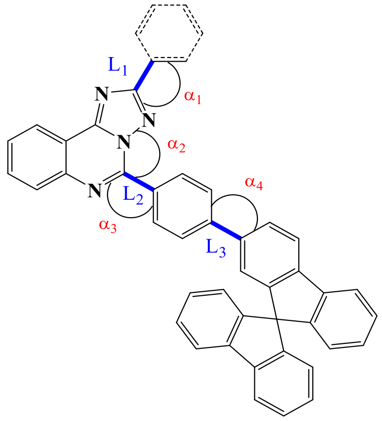

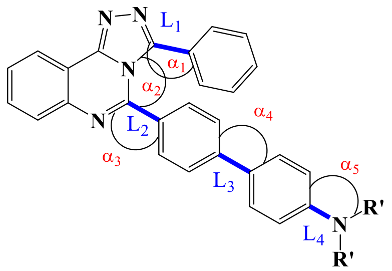

6a-c,e,h **6j** **10** **11**

12a,b

Comp.	Solvent	S	α1, °	α2, °	α3, °	α4, °	α5, °	L1, Å	L2, Å	L3, Å	L4, Å
**6a**	Толуол	S_0	9.4	0.1	34.5	-	14.1	1.465	1.463	-	1.396
		S_1	12.1	1.8	3.6	-	7.5	1.465	1.418	-	1.376
	MeCN	S_0	19.7	0.6	**46.8**	-	12.5	1.465	1.461	-	1.384
		S_1	6.9	3.3	**49.8**	-	0.9	1.463	1.466	-	1.377
**6b**	Толуол	S_0	7.5	0.0	40.4	-	22.7	1.465	1.469	-	1.415
		S_1	0.6	0.3	1.2	-	26.2	1.465	1.426	-	1.384
	MeCN	S_0	9.7	0.5	45.9	-	22.5	1.465	1.467	-	1.411
		S_1	3.4	3.3	21.2	-	68.2	1.464	1.441	-	1.457
**6c**	Толуол	S_0	5.4	0.4	37.5	-	45.5	1.465	1.476	-	1.430
		S_1	0.7	0.7	1.5	-	38.0	1.464	1.410	-	1.394
	MeCN	S_0	21.4	1.3	55.0	-	45.3	1.465	1.476	-	1.431
		S_1	6.2	4.5	24.2	-	60.3	1.464	1.443	-	1.438
**6e**	Толуол	S_0	4.2	1.4	40.8	-	24.6	1.462	1.469	-	1.418
		S_1	4.9	1.0	8.8	-	26.0	1.462	1.429	-	1.384
	MeCN	S_0	16.3	1.4	50.2	-	18.4	1.461	1.467	-	1.408
		S_1	5.9	2.5	20.5	-	68.3	1.461	1.440	-	1.457
**6h**	Толуол	S_0	13.7	1.0	38.1	-	20.9	1.463	1.469	-	1.415
		S_1	12.2	1.1	5.5	-	26.6	1.463	1.428	-	1.384
	MeCN	S_0	9.0	0.6	44.3	-	15.8	1.462	1.467	-	1.407
		S_1	10.4	2.5	20.3	-	69.5	1.462	1.440	-	1.458

**6j**	Толуол	S_0	5.0	0.3	38.6	-	-	1.465	1.479	-	-
		S_1	5.2	3.7	12.5	-	-	1.464	1.428	-	-
	MeCN	S_0	1.8	2.3	51.2	-	-	1.464	1.479	-	-
		S_1	4.6	2.6	20.1	-	-	1.463	1.439	-	-
**10**	Толуол	S_0	3.5	1.7	38.5	48.8	0.4	1.465	1.475	1.472	1.391
		S_1	6.0	0.6	39.3	3.0	4.5	1.465	1.472	1.423	1.375
	MeCN	S_0	13.4	10.4	79.2	**47.0**	3.4	1.477	1.497	1.514	1.389
		S_1	4.1	4.4	24.3	**57.7**	0.6	1.463	1.448	1.476	1.375
**11**	Толуол	S_0	64.3	3.1	63.4	46.7	0.3	1.464	1.478	1.472	1.390
		S_1	59.0	3.2	55.5	4.6	2.4	1.464	1.476	1.424	1.375
	MeCN	S_0	84.3	0.7	84.0	**43.2**	1.0	1.464	1.478	1.470	1.385
		S_1	66.6	8.2	64.1	**46.5**	0.2	1.464	1.468	1.472	1.373
**12a**	Толуол	S_0	5.6	1.4	48.9	64.3	-	1.465	1.478	1.481	-
		S_1	4.8	1.1	11.3	34.8	-	1.464	1.420	1.469	-
	MeCN	S_0	18.9	1.1	55.5	51.2	-	1.464	1.478	1.480	-
		S_1	4.8	5.7	18.7	1.7	-	1.463	1.439	1.465	-
**12b**	Толуол	S_0	-	0.5	61.0	63.1	-	-	1.476	1.480	-
		S_1	-	1.8	3.3	13.7	-	-	1.416	1.464	-
	MeCN	S_0	-	0.1	63.7	51.2	-	-	1.479	1.481	-
		S_1	-	6.4	7.6	0.3	-	-	1.418	1.463	-

1. Blank, J.; Kandt, M.; Pfeiffer, W.-D.; Hetzheim, A.; Langer, P. Domino Cyclization of 2-Isothiocyanatobenzonitrile with Carboxylic Hydrazides − One-Pot Synthesis of 1,2,4-Triazolo[1,5-c]Quinazoline-5(6H)-Thiones. *European J. Org. Chem.* **2003**, *2003*, 182–189, doi:10.1002/1099-0690(200301)2003:1<182::AID-EJOC182>3.0.CO;2-S.

2. Mamedov, V.A.; Zhukova, N.A.; Kadyrova, M.S. The Dimroth Rearrangement in the Synthesis of Condensed Pyrimidines – Structural Analogs of Antiviral Compounds. *Chem. Heterocycl. Compd.* **2021**, *57*, 342–368, doi:10.1007/s10593-021-02913-7.

3. Ravi, M.; Samanta, A.; Radhakrishnan, T.P. Excited State Dipole Moments from an Efficient Analysis of Solvatochromic Stokes Shift Data. *J. Phys. Chem.* **1994**, *98*, 9133–9136, doi:10.1021/j100088a007.

4. Reichardt, C. Solvatochromic Dyes as Solvent Polarity Indicators. *Chem. Rev.* **1994**, *94*, 2319–2358.

5. Thiaré, D.D.; Khonté, A.; Diop, A.; Cissé, L.; Coly, A.; Tine, A.; Delattre, F. Determination of Ground and Excited State Dipole Moments of Amino-Benzimidazole by Solvatochromic Shift Methods and Theoretical Calculations. *J. Mol. Liq.* **2015**, *211*, 640–646, doi:10.1016/j.molliq.2015.07.071.
